# Longitudinal association between lifetime workforce participation and risk of self-reported cognitive decline in community-dwelling older adults

**DOI:** 10.1371/journal.pone.0234392

**Published:** 2020-06-08

**Authors:** Kimiko Tomioka, Norio Kurumatani, Keigo Saeki

**Affiliations:** Nara Prefectural Health Research Center, Nara Medical University, Kashihara, Japan; University of Indianapolis, UNITED STATES

## Abstract

**Background:**

Although many governments are promoting workforce participation (WP) by older people, evidence of WP’s effects on active aging is inadequate. We examined whether there is a gender-specific beneficial effect of lifetime WP from adulthood though old age against self-reported cognitive decline (CD) among community-dwelling older adults.

**Methods:**

We used data from a community-based prospective study of 2,422 men and 2,852 women aged ≥65 with neither poor cognition nor disability in basic activities of daily living at baseline. Self-reported CD was measured using the Cognitive Performance Scale. Lifetime WP evaluated the presence or absence of WP at baseline, the longest-held occupation, and lifetime working years (total working years throughout lifetime). Generalized estimating equations of the multivariable Poisson regression model were applied to evaluate a cumulative incidence ratio (CIR) for self-reported CD and a 95% confidence interval (CI), controlled for age, education, self-perceived economic status, chronic medical conditions, smoking history, physical activity, depression, and instrumental activities of daily living. To examine any gender-specific association, stratified analyses by gender were performed.

**Results:**

The 33-month cumulative incidence of self-reported CD was 15.7% in men and 14.4% in women. After covariate adjustments and mutual adjustment for three items of lifetime WP, men who had their longest held job in a white-collar occupation reported significantly decreased self-reported CD compared to men engaged in blue-collar jobs (CIR 0.72; 95% CI, 0.57–0.91), and women had a significant dose–response relationship between longer lifetime working years and less decline in subjective cognitive functioning (*P* for trend <0.029). Among both genders, WP at baseline was not associated with self-reported CD.

**Conclusions:**

Our results suggest that lifetime WP, especially lifetime principal occupation in men and lifetime working years in women, may play a more prominent role in preventing self-reported CD than later-life WP.

## Introduction

With the arrival of a super-aged society, people have high hopes for workforce participation (WP) by older adults [1]. Reflecting current demographic changes in society, the number of older people who work beyond age 65 has increased; in Japan, the proportion of people aged 65 and above in the total labor force increased from 4.9% in 1980 to 12.4% in 2017 [2]. However, we have insufficient evidence of the effects on health of working in later life. This lack of evidence may be explained as follows: First, when people previously reached retirement age, they generally retired on a pension. Therefore, evidence regarding the health effects of working/retirement are mostly based on the productive population [3–5]. Second, health indicators have generational differences. For working-age people it is about prevention of lifestyle-related diseases; for older adults, the focus is on prevention of functional decline [6]. Because the increase in the number of older people needing support places a heavy burden on the social security system [7], there is an urgent need for us to examine whether WP in later age may be associated with nursing care prevention.

Numerous studies have cited cognitive impairment/dementia as a leading cause of long-term nursing care need [8,9]. Because the prevalence of cognitive decline (CD) shows a clear increase with age [10], as society ages, the number of cognitively impaired older adults will also continue to increase. Therefore, it is critical to investigate whether WP by older people may protect against CD.

Some previous studies report that paid employment in later life had a positive effect on mental health and functional capacity [11–15]. Fujiwara et al. investigated the influence of working at an older age on basic activities of daily living among Japanese adults aged 65 years or older, and found that engaging in paid work was related to a protective effect against functional decline among men but not women [11]. Fujiwara’s study indicates gender differences in the health effects of WP among older adults. Additionally, there is epidemiological evidence that the lifetime principal occupation, which is defined as the longest held occupation in a lifetime, is related to the subsequent risk for Alzheimer’s disease and dementia among community-dwelling older adults [16], and that midlife occupational activities may affect health in old age [17–21]. Recent life-course research reported that men and women who engaged in paid work after state pension age tended to have better health at follow-up, but when life-course labor market participation was accounted for, the health benefits of extending one’s working life were no longer significant [22]. This study suggests that lifetime work histories may confound the association between WP later in life and health in old age. Most previous research with positive outcomes of work in later life failed to account for work histories in adulthood [11–15] or gender differences [12,14]. Therefore, in assessing the effects of continuing to work after retirement age on cognitive performance, we need to consider employment experiences in adulthood as well as gender differences.

We therefore examined the longitudinal association between lifetime work experience, including WP in both old-age and midlife, and the risk of CD in community-dwelling older adults, focusing on gender differences.

## Materials and methods

### Study population

The target area for this study was A City in Nara Prefecture in Japan, a commuter town with a lower population aging rate than the national average. The design of this cohort study has been detailed elsewhere [20]. Briefly, baseline and follow-up surveys were conducted in March 2014 and November 2016, respectively. Self-completed questionnaires were distributed to all citizens living in A City who were aged 65 or over as of January 1, 2014; people residing in nursing homes or in a hospital at the time of survey were removed from the distribution list. The exclusion criteria in this study were: 1) people with poor cognitive functioning at baseline, 2) people with dependency in basic activities of daily living at baseline, and/or 3) people with missing data regarding cognitive performance, basic activities of daily living, and/or WP. The assessment of basic activities of daily living is explained in the supplementary information (S1 Text).

**Fig 1:**
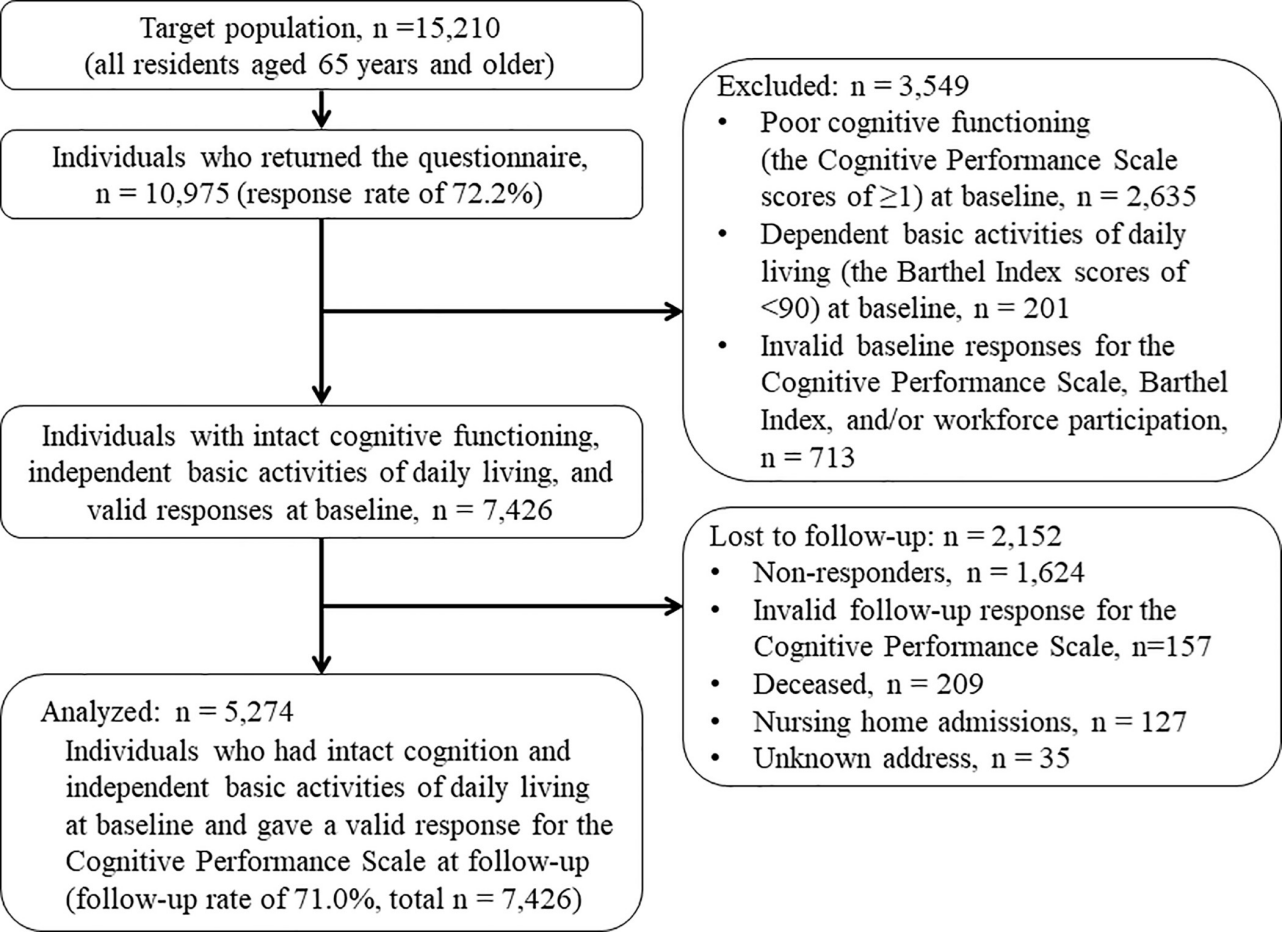
Flow chart of study participants.

Fig 1 displays a flowchart of study participants. The baseline questionnaires were mailed to 15,210 residents aged 65 or older. Of these, 10,975 (72.2%) returned the survey. A total of 3,549 persons were excluded from the follow-up survey because of poor cognition (n = 2,635), dependent basic activities of daily living (n = 201), and missing data (n = 713). A total of 2,152 persons were unsuccessful in follow-up because of non-response (n = 1,624), missing data (n = 157), death (n = 209), institutionalization (n = 127), and unknown addresses (n = 35). Eventually, 5,274 persons were analyzed for the purpose of this study (mean age ± standard deviation: 72.5 ± 5.7; age range: 65–100).

Comparisons of baseline characteristics between analyzed participants and subjects lost to follow-up are presented in S1 Table. Subjects lost to follow-up were significantly older, less likely to be married, get regular exercise, and enter the labor force, as well as being more likely to be underweight, have depression, and have poorer instrumental activities of daily living than participants included in this analysis. However, there was no difference in gender and smoking history between these two groups.

This study was approved by the Nara Medical University Ethics Committee (approval number 939). Submission of self-completed questionnaires was considered agreement to participate in the research. Authors had only accessed to fully anonymized data.

### Assessment of self-reported cognitive decline (CD)

For assessment of self-reported CD, we used the Cognitive Performance Scale [23], which has adequate validity and reliability based on its high correlation with the Mini-Mental State Examination [24]. According to a study testing for validity and reliability, the Japanese version of the Cognitive Performance Scale has been shown to have a Spearman’s correlation coefficient of 0.84 with the Karasawa’s Clinical Evaluation Scale for Geriatric Mental Function and an inter-rater reliability coefficient of 0.77 [25]. To calculate the Cognitive Performance Scale scores, the following items are required [23]: Short-Term Memory (“*Can you recall what was learned or known after 5 minutes*?”), Decision-Making (“*Can you make everyday decisions about tasks or activities of daily living*?”), Understood by Others (“*Can you express or communicate requests*, *needs*, *opinions*, *urgent problems*, *and social conversations*?”), and Activities of Daily Living Self-Performance in Eating (“*Can you eat without staff help/oversight or assistance*?”). The Cognitive Performance Scale has a total score range of 0 to 6, with lower scores indicating a higher level of cognitive functioning. Previous studies have shown that the Cognitive Performance Scale score is highly correlated with objective assessment of cognitive status and can predict dementia risk [26,27]. According to community-based studies in Japan [25,28], individuals aged ≥65 years who have a Cognitive Performance Scale score of 1 are more likely to develop mild cognitive impairment or long-term nursing care need than those with a Cognitive Performance Scale score of 0, suggesting that the cut-off point for screening older adults who are at risk of CD is a Cognitive Performance Scale score of 1 or higher. Based on previous studies [25–28], we dichotomized cognitive status into intact cognition (a Cognitive Performance Scale score of zero) and poor cognitive functioning (a Cognitive Performance Scale score of 1 or higher). To examine the association between lifetime WP and self-reported CD, we excluded persons with poor cognitive functioning at baseline (i.e., those with a Cognitive Performance Scale score of 1 or higher at baseline) from our follow-up study. Therefore, in this study, persons with self-reported CD were defined as individuals whose Cognitive Performance Scale score was 0 at baseline but who had a Cognitive Performance Scale score of 1 or higher at the 33-month follow-up.

### Assessment of lifetime workforce participation (WP)

Lifetime WP assessed the presence or absence of WP at baseline, occupation for the longest held job, and lifetime working years.

#### WP at baseline

We defined subjects with WP in later life as those having paid work at the baseline survey [11, 13–15].The question regarding WP at baseline was, “Do you have a job with income?” and participants were simply asked to answer either “Yes” or “No.”

#### The longest-held occupation and lifetime working years

Research findings to date show that manual work (or blue-collar work) has been associated with an increased risk of developing cognitive impairment [16,29], and that lifetime labor market attachment has a potential effect on the association between later-life WP and later-life health [22]. Therefore, we adopted the longest-held occupation and lifetime working years as lifetime work experience in this study.

The longest-held occupation was categorized as white-collar, pink-collar, blue-collar, and other (i.e., other unclassified occupations and persons without work experience) [30]. Details of the assessment of the longest-held occupation are described in the supplementary information (S1 Text).

For lifetime working years, participants were asked about the total number of years that they had worked; 4 years or less, 5–14 years, 15–24 years, and 25 years or over. Because only a few answered “24 years or less” among men, when analyzing the data for men, we categorized lifetime working years as short (0–24 years) and long (>25 years). In contrast, because women had a diversity of lifetime working years, when analyzing the data for women, we classified lifetime working years into four categories (i.e., 0–4 years, 5–14 years, 15–24 years, and ≥25 years), and examined the dose-response relationship between levels of lifetime working years and self-reported CD among women: *P* for trend was computed by entering the categories as a continuous term (score variable: 1, 2, 3, 4) in the generalized estimating equations of the Poisson regression model.

### Covariates

Based on previous studies [29–32], socio-demographic factors (age, education, self-perceived economic status, and chronic medical conditions), lifestyle habits (smoking history and physical activity), and mental and physical health (depression and instrumental activities of daily living) were selected as covariates that may be important confounders of the association between lifetime WP and self-reported CD. Information about gender and age was provided by the municipality, and data on other covariates were drawn from the questionnaire.

Age was classified into 65–69 years, 70–74 years, 75–79 years, and ≥80 years. Educational attainment (i.e., years of formal education) was split into ≥12 years and <12 years. For self-perceived economic status, we asked respondents how they felt about their current financial state, giving them four answers to choose from: “very well off,” “somewhat well off,” “somewhat poor,” and “very poor”. Self-perceived economic status was categorized as well off (very/somewhat well off) and poor (very/somewhat poor). Chronic medical conditions included hypertension, diabetes mellitus, heart disease, and cerebrovascular disease, because these four diseases are reported to be associated with cognitive decline among community-dwelling older adults [31]. Subjects were asked if they were currently under medical treatment for hypertension, diabetes mellitus, heart disease, or cerebrovascular disease. Respondents selected “yes” or “no” for each illness. The number of chronic medical conditions under medical treatment was categorized as none, one, and ≥2. Smoking history was split into never-smokers and ex/current smokers. Physical activity was categorized as active (i.e., individuals with regular exercise once per month or more) and inactive (i.e., those with exercise less than once per month). Depression was assessed using the five-item short form of the Geriatric Depression Scale [33]. The five-item Geriatric Depression Scale has established its validity and reliability; the scoring ranges from 0 to 5, with higher scores representing a higher level of depression. Individuals with scores of ≥2 were categorized as being depressed. Instrumental activities of daily living were measured by using five items from the Tokyo Metropolitan Institute of Gerontology Index of Competence [34]. The Tokyo Metropolitan Institute of Gerontology Index of Competence is a commonly used standard scale to assess higher-level functional capacity in Japan and its validity and reliability have been confirmed in previous research [35]; the scoring ranges from 0 to 5, with higher scores representing a higher level of competency. Individuals with a perfect score of 5 points were classified as having independent instrumental activities of daily living, while those with scores of ≤4 were classified as having poor instrumental activities of daily living.

Regarding multicollinearity of the variables about covariates, we checked that none of the variables had >5.0 of the variance inflation factor and there was no trouble with multicollinearity in the variables used for covariates.

In this study, 914 persons (17.3% of analyzed participants) had at least one missing covariate data. Therefore, regarding missing covariate data, we carried out multiple imputations by chained equations [36]. Details of multiple imputations are described in the supplementary information (S1 Text).

### Statistical analyses

We used the generalized estimating equations of the multivariable Poisson regression model and calculated a cumulative incidence ratio (CIR) for self-reported CD and a 95% confidence interval (CI). The independent variables were WP at baseline, occupation for the longest held job, and lifetime working years. First, we calculated a crude CIR for self-reported CD. Next, the age-adjusted CIR was calculated (Model 1). Subsequently, in Model 2, all covariates (i.e., socio-demographic factors, lifestyle habits, and mental and physical health) were added simultaneously. In the final Model 3, WP at baseline, the longest-held occupation, and lifetime working years were added to the variables in Model 2 (i.e., Model 3 was mutually adjusted for three items of lifetime WP, and examined the independent association between each item of lifetime WP and self-reported CD). The analyses were performed according to gender.

Furthermore, the association between lifetime work experience and self-reported CD can be affected by age, education, and physical activity as well as gender [29,31,32]. For example, both the prevalence of CD and the unemployment rate are higher in people aged 75 years or older (the old-old) than in those aged 65 to 74 years (the young-old). Regarding education, it may be a potential cause of WP; education is associated with WP (i.e., longer years of formal education can predict higher levels of occupational class). Regarding physical activity, it may be on the pathway between WP and CD; WP may increase physical activity, and regular physical activity is associated with a reduced risk of CD. Therefore, we performed additional stratified analyses by age, education, and physical activity.

We set a significant level of 0.05 (two-tailed), and used IBM SPSS Statistics (version 24.0; IBM Corp, Armonk, NY, USA) for statistical analyses.

## Results

Of the 5,274 participants, 31.7% were persons aged 75 years or older, and 45.9% were men. The proportion of subjects with paid work at baseline was significantly higher among men than women (male 30.3% vs. female 15.3%, *P* <0.001 by chi-squared test), while the cumulative incidence of self-reported CD during the 33-month follow-up showed no gender difference (male 15.7% vs. female 14.4%, *P* = 0.189 by chi-squared test). Baseline characteristics of analyzed participants by gender are presented in S2 Table.

Table 1 shows the characteristics of participants with or without self-reported CD by gender. Irrespective of gender, persons with self-reported CD were more likely to be older, have poorer levels of education and economic status, feel more depressed, have poorer instrumental activities of daily living, were less likely to be currently employed at baseline, and have a shorter work history, compared to those without self-reported CD. Men with self-reported CD were more likely to have chronic medical conditions, physical inactivity, and blue-collar work experience than men without self-reported CD.

**Table 1:** Characteristics of participants with or without self-reported cognitive decline by gender.

Baseline Characteristics	Men (n = 2,422)					Women (n = 2,852)				
	No decline		Decline		*P*^a^	No decline		Decline		*P*^a^
	(n = 2,041)		(n = 381)			(n = 2,441)		(n = 411)		
Age: ≥75 years	534	(26.2)	197	(51.7)	<0.001	702	(28.8)	240	(58.4)	<0.001
Educational attainment: <l2 years	398	(19.5)	121	(31.8)	<0.001	552	(22.6)	160	(38.9)	<0.001
Self-perceived economic status: poor	1,072	(52.5)	228	(59.8)	0.010	1,295	(53.1)	248	(60.3)	0.006
Number of chronic medical conditions^b^^:^≥2	292	(14.3)	72	(18.9)	0.024	172	(7.0)	38	(9.2)	0.125
Smoking history: ex/current smokers	1,431	(70.1)	266	(69.8)	0.951	189	(7.7)	37	(9.0)	0.430
Physical activity: inactive	1,243	(60.9)	265	(69.6)	0.002	1,596	(65.4)	280	(68.1)	0.286
Persons with depression	250	(12.2)	103	(27.0)	<0.001	386	(15.8)	147	(35.8)	<0.001
Instrumental activities of daily living: poor	215	(10.5)	93	(24.4)	<0.001	43	(1.8)	32	(7.8)	<0.001
Workforce participation at baseline: non-participation	1,388	(68.0)	300	(78.7)	<0.001	2,043	(83.7)	373	(90.8)	<0.001
Blue-collar occupation for the longest-held job^c^	403	(19.7)	114	(29.9)	<0.001	496	(20.3)	93	(22.6)	0.292
Lifetime working years: ≤25 years	115	(5.6)	37	(9.7)	0.003	1,540	(63.1)	287	(69.8)	0.009

Data are given as n (%).

^a^Differences between subjects with or without decline were analyzed using the chi-squared test.

^b^Chronic medical conditions included hypertension, diabetes mellitus, heart disease, and cerebrovascular disease.

^c^Blue-collar included manufacturing, transport, maintenance, construction, mining, security, agriculture, forestry, fishery, delivery, cleaning, and packing workers.

Table 2 shows the CIRs for self-reported CD associated with lifetime WP. Among both men and women, in the crude model, participants with WP at baseline tended to have a lower risk of self-reported CD compared to non-participants. However, after age adjustment (Model 1), these associations were insignificant. After adjustment for all covariates (Model 2), adjusted CIR (95% CI) of participants with WP at baseline was 0.92 (0.73–1.17) in men and 0.81 (0.59–1.12) in women, compared to non-participants. Regarding the longest-held occupation, among men, even after adjustment for all covariates (Model 2), participants engaged in white-collar jobs had a significantly lower CIR for self-reported CD than blue-collar workers (adjusted CIR 0.71; 95%CI, 0.57–0.90). Among women, in the crude model, persons engaged in pink-collar jobs were less likely to have self-reported CD than participants engaged in blue-collar jobs, but this association was not seen after age adjustment (Model 1). Regarding lifetime working years, among both genders, lifetime working years of 25 years or more were associated with a decreased risk of self-reported CD, even after adjustment for all covariates (Model 2). Adjusted CIR (95% CI) of ≥25 lifetime working years was 0.74 (0.56–0.99) in men and 0.72 (0.57–0.90) in women. Additionally, among women, there was a significant dose-response relationship between longer lifetime working years and lower self-reported CD (*P* for trend = 0.001).

**Table 2:** Cumulative incidence ratio (95% confidence interval) for 33-month cognitive decline associated with lifetime workforce participation, stratified by gender.

		n	Model 0:	Model 1:	Model 2^a^:	Model 3^b^:
			Crude model	Age-adjusted model	Covariates-adjusted model	Mutually adjusted model
Men						
Workforce participation at baseline						
	Non-participation	1,688	1.00	1.00	1.00	1.00
	Participation	734	***0*.*62 (0*.*49–0*.*78)***	0.84 (0.67–1.06)	0.92 (0.73–1.17)	0.92 (0.73–1.16)
The longest-held occupation						
	Blue-collar	517	1.00	1.00	1.00	1.00
	White-collar	1,152	***0*.*59 (0*.*47–0*.*74)***	***0*.*64 (0*.*52–0*.*79)***	***0*.*71 (0*.*57–0*.*90)***	***0*.*72 (0*.*57–0*.*91)***
	Pink-collar	636	***0*.*64 (0*.*49–0*.*82)***	**0.73 (0.57–0.94)**	0.82 (0.63–1.06)	0.82 (0.63–1.07)
	Other	117	1.09 (0.76–1.56)	1.02 (0.71–1.45)	1.13 (0.80–1.59)	1.05 (0.72–1.53)
Lifetime working years						
	Short: 0–24 years	152	1.00	1.00	1.00	1.00
	Long: ≥25 years	2,270	***0*.*62 (0*.*46–0*.*84)***	***0*.*74 (0*.*55–1*.*00)***	***0*.*74 (0*.*56–0*.*99)***	0.83 (0.60–1.14)
Women						
Workforce participation at baseline						
	Non-participation	2,416	1.00	1.00	1.00	1.00
	Participation	436	***0*.*56 (0*.*41–0*.*78)***	0.74 (0.54–1.02)	0.81 (0.59–1.12)	0.89 (0.65–1.23)
The longest-held occupation						
	Blue-collar	589	1.00	1.00	1.00	1.00
	White-collar	402	0.82 (0.60–1.12)	0.79 (0.58–1.07)	0.97 (0.72–1.31)	1.02 (0.75–1.38)
	Pink-collar	1,408	***0*.*77 (0*.*61–0*.*97)***	0.81 (0.64–1.02)	0.97 (0.77–1.22)	0.96 (0.76–1.22)
	Other	453	***1*.*33 (1*.*03–1*.*72)***	1.14 (0.88–1.47)	1.27 (0.99–1.63)	1.21 (0.91–1.60)
Lifetime working years						
	0–4 years	635	1.00	1.00	1.00	1.00
	5–14 years	682	0.87 (0.68–1.10)	1.01 (0.80–1.28)	0.99 (0.78–1.24)	1.09 (0.84–1.42)
	15–24 years	510	0.76 (0.58–1.00)	0.84 (0.64–1.09)	0.81 (0.63–1.06)	0.91 (0.67–1.22)
	≥25 years	1,025	**0.68 (0.54–0.86)**	**0.75 (0.60–0.95)**	***0*.*72 (0*.*57–0*.*90)***	0.81 (0.62–1.06)
	*P* for trend		0.001	0.005	0.001	0.029

Bold italic values highlight statistical significance (*P* <0.05).

^a^Adjusted for age, education, self-perceived economic status, chronic medical conditions, smoking history, physical activity, depression, and instrumental activities of daily living.

^b^In addition to Model 2, workforce participation at baseline, the longest-held occupation, and lifetime working years were included (i.e., Model 3 was mutually adjusted for three items of lifetime workforce participation).

In the final model (Model 3), where the data were mutually adjusted for all items of lifetime WP besides adjustment for all covariates, among men, lifetime working years of 25 years or more lost its significance (adjusted CIR 0.83; 95% CI, 0.60–1.14), while mutual adjustments did not alter a significant association between white-collar occupations and the prevention of self-reported CD (adjusted CIR 0.72; 95% CI, 0.57–0.91), suggesting that the longest-held occupation is an independent predictor of self-reported CD among older adult males. Among women, even after mutual adjustments (Model 3), a dose–response relationship between longer lifetime working years and lower risk of self-reported CD remained significant (*P* for trend = 0.029), suggesting that lifetime working years independently predict self-reported CD among older adult females.

For the effect of multiple imputations, we conducted a comparison between the findings obtained from multiple imputation data and those from the complete data (results are provided in S3 Table); the two indicated broadly similar results, and our findings had little effect on multiple imputations.

In addition, we conducted the analysis of covariance (ANCOVA) using the total Cognitive Performance Scale score, but the results based on the ordinal outcome by the ANCOVA did not substantially change from those based on the dichotomized outcome by Poisson regression models (results are provided in S4 Table).

### Additional analyses

The main results of additional stratified analyses are shown in Table 3 (the detailed results are provided in S5 and S6 Tables). Among men, we found significant inverse associations between white-collar occupations and self-reported CD in people aged 65–74 years, people with high education (≥12 years of education), or people with physical activity, but the association was not significant in people aged ≥75 years, people with low education (<12 years of education), or people without physical activity. Additionally, among men with physical activity, people who engaged in pink-collar occupations tended to have a decreased CIR for self-reported CD than blue-collar workers. Among women, a dose-response relationship between longer lifetime working years and lower risk of self-reported CD was significant in people aged 65–74 years, people with low education, or people without physical activity, but not significant in people aged 75 years or more, people with high education, or people with physical activity.

**Table 3:** Adjusted cumulative incidence ratio for 33-month cognitive decline based on stratified analyses by age, education, and physical activity.

		Stratified by age in years				Stratified by years of education				Stratified by physical activity			
		65–74 years		≥75 years		≥12 years		<12 years		Active		Inactive	
		n	CIR^a^ (95% CI)	n	CIR^a^ (95% CI)	n^b^	CIR^c^ (95% CI)	n^b^	CIR^c^ (95% CI)	n^b^	CIR^c^ (95% CI)	n^b^	CIR^c^ (95% CI)
Men (n = 2,422)													
Occupation for the longest held job													
	Blue-collar	327	1.00	190	1.00	261	1.00	256	1.00	161	1.00	355	1.00
	White-collar	821	***0*.*64 (0*.*45–0*.*91)***	331	0.82 (0.61–1.11)	1,005	***0*.*71 (0*.*52–0*.*96)***	147	0.77 (0.53–1.11)	475	***0*.*57 (0*.*38–0*.*87)***	678	0.77 (0.58–1.02)
	Pink-collar	477	0.75 (0.51–1.11)	159	0.88 (0.62–1.24)	550	0.81 (0.59–1.11)	86	0.79 (0.47–1.32)	242	***0*.*60 (0*.*37–0*.*99)***	394	0.92 (0.68–1.25)
	Other	66	1.03 (0.58–1.82)	51	1.11 (0.68–1.81)	84	0.97 (0.57–1.65)	33	1.17 (0.69–1.99)	37	0.41 (0.15–1.11)	80	1.29 (0.85–1.94)
Women (n = 2,852)													
Lifetime working years													
	0–4 years	382	1.00	253	1.00	504	1.00	131	1.00	216	1.00	419	1.00
	5–14 years	483	0.87 (0.56–1.33)	199	1.27 (0.92–1.76)	531	***1*.*59 (1*.*13–2*.*26)***	151	***0*.*66 (0*.*43–1*.*00)***	243	1.56 (0.96–2.53)	439	0.95 (0.69–1.30)
	15–24 years	344	0.74 (0.46–1.19)	166	1.02 (0.70–1.49)	373	1.29 (0.86–1.93)	137	***0*.*58 (0*.*38–0*.*89)***	186	0.92 (0.51–1.66)	324	0.93 (0.65–1.31)
	≥25 years	701	***0*.*57 (0*.*37–0*.*88)***	324	0.99 (0.71–1.38)	722	1.11 (0.76–1.62)	303	***0*.*54 (0*.*37–0*.*78)***	333	1.11 (0.68–1.82)	692	***0*.*72 (0*.*52–0*.*98)***
	*P* for trend		0.005		0.507		0.692		0.003		0.646		0.025

Results in bold italic indicate *P* <0.05.

CI, confidence interval; CIR, cumulative incidence ratio.

^a^Adjusted for all covariates and three items of lifetime workforce participation (i.e., workforce participation at baseline, the longest-held occupation, and lifetime working years).

^b^The pooled number by multiple imputations.

^c^Adjusted for all covariates excluding the stratifying variables and three items of lifetime workforce participation.

## Discussion

This study examined the association between lifetime WP and risk of self-reported CD among community-dwelling older adults in Japan. The results showed that independent of their other lifetime WP and important confounders including socio-demographic factors, lifestyle habits, and mental and physical health, the longest held occupation in a lifetime was associated with male self-reported CD, and lifetime working years were related to female self-reported CD. Men who engaged in white-collar jobs had a significantly lower risk of self-reported CD compared to men who were blue-collar workers, while women showed a significant dose-response relationship between lifetime working years and self-reported CD, in that as the years of lifetime working experience lengthened, the CIR of female self-reported CD became lower.

According to additional stratified analyses in this study, in both genders, significant results were found only among people aged 65–74 years, suggesting that the age effect in the association between lifetime WP and self-reported CD was stronger in the young-old than in the old-old and had no gender differences. Regarding the effect of factors other than age, in men, significant associations were confirmed only among participants who had high education or those with physical activity, while in women, a significant dose-response relationship was confirmed only among people who had low education or those without physical activity. Previous studies have demonstrated that longer years in education and greater physical activity reduce the risk of CD [29,31,32]. Therefore, in men, the longest held occupation had a greater association with self-reported CD among older adults with low risk of CD, but the female protective effect of longer lifetime working years on incident self-reported CD was stronger in the high-risk group than in the low-risk group. Thus, our findings suggest that lifetime WP, especially lifetime principal occupation in men and lifetime working years in women, may be a stronger predictor of self-reported CD among the young-old people than the old-old people, regardless of gender, while the effect modification by education and physical activity varied according to gender.

It is noted that frequently stimulating the brains of older people can prevent synaptic loss in the nerve pathways, leading to preservation of cognitive functioning [37]. Previous studies reported that later life mental/cognitive activities, such as reading, writing, studying, playing music instruments, doing crossword puzzles, and playing board games or cards, had a protective effect against CD [38,39]. Regarding the mechanism underlying the association between mental activities and cognition, the use-it-or-lose-it hypothesis [40] is commonly accepted. Additionally, a new challenge hypothesis is proposed, which suggests that older people need to deal with new cognitive challenges as well as using their skills and abilities, in order to preserve good cognitive functioning [41]. According to Japanese government data from 2018 [2], for all employees, the percentage of non-regular employees totaled 32.1% for the age group of 45 to 54 and 76.3% for those aged 65 years and over. Older adults are employed primarily in casual, unskilled work, with little responsibility [14]. In this study, among both genders, no association has been recognized between WP at baseline and cognitive functioning. It is possible that working in older age may be boring and not present new cognitive challenges to older adults.

Numerous studies have reported that working in a white-collar job before age 65 is associated beneficially with late-life cognitive functioning [29,32,42]. Past findings, also supported in this study, suggest that older men may be strongly affected by their principal lifetime occupation. This is supported by the concept of “cognitive reserve (CR).” The CR concept suggests that "*individual differences in how people process tasks allow some to cope better than others with brain pathology*" [43] and it is commonly used in referring to “*differences in the flexibility or adaptivity of cognitive networks*” [44]. Previous studies suggest that lifetime exposure to a higher occupational class may enhance CR, leading to the prevention of age-related CD [45]. In particular, the complexity of the work is a critical element in measuring CR and has a great impact on cognition [46]. Stern et al. classified white-collar jobs, including business managers and professionals, as being of high level complexity/an intellectual occupation and reported that higher lifetime occupational level was associated with a reduced risk of CD compared to lower occupational levels [47]. In our study, the majority of older men were white-collar workers, and almost all older men had more than 25 years of working experience. Among men, the low number of older men with less than 25 years of lifetime working years may have a low statistical power and result in no association between lifetime working years and CD, while the degree of work complexity based on the longest-held occupation might make a difference in CR and contribute to the maintenance of cognitive functioning in old age. In contrast, older women made up a majority of pink-collar workers. The small number of female white-collar workers and blue-collar workers may conduce to a low statistical power in detecting differences in CD among the occupational groups. Additionally, the work profile of women was characterized by a diversity of lifetime working years. Among older women, longer lifetime working years may contribute to increasing the CR of female workers and play a significant role in self-reported CD prevention in old age. This hypothesis is supported by a previous study reporting that the amount of time spent in mentally stimulating work is associated with a risk of developing CD and that longer working years might lead to greater CR [48].

There are some limitations to this study. First, as indicated in S1 Table, among the 2,152 subjects who were difficult to follow up, a significantly higher number were 75 years old or older, did less physical activity, had depression, and were not working at baseline compared to the subjects we analyzed. This shows selective absence of a group with a higher risk of CD. It is unclear whether the association between lifetime WP and self-reported CD was overestimated or underestimated. Second, our female findings may possibly apply to a select group of healthy older women who were actively involved with WP in their lifetime. Therefore, it would appear that the female dose-response relationship between longer lifetime working years and less decline in subjective cognitive functioning may have the healthy worker effect. Third, although we excluded older adults who had poor cognitive functioning at baseline from our analyses, our findings suggest the possibility of reverse causality. Because “*withdrawal from work or social activities*” is considered to be one of the 10 warning signs of Alzheimer’s disease [49], community-dwelling older adults with preclinical CD may tend to quit their WP at baseline. Therefore, no association between WP at baseline and self-reported CD in this study may be influenced by reverse causality. Fourth, data resources for this study were based on self-assessment. The absence of objective data can produce misclassification, leading to null association between working and cognitive functioning. Fifth, in this study, lifetime WP evaluated WP at baseline, the longest-held occupation, and lifetime working years. Although WP was measured at baseline, the other two factors were based on retrospective data. Therefore, there is a possibility that the longest-held occupation and lifetime working years have recall bias and variability among study participants. Additionally, we cannot evaluate the latent period between exposure to the longest-held occupation/lifetime working years and incident CD; it is unclear whether the preventive effect against CD is effective for WP in their earlier years or for WP in their later years. Sixth, there is the possibility of unmeasured confounding. For example, management of cardiovascular disease may affect the development of CD [31]. Additionally, many studies have shown that persons with genetic susceptibility genes, such as the apolipoprotein E-ε4 allele, have an increased risk of CD [31,50]. Although our analyses took into consideration important cardiovascular risk factors, including diabetes and hypertension, we had no data on disease control and genetic background. Seventh, our study succeeds in adjusting for important confounders, but there is the possibility of residual confounding. To overcome the influence of confounding bias, further studies are needed which employ instrumental variables such as a propensity score-matched study and a Mendelian randomization analysis [51]. Eighth, in terms of job opportunity and employment structure, many researchers point out that there are cross-country differences and regional differences [3,5,11]. Therefore, any attempt to generalize our findings should be viewed with caution. Ninth, in this study, evaluations were made only in relation to the presence or absence of WP at baseline. It has been suggested that effects on cognitive health differ depending on the specific characteristics of the job, such as complexity, routine, and novelty in work tasks [41], and that working less than 35 hours per week is appropriate for good health in older people [12]. Therefore, it is necessary to clarify the job context and working hours that contribute to prevention of age-related CD. Tenth, recent studies have suggested that the WP-cognition relationship in healthy older people may vary according to cognitive domains [17,39,45]. However, we evaluated only the presence or absence of CD based on the Cognitive Performance Scale. Future studies need to evaluate various cognitive domains. Finally, because the onset of CD generally has a long latent period [31], the 33-month follow-up period in our study may have been too short to confirm a protective effect of lifetime WP on cognition. Additional stratified analyses showed that our findings were applicable to people aged 65–74 years, but not to people aged ≥75 years, suggesting that a bias due to the short observation period for the incidence of self-reported CD may exist in relation to people aged 75 years or older.

Our findings have some practical implications. Many governments, including Japan’s, are forging ahead with the promotion of a society where people can work regardless of age [1]. Our findings suggest that paying attention to lifetime WP, particularly occupations held for the longest time in men and lifetime working years in women, has potential to be more effective in preventing CD than promoting WP by older people. The information in this study may alert public health professionals and policy makers to the possibility that an effective preventive strategy against CD might involve occupational health care to people while they are still young.

## Conclusions

In this study, we have demonstrated that white-collar occupation for the longest held job is associated with the prevention of self-reported CD for men, and that longer lifetime working years have a protective effect on self-reported CD for women. These associations are independent of important confounders including education and other lifetime WP items. For WP at baseline, there is no association with cognitive functioning among both genders. Our findings suggest that not only encouraging older people to engage in work, but also addressing work conditions over the course of their lifetime may be an effective strategy for protection from CD among community-dwelling older people.

## Supporting information

S1 Table Baseline characteristics of analyzed participants and subjects who were lost to follow-up. (PDF)

S2 Table Baseline characteristics of analyzed participants by gender. (PDF)

S3 Table Comparison between the findings obtained from multiple imputation data and those from complete data, based on a mutually adjusted model. (PDF)

S4 Table Association between lifetime workforce participation and the Cognitive Performance Scale score by gender, based on the analysis of covariance (ANCOVA). (PDF)

S5 Table Adjusted cumulative incidence ratio for 33-month cognitive decline based on stratified analyses by age, education, and physical activity among men (n = 2,422). (PDF)

S6 Table Adjusted cumulative incidence ratio for 33-month cognitive decline based on stratified analyses by age, education, and physical activity among women (n = 2,852). (PDF)

S1 Text Detailed explanation of basic activities of daily living, the longest-held occupation, and multiple imputations. (PDF)

S1 File De-identified dataset used for the statistical analyses. (PDF)
