# Supplementary material for: Longitudinal association between lifetime workforce participation and risk of self-reported cognitive decline in community-dwelling older adults
Source: PLoS One. 2020 Jun 8;15(6):e0234392. doi: 10.1371/journal.pone.0234392 (PMC7279604; doi:10.1371/journal.pone.0234392)
Supplement: S1 Table — (PDF) [file pone.0234392.s001.pdf]

S1 Table. Baseline characteristics of analyzed participants and subjects who were lost to follow-up

Baseline characteristics	Analyzed participants n = 5,274	Lost to follow-up n = 2,152	*P*-value[a]
Gender: men	45.9%	45.2%	0.555
Age: ≥75 years	31.7%	38.9%	<0.001
Smoking history: ex/current smokers[b]	38.3%	39.7%	0.280
Physical activity: inactive[c]	64.1%	71.3%	<0.001
Persons with depression[d]	17.7%	22.5%	<0.001
Instrumental activities of daily living: poor[e]	7.7%	9.9%	<0.001
Working status at baseline: non-working	77.8%	80.6%	0.008

[a]Differences between the two groups were analyzed using chi-squared test.
[b]Among individuals with valid response for smoking history: n = 5,025 and n = 2,026.
[c]Among individuals with valid response for physical activity: n = 5,263 and n = 2,144.
[d]Among individuals with valid response for depression: n = 5,013 and n = 2,013.
[e]Among individuals with valid response for instrumental activities of daily living: n = 5,262 and n = 2,143.

Because of the high number of invalid responses for education, economic status, chronic medical conditions, and occupation among persons lost to follow-up, it was difficult to compare these factors between analyzed participants and those lost to follow-up.
