# Supplementary material for: Longitudinal association between lifetime workforce participation and risk of self-reported cognitive decline in community-dwelling older adults
Source: PLoS One. 2020 Jun 8;15(6):e0234392. doi: 10.1371/journal.pone.0234392 (PMC7279604; doi:10.1371/journal.pone.0234392)
Supplement: S2 Table — (PDF) [file pone.0234392.s002.pdf]

S2 Table. Baseline characteristics of analyzed participants by gender

	Men (n = 2,422)		Women (n = 2,852)		*P*[a]
**Socio-demographic factors**					
Age at baseline					0.034
65–69 years	868	(35.8)	1,013	(35.5)	
70–74 years	823	(34.0)	897	(31.5)	
75–79 years	449	(18.5)	541	(19.0)	
≥80 years	282	(11.6)	401	(14.1)	
Educational attainment					0.002
≥12 years	1,889	(78.0)	2,111	(74.0)	
<12 years	519	(21.4)	712	(25.0)	
Missing	14	(0.6)	29	(1.0)	
Self-perceived economic status					0.253
Well off	954	(39.4)	1,081	(37.9)	
Poor	1,300	(53.7)	1,543	(54.1)	
Missing	168	(6.9)	228	(8.0)	
Number of chronic medical conditions[b] under medical treatment					<0.001
None	886	(36.6)	1,268	(44.5)	
One	984	(40.6)	1,087	(38.1)	
≥2	364	(15.0)	210	(7.4)	
Missing	188	(7.8)	287	(10.1)	
**Lifestyle habits**					
Smoking history					<0.001
Never-smokers	617	(25.5)	2,485	(87.1)	
Ex/current smokers	1,697	(70.1)	226	(7.9)	
Missing	108	(4.5)	141	(4.9)	
Physical activity					0.022
Active	914	(37.7)	976	(34.2)	
Inactive	1,504	(62.1)	1,869	(65.5)	
Missing	4	(0.2)	7	(0.2)	

S2 Table. Continued

	Men (n = 2,422)		Women (n = 2,852)		P^a
**Mental and physical health**					
Depression (score of the 5-item short form of the Geriatric Depression Scale)					0.001
No depression (scores of 0–1)	1,946	(80.3)	2,181	(76.5)	
Depression (scores of ≥2)	353	(14.6)	533	(18.7)	
Missing	123	(5.1)	138	(4.8)	
Instrumental activities of daily living (score of the 5-item subscale of the Tokyo Metropolitan Institute of Gerontology Index of Competence)					<0.001
Independent (a perfect score of 5)	2,105	(86.9)	2,774	(97.3)	
Poor (scores of ≤4)	308	(12.7)	75	(2.6)	
Missing	9	(0.4)	3	(0.1)	
**Lifetime workforce participation**					
Workforce participation at baseline					<0.001
Non-participation	1,688	(69.7)	2,416	(84.7)	
Participation	734	(30.3)	436	(15.3)	
The longest-held occupation					<0.001
Blue-collar[c]	517	(21.3)	589	(20.7)	
White-collar[d]	1,152	(47.6)	402	(14.1)	
Pink-collar[e]	636	(26.3)	1,408	(49.4)	
Other[f]	117	(4.8)	453	(15.9)	
Lifetime working years					<0.001
0–4 years	104	(4.3)	635	(22.3)	
5–14 years	27	(1.1)	682	(23.9)	
15–24 years	21	(0.9)	510	(17.9)	
≥25 years	2,270	(93.7)	1,025	(35.9)	

Data are given as n (%).
[a]Differences between men and women were analyzed using chi-squared test.
[b]Chronic medical conditions included hypertension, diabetes mellitus, heart disease, and cerebrovascular disease.
[c]Blue collar included manufacturing, transport, maintenance, construction, mining, security, agriculture, forestry, fishery, delivery, cleaning, and packing workers.
[d]White collar included administrators, managers, and professionals.
[e]Pink collar included clerical, sales, and services workers.
[f]Other included persons not classifiable by occupation and persons without work experience.
