# Supplementary material for: Longitudinal association between lifetime workforce participation and risk of self-reported cognitive decline in community-dwelling older adults
Source: PLoS One. 2020 Jun 8;15(6):e0234392. doi: 10.1371/journal.pone.0234392 (PMC7279604; doi:10.1371/journal.pone.0234392)
Supplement: S3 Table — (PDF) [file pone.0234392.s003.pdf]

S3 Table. Comparison between the findings obtained from multiple imputation data and those from complete data, based on a mutually adjusted model

	Multiple imputation data		Complete data	
	n	CIR (95% CI)	n	CIR (95% CI)
**Men (n = 2,422)**				
Workforce participation at baseline				
Non-participation	1,688	1.00	1,437	1.00
Participation	734	0.92 (0.73-1.16)	617	0.94 (0.73-1.21)
The longest-held occupation				
Blue-collar	517	1.00	428	1.00
White-collar	1,152	***0.72 (0.57-0.91)***	988	***0.68 (0.53-0.87)***
Pink-collar	636	0.82 (0.63-1.07)	543	0.82 (0.62-1.08)
Other	117	1.05 (0.72-1.53)	95	1.03 (0.69-1.55)
Lifetime working years				
Short: 0–24 years	152	1.00	121	1.00
Long: ≥25 years	2,270	0.83 (0.60-1.14)	1,933	0.85 (0.59-1.21)
**Women (n = 2,852)**				
Workforce participation at baseline				
Non-participation	2,416	1.00	1,954	1.00
Participation	436	0.89 (0.65-1.23)	352	0.89 (0.61-1.29)
The longest-held occupation				
Blue-collar	589	1.00	456	1.00
White-collar	402	1.02 (0.75-1.38)	341	1.01 (0.73-1.40)
Pink-collar	1,408	0.96 (0.76-1.22)	1,169	0.90 (0.69-1.18)
Other	453	1.21 (0.91-1.60)	340	1.07 (0.77-1.50)
Lifetime working years				
0–4 years	635	1.00	504	1.00
5–14 years	682	1.09 (0.84-1.42)	551	0.98 (0.72-1.33)
15–24 years	510	0.91 (0.67-1.22)	414	0.83 (0.59-1.15)
≥25 years	1,025	0.81 (0.62-1.06)	837	0.77 (0.57-1.03)
P for trend		0.029		0.034

Results in bold italic indicate $P < 0.05$.

CI, confidence interval; CIR, cumulative incidence ratio.

The model was adjusted for age, education, self-perceived economic status, chronic medical conditions, smoking history, physical activity, depression, instrumental activities of daily living, workforce participation at baseline, the longest-held occupation, and lifetime working years.
