# Supplementary material for: Longitudinal association between lifetime workforce participation and risk of self-reported cognitive decline in community-dwelling older adults
Source: PLoS One. 2020 Jun 8;15(6):e0234392. doi: 10.1371/journal.pone.0234392 (PMC7279604; doi:10.1371/journal.pone.0234392)
Supplement: S4 Table — (PDF) [file pone.0234392.s004.pdf]

S4 Table. Association between lifetime workforce participation and the Cognitive Performance Scale score by gender, based on the analysis of covariance (ANCOVA)

	Adjusted mean (standard error)					Estimated value of parameter	
	Mean (SE)				*P*-value[a]	β (95% CI)	*P*-value
**Men (n = 2,422)**							
Workforce participation at baseline							
Non-participation	0.46 (0.03)				0.236	Reference	
Participation	0.43 (0.04)					-0.031 (-0.081 to 0.020)	0.236
Occupation for the longest held job							
Blue-collar	0.47 (0.04)	*			0.002	Reference	
White-collar	0.38 (0.04)	*	*			-0.086 (-0.149 to -0.024)	0.007
Pink-collar	0.39 (0.04)			*		-0.073 (-0.142 to -0.005)	0.035
Other	0.56 (0.06)		*	*		0.091 (-0.028 to 0.210)	0.135
Lifetime working years							
Short: 0–24 years	0.44 (0.05)				0.766	Reference	
Long: ≥25 years	0.46 (0.03)					0.015 (-0.084 to 0.114)	0.766
**Women (n = 2,852)**							
Workforce participation at baseline							
Non-participation	0.52 (0.04)				0.670	Reference	
Participation	0.51 (0.05)					-0.014 (-0.077 to 0.049)	0.670
Occupation for the longest held job							
Blue-collar	0.49 (0.05)				0.690	Reference	
White-collar	0.52 (0.05)					0.033 (-0.044 to 0.110)	0.400
Pink-collar	0.51 (0.05)					0.023 (-0.037 to 0.082)	0.454
Other	0.54 (0.05)					0.047 (-0.035 to 0.129)	0.264
Lifetime working years							
0–4 years	0.56 (0.05)				0.111	Reference	
5–14 years	0.52 (0.05)					-0.038 (-0.109 to 0.033)	0.297
15–24 years	0.50 (0.05)					-0.059 (-0.136 to 0.017)	0.128
≥25 years	0.48 (0.05)					-0.082 (-0.151 to -0.014)	0.019
						P for trend = 0.015	

CI, confidence interval; SE, standard error.

The Cognitive Performance Scale score was adjusted for age, education, self-perceived economic status, chronic medical conditions, smoking history, physical activity, depression, instrumental activities of daily living, workforce participation at baseline, the longest-held occupation, and lifetime working years.

[a] This is by use of an analysis of covariance (ANCOVA) for differences in mean scores.

* $P<0.05$ with the Bonferroni correction method.
