# Supplementary material for: Longitudinal association between lifetime workforce participation and risk of self-reported cognitive decline in community-dwelling older adults
Source: PLoS One. 2020 Jun 8;15(6):e0234392. doi: 10.1371/journal.pone.0234392 (PMC7279604; doi:10.1371/journal.pone.0234392)
Supplement: S5 Table — (PDF) [file pone.0234392.s005.pdf]

S5 Table. Adjusted cumulative incidence ratio for 33-month cognitive decline based on stratified analyses by age, education, and physical activity among men (n = 2,422)

Stratified analyses by age in years

	65–74 years (n = 1,691)			≥75 years (n = 731)		
	n	CIR[a] (95% CI)	*P*	n	CIR[a] (95% CI)	*P*
Workforce participation at baseline						
Non-participation	1,079	1.00		609	1.00	
Participation	612	0.86 (0.64-1.16)	0.325	122	0.98 (0.69-1.39)	0.898
Occupation for the longest held job						
Blue-collar	327	1.00		190	1.00	
White-collar	821	0.64 (0.45-0.91)	0.014	331	0.82 (0.61-1.11)	0.193
Pink-collar	477	0.75 (0.51-1.11)	0.154	159	0.88 (0.62-1.24)	0.450
Other	66	1.03 (0.58-1.82)	0.921	51	1.11 (0.68-1.81)	0.676
Lifetime working years						
Short: 0–24 years	87	1.00		65	1.00	
Long: ≥25 years	1,604	0.68 (0.42-1.10)	0.115	666	0.98 (0.63-1.50)	0.912

Stratified analyses by years in education

	≥12 years (n[b] = 1,900)			<12 years (n[b] = 522)		
	n[b]	CIR[c] (95% CI)	*P*	n[b]	CIR[c] (95% CI)	*P*
Workforce participation at baseline						
Non-participation	1,305	1.00		383	1.00	
Participation	595	0.87 (0.66-1.16)	0.354	139	1.08 (0.72-1.62)	0.718
Occupation for the longest held job						
Blue-collar	261	1.00		256	1.00	
White-collar	1,005	0.71 (0.52-0.96)	0.027	147	0.77 (0.53-1.11)	0.163
Pink-collar	550	0.81 (0.59-1.11)	0.191	86	0.79 (0.47-1.32)	0.370
Other	84	0.97 (0.57-1.65)	0.908	33	1.17 (0.69-1.99)	0.550
Lifetime working years						
Short: 0–24 years	104	1.00		48	1.00	
Long: ≥25 years	1,796	0.92 (0.59-1.45)	0.730	474	0.78 (0.48-1.27)	0.317

Stratified analyses by physical activity

	Active (n^b = 915)			Inactive (n^b = 1,507)		
	n^b	CIR[c] (95% CI)	*P*	n^b	CIR[c] (95% CI)	*P*
Workforce participation at baseline						
Non-participation	642	1.00		1,046	1.00	
Participation	273	1.11 (0.74-1.67)	0.603	461	0.86 (0.64-1.14)	0.292
Occupation for the longest held job						
Blue-collar	161	1.00		355	1.00	
White-collar	475	0.57 (0.38-0.87)	0.008	678	0.77 (0.58-1.02)	0.068
Pink-collar	242	0.60 (0.37-0.99)	0.046	394	0.92 (0.68-1.25)	0.616
Other	37	0.41 (0.15-1.11)	0.079	80	1.29 (0.85-1.94)	0.226
Lifetime working years						
Short: 0–24 years	51	1.00		101	1.00	
Long: ≥25 years	864	1.10 (0.54-2.24)	0.797	1,406	0.77 (0.54-1.09)	0.143

CI, confidence interval; CIR, cumulative incidence ratio.

[a]Adjusted for all covariates and three items of lifetime workforce participation.

[b]The pooled number by multiple imputations.

[c]Adjusted for all covariates excluding the stratifying variables and three items of lifetime workforce participation.
