# Supplementary material for: Longitudinal association between lifetime workforce participation and risk of self-reported cognitive decline in community-dwelling older adults
Source: PLoS One. 2020 Jun 8;15(6):e0234392. doi: 10.1371/journal.pone.0234392 (PMC7279604; doi:10.1371/journal.pone.0234392)
Supplement: S6 Table — (PDF) [file pone.0234392.s006.pdf]

S6 Table. Adjusted cumulative incidence ratio for 33-month cognitive decline based on stratified analyses by age, education, and physical activity among women (n = 2,852)

Stratified by age in years

	65–74 years (n = 1,910)			≥75 years (n = 942)		
	n	CIR[a] (95% CI)	*P*	n	CIR[a] (95% CI)	*P*
Workforce participation at baseline						
Non-participation	1,555	1.00		861	1.00	
Participation	355	1.17 (0.77-1.77)	0.466	81	0.60 (0.33-1.07)	0.083
Occupation for the longest held job						
Blue-collar	393	1.00		196	1.00	
White-collar	259	0.94 (0.57-1.56)	0.813	143	1.07 (0.73-1.56)	0.735
Pink-collar	1,012	0.77 (0.53-1.13)	0.188	396	1.15 (0.86-1.54)	0.345
Other	246	1.10 (0.68-1.79)	0.699	207	1.31 (0.93-1.85)	0.119
Lifetime working years						
0–4 years	382	1.00		253	1.00	
5–14 years	483	0.87 (0.56-1.33)	0.511	199	1.27 (0.92-1.76)	0.147
15–24 years	344	0.74 (0.46-1.19)	0.215	166	1.02 (0.70-1.49)	0.908
≥25 years	701	0.57 (0.37-0.88)	0.011	324	0.99 (0.71-1.38)	0.953
		P for trend = 0.005			*P* for trend = 0.507	

Stratified by years of education

	≥12 years (n[b] = 2,130)			<12 years (n[b] = 722)		
	n[b]	CIR[c] (95% CI)	*P*	n[b]	CIR[c] (95% CI)	*P*
Workforce participation at baseline						
Non-participation	1,793	1.00		623	1.00	
Participation	337	0.94 (0.63-1.41)	0.770	99	0.83 (0.48-1.41)	0.484
Occupation for the longest held job						
Blue-collar	302	1.00		287	1.00	
White-collar	328	0.99 (0.65-1.52)	0.978	74	1.10 (0.70-1.73)	0.681
Pink-collar	1,170	0.92 (0.65-1.31)	0.663	238	1.07 (0.79-1.47)	0.657
Other	330	1.65 (1.08-2.52)	0.020	123	0.83 (0.55-1.25)	0.369
Lifetime working years						
0–4 years	504	1.00		131	1.00	
5–14 years	531	1.59 (1.13-2.26)	0.009	151	0.66 (0.43-1.00)	0.048
15–24 years	373	1.29 (0.86-1.93)	0.222	137	0.58 (0.38-0.89)	0.012
≥25 years	722	1.11 (0.76-1.62)	0.586	303	0.54 (0.37-0.78)	0.001
		P for trend = 0.692			*P* for trend = 0.003	

Stratified by physical activity

	Active (n^b = 978)			Inactive (n^b= 1,874)		
	n^b	CIR[c] (95% CI)	*P*	n^b	CIR[c] (95% CI)	*P*
Workforce participation at baseline						
Non-participation	832	1.00		1,584	1.00	
Participation	146	1.11 (0.67-1.84)	0.693	290	0.78 (0.51-1.19)	0.251
Occupation for the longest held job						
Blue-collar	177	1.00		412	1.00	
White-collar	153	1.07 (0.63-1.83)	0.807	249	0.99 (0.68-1.44)	0.950
Pink-collar	522	0.88 (0.57-1.35)	0.557	886	1.02 (0.78-1.35)	0.868
Other	126	1.45 (0.85-2.48)	0.170	327	1.17 (0.83-1.63)	0.374
Lifetime working years						
0–4 years	216	1.00		419	1.00	
5–14 years	243	1.56 (0.96-2.53)	0.075	439	0.95 (0.69-1.30)	0.745
15–24 years	186	0.92 (0.51-1.66)	0.782	324	0.93 (0.65-1.31)	0.666
≥25 years	333	1.11 (0.68-1.82)	0.671	692	0.72 (0.52-0.98)	0.039
		P for trend = 0.646			*P* for trend = 0.025	

CI, confidence interval; CIR, cumulative incidence ratio.

[a]Adjusted for all covariates and three items of lifetime workforce participation.

[b]The pooled number by multiple imputations.

[c]Adjusted for all covariates excluding the stratifying variables and three items of lifetime workforce participation.
