# Supplementary material for: Longitudinal association between lifetime workforce participation and risk of self-reported cognitive decline in community-dwelling older adults
Source: PLoS One. 2020 Jun 8;15(6):e0234392. doi: 10.1371/journal.pone.0234392 (PMC7279604; doi:10.1371/journal.pone.0234392)
Supplement: S1 Text — (PDF) [file pone.0234392.s007.pdf]

S1 Text. Detailed explanation of basic activities of daily living, the longest-held occupation, and multiple imputations

Assessment of basic activities of daily living

Basic activities of daily living were evaluated using the Barthel index (score range 0–100) [1]; higher scores indicate better basic activities of daily living. A score of ≥90 identified respondents with independent basic activities of daily living [2]. Therefore, persons with a score of <90 at baseline were excluded from the present study.

Assessment of the longest-held occupation

All subjects were asked about the presence or absence of lifetime work experience. Then, for those with work experience, we inquired about what their longest held occupation was. Occupations were classified into the twelve types based on the Japan Standard Occupational Classification (the Major Groups) [3]. Based on this classification, occupations were restructured into white-collar (i.e., administrators, managers, and professionals), pink-collar (i.e., clerical, sales, and services workers), blue-collar (i.e., workers in manufacturing, transport, maintenance, construction, mining, security, agriculture, forestry, fishery, delivery, cleaning, and packing), and other (i.e., persons not classifiable by occupation and persons without work experience) [4].

Multiple imputations

Using multiple imputations with multivariate imputation by chained equations [5], we

created five sets of imputation data and performed analyses on the complete pooled data set. Gender, age, education, self-perceived economic status, chronic medical conditions, smoking history, physical activity, depression, and instrumental activities of daily living, the independent variables (i.e., workforce participation at baseline, the longest-held job, and lifetime working years), and outcome (i.e., cognitive decline) were entered into the imputation procedure. For this study, there were no missing data on gender and age. The following were imputed as ordinal variables: educational attainment (0.8% missing), self-perceived economic status (7.5% missing), chronic medical conditions (9.0% missing), smoking history (4.7% missing), physical activity (0.2% missing), depression (4.9% missing), and instrumental activities of daily living (0.2% missing). Multiple imputations were conducted using the IBM SPSS Missing Values Option.

We compared the individuals with and without missing covariate data. Compared to individuals with complete covariates (n = 4,360), those with missing covariate values (n = 914) were older and tended to be female. The presence or absence of missing covariate data was not associated with cognitive decline. For individuals with complete date and those with missing data respectively, the proportion of people aged 75 or older was 30.3% and 38.6% (P <0.001 by chi-squared test), the proportion of women was 52.9% and 59.7% (P <0.001 by chi-squared test), and the proportion of people with cognitive decline was 14.6% and 17.0% (P = 0.075 by chi-squared test).

REFERENCES

1. Mahoney FI, Barthel DW. Functional evaluation: The Barthel Index. Md State Med J

1965;14:61–65.

2. Granger CV, Albrecht GL, Hamilton BB. Outcome of comprehensive medical rehabilitation: Measures of PULSES profile and Barthel Index. Arch Phys Med Rehabil. 1979;60:145–154.
3. Ministry of Internal Affairs and Communications Japan. Japan Standard Occupational Classification. http://www.soumu.go.jp/english/dgpp_ss/seido/shokgyou/index-co.htm (accessed January 1, 2019).
4. Welsh J, Strazdins L, Charlesworth S, Kulik CT, Butterworth P. Health or harm? A cohort study of the importance of job quality in extended workforce participation by older adults. BMC Public Health. 2016;16:885.
5. White IR, Royston P, Wood AM. Multiple imputation using chained equations: Issues and guidance for practice. Stat Med 2011;30:377–399.
