# Supplementary material for: Longitudinal association between lifetime workforce participation and risk of self-reported cognitive decline in community-dwelling older adults
Source: PLoS One. 2020 Jun 8;15(6):e0234392. doi: 10.1371/journal.pone.0234392 (PMC7279604; doi:10.1371/journal.pone.0234392)
Supplement: S1 File — (PDF) [file pone.0234392.s008.pdf]

ID	Age2014	Gender	CD	Marital status	Education	Economic status	BMI	Chronic diseases	Drink	Smoking	Sports	Volunteer	Depression	IADL	WP at baseline	Occupation	Working years
1	75	2	0	1	0	0	1	0	0	0	0	0	0	0	0	4	1
2	70	1	0	0	0	1	1	1	1	1	0	0	0	0	0	1	4
3	67	2	0	0	0	1	1	2	0	1	0	0	0	0	0	3	4
4	76	2	0	0	0	0	1	2	0	1	1	0	0	0	0	3	4
9	70	2	0	0	0	1	3	0	0	0	1	1	0	0	0	2	2
11	68	2	0	0	0	0	1	1	0	0	1	0	0	0	0	2	3
12	79	1	1	1	0	0	1	0	1	1	0	0	0	1	0	3	4
14	83	2	0	0	0	0	1	0	0	0	1	0	0	0	0	2	4
15	67	1	0	0	0	0	3	1	0	1	1	0	0	0	0	1	4
16	67	2	0	0	0	0	1	1	0	0	0	1	0	0	0	3	2
18	79	2	1	1	0	0	1	1	0	0	1	1	0	0	0	1	2
19	85	2	1	1	0	0	1	1	0	0	0	1	0	1	0	4	1
20	79	2	0	0	0	0	3	1	0	0	0	0	1	0	0	3	1
21	70	1	0	0	0	1	1	1	0	1	1	0	0	0	1	2	4
23	73	1	0	0	0	0	3	1	0	1	0	0	0	0	0	1	4
28	68	2	0	0	0	1	1	2	0	0	0	0	1	0	0	4	1
30	77	2	0	0	0	0	1	2	0	0	0	0	0	0	0	1	4
33	85	1	1	0	1	0	1	1	0	1	0	1	0	1	0	1	4
34	81	2	1	0	0	0	1	0	0	0	1	0	1	0	0	2	4
37	68	1	1	0	0	0	1	1	1	1	0	0	0	0	1	3	4
39	67	2	0	0	0	0	1	1	0	0	0	0	1	0	0	2	2
43	70	1	0	0	0	0	1	0	0	1	1	1	0	1	0	1	4
44	69	1	1	0	0	1	2	0	0	1	0	0	0	0	1	2	4
45	66	2	0	0	0	1	1	1	0	0	0	0	0	0	0	2	4
47	72	2	0	0	0	0	1	0	0	0	0	0	1	0	0	2	4
50	67	2	0	0	0	1	1	1	0	0	0	0	0	0	0	2	1
54	67	1	0	0	0	0	1	1	0	1	0	1	0	0	1	1	4
57	66	1	0	0	0	1	1	1	1	0	1	0	0	#NULL!	1	2	4
58	75	1	0	0	0	1	2	0	0	0	0	0	0	0	0	4	1
59	68	2	0	0	0	1	1	0	0	0	0	1	0	0	0	4	1
60	78	1	0	0	0	0	1	1	1	1	0	0	0	0	0	1	4
62	68	1	0	0	0	0	1	0	1	1	1	0	0	0	0	2	4
63	66	2	0	0	0	0	3	1	0	0	0	0	0	0	0	2	1
69	80	1	1	0	0	0	1	0	1	1	0	0	1	0	0	1	4
70	76	2	0	0	0	0	1	0	0	0	1	0	0	0	0	2	4
71	77	2	0	1	0	0	1	1	0	0	1	0	0	0	0	1	4

72	65	2	0	0	0	0	1	0	0	0	1	0	0	0	1	2	4
76	77	2	0	0	0	0	2	1	0	0	1	0	1	0	0	4	1
78	66	2	0	0	1	1	1	1	1	0	1	0	1	0	0	3	1
79	66	1	0	0	0	0	2	0	1	1	1	0	1	0	1	1	4
80	68	1	0	0	#NULL!	1	1	2	1	1	0	0	0	0	0	2	4
81	66	2	0	0	0	1	1	1	0	0	1	0	0	0	0	2	4
84	78	2	0	0	0	1	#NULL!	1	0	0	1	0	1	0	0	4	1
85	89	2	0	1	0	1	1	0	0	0	0	0	1	1	0	2	2
87	88	2	1	0	0	#NULL!	1	1	0	0	0	1	0	0	0	2	4
90	71	1	0	0	0	0	2	1	1	1	1	0	0	0	0	1	4
91	68	1	0	0	0	0	3	1	1	1	0	1	0	1	1	1	4
93	65	2	0	1	0	0	1	0	0	0	0	0	0	0	1	2	4
94	75	1	0	0	0	0	1	2	1	1	1	0	0	0	0	1	4
95	73	2	0	0	1	1	1	0	0	0	0	0	0	0	0	1	3
98	81	1	1	0	0	0	1	0	0	0	0	0	1	1	0	1	4
99	77	2	1	0	0	0	2	1	0	0	0	0	1	0	0	2	1
100	72	1	0	0	0	0	1	1	1	0	1	1	0	0	0	1	4
101	70	2	0	0	0	0	1	0	0	0	1	1	0	0	0	1	3
102	70	1	0	#NULL!	0	1	1	#NULL!	#NULL!	#NULL!	0	0	#NULL!	0	1	1	4
103	67	2	0	0	0	1	3	0	0	0	1	1	0	0	0	2	2
104	71	1	0	0	0	0	3	1	1	1	0	0	0	0	0	2	4
106	84	1	1	#NULL!	0	0	#NULL!	0	0	0	0	1	0	0	0	1	4
107	73	2	0	1	0	1	2	#NULL!	#NULL!	#NULL!	1	0	#NULL!	0	0	2	4
108	69	2	0	1	1	0	2	0	0	0	0	1	0	0	1	1	4
109	70	2	1	1	1	1	3	1	0	0	0	0	1	0	0	1	4
110	71	2	0	1	0	1	3	1	#NULL!	1	0	1	1	0	0	3	1
112	67	1	0	0	0	0	1	0	1	1	1	1	0	0	1	2	4
113	66	2	0	0	0	0	1	0	0	0	1	1	0	0	0	1	4
114	70	1	0	0	0	1	1	2	1	1	1	0	1	0	1	2	4
115	66	1	0	0	0	0	3	0	0	1	1	0	0	0	1	1	4
116	65	2	0	1	0	0	1	1	0	0	1	0	0	0	0	1	2
118	72	1	0	0	0	1	1	1	0	0	1	0	0	0	0	1	1
119	72	2	0	0	0	1	1	1	0	0	1	0	1	0	0	1	1
122	68	2	0	0	0	0	1	0	1	0	1	1	0	0	1	2	3
129	65	1	0	0	0	1	1	0	1	1	1	0	0	0	1	1	4
130	72	1	0	0	0	0	1	2	1	1	1	0	0	0	0	1	4
131	71	2	0	0	0	0	1	0	0	0	0	0	0	0	0	2	2
136	74	2	0	1	0	0	1	0	0	0	1	1	0	0	0	1	4
137	67	1	0	0	0	0	3	1	1	1	1	0	0	0	1	3	4

138	66	2	0	0	0	0	1	1	0	0	0	0	0	0	0	2	4
139	68	1	0	0	1	0	1	1	0	0	0	0	0	0	1	1	4
140	70	2	0	1	1	1	3	0	0	0	1	0	0	0	0	2	4
141	77	1	0	0	0	#NULL!	1	1	1	1	0	0	0	0	0	1	4
142	72	2	0	0	0	1	1	0	0	0	1	1	0	0	0	2	4
143	82	1	0	0	1	1	1	1	1	1	0	0	1	0	0	1	4
145	82	2	0	0	0	#NULL!	3	#NULL!	#NULL!	#NULL!	1	0	#NULL!	0	0	2	2
151	77	1	0	0	1	1	1	0	0	1	0	0	0	0	0	3	4
152	77	2	1	0	1	1	3	1	0	0	0	0	1	1	0	3	2
153	65	1	0	0	0	1	1	#NULL!	1	1	1	0	0	0	0	1	4
154	86	2	0	1	0	0	1	2	0	0	0	0	0	1	0	4	1
156	78	2	1	1	0	1	1	1	0	0	1	0	0	0	0	3	2
159	77	2	0	1	0	1	1	0	0	0	0	0	0	0	0	1	3
167	65	2	1	0	0	1	1	1	0	0	0	0	0	0	0	2	3
168	77	1	0	0	0	0	1	2	0	1	0	0	0	0	0	1	4
169	76	2	0	0	0	0	1	0	0	0	0	0	0	0	0	2	2
172	65	1	0	0	0	1	1	#NULL!	#NULL!	#NULL!	0	0	#NULL!	0	0	2	4
174	78	1	0	0	0	0	1	0	0	1	1	1	0	0	0	1	4
175	74	2	0	0	0	0	1	1	0	0	0	0	0	0	0	2	2
178	74	1	0	0	0	1	3	1	1	1	1	1	0	0	0	1	4
179	65	2	0	0	0	1	3	1	0	0	0	0	0	0	0	2	1
180	75	1	0	0	0	0	3	2	0	1	0	0	0	0	1	1	4
181	73	2	0	0	0	0	1	1	0	0	1	1	0	0	0	2	4
183	71	2	0	0	0	1	1	1	0	0	1	0	0	0	0	2	1
185	76	2	0	0	0	0	1	0	0	0	1	1	0	0	0	3	2
186	66	2	0	0	0	0	1	1	1	0	1	0	0	0	0	2	4
187	74	2	0	1	0	1	2	0	0	0	0	0	0	0	1	2	4
190	75	1	0	0	0	0	1	2	0	1	1	0	0	0	0	1	4
193	66	2	0	0	0	0	1	1	0	0	1	0	0	0	0	1	2
194	71	1	0	0	0	#NULL!	1	0	0	1	1	0	0	0	0	1	4
196	65	2	0	0	0	1	3	0	0	0	0	0	0	0	0	1	4
199	66	2	0	0	0	1	1	1	0	0	1	0	0	0	0	2	4
201	76	2	0	0	0	#NULL!	1	1	0	0	0	0	0	0	0	2	2
204	73	1	0	0	1	1	1	2	0	1	0	1	1	0	0	4	4
207	67	2	0	1	0	0	1	0	0	0	1	1	0	0	0	2	1
210	69	2	0	0	0	0	2	0	0	0	1	1	0	0	0	2	1
211	77	1	0	0	0	0	1	0	0	0	0	0	0	0	0	1	4
213	74	1	1	0	1	1	3	0	1	1	0	0	0	0	0	3	4
215	79	1	0	0	0	1	1	1	0	0	1	0	0	1	0	1	4

216	72	2	0	0	0	1	1	0	0	0	0	1	0	0	0	2	3
217	75	1	0	1	0	0	3	1	0	0	1	0	0	0	0	2	4
220	73	2	0	0	0	0	1	2	0	0	1	1	0	0	0	2	3
221	78	1	1	0	0	1	1	0	1	0	1	1	0	0	0	1	4
222	72	2	0	0	0	0	1	1	1	#NULL!	0	1	0	0	0	2	1
223	80	1	0	0	0	0	1	2	1	1	0	1	0	0	0	1	4
224	79	2	0	0	0	0	3	1	0	0	0	0	0	0	1	1	3
226	68	2	0	0	0	0	3	0	0	0	1	0	1	0	0	1	2
227	76	2	0	0	0	1	1	1	0	0	0	0	0	0	0	1	4
229	73	1	0	0	0	0	1	0	1	1	1	0	0	0	0	1	4
233	67	2	0	0	0	0	2	#NULL!	0	0	0	1	0	0	0	2	1
236	82	2	0	1	0	1	1	1	0	0	0	0	1	0	0	2	4
237	86	1	0	0	0	#NULL!	2	2	1	1	0	0	0	0	0	3	4
238	83	2	1	0	0	#NULL!	2	0	0	0	0	0	0	0	0	3	3
240	81	2	0	1	0	1	1	0	0	0	0	0	0	#NULL!	0	2	3
241	71	1	0	0	0	1	1	0	0	1	0	0	0	0	0	3	4
242	67	2	0	0	0	1	1	2	0	1	0	0	0	0	1	3	4
244	65	2	0	1	0	1	1	0	0	1	0	0	1	0	0	2	2
246	66	1	0	0	0	0	1	1	1	0	1	0	0	0	1	1	4
250	66	2	0	0	0	1	3	0	0	0	0	0	0	0	0	2	4
251	87	2	0	1	1	1	1	0	0	0	0	0	0	1	0	1	4
256	68	2	0	1	1	0	3	1	0	0	0	0	0	0	0	2	4
259	69	2	0	0	0	1	1	0	0	0	1	0	0	0	0	2	3
260	72	1	0	0	0	1	1	0	0	1	1	0	0	0	0	1	4
261	69	2	0	0	0	1	1	0	0	0	1	0	0	0	0	2	2
263	65	1	0	0	0	#NULL!	1	0	0	1	0	0	0	0	1	1	4
264	65	2	0	0	0	#NULL!	3	0	0	0	0	0	0	0	0	2	4
266	65	1	0	0	0	0	1	1	1	1	0	0	0	1	1	1	4
272	78	1	0	0	0	0	3	2	0	1	1	0	0	0	1	3	4
274	71	2	0	1	0	1	1	1	0	0	0	0	1	0	0	2	4
275	70	1	0	0	1	1	1	1	0	1	0	0	0	0	1	2	2
277	65	1	0	0	0	1	1	1	1	1	1	0	0	1	0	1	4
279	65	1	0	0	0	1	1	0	0	1	1	0	0	0	1	1	4
285	73	1	0	0	0	1	1	1	1	1	1	1	0	0	1	2	4
286	69	2	0	0	0	1	1	0	0	0	0	0	0	0	0	2	3
289	66	1	0	0	0	0	1	1	0	1	1	0	0	0	1	1	4
293	74	1	0	0	0	0	1	2	0	1	0	0	0	0	0	4	1
294	69	2	0	0	0	0	1	0	0	0	1	0	0	0	0	2	2
296	77	1	0	0	0	1	1	2	1	1	0	0	0	0	0	2	4

297	76	2	0	0	0	1	1	1	1	1	0	0	0	0	0	4	4
298	81	2	0	1	0	1	1	0	0	0	0	0	0	0	0	4	1
300	89	2	1	0	0	0	#NULL!	#NULL!	#NULL!	#NULL!	0	0	#NULL!	0	0	4	1
306	66	2	0	0	0	0	2	0	0	0	1	0	0	0	1	2	3
307	66	1	0	0	0	1	3	1	1	0	0	1	0	0	0	1	4
311	73	1	0	1	0	1	1	2	0	0	0	0	1	0	0	1	4
312	82	1	1	0	#NULL!	1	1	1	0	0	0	0	0	0	0	1	4
314	65	1	0	0	0	0	3	1	1	1	1	1	0	0	1	1	4
315	69	1	0	0	0	0	1	1	1	1	0	0	0	0	0	1	4
317	82	2	1	1	1	0	2	0	0	0	1	0	0	1	0	1	3
319	70	2	1	0	0	0	3	0	0	0	1	1	1	0	0	2	3
320	66	2	0	1	0	0	1	1	1	0	0	0	0	0	0	2	3
321	71	1	1	0	0	0	1	0	0	0	0	0	0	0	0	1	4
326	65	1	0	0	0	0	3	1	0	1	0	0	0	0	0	3	4
328	66	1	1	0	0	1	1	0	0	0	1	1	0	1	0	1	4
333	67	1	0	0	0	#NULL!	3	1	1	1	1	0	0	0	0	1	4
334	76	2	0	0	0	0	3	1	0	0	0	0	0	0	0	2	4
336	66	2	0	0	0	0	1	0	0	0	0	0	1	0	0	2	1
337	66	1	0	0	0	1	3	1	0	1	0	0	1	0	0	1	1
338	66	2	0	1	0	0	1	0	0	0	1	0	1	0	0	2	2
342	72	1	0	0	0	1	1	1	0	1	0	0	0	0	0	2	4
343	65	2	0	0	0	1	1	1	1	0	1	1	0	0	0	2	3
345	65	1	0	0	0	0	1	0	1	1	0	0	0	0	1	1	4
346	69	1	0	0	0	0	1	0	0	1	0	0	0	1	0	1	4
347	67	2	0	0	0	1	1	0	0	0	0	0	0	0	0	2	2
355	78	1	0	0	0	1	3	1	#NULL!	1	0	0	#NULL!	0	0	4	4
359	79	1	0	0	0	0	1	0	0	0	1	1	0	0	0	2	4
360	72	2	0	0	1	1	2	0	0	0	0	0	0	0	0	2	4
362	76	2	0	0	0	1	3	#NULL!	0	0	0	0	1	0	0	3	4
364	68	1	0	0	0	1	1	0	0	1	1	0	0	0	0	2	4
365	70	1	0	0	0	0	1	1	1	1	0	0	0	0	0	1	4
366	65	2	0	0	0	0	2	1	1	0	0	0	0	0	0	2	3
367	84	2	0	1	0	0	1	1	0	0	0	0	1	0	0	1	4
369	73	1	0	0	0	1	1	1	1	0	0	1	0	0	0	3	4
370	70	2	0	0	0	1	1	1	0	0	0	0	1	0	0	2	4
371	72	1	0	0	0	0	1	0	1	1	1	1	0	0	0	2	4
372	66	2	0	0	0	1	2	1	1	0	1	1	0	0	0	2	3
373	75	2	0	1	0	1	1	1	0	0	0	0	0	0	0	2	3
374	83	1	0	0	0	0	1	0	0	1	1	1	0	0	1	2	4

376	68	1	0	0	0	0	1	0	0	0	0	0	0	0	1	1	4
377	74	1	0	0	0	0	1	0	0	1	0	0	0	0	1	1	4
378	72	2	0	0	0	0	1	1	0	0	1	1	0	0	1	1	4
379	72	2	1	0	0	1	3	1	0	0	0	0	0	0	0	3	3
381	74	2	0	0	1	0	1	1	0	0	0	0	0	0	0	3	2
382	73	1	1	0	0	0	1	1	0	1	0	0	0	0	0	3	4
385	77	2	0	0	1	1	1	1	0	0	1	0	1	0	0	3	4
387	72	1	0	0	0	0	3	0	1	1	1	0	0	0	1	1	4
388	68	1	0	0	0	0	3	1	1	1	0	1	0	0	0	1	4
392	69	1	1	0	0	1	1	#NULL!	#NULL!	#NULL!	1	1	#NULL!	0	0	1	4
393	72	1	0	0	0	1	1	2	0	0	0	0	1	0	0	2	4
403	73	2	1	0	0	1	1	0	0	0	0	0	1	0	0	1	1
404	74	2	0	0	0	0	1	1	0	0	1	1	0	0	0	2	4
405	72	1	1	0	1	0	1	1	1	1	0	0	1	1	0	1	4
408	82	1	0	0	1	0	1	1	1	1	1	1	0	0	0	4	1
411	70	1	0	0	0	1	3	1	1	1	0	0	0	0	0	1	4
413	73	1	0	0	0	0	3	1	1	1	1	1	0	0	1	1	4
414	71	2	0	0	0	0	2	0	1	0	1	1	0	0	0	1	3
420	68	1	0	0	0	1	2	2	1	1	1	0	0	0	0	1	4
422	74	1	0	0	0	1	3	1	0	1	0	0	0	1	0	1	4
423	72	2	0	0	0	0	3	1	0	0	0	0	0	0	0	2	1
425	71	1	0	0	0	1	3	0	0	1	1	1	0	0	0	2	4
426	66	2	0	0	0	1	1	1	0	0	1	1	0	0	0	1	4
428	71	1	0	0	0	#NULL!	1	2	0	1	1	0	0	0	1	2	4
429	73	1	0	0	0	1	1	0	0	1	1	0	0	0	1	1	4
433	66	2	0	0	1	0	1	0	0	0	0	0	0	0	0	2	3
435	72	1	1	0	0	#NULL!	1	1	#NULL!	1	0	0	0	1	0	2	4
436	70	2	0	0	0	0	1	2	0	0	0	1	1	0	0	4	1
439	69	2	0	1	0	0	1	0	0	1	1	1	0	0	1	2	3
442	71	1	0	0	0	1	1	0	0	1	0	0	0	0	0	1	4
443	71	2	0	1	0	1	1	1	0	0	0	1	1	0	0	2	3
444	72	2	0	0	0	0	1	0	0	0	0	0	0	0	0	2	1
445	71	1	0	0	0	0	1	1	0	0	0	0	0	0	0	2	4
446	88	1	0	1	0	0	1	1	1	1	0	1	0	0	0	1	4
450	67	1	0	0	0	1	1	0	1	1	1	0	0	0	0	2	4
451	65	2	0	0	0	#NULL!	1	1	1	0	1	0	0	0	0	2	4
453	65	1	0	0	0	0	1	0	0	1	0	0	0	0	0	1	4
455	71	2	0	0	0	1	1	0	0	0	0	0	0	0	0	2	2
459	70	2	1	0	0	1	1	#NULL!	#NULL!	#NULL!	1	0	#NULL!	0	0	2	2

460	70	1	0	0	0	1	1	0	0	0	1	1	0	0	0	1	4
466	65	2	0	0	0	1	3	1	0	0	0	0	0	0	0	2	3
468	67	1	0	1	0	1	3	1	1	1	1	0	0	0	1	1	4
472	77	1	1	0	1	1	3	2	1	1	1	1	0	0	0	3	4
473	76	2	1	0	1	1	3	1	0	0	0	0	0	0	0	2	1
474	86	2	0	1	1	0	2	0	0	0	0	0	0	0	0	2	4
476	74	1	0	0	0	1	1	2	0	1	0	0	0	0	0	1	4
478	72	1	0	0	0	1	1	1	1	1	1	0	0	0	0	1	4
479	66	2	0	0	0	0	1	0	0	0	1	1	0	0	0	2	3
480	81	2	1	1	0	0	1	0	0	0	1	1	0	0	0	1	4
481	66	1	0	0	0	0	3	1	0	1	0	0	0	0	1	2	4
483	74	1	0	0	0	0	1	1	1	1	1	0	0	0	0	1	4
485	73	1	1	0	1	1	1	1	1	1	0	0	0	0	0	3	4
486	69	2	0	0	0	0	3	1	0	0	0	1	0	0	0	3	4
487	66	1	0	0	0	0	3	0	0	1	0	0	0	0	1	1	4
488	76	1	0	0	0	0	1	1	0	0	1	0	0	0	1	1	4
489	72	2	0	0	0	#NULL!	1	2	0	0	1	0	0	0	0	4	1
490	72	1	1	0	0	0	1	0	0	0	0	0	0	1	0	1	4
491	90	2	0	1	1	0	1	0	0	0	0	0	0	0	0	4	2
497	70	1	0	0	0	0	3	1	1	1	1	1	0	0	0	1	4
498	68	2	0	0	0	0	1	0	0	0	1	1	0	0	0	2	4
500	78	2	1	0	1	1	2	0	0	0	1	0	1	0	0	2	4
501	69	1	0	0	0	1	1	0	1	1	0	1	0	0	0	1	4
503	67	2	0	0	1	0	1	0	0	0	1	1	0	0	0	3	2
505	67	2	0	1	0	0	1	1	0	0	0	0	0	0	0	2	2
506	72	1	0	0	0	1	1	2	0	1	1	1	0	0	0	2	4
507	67	2	0	0	0	1	3	0	0	0	0	0	1	0	0	2	3
511	66	1	0	1	0	0	1	1	1	1	1	0	0	0	1	2	4
514	71	2	0	1	0	1	3	1	0	0	0	1	1	0	0	3	3
516	67	1	0	1	0	1	1	0	1	1	1	0	1	0	0	1	4
517	66	1	0	0	0	0	1	1	0	1	0	0	0	0	1	1	4
518	65	2	0	0	0	0	1	0	0	0	1	0	0	0	1	1	4
519	73	1	0	0	0	#NULL!	1	2	0	1	0	0	0	0	0	4	1
521	77	2	0	1	#NULL!	1	1	#NULL!	0	0	1	1	0	0	0	4	1
522	69	1	0	0	0	0	1	2	1	0	1	1	0	0	1	2	4
526	68	2	0	1	0	1	1	1	0	1	1	1	0	0	0	1	4
527	70	1	0	0	1	1	3	2	0	1	0	0	0	0	0	1	4
534	76	2	0	1	0	1	3	1	0	0	0	1	0	0	0	2	1
535	83	2	0	1	0	1	2	0	0	0	0	0	1	0	0	3	1

536	66	1	0	0	0	1	1	0	1	0	1	1	0	0	0	2	4
537	66	2	0	1	0	0	1	1	0	0	0	0	0	0	1	2	3
539	77	2	0	0	0	1	1	1	0	0	1	0	0	0	0	2	2
540	85	1	1	0	0	0	1	1	0	1	0	0	0	1	0	1	4
541	78	2	1	0	0	0	1	2	0	0	0	0	1	1	0	2	2
544	81	1	0	#NULL!	1	#NULL!	1	#NULL!	#NULL!	#NULL!	0	0	#NULL!	0	0	3	4
545	67	1	0	0	0	1	3	#NULL!	0	1	1	0	0	0	1	1	4
546	72	2	0	0	0	1	1	0	0	0	1	0	0	0	0	4	4
547	69	1	0	0	0	1	1	0	0	1	0	0	1	0	1	1	4
549	74	1	0	0	0	1	1	1	1	0	0	1	1	0	0	3	4
550	66	2	0	0	0	1	1	1	0	0	0	1	0	0	0	3	4
551	65	2	0	1	0	#NULL!	2	0	0	0	0	0	1	0	0	3	3
557	68	1	1	0	0	1	1	2	0	1	1	0	1	0	0	3	4
560	65	1	0	0	0	0	3	0	1	1	1	0	0	0	0	1	4
563	68	1	0	0	0	0	1	1	0	1	1	0	0	0	0	2	4
564	75	2	1	1	0	0	3	1	0	0	1	0	#NULL!	0	0	2	2
565	81	1	0	0	0	#NULL!	1	0	1	0	0	1	0	0	0	1	4
572	66	2	0	0	0	1	1	1	0	0	0	0	0	0	0	2	4
574	65	2	0	0	0	0	2	0	0	0	1	0	0	0	0	1	4
575	80	2	0	1	0	1	1	1	0	0	0	0	0	0	0	2	3
579	79	2	1	0	0	1	3	1	0	0	0	0	0	0	0	4	1
580	72	1	0	0	0	1	3	#NULL!	#NULL!	#NULL!	0	0	#NULL!	0	0	1	4
581	68	2	0	0	0	1	1	1	0	0	0	0	0	0	0	4	1
582	83	2	0	1	0	#NULL!	1	#NULL!	#NULL!	#NULL!	0	0	#NULL!	0	0	4	1
584	80	2	0	0	0	0	2	2	0	0	0	0	0	0	0	4	1
585	86	2	1	0	0	1	1	0	0	0	0	0	1	0	0	4	1
587	67	2	0	0	0	0	2	1	0	0	1	0	1	0	0	2	4
588	66	1	0	0	0	1	1	1	1	1	1	0	0	0	1	1	4
590	67	2	0	0	0	0	1	0	0	0	0	1	0	0	1	2	4
593	71	2	0	0	0	0	1	0	0	0	0	0	1	0	0	2	1
594	70	1	0	0	0	#NULL!	1	#NULL!	1	1	1	1	0	0	0	1	4
595	66	1	0	0	0	0	1	0	1	0	0	0	0	0	1	1	4
597	79	1	0	0	0	0	1	0	0	1	1	1	0	0	0	1	4
600	69	1	0	0	0	0	1	1	1	1	0	1	0	0	1	1	4
601	69	2	0	0	0	0	1	0	0	0	1	1	0	0	0	2	3
603	75	2	0	0	0	1	1	0	0	0	0	0	1	0	0	1	4
606	75	1	0	0	0	0	1	0	0	0	1	1	0	1	0	2	4
607	69	2	0	0	0	0	1	0	0	0	1	0	0	0	0	3	4
610	72	1	0	0	0	1	1	1	0	0	1	1	1	0	1	2	4

613	76	2	0	0	0	0	1	1	0	0	1	1	1	0	0	2	1
616	84	2	0	1	0	0	1	1	0	0	0	0	0	1	0	2	4
617	81	2	0	1	0	1	1	1	0	0	0	0	0	0	0	2	4
618	66	1	0	0	0	0	1	0	1	0	1	0	0	0	1	1	4
620	70	1	0	0	1	0	1	0	0	1	0	0	#NULL!	0	1	1	4
622	69	1	1	0	0	1	3	2	0	1	1	0	0	0	0	2	4
623	68	1	0	0	0	0	1	1	0	1	0	0	0	0	0	1	4
624	80	2	0	1	0	0	1	#NULL!	0	0	1	0	0	0	0	3	1
626	69	2	0	0	1	0	1	1	1	0	1	0	0	0	0	3	2
628	78	2	0	0	0	0	1	1	0	1	0	0	1	0	0	2	2
632	69	2	0	0	0	0	1	0	0	1	0	0	1	0	0	4	2
634	69	1	0	0	1	1	1	0	1	1	0	0	0	1	1	3	4
636	71	1	0	1	0	0	1	0	1	1	1	0	0	0	0	1	4
638	71	2	0	0	1	#NULL!	1	#NULL!	0	0	1	1	0	0	0	3	3
639	66	1	0	0	#NULL!	1	1	#NULL!	1	0	0	0	0	0	0	1	4
640	79	1	0	0	0	0	1	0	1	1	1	1	0	0	0	3	4
641	71	2	0	0	1	1	1	1	0	0	1	0	0	0	0	3	1
645	72	1	0	0	0	1	1	0	0	0	1	0	0	0	0	2	4
646	70	2	0	0	0	1	1	2	0	0	0	1	0	0	0	2	4
647	79	1	0	0	0	1	1	1	0	1	0	0	0	0	0	1	4
648	71	2	0	0	0	1	3	1	0	0	0	0	1	0	0	4	1
649	69	1	0	0	1	1	1	0	1	0	0	0	0	0	0	1	4
651	66	1	0	0	0	0	1	0	1	0	1	1	0	0	0	2	4
652	66	2	0	0	0	0	1	0	0	0	1	0	0	0	0	2	2
653	73	1	0	0	0	1	1	1	1	1	0	1	0	0	0	1	4
654	68	2	0	0	0	0	1	0	0	0	0	0	0	0	0	1	2
655	71	1	0	0	0	0	1	0	0	1	0	0	0	0	1	1	4
656	66	2	0	0	0	0	1	0	0	0	0	0	0	0	1	2	4
657	71	1	0	0	0	1	3	1	0	1	1	1	0	0	0	3	1
658	66	2	0	0	0	0	3	0	0	0	1	1	1	0	0	1	3
661	69	2	0	0	1	0	1	0	0	1	0	0	0	0	0	3	1
662	66	1	1	0	1	0	1	#NULL!	0	1	1	0	0	0	0	3	4
666	68	1	1	0	0	1	1	1	1	0	1	0	1	0	0	1	4
667	66	2	0	0	0	1	1	0	0	0	1	1	0	0	0	2	2
671	75	2	0	0	1	0	1	0	0	0	0	0	0	0	0	2	2
674	67	1	0	0	0	0	1	0	0	0	0	0	0	0	1	1	4
675	88	2	1	1	1	0	1	0	0	0	0	0	0	0	0	2	1
680	68	2	1	0	0	0	1	0	1	0	1	0	0	0	0	1	4
682	76	2	0	0	0	1	1	1	0	0	0	1	1	0	0	4	1

685	73	2	0	0	0	1	1	1	0	0	0	1	0	0	0	1	3
686	73	1	0	0	0	0	1	0	0	0	0	0	0	0	0	1	4
687	75	2	0	0	0	1	2	0	0	0	0	1	0	0	0	1	4
688	71	1	0	0	0	#NULL!	1	0	0	1	1	1	0	#NULL!	0	1	4
689	69	2	0	0	0	1	1	1	0	0	1	0	0	0	0	2	2
690	68	1	0	0	0	1	3	0	0	1	1	0	0	0	1	2	4
693	73	1	0	1	0	0	1	1	1	1	1	1	0	0	0	1	4
696	67	2	0	0	0	1	1	0	0	0	0	0	0	0	1	3	4
699	79	2	0	0	1	0	1	#NULL!	0	0	0	0	0	0	0	4	4
700	77	1	0	0	1	0	1	1	0	0	0	0	0	0	0	1	4
701	71	1	0	0	0	0	1	1	1	1	1	1	0	0	1	2	4
702	67	2	0	0	0	0	3	1	0	1	0	0	0	0	0	2	2
709	72	1	0	0	0	1	1	0	1	0	1	1	0	0	0	1	4
712	75	1	1	0	0	0	1	1	1	1	0	1	0	1	1	1	4
715	79	2	1	0	1	1	1	1	0	0	1	0	0	0	0	3	3
718	66	2	0	0	0	0	2	#NULL!	1	0	0	0	0	0	1	1	2
720	82	1	1	1	0	0	1	0	1	1	1	0	0	0	0	2	4
721	69	1	1	0	0	1	1	1	1	1	0	0	0	0	0	3	1
722	66	2	0	0	0	1	1	2	0	0	1	0	0	0	0	2	3
725	71	2	0	0	0	#NULL!	1	0	0	0	0	0	1	0	0	4	4
733	71	1	0	0	0	0	1	2	0	1	1	1	0	0	0	1	4
734	70	2	0	0	0	0	3	0	0	0	1	0	0	0	0	2	3
735	72	1	0	0	0	1	1	0	0	1	0	0	0	0	1	1	4
736	69	2	0	0	0	0	3	1	0	0	1	0	0	0	0	2	2
738	74	1	0	1	0	0	3	1	0	0	1	1	0	0	0	3	4
739	69	1	0	0	0	0	1	#NULL!	#NULL!	#NULL!	1	1	#NULL!	0	0	1	4
740	67	2	0	0	0	#NULL!	1	1	0	0	0	0	0	0	0	1	1
742	73	2	0	0	0	1	1	0	0	0	0	0	0	0	0	2	4
743	72	1	1	0	0	0	1	2	1	1	0	0	1	0	0	4	1
744	67	2	0	0	0	0	1	1	1	1	1	0	0	0	0	1	2
745	80	1	0	0	0	0	1	1	0	1	0	0	1	0	0	1	4
746	77	2	0	0	0	0	3	1	0	0	0	0	0	0	0	4	1
747	81	1	0	0	0	0	1	1	0	1	0	0	0	1	0	1	4
750	72	1	0	0	0	#NULL!	1	2	1	1	1	0	0	#NULL!	0	1	4
751	68	2	0	0	0	1	1	0	0	0	0	0	1	0	0	2	1
752	70	2	0	1	0	0	1	1	0	0	0	1	0	0	1	2	1
753	72	1	0	0	0	1	1	2	1	1	0	0	0	0	0	1	4
755	68	2	0	1	0	1	1	0	1	0	0	0	0	0	1	1	4
756	69	1	0	0	0	#NULL!	1	1	1	1	0	0	0	0	0	2	4

757	67	2	0	0	0	0	3	0	0	0	1	0	0	0	0	2	4
758	66	1	0	0	0	0	3	1	1	1	1	1	0	0	1	2	4
759	65	1	0	0	0	#NULL!	3	0	0	1	0	0	0	0	1	1	4
761	78	1	0	0	0	0	2	1	0	0	1	1	0	0	0	1	1
762	65	2	0	0	0	0	2	0	0	0	1	0	0	0	0	1	4
765	67	2	1	0	#NULL!	1	3	0	0	0	0	0	0	0	0	3	3
767	72	2	0	1	0	0	1	0	0	0	0	0	0	0	0	2	4
769	87	2	1	1	0	0	1	1	0	0	0	0	0	1	0	2	3
770	68	2	0	0	0	0	3	0	0	0	1	0	0	0	0	2	4
771	70	1	1	0	1	1	1	0	0	1	1	0	0	0	1	3	4
772	69	2	0	0	1	1	1	1	0	0	1	0	0	0	0	2	2
773	70	1	0	0	0	1	1	0	0	1	0	0	1	0	0	3	4
774	67	2	0	0	0	1	1	1	0	1	0	0	0	0	0	1	1
775	72	1	0	0	0	#NULL!	1	0	1	1	1	0	0	0	0	1	4
776	71	2	0	0	0	0	1	0	0	0	1	0	0	0	0	2	2
782	79	1	0	0	0	1	1	0	0	0	1	0	0	0	0	2	4
788	72	1	1	0	1	0	1	1	0	1	0	0	0	1	0	4	1
789	65	2	0	0	0	0	1	0	0	#NULL!	1	0	0	0	0	3	3
793	75	2	0	0	0	1	1	1	0	0	0	1	0	0	0	2	4
801	74	1	0	0	0	1	3	1	1	1	1	1	0	0	0	2	4
806	70	1	1	0	0	0	1	1	0	1	0	0	0	0	0	1	4
807	81	2	0	1	0	1	1	0	0	0	0	0	0	0	0	2	1
814	81	2	0	0	0	0	2	1	0	0	1	0	0	0	0	2	4
816	77	2	0	0	0	#NULL!	3	0	0	1	0	0	0	0	0	2	2
819	71	2	0	0	1	1	1	1	1	0	1	0	0	0	0	2	4
822	73	1	0	1	1	0	3	0	0	0	1	1	0	0	0	3	4
824	69	1	0	0	0	0	1	0	1	1	0	0	0	0	1	3	4
826	76	2	0	#NULL!	1	1	1	1	0	0	0	0	0	0	0	4	4
827	88	1	1	0	1	1	1	1	0	0	1	0	0	0	0	3	4
829	66	2	0	0	0	0	1	0	0	0	1	1	0	0	1	2	4
831	70	1	0	0	0	1	1	0	1	0	0	0	0	0	1	1	4
833	65	2	0	0	0	1	1	0	0	0	1	0	0	0	0	2	2
835	70	1	0	0	0	0	1	0	0	1	0	1	0	0	0	2	3
837	72	2	0	1	0	1	3	1	0	0	1	1	0	0	0	4	1
838	83	2	0	1	0	1	3	2	0	0	0	0	0	0	0	3	4
839	73	1	0	0	0	0	1	2	0	1	0	0	0	0	0	1	4
840	69	2	0	0	0	0	1	0	0	0	0	0	0	0	0	2	1
850	80	2	0	1	0	1	3	0	0	0	0	0	1	0	0	2	3
852	67	1	0	#NULL!	0	#NULL!	1	#NULL!	#NULL!	#NULL!	1	0	#NULL!	1	0	1	4

853	67	2	0	0	0	0	1	0	0	0	1	0	0	0	0	2	3
854	79	2	1	1	1	1	1	2	0	0	1	0	1	0	0	4	4
855	72	1	0	0	0	1	1	0	1	1	0	0	0	0	0	1	4
856	67	2	0	0	0	1	3	0	0	0	0	0	0	0	0	2	1
857	73	1	0	0	0	0	1	1	1	1	1	0	0	0	0	2	3
858	69	2	0	0	0	0	1	0	0	0	1	1	0	0	0	4	1
859	76	2	0	0	0	1	1	0	0	0	0	0	0	0	0	3	4
860	71	1	0	0	0	1	1	0	0	0	0	0	0	0	0	2	4
862	83	1	0	0	1	0	3	1	0	0	1	0	0	0	0	3	4
863	81	2	1	0	0	0	1	#NULL!	0	0	0	0	0	0	0	1	1
865	82	2	0	0	0	0	1	1	0	0	0	0	0	0	1	2	4
866	81	1	0	0	0	0	1	#NULL!	#NULL!	#NULL!	0	1	#NULL!	0	0	1	4
867	77	1	0	0	0	0	1	0	1	0	1	1	0	0	1	1	4
870	69	1	0	0	0	0	1	2	1	1	0	0	0	0	1	1	4
871	66	1	0	0	0	0	1	#NULL!	1	1	1	1	0	0	1	1	1
873	65	1	0	0	0	0	1	1	0	1	0	0	0	1	0	1	4
874	84	2	0	1	0	1	1	1	0	0	1	0	0	0	0	3	3
876	71	1	0	0	0	0	1	2	1	1	0	0	0	0	1	2	4
877	68	2	0	0	0	0	1	1	0	0	1	0	0	0	1	1	4
879	69	2	0	0	0	0	1	0	0	0	1	1	0	0	0	2	4
881	67	2	0	0	0	1	1	0	1	0	1	0	0	0	0	2	2
883	68	1	0	0	0	1	1	0	0	1	0	0	0	0	0	4	4
885	65	1	0	0	0	1	3	1	0	1	0	0	0	0	0	1	4
886	72	1	1	0	0	0	2	2	1	1	0	0	0	0	0	1	4
887	71	2	0	0	0	0	1	0	1	0	1	1	0	0	1	2	4
890	74	2	0	0	0	1	1	2	0	0	1	0	1	0	0	1	4
894	72	2	0	1	0	1	2	0	0	0	1	0	0	0	0	2	4
896	100	2	0	1	1	1	1	0	0	0	0	0	0	1	0	3	2
898	74	1	1	0	1	1	1	1	1	1	0	0	0	0	0	2	4
899	72	2	0	0	1	1	1	0	0	0	0	0	0	0	0	2	3
905	65	2	0	0	0	1	1	0	0	0	0	1	0	0	0	2	2
913	83	1	0	0	1	1	3	1	1	1	0	0	1	1	0	1	4
918	65	1	0	0	0	0	3	1	0	1	0	1	0	0	1	1	4
921	71	1	0	0	0	0	3	#NULL!	#NULL!	#NULL!	1	0	#NULL!	0	1	1	4
922	65	2	0	0	0	0	1	0	0	0	0	0	0	0	0	4	1
923	70	1	0	0	0	0	1	0	0	0	0	0	0	1	0	2	4
926	73	1	0	0	0	0	1	0	1	0	0	0	0	0	0	1	4
928	71	2	0	#NULL!	0	#NULL!	1	#NULL!	#NULL!	#NULL!	0	0	#NULL!	0	0	1	4
929	71	1	0	0	0	0	1	0	1	1	0	0	0	1	1	1	4

933	66	2	1	0	0	0	1	0	0	0	1	0	1	0	0	2	2
937	73	1	0	1	0	1	1	0	1	1	1	0	0	0	1	1	4
938	77	1	0	0	0	0	1	0	0	0	0	0	0	0	0	2	4
939	71	2	0	0	0	0	2	0	0	0	1	0	0	0	0	2	1
944	69	1	0	0	0	0	1	0	0	1	0	0	0	0	0	1	4
946	72	1	0	0	0	0	1	0	0	0	0	0	0	0	0	2	4
947	70	1	0	0	0	1	1	0	1	1	1	1	0	0	0	2	4
948	67	2	0	0	0	#NULL!	1	1	0	0	1	0	0	0	0	2	2
949	67	1	0	0	0	1	#NULL!	2	0	1	1	0	1	1	1	4	4
952	70	2	0	0	1	0	1	1	0	0	1	1	0	0	1	2	2
954	66	1	0	0	0	0	3	2	1	1	1	0	0	0	0	2	4
955	68	1	0	0	0	1	1	0	0	1	1	1	0	1	1	2	4
956	68	2	0	0	0	1	3	1	0	0	1	1	0	0	0	2	3
957	66	1	0	0	0	0	3	0	1	1	1	0	0	0	1	2	4
959	73	2	0	1	0	0	1	0	0	0	1	0	0	0	1	1	4
961	87	1	1	0	0	#NULL!	1	1	0	1	0	0	1	0	0	2	4
962	85	2	0	0	1	#NULL!	1	1	0	0	0	0	1	0	0	3	2
965	67	1	1	0	0	1	3	1	1	1	1	1	0	0	0	2	4
966	76	2	0	1	0	0	1	0	0	0	0	0	0	0	1	3	4
967	70	1	0	0	0	0	1	1	0	0	1	0	0	0	0	1	4
968	66	2	0	0	0	0	1	0	0	0	1	0	0	0	0	2	2
969	75	1	1	1	0	0	1	1	0	1	1	1	0	0	0	1	4
970	74	1	1	0	0	1	1	1	0	1	0	0	#NULL!	0	0	3	4
972	69	1	0	0	0	0	1	1	1	0	1	1	0	0	0	2	4
973	67	1	0	0	0	1	1	#NULL!	0	0	1	1	0	0	0	2	4
978	67	1	0	0	0	1	3	0	1	0	1	0	0	0	1	2	4
979	67	1	0	0	1	1	1	0	1	1	0	0	1	0	1	3	4
980	84	1	1	0	1	1	1	2	1	1	0	0	0	0	0	2	4
983	78	2	1	1	0	1	1	2	0	0	0	0	0	0	0	4	1
984	78	1	0	0	0	0	3	1	0	0	0	0	0	0	1	1	4
985	75	2	0	0	0	0	2	#NULL!	0	0	1	1	1	0	0	2	1
987	78	2	0	0	0	0	#NULL!	0	0	0	0	0	0	0	0	4	1
990	82	2	0	1	0	0	1	1	0	0	1	1	0	0	0	4	1
992	80	2	1	0	1	0	1	0	0	0	0	0	0	1	0	1	2
996	75	2	0	1	0	1	1	1	0	1	1	1	0	0	1	3	2
997	70	1	1	0	0	0	3	#NULL!	1	1	1	0	0	1	1	1	4
1000	67	2	0	0	0	1	1	#NULL!	0	1	1	0	0	0	0	2	4
1002	72	1	0	0	1	#NULL!	1	1	0	1	1	0	0	0	0	3	4
1003	67	2	0	0	0	1	1	0	0	0	1	0	0	0	0	2	4

1004	80	1	0	0	0	1	2	2	1	1	1	1	1	0	0	1	4
1005	70	2	1	0	0	1	1	0	0	0	1	1	1	0	0	1	1
1007	67	2	0	0	0	0	1	0	0	0	1	0	0	0	0	1	1
1011	70	2	0	0	0	1	2	1	0	0	0	1	0	0	0	3	2
1015	65	1	0	0	0	0	3	1	1	0	0	0	0	0	1	1	4
1017	74	2	0	#NULL!	0	#NULL!	1	0	0	0	1	0	0	0	1	1	1
1018	65	1	0	0	0	1	1	1	0	0	1	1	0	0	1	1	4
1019	74	1	0	0	0	1	1	0	1	1	1	0	0	0	0	2	4
1020	66	2	0	0	1	1	1	1	0	0	1	0	0	0	0	3	1
1021	80	2	0	1	0	0	1	1	0	0	0	0	0	0	0	4	1
1022	78	2	1	1	0	1	1	0	1	0	0	0	0	0	0	3	3
1025	78	1	0	0	1	#NULL!	1	2	0	0	1	1	0	0	0	3	4
1026	76	2	0	0	1	1	1	1	0	0	0	0	0	0	0	3	2
1027	74	1	0	0	0	0	1	0	0	0	1	1	0	0	1	3	4
1029	70	1	0	0	0	1	1	1	0	1	1	0	0	0	0	1	4
1030	69	2	0	0	0	1	3	0	0	0	1	0	0	0	0	2	2
1032	83	1	1	#NULL!	0	#NULL!	1	0	0	1	0	0	0	0	1	1	4
1033	69	1	0	0	#NULL!	1	1	#NULL!	1	1	1	0	0	0	0	1	4
1034	66	2	0	0	0	1	1	#NULL!	#NULL!	#NULL!	1	0	#NULL!	0	0	3	4
1040	80	2	1	0	0	1	1	1	0	0	1	0	1	0	0	2	2
1041	71	1	0	0	0	1	1	1	0	1	0	0	0	0	1	2	4
1042	78	1	0	0	0	0	1	0	0	0	1	1	0	0	0	1	4
1044	75	2	0	0	0	0	1	0	0	0	1	1	0	0	0	4	1
1045	76	2	1	1	1	0	2	#NULL!	0	0	0	0	0	0	0	4	2
1046	69	2	0	#NULL!	0	#NULL!	1	#NULL!	#NULL!	#NULL!	1	0	#NULL!	0	0	2	2
1047	69	1	0	0	0	1	1	0	1	1	0	0	1	0	1	2	4
1048	86	2	0	1	0	0	1	0	0	0	1	0	0	0	0	4	1
1049	82	1	1	0	0	0	1	0	0	1	0	0	1	0	0	3	4
1050	76	2	0	0	0	0	1	1	0	0	0	0	0	0	0	4	1
1052	74	1	0	0	0	1	3	1	0	1	0	0	0	0	1	2	4
1053	72	2	0	0	1	1	1	1	0	0	0	0	0	0	0	2	4
1062	88	1	0	0	0	0	1	0	1	1	0	0	0	1	0	1	1
1064	65	1	0	0	1	1	3	1	0	0	0	0	1	1	1	1	4
1066	74	1	0	0	0	0	1	2	0	1	1	0	0	0	0	1	4
1069	66	2	0	1	1	0	1	#NULL!	0	0	1	0	0	0	0	4	1
1071	68	1	0	0	0	1	1	1	0	0	0	0	0	1	1	2	4
1072	66	1	0	0	0	0	1	1	1	0	0	1	0	1	1	1	4
1073	71	1	0	1	0	0	1	1	1	1	1	1	0	0	1	1	4
1076	78	1	0	0	0	1	1	1	0	0	1	0	1	0	0	1	4

1077	72	2	0	0	0	1	1	1	0	0	0	0	0	0	0	4	1
1080	66	1	0	0	#NULL!	1	1	0	0	1	0	0	0	0	0	2	4
1083	72	1	0	1	0	1	1	0	1	0	0	0	0	0	1	4	4
1084	69	1	0	0	0	1	1	1	0	0	0	0	0	1	1	2	4
1085	66	2	0	1	0	1	1	0	0	0	0	0	0	0	0	2	3
1086	66	1	0	1	0	1	1	0	0	0	1	0	0	0	0	1	4
1089	65	1	0	0	0	1	1	1	1	1	0	0	0	0	1	2	2
1094	84	1	1	0	0	#NULL!	1	0	1	1	1	1	0	0	0	1	4
1095	77	2	0	0	1	0	1	0	0	0	1	1	1	0	0	3	1
1102	65	2	0	0	0	1	3	1	1	1	1	0	0	0	1	2	4
1103	70	1	0	0	1	1	1	2	1	1	0	0	0	0	0	1	4
1104	66	2	1	0	1	1	1	0	0	1	0	0	0	0	0	1	3
1105	78	1	0	1	0	0	1	0	1	1	1	1	0	0	0	3	1
1108	77	2	1	1	0	1	1	0	0	0	1	0	0	0	0	2	2
1110	72	2	0	0	0	1	1	0	0	0	0	0	0	0	0	2	3
1111	69	2	0	1	0	0	3	2	0	0	0	0	1	0	1	3	4
1112	72	1	0	1	1	0	1	1	0	1	0	0	0	0	0	3	4
1114	82	1	0	0	0	1	1	0	0	1	1	0	0	0	0	2	4
1117	84	1	1	1	0	1	3	#NULL!	1	0	0	0	0	0	0	3	4
1118	86	1	0	0	1	1	3	1	1	0	0	0	0	1	0	1	4
1119	82	2	0	0	0	1	2	0	0	0	0	0	0	0	0	2	2
1120	69	2	0	1	0	0	3	2	0	0	0	0	0	0	0	2	4
1122	71	1	0	0	1	1	1	0	1	0	0	0	0	0	0	1	1
1123	70	2	0	0	0	1	1	1	0	0	1	0	0	0	0	2	3
1126	70	1	1	0	0	1	1	1	0	1	0	0	1	0	1	2	4
1127	69	2	0	0	0	1	2	0	0	0	0	0	0	0	0	2	4
1129	68	2	0	0	0	1	1	0	0	0	1	0	1	0	0	2	3
1132	70	1	0	1	0	1	1	0	1	0	0	0	0	0	1	1	4
1134	77	1	0	0	0	1	1	1	1	1	0	0	1	0	0	3	4
1135	72	2	0	0	1	1	3	0	0	0	0	0	0	0	0	1	4
1136	67	2	0	0	1	1	#NULL!	1	0	0	0	0	0	0	1	3	4
1139	78	2	0	0	0	1	1	0	0	0	0	0	0	0	0	2	1
1141	76	2	1	0	0	1	1	0	0	0	0	0	0	0	0	2	2
1142	76	2	0	0	#NULL!	#NULL!	1	0	0	0	0	0	0	0	0	2	4
1143	72	1	0	0	0	#NULL!	1	1	0	1	0	0	1	0	1	1	4
1144	77	1	1	0	0	0	3	1	1	1	0	1	1	0	0	3	4
1145	73	2	1	0	0	0	1	0	0	0	1	1	1	0	0	3	2
1146	77	2	0	1	1	1	3	#NULL!	0	0	0	0	1	0	0	3	2
1147	75	1	0	0	0	0	1	1	0	1	0	0	0	1	0	1	4

1148	70	2	0	0	0	0	2	0	0	0	1	0	0	0	0	2	3
1149	69	2	0	1	1	1	1	1	0	0	0	0	0	0	0	3	4
1151	73	2	0	0	1	1	1	0	0	0	0	1	0	0	0	3	3
1154	71	2	0	1	0	1	1	#NULL!	#NULL!	#NULL!	0	0	#NULL!	0	0	1	4
1156	75	1	0	0	1	1	3	1	1	1	0	0	0	1	1	1	4
1157	66	1	0	0	0	1	1	0	0	1	0	0	0	0	0	3	4
1158	66	2	0	1	1	1	1	0	0	0	1	0	0	0	1	3	4
1160	83	1	0	0	1	1	1	2	0	0	0	0	1	0	0	2	4
1163	83	2	1	0	0	1	1	#NULL!	#NULL!	#NULL!	0	0	#NULL!	1	0	2	1
1166	76	1	1	0	0	1	1	1	0	0	0	0	0	1	0	1	4
1167	70	2	0	0	0	1	2	0	0	0	0	1	0	0	0	2	2
1168	65	1	0	0	1	1	1	0	1	1	0	0	0	0	1	2	4
1170	77	2	1	1	0	1	1	2	0	0	0	0	0	0	0	1	4
1175	82	1	0	0	0	1	1	1	0	1	0	1	0	0	1	4	4
1180	80	2	0	0	0	1	1	0	0	0	0	0	0	0	0	3	2
1181	80	2	0	1	0	1	2	1	0	0	0	0	0	0	0	2	3
1186	67	2	0	1	0	1	1	0	0	0	0	0	0	0	0	2	2
1190	73	2	0	0	0	1	1	1	0	1	0	0	1	1	0	2	1
1191	69	1	0	0	0	1	1	0	0	0	0	0	1	0	0	1	4
1192	73	1	0	0	0	1	1	0	0	1	0	0	0	0	0	4	1
1193	69	2	0	0	0	1	1	0	0	0	0	0	0	0	0	4	1
1194	79	2	1	0	0	0	2	1	0	0	0	0	0	0	0	2	2
1195	72	1	0	0	0	0	1	2	1	1	1	0	0	0	0	2	4
1196	80	2	0	1	0	#NULL!	1	0	0	0	0	0	0	0	0	2	1
1197	68	2	0	0	0	1	1	1	1	0	1	0	0	0	0	2	3
1198	79	2	0	1	0	1	1	1	0	0	1	0	0	0	0	4	1
1199	68	1	0	0	0	1	1	0	0	1	0	0	0	0	1	2	4
1200	66	2	0	0	0	1	1	0	0	0	0	0	0	0	1	1	2
1202	73	2	0	0	1	1	1	0	0	0	0	0	1	0	0	1	2
1205	82	1	0	0	0	0	1	2	0	1	1	1	1	0	0	2	4
1206	80	2	0	0	0	1	1	1	0	0	0	0	1	0	0	2	2
1210	67	2	0	1	0	1	3	0	0	1	1	0	1	0	0	2	4
1212	79	2	1	0	1	1	1	1	0	0	0	0	1	0	0	3	1
1216	80	1	1	0	1	#NULL!	3	#NULL!	1	1	0	0	0	0	0	3	4
1217	78	2	0	0	1	0	1	1	0	0	0	0	0	0	0	2	3
1219	75	1	0	0	0	1	1	0	0	1	0	0	0	0	0	1	1
1220	72	2	0	0	0	1	1	1	0	0	0	0	0	0	0	3	1
1221	80	1	0	0	1	1	1	0	0	0	1	1	0	0	0	3	4
1222	79	2	1	0	1	1	1	1	0	0	0	1	0	0	0	4	1

1223	73	1	0	0	0	0	3	1	0	0	0	0	0	0	0	3	4
1224	68	2	0	0	0	0	1	0	0	0	0	0	0	0	0	2	2
1225	74	2	0	1	1	1	3	0	0	0	0	1	0	0	0	3	4
1232	73	1	0	0	0	0	1	1	0	1	0	0	0	0	0	1	4
1233	65	2	0	0	0	0	3	0	0	0	0	0	0	0	0	2	3
1235	78	1	1	0	0	0	1	1	0	0	1	1	0	1	1	3	4
1236	77	2	0	0	0	#NULL!	1	0	0	0	0	0	0	0	0	1	4
1238	79	2	0	1	0	#NULL!	3	1	0	0	1	0	0	0	0	1	3
1240	77	1	1	0	0	1	1	1	0	1	1	0	1	0	0	1	4
1241	70	2	0	0	0	1	1	1	0	1	0	0	0	0	0	2	4
1246	66	1	0	0	0	1	2	0	0	0	0	0	0	0	1	2	4
1247	69	1	0	0	0	#NULL!	1	0	1	0	0	0	0	0	1	2	4
1249	77	1	0	0	1	0	1	0	0	1	0	0	1	1	0	3	4
1250	76	2	0	0	1	0	1	#NULL!	0	0	0	0	0	0	0	3	3
1254	68	2	0	0	1	1	1	0	0	0	0	0	0	0	0	3	1
1255	66	2	0	1	0	1	2	1	0	0	0	0	1	0	0	2	3
1256	82	2	0	1	0	1	1	1	0	0	0	0	0	0	0	2	2
1257	71	1	0	0	0	1	2	1	0	1	0	0	0	0	1	2	4
1260	81	1	0	1	0	0	1	0	0	1	0	0	0	0	0	1	4
1262	75	2	1	1	0	0	1	0	0	0	1	0	1	0	0	1	2
1264	85	2	0	1	1	0	2	1	0	0	0	0	0	0	0	4	1
1267	71	1	0	1	0	1	1	2	0	1	0	0	0	0	0	2	4
1270	65	1	0	0	1	1	3	0	0	1	0	0	0	0	1	3	4
1271	74	1	0	0	0	1	3	2	0	0	0	0	0	0	0	3	4
1272	69	2	0	0	1	1	1	0	0	0	0	0	0	0	0	2	1
1277	67	1	0	0	0	1	1	0	0	1	1	0	0	0	0	4	1
1278	66	2	0	0	1	1	1	0	0	0	1	0	0	0	0	1	2
1281	70	1	0	0	0	1	1	1	1	1	1	1	0	0	0	1	4
1282	77	1	0	0	1	1	1	0	0	1	1	1	0	0	0	1	4
1283	76	2	0	0	1	1	1	0	0	0	0	0	0	0	0	1	2
1287	71	1	0	0	1	1	3	1	0	0	0	0	0	0	0	3	4
1288	73	2	0	1	1	#NULL!	#NULL!	2	1	0	0	0	0	0	0	2	2
1289	73	1	0	0	0	1	1	0	0	1	1	0	1	1	0	1	4
1290	67	2	0	0	0	1	1	1	0	0	1	1	0	0	0	2	1
1292	70	2	0	0	0	1	2	1	0	0	0	0	0	0	0	2	2
1295	66	1	0	0	0	0	1	0	1	1	1	0	0	0	1	4	4
1296	68	2	0	1	0	0	1	2	1	1	1	0	0	0	0	2	2
1298	80	2	1	1	0	1	1	#NULL!	#NULL!	#NULL!	0	0	#NULL!	0	0	2	3
1303	65	1	0	0	0	1	1	1	1	1	0	0	0	0	0	2	4

1304	66	2	0	1	0	0	2	0	1	1	1	0	0	0	0	2	4
1305	65	1	0	0	0	1	1	1	1	1	0	0	1	0	1	1	4
1307	75	2	1	0	0	0	1	0	0	0	0	0	0	0	0	4	4
1308	71	1	0	0	0	1	1	2	1	1	#NULL!	#NULL!	0	0	1	1	4
1309	66	2	0	0	0	1	1	#NULL!	0	1	1	1	0	0	0	2	2
1310	75	2	0	1	1	1	1	0	0	1	0	0	1	0	0	4	1
1311	77	1	0	0	0	#NULL!	1	0	0	1	0	0	0	0	1	1	4
1314	66	1	0	0	0	1	1	0	1	1	1	0	0	0	0	1	4
1315	76	1	1	0	0	1	1	0	0	1	0	0	1	1	0	2	4
1316	73	2	0	0	0	1	1	1	0	0	0	0	1	0	0	1	1
1319	72	1	0	0	1	1	1	#NULL!	0	1	0	0	0	0	0	2	4
1322	67	1	0	0	0	1	1	1	1	1	0	0	1	0	1	1	2
1323	74	2	1	1	1	1	1	1	0	0	0	0	1	0	0	2	4
1326	67	1	0	0	0	0	1	0	0	1	0	0	0	0	0	1	4
1327	71	1	0	0	1	1	1	1	1	1	0	0	1	0	0	1	4
1333	80	2	1	0	1	1	1	#NULL!	0	0	0	0	0	0	0	3	4
1335	75	1	1	1	#NULL!	1	1	#NULL!	#NULL!	#NULL!	1	0	#NULL!	0	0	4	4
1336	70	2	1	0	1	1	1	0	1	0	1	0	0	0	0	3	3
1337	75	1	0	0	0	1	3	0	1	1	1	0	0	1	0	1	4
1340	66	1	0	0	0	1	3	2	0	1	0	0	0	0	1	2	4
1349	89	2	1	0	0	1	1	1	0	0	0	1	1	0	0	1	2
1350	88	1	1	0	0	1	1	1	0	0	0	0	1	0	1	1	4
1352	84	2	0	0	0	0	2	0	0	0	0	0	0	0	0	2	2
1353	74	1	0	0	0	0	3	0	0	1	0	0	0	0	0	3	4
1354	77	2	0	0	0	1	2	0	0	0	0	0	1	0	0	4	1
1355	76	1	0	0	1	1	3	1	0	0	0	0	0	0	1	1	4
1356	66	2	0	1	0	1	1	#NULL!	0	0	0	0	0	0	0	2	3
1358	73	1	0	0	0	1	1	0	0	1	0	0	0	0	0	1	4
1364	70	1	1	0	0	0	3	0	0	1	0	1	0	0	0	1	4
1365	67	2	0	1	0	0	1	#NULL!	0	0	0	0	0	0	1	2	4
1367	78	1	0	1	0	0	1	1	1	1	0	1	1	0	0	1	4
1368	66	1	0	0	0	1	3	1	0	1	1	0	0	0	1	1	4
1370	68	1	0	0	0	#NULL!	1	0	1	0	0	0	0	0	1	1	4
1373	69	1	0	0	0	1	1	2	0	1	1	1	0	0	0	2	4
1374	82	1	0	1	0	1	3	#NULL!	#NULL!	#NULL!	0	0	#NULL!	0	0	2	4
1377	75	1	0	0	0	0	3	#NULL!	#NULL!	#NULL!	1	0	#NULL!	0	1	4	4
1381	71	2	0	0	0	0	1	1	1	0	1	0	0	0	0	2	4
1383	71	1	1	0	0	0	3	1	1	1	0	0	1	1	0	1	4
1384	70	2	0	0	0	0	1	2	1	0	1	1	0	0	0	1	1

1385	75	1	0	0	0	0	1	0	0	1	1	1	0	0	0	1	4
1387	73	1	1	0	0	0	1	1	1	1	1	1	0	0	0	2	4
1388	71	2	0	0	1	0	1	0	1	0	0	1	0	0	0	2	3
1391	76	2	0	1	0	1	1	0	0	0	1	0	0	0	0	2	4
1393	68	2	0	0	0	1	2	#NULL!	0	0	0	0	0	0	0	2	2
1394	71	1	0	0	0	1	1	1	0	0	0	1	0	0	0	1	4
1395	67	2	0	0	0	1	1	1	0	0	0	1	0	0	1	1	4
1397	69	2	0	1	0	1	3	1	0	0	0	0	0	0	0	1	2
1398	80	2	0	1	0	0	1	1	1	0	1	1	0	0	0	4	1
1399	70	1	0	0	0	1	1	0	1	0	1	0	0	0	1	1	4
1400	66	2	0	0	0	1	3	1	1	0	1	0	0	0	0	2	2
1401	70	1	0	0	0	0	1	0	0	1	0	1	0	0	0	1	4
1402	72	2	0	0	0	1	1	0	0	1	0	0	0	0	0	2	4
1403	69	1	0	0	0	0	#NULL!	1	0	0	1	1	0	0	1	1	4
1405	69	1	1	0	0	1	1	1	0	1	0	0	0	0	0	1	4
1406	69	2	0	0	0	1	2	0	0	0	1	1	0	0	0	2	2
1409	66	1	0	0	0	0	3	1	0	0	1	0	0	0	0	1	4
1412	65	2	0	0	0	0	1	0	0	0	0	0	0	0	0	2	3
1413	89	2	0	1	0	#NULL!	1	0	0	0	0	0	0	0	0	3	2
1414	66	1	0	0	0	1	3	1	1	1	0	0	0	1	0	2	4
1416	74	1	0	0	0	0	1	2	0	1	0	0	0	1	0	1	4
1417	69	2	1	0	0	1	1	0	0	0	0	0	0	0	0	1	2
1418	79	2	0	0	0	1	1	0	0	0	0	0	0	0	0	4	1
1419	78	1	0	0	0	1	1	1	0	1	0	0	1	0	0	4	1
1421	71	1	1	0	0	1	1	1	0	1	0	0	0	0	0	2	4
1422	67	2	0	0	0	0	2	0	0	0	0	0	0	0	0	4	1
1423	67	1	0	0	0	0	1	#NULL!	#NULL!	#NULL!	1	1	#NULL!	0	0	2	4
1424	70	1	0	0	0	0	1	0	1	1	1	0	0	0	1	1	4
1425	84	2	1	1	0	0	1	0	0	0	1	0	0	0	0	1	4
1426	69	1	0	0	0	1	1	2	1	0	0	0	0	0	0	1	4
1428	80	2	1	1	0	0	1	#NULL!	1	0	0	0	1	0	0	4	4
1429	76	1	1	0	0	0	1	1	1	1	0	0	0	0	0	1	4
1430	73	2	0	0	0	0	1	1	0	0	0	0	0	0	0	4	1
1432	65	2	0	0	0	1	3	1	1	0	0	0	0	0	0	3	3
1433	65	1	0	0	0	1	1	1	1	1	0	0	0	0	0	1	4
1435	66	2	0	0	0	1	1	0	0	0	0	0	1	0	0	2	2
1436	66	2	0	1	0	1	3	1	0	0	0	0	0	0	0	2	2
1440	67	1	0	0	0	1	1	#NULL!	0	0	1	1	0	0	0	3	4
1441	67	2	1	0	0	1	1	0	0	0	0	1	1	0	0	2	3

1443	68	1	0	0	0	1	3	1	0	1	0	0	1	0	0	1	4
1444	67	2	0	0	1	1	3	1	0	0	0	0	0	0	0	3	2
1445	66	2	0	1	0	1	2	1	0	0	1	0	1	0	0	2	1
1446	70	1	0	0	0	0	3	2	1	1	0	0	0	0	0	2	4
1447	65	2	0	0	1	0	3	1	0	0	0	0	0	0	0	3	1
1449	69	1	0	0	0	0	3	1	0	1	0	0	0	0	0	1	4
1450	66	1	0	0	0	0	1	0	0	1	1	0	0	0	0	1	4
1451	67	1	0	0	0	#NULL!	1	0	1	1	0	0	1	0	0	1	4
1452	72	2	0	1	0	0	1	0	0	0	0	0	0	0	0	2	1
1457	68	1	0	0	0	0	1	2	1	1	1	0	1	0	1	2	4
1458	65	2	1	0	0	0	1	1	1	0	1	1	0	0	0	3	3
1460	68	1	0	0	0	1	1	1	1	1	1	1	0	0	1	1	4
1461	66	2	0	0	0	1	1	0	0	0	1	1	1	0	0	2	3
1462	68	2	0	1	0	1	1	0	0	0	1	0	0	0	0	2	2
1463	82	1	0	1	1	0	1	1	1	1	1	0	0	0	0	2	4
1465	67	2	0	0	0	#NULL!	1	0	0	0	1	0	0	0	0	3	3
1467	84	1	1	0	1	1	1	2	0	0	0	1	1	1	0	1	4
1468	78	2	0	0	0	1	1	0	0	0	1	1	0	0	0	2	2
1470	65	1	0	0	0	0	1	#NULL!	0	1	0	0	0	0	1	3	4
1471	83	1	1	0	0	1	1	0	1	1	0	0	1	0	0	2	4
1472	75	2	1	0	0	1	3	1	0	0	0	1	0	0	0	2	3
1476	90	2	1	#NULL!	0	#NULL!	1	0	0	#NULL!	1	0	0	0	0	2	3
1477	71	1	0	0	0	1	3	1	1	1	1	0	0	0	0	2	4
1478	68	2	0	0	0	1	1	0	0	0	1	0	0	0	0	2	4
1479	65	1	0	0	0	0	3	1	1	1	1	0	0	0	0	3	4
1485	67	2	0	0	0	0	3	0	0	0	1	0	0	0	0	2	2
1486	66	1	0	0	1	0	3	0	0	1	1	0	0	0	0	1	4
1488	84	1	0	1	0	0	1	0	1	1	1	1	0	0	0	2	4
1489	67	1	0	0	0	#NULL!	1	1	0	1	1	0	0	0	1	1	4
1490	66	2	0	0	0	1	2	0	0	0	0	0	0	0	0	2	4
1494	72	1	0	0	0	0	1	1	1	1	0	0	0	0	0	1	4
1495	66	2	0	0	0	0	1	0	0	0	1	0	0	0	0	2	3
1498	66	1	0	0	0	1	3	1	0	1	0	0	1	0	0	1	4
1500	65	2	0	1	0	0	1	0	0	0	1	1	0	0	0	2	4
1502	66	2	0	1	0	1	1	0	0	0	1	1	0	0	0	1	1
1505	77	2	0	1	0	0	1	1	0	0	1	1	0	0	0	4	1
1506	72	2	0	0	0	0	1	1	0	0	0	0	1	0	0	2	2
1507	71	1	0	0	0	1	1	0	1	0	0	0	0	0	0	1	4
1509	82	1	0	1	1	1	1	#NULL!	#NULL!	#NULL!	1	1	#NULL!	0	0	3	4

1511	66	1	0	0	0	1	1	1	0	1	1	0	0	0	1	1	4
1512	67	1	1	0	0	0	1	0	0	1	1	0	0	0	1	1	4
1513	74	1	0	0	0	1	1	0	1	1	1	1	0	0	0	1	4
1514	70	2	0	0	0	1	3	1	0	0	1	0	1	0	0	2	2
1516	81	1	0	1	1	1	1	2	0	1	0	1	0	0	0	1	4
1520	72	1	0	0	0	1	1	1	0	1	1	1	0	0	1	1	4
1521	70	2	1	0	0	1	3	0	0	0	0	0	0	0	1	2	4
1527	71	2	0	0	0	1	1	1	0	1	0	0	0	0	1	2	4
1529	66	1	0	0	0	0	3	1	0	1	1	1	0	0	1	1	4
1533	71	1	0	0	1	1	1	2	1	1	1	0	0	0	1	3	4
1536	65	1	0	0	0	0	3	1	1	0	1	1	0	0	1	3	4
1538	68	1	0	0	0	1	2	0	1	1	1	1	0	0	0	1	4
1540	69	2	0	0	0	1	1	1	0	0	0	0	0	0	0	3	3
1542	76	2	0	0	1	0	1	1	0	0	0	0	0	0	0	1	2
1547	71	1	0	0	0	#NULL!	1	1	1	1	1	1	0	0	1	2	4
1548	70	2	1	0	0	#NULL!	1	0	0	0	0	0	0	0	0	2	1
1549	85	1	1	0	0	0	1	0	0	1	0	0	1	0	1	4	4
1550	79	2	0	0	0	1	1	2	0	0	0	0	1	0	0	2	1
1551	74	1	1	0	0	0	1	1	1	1	1	1	0	1	0	3	4
1552	70	2	0	#NULL!	0	0	1	#NULL!	#NULL!	#NULL!	1	0	#NULL!	0	0	4	1
1554	77	1	0	0	0	0	1	0	0	1	1	0	0	0	0	3	4
1558	72	2	0	0	1	1	1	1	0	0	1	0	0	0	0	3	4
1566	66	1	0	0	0	0	3	1	1	1	1	0	0	0	1	2	4
1572	74	1	0	0	1	#NULL!	1	0	1	0	0	0	0	0	0	3	4
1573	70	2	0	0	1	#NULL!	1	1	0	0	0	0	0	0	0	4	2
1576	72	1	0	0	0	1	1	1	0	1	1	0	0	0	0	2	4
1577	66	2	0	0	0	1	1	0	0	0	0	0	1	0	0	4	1
1579	65	2	0	0	0	1	3	#NULL!	0	0	1	0	0	0	0	2	3
1580	72	1	1	0	1	0	#NULL!	1	1	1	0	0	0	0	0	2	4
1581	65	2	0	0	1	0	3	#NULL!	0	0	0	0	0	0	1	1	4
1582	81	1	0	0	0	1	1	1	0	1	0	0	1	0	0	3	4
1592	80	1	0	0	1	1	1	0	0	1	0	0	0	0	0	4	4
1593	78	2	1	1	0	#NULL!	1	1	0	0	0	0	1	0	0	3	2
1600	73	1	0	0	0	1	1	0	0	1	0	0	1	0	0	2	4
1601	75	2	0	0	1	1	1	0	0	0	0	0	0	0	0	3	4
1602	65	1	0	0	0	1	1	0	1	0	1	0	0	0	1	1	4
1605	68	2	0	1	0	1	3	0	0	0	0	1	0	0	1	2	4
1606	67	1	0	0	0	1	2	0	0	1	1	1	0	0	0	1	4
1611	75	2	0	1	1	0	1	1	1	#NULL!	1	0	0	0	0	1	4

1612	70	1	0	0	0	0	3	2	1	0	1	1	0	0	0	3	4
1613	70	2	0	0	0	1	1	1	0	0	1	1	0	0	0	2	4
1616	87	2	0	0	0	0	1	#NULL!	0	0	0	1	0	0	0	1	4
1625	67	2	0	1	0	0	2	0	0	0	0	0	1	0	0	3	3
1627	82	1	0	1	0	1	3	2	0	1	1	0	0	0	0	1	4
1633	81	1	0	0	0	0	1	0	1	1	0	0	0	0	1	1	4
1634	73	2	0	0	0	1	1	0	0	0	0	0	1	0	0	2	2
1639	66	1	0	0	0	1	1	1	1	1	0	0	1	0	0	1	4
1642	87	2	1	0	0	0	1	1	0	0	1	0	#NULL!	0	0	4	1
1643	80	1	1	0	0	0	3	0	1	1	0	0	0	0	1	4	4
1646	75	1	0	0	0	1	1	1	0	1	1	1	0	0	0	1	4
1647	72	2	0	0	0	0	1	0	0	0	1	1	0	0	0	1	3
1653	67	2	0	0	0	0	2	0	0	0	0	0	0	0	0	2	3
1655	75	1	0	0	1	1	1	0	0	1	1	1	0	0	0	3	4
1657	69	2	0	1	1	0	1	#NULL!	#NULL!	#NULL!	0	1	#NULL!	0	0	2	2
1658	81	1	1	1	0	#NULL!	1	#NULL!	1	0	0	0	0	0	0	1	4
1659	76	2	0	0	0	1	2	0	0	0	0	0	1	0	0	2	1
1660	71	1	1	1	0	1	3	1	0	1	0	1	1	0	0	4	4
1661	74	2	0	1	1	1	1	1	0	1	0	0	0	0	0	2	2
1665	73	1	0	0	0	1	1	0	0	0	0	0	0	0	1	4	4
1669	72	2	0	0	1	0	1	1	0	0	0	0	0	0	0	1	4
1671	74	2	0	0	1	1	3	#NULL!	0	0	0	0	0	0	0	2	4
1676	65	1	0	0	0	1	1	0	0	1	0	0	0	0	1	3	4
1677	65	2	0	0	0	#NULL!	1	0	0	0	0	1	0	0	1	2	3
1679	73	2	0	1	0	0	3	1	0	0	1	1	0	0	1	2	2
1680	67	1	0	0	0	1	1	0	0	0	1	1	0	0	0	2	4
1684	65	2	0	1	0	0	1	0	0	1	1	0	0	0	1	2	2
1691	84	2	1	1	0	0	1	0	0	0	0	0	1	0	0	4	1
1692	83	2	0	1	0	0	2	0	0	1	1	0	0	0	0	3	1
1693	72	2	0	#NULL!	1	#NULL!	1	#NULL!	#NULL!	#NULL!	0	0	#NULL!	0	0	2	4
1697	65	2	0	1	1	1	1	1	0	1	0	0	0	0	0	3	3
1698	66	2	0	1	0	1	1	0	0	0	0	0	0	0	1	3	4
1699	71	2	0	1	0	1	2	#NULL!	#NULL!	#NULL!	0	0	#NULL!	0	0	3	4
1701	76	1	0	0	0	1	#NULL!	1	0	1	1	0	1	0	0	1	4
1702	76	2	0	0	0	1	1	#NULL!	0	0	0	0	0	0	0	2	2
1704	73	2	0	1	0	1	3	1	0	0	1	0	0	0	0	2	2
1714	82	1	1	0	1	0	1	1	1	0	1	1	0	0	0	3	4
1715	74	2	0	0	1	0	1	1	0	0	0	1	0	0	0	3	4
1721	76	1	0	0	0	1	1	1	0	1	1	0	0	0	0	1	4

1722	66	2	0	0	#NULL!	1	1	#NULL!	0	1	0	0	0	0	1	2	1
1724	72	1	1	0	0	1	1	2	1	1	1	1	0	0	0	1	4
1725	84	2	1	1	1	1	3	1	0	0	0	0	1	0	0	2	3
1726	66	2	0	1	0	1	3	0	0	0	0	0	0	0	1	3	4
1727	75	2	0	1	1	0	1	0	0	0	0	0	0	0	0	3	3
1729	66	1	0	0	1	0	3	0	0	1	1	0	0	0	1	4	4
1730	67	1	0	0	0	1	1	0	1	1	1	0	0	0	1	2	4
1733	65	2	0	0	0	1	1	#NULL!	0	0	1	0	0	0	0	2	2
1736	75	1	0	0	0	1	3	1	0	1	0	0	0	0	0	3	4
1737	68	2	0	0	0	1	3	#NULL!	#NULL!	#NULL!	0	0	#NULL!	0	0	2	4
1741	69	2	0	0	1	0	1	1	0	0	0	0	0	0	0	3	3
1743	72	1	1	0	0	1	1	1	1	0	0	1	0	0	1	1	1
1744	79	1	1	0	1	1	1	1	1	1	0	0	0	0	0	3	4
1747	83	2	0	0	0	1	1	0	0	0	0	0	1	0	0	4	1
1750	73	1	0	0	1	1	3	2	0	1	1	1	0	0	0	3	4
1751	68	2	1	0	1	0	1	0	0	0	0	0	0	0	0	3	1
1755	66	1	0	0	0	0	1	#NULL!	#NULL!	#NULL!	1	1	#NULL!	0	1	4	4
1757	75	2	0	1	0	1	1	#NULL!	0	0	0	1	0	0	0	3	2
1760	70	1	0	0	0	1	3	2	0	0	0	0	1	0	0	3	4
1761	65	2	0	0	1	1	1	1	0	0	0	0	0	0	0	3	2
1762	67	2	0	1	1	1	2	#NULL!	#NULL!	#NULL!	0	0	#NULL!	0	0	3	3
1764	65	1	0	0	0	1	1	0	1	0	1	1	0	0	1	2	4
1766	71	1	1	0	0	1	1	0	0	1	0	0	0	1	0	1	4
1767	71	2	0	0	0	1	1	0	0	0	0	0	0	0	0	4	1
1768	71	1	0	0	0	0	1	1	0	1	1	1	0	0	0	2	4
1770	66	1	1	0	0	1	1	0	1	1	0	0	0	0	0	2	4
1771	66	1	0	0	0	0	1	0	0	1	0	0	0	0	0	1	4
1774	74	2	0	0	0	1	1	0	0	0	0	1	0	0	0	3	3
1776	67	2	0	1	0	1	1	0	0	0	0	0	0	0	1	2	4
1777	80	2	0	1	1	0	1	1	0	0	0	0	0	0	0	2	4
1782	69	1	0	0	1	1	1	2	1	1	0	0	1	0	0	3	4
1783	74	1	0	0	1	1	1	0	0	0	0	0	0	0	0	4	4
1786	72	2	0	0	0	1	1	1	0	0	0	0	0	0	0	3	4
1788	76	1	0	#NULL!	0	#NULL!	#NULL!	0	0	1	0	0	0	0	1	2	4
1790	85	2	1	1	0	1	3	1	1	1	0	0	0	0	0	3	3
1794	70	1	0	0	1	1	1	1	0	1	0	0	0	0	0	1	4
1795	69	2	0	0	0	1	1	1	0	0	1	0	0	0	0	2	3
1796	72	1	0	0	0	1	1	1	0	1	0	0	0	1	0	1	4
1797	71	2	1	0	1	1	1	1	0	0	0	0	0	0	0	2	2

1801	73	1	1	0	1	#NULL!	#NULL!	0	0	1	0	0	1	1	0	3	4
1803	70	2	0	1	0	1	1	0	0	0	1	0	0	0	1	1	2
1804	68	1	0	0	0	1	1	#NULL!	#NULL!	#NULL!	0	0	#NULL!	0	0	3	4
1805	74	1	0	0	0	0	1	1	1	1	0	0	0	0	1	1	4
1809	74	1	0	0	0	1	3	1	1	1	0	1	0	0	0	3	4
1810	72	2	0	0	0	1	1	1	0	0	0	1	0	0	0	3	4
1811	84	2	1	0	1	1	3	2	0	0	0	0	0	1	0	3	4
1814	71	2	0	0	0	1	1	0	0	0	0	0	0	0	0	1	4
1815	74	1	0	0	1	0	1	0	1	1	0	0	0	0	0	2	4
1818	72	1	0	0	1	1	#NULL!	1	1	0	0	0	0	0	1	3	4
1819	71	2	0	0	1	1	3	1	0	0	1	0	0	0	0	2	2
1820	74	1	0	0	0	1	1	0	1	0	1	0	0	0	0	2	4
1824	66	2	0	0	0	1	1	0	0	0	0	0	0	0	0	4	1
1825	76	2	1	1	0	1	2	1	1	0	0	0	0	0	0	2	2
1826	75	1	0	0	0	1	1	#NULL!	1	1	0	0	0	0	0	3	4
1827	73	2	0	0	0	1	1	1	1	0	1	0	0	0	0	2	1
1830	76	1	0	0	0	1	1	2	0	1	0	0	0	0	0	2	4
1832	73	1	1	0	1	1	1	#NULL!	1	1	1	1	0	0	1	3	1
1835	77	1	0	0	0	1	1	1	0	1	0	0	0	0	1	1	4
1836	71	2	0	0	0	1	1	0	0	0	0	1	0	0	1	3	3
1840	76	1	0	0	0	0	1	0	1	1	1	1	0	0	0	2	4
1841	74	2	0	0	0	0	2	0	0	0	0	0	0	0	0	2	2
1843	84	1	1	0	0	1	1	0	0	0	0	0	0	0	0	1	4
1844	78	2	1	0	1	1	1	0	0	0	0	0	0	0	0	3	2
1846	74	2	0	0	0	1	1	1	0	0	1	1	0	0	0	2	3
1848	78	2	1	0	1	1	1	0	0	0	0	0	1	1	0	4	4
1852	68	2	0	0	1	1	1	0	0	0	0	0	0	0	0	2	3
1854	75	1	1	0	0	1	1	0	0	1	1	0	0	1	0	2	4
1855	71	2	0	0	0	1	1	1	0	0	1	0	0	0	0	2	2
1856	73	2	0	#NULL!	0	1	1	0	0	0	1	0	0	0	0	2	1
1857	74	1	0	0	1	1	1	1	0	0	0	0	0	0	1	2	4
1859	65	1	0	0	0	0	1	1	1	1	1	0	0	0	0	2	4
1865	79	2	0	1	1	1	2	1	0	0	0	0	0	0	0	3	3
1867	78	1	1	#NULL!	0	#NULL!	1	0	1	0	0	1	0	0	0	2	4
1868	70	1	0	0	1	1	1	0	0	1	0	0	1	1	0	3	4
1869	67	2	0	0	1	1	1	0	0	1	0	0	0	0	1	3	4
1870	76	2	0	1	1	1	1	1	0	0	0	0	1	1	0	3	2
1875	74	2	0	1	0	1	1	1	0	0	1	0	1	0	0	4	1
1876	76	1	0	1	1	1	1	1	1	1	1	1	0	0	0	2	4

1878	65	2	0	0	0	0	1	0	0	0	1	0	0	0	1	4	4
1880	72	1	0	0	1	1	3	1	1	1	0	0	0	0	0	3	4
1881	69	2	0	0	0	0	1	#NULL!	0	0	0	0	0	0	0	3	3
1882	71	1	0	0	1	1	3	1	1	1	0	0	0	0	1	1	4
1883	70	2	0	0	1	1	1	1	0	0	0	0	0	0	1	3	4
1888	78	2	0	1	0	0	#NULL!	1	0	0	1	1	1	0	0	4	1
1889	73	2	0	0	1	1	1	0	0	0	1	0	0	0	0	2	3
1891	76	1	1	0	1	1	1	#NULL!	0	1	0	0	1	0	0	3	4
1893	68	1	0	0	0	1	1	0	1	0	1	0	0	0	1	2	4
1894	66	1	0	0	0	1	3	1	1	1	0	0	1	1	1	1	4
1896	69	1	0	0	0	#NULL!	1	1	0	1	1	0	0	0	0	2	4
1897	83	2	0	0	0	0	1	#NULL!	0	0	0	0	1	0	0	4	1
1907	70	1	0	0	0	0	1	1	1	1	0	0	0	1	1	2	4
1908	69	2	0	0	0	1	1	1	0	0	1	1	1	0	1	1	4
1909	76	1	1	1	1	1	1	0	0	1	0	0	1	0	0	4	4
1910	79	1	0	0	0	1	3	1	0	0	0	0	0	0	0	1	4
1914	79	2	0	1	0	1	3	1	0	0	1	1	0	0	0	2	4
1915	81	1	0	0	0	1	3	2	0	1	1	0	1	0	0	2	4
1916	74	2	1	0	0	1	3	1	0	0	0	0	0	0	0	3	2
1919	76	2	0	0	1	0	1	1	0	0	0	0	0	0	0	4	3
1922	79	1	0	1	1	1	1	0	0	0	1	1	0	0	0	3	4
1924	72	2	0	0	0	1	1	1	#NULL!	#NULL!	0	0	0	0	0	4	2
1925	69	1	0	0	0	1	1	1	1	1	1	0	0	0	1	1	4
1926	71	2	1	1	0	1	3	1	0	0	#NULL!	#NULL!	1	0	1	2	4
1927	90	2	1	1	1	#NULL!	1	1	0	0	0	0	1	1	0	4	1
1929	67	2	0	0	0	0	1	1	1	0	0	0	0	0	1	2	4
1931	70	2	0	0	0	0	1	#NULL!	0	1	0	0	0	0	1	3	4
1934	79	2	0	#NULL!	0	#NULL!	1	0	0	0	0	0	0	0	0	1	3
1935	82	1	1	0	0	1	1	1	0	1	0	0	1	1	1	2	2
1937	77	2	1	1	1	1	1	0	0	0	1	0	0	0	0	4	1
1944	77	1	0	0	1	1	1	2	0	1	0	0	0	0	0	3	4
1948	72	1	0	0	1	1	1	#NULL!	0	1	1	1	0	0	0	1	4
1949	69	2	0	0	0	1	1	#NULL!	0	0	1	1	0	0	0	2	3
1952	73	2	0	0	0	1	2	1	0	0	0	0	0	0	1	4	1
1955	79	2	0	1	1	1	1	1	0	0	0	0	1	0	0	4	4
1956	74	1	1	0	1	1	3	2	0	1	0	0	0	0	1	3	4
1959	66	2	0	1	0	0	1	1	0	0	0	0	1	0	0	3	4
1961	78	2	1	0	1	1	1	0	0	1	0	0	1	0	0	2	4
1962	74	1	0	0	1	1	1	0	0	1	0	0	0	0	0	3	4

1966	69	1	1	0	0	1	1	2	0	1	0	0	0	0	0	3	4
1972	70	2	0	1	1	1	1	1	0	0	0	0	1	0	0	3	4
1978	73	2	0	1	0	1	1	0	0	0	0	1	0	0	0	2	1
1983	70	2	0	1	0	1	1	#NULL!	1	1	0	0	1	0	1	2	1
1984	70	1	0	0	0	0	1	#NULL!	0	0	1	0	0	0	1	1	4
1985	76	2	0	1	0	1	1	1	0	0	0	0	0	0	0	2	3
1990	65	1	0	0	0	1	1	1	0	1	0	0	1	1	1	1	4
1991	76	2	0	1	1	1	1	2	0	0	0	0	#NULL!	0	1	1	4
1992	83	2	0	1	0	1	1	0	0	0	0	0	#NULL!	0	0	3	4
1993	80	2	1	1	1	0	1	1	0	0	0	0	0	0	0	2	4
1997	85	1	0	0	0	0	1	2	1	1	0	0	1	0	0	1	4
1998	81	2	0	0	0	#NULL!	3	1	0	0	0	0	0	0	0	4	1
2000	77	2	0	0	0	1	1	0	0	0	0	0	0	0	0	4	1
2009	76	1	0	0	0	0	1	1	1	1	0	0	1	0	0	1	4
2014	67	1	0	0	0	1	1	0	0	1	0	0	0	0	1	2	4
2015	69	1	0	0	0	0	1	0	1	1	1	1	0	1	1	1	4
2016	66	2	0	0	0	0	2	0	0	0	1	1	0	0	0	2	3
2018	66	1	0	0	0	0	1	1	1	1	1	1	0	0	0	2	4
2022	73	2	0	1	0	0	1	0	0	0	1	0	0	0	0	2	4
2025	81	2	1	1	0	1	1	0	0	0	0	0	0	0	0	2	2
2027	71	2	0	0	1	1	1	0	0	0	1	0	1	0	0	3	4
2029	65	1	0	0	0	1	1	#NULL!	0	0	0	0	0	0	1	2	4
2032	67	1	0	0	0	1	3	#NULL!	1	1	0	0	1	0	0	1	4
2033	85	2	1	1	0	0	3	1	0	0	0	0	0	0	0	2	2
2036	67	2	0	1	0	1	1	#NULL!	0	0	0	0	0	0	0	2	2
2038	74	2	0	1	0	0	1	1	1	0	1	1	0	0	0	4	1
2039	73	2	0	1	0	0	2	0	0	0	0	1	0	0	0	3	4
2043	74	2	0	0	0	1	2	0	0	0	0	1	0	0	0	2	1
2047	77	1	1	0	1	1	1	1	1	1	0	1	0	0	0	1	4
2048	72	2	1	0	1	1	1	#NULL!	1	0	0	0	1	0	1	2	1
2049	84	1	0	0	0	0	3	0	1	0	1	0	0	0	1	1	4
2050	77	2	0	0	0	0	1	1	0	0	1	0	0	0	0	1	3
2052	78	2	0	0	0	0	3	0	0	0	1	1	0	0	0	1	4
2053	76	2	0	1	1	0	#NULL!	#NULL!	0	0	0	0	0	0	1	1	4
2055	88	2	0	1	0	0	1	2	0	0	0	0	0	1	0	3	2
2057	68	2	0	1	0	1	1	1	1	1	0	0	0	0	1	4	4
2058	67	2	0	1	1	0	1	1	0	0	0	0	0	0	1	3	4
2059	81	2	1	1	0	1	1	0	1	0	1	0	0	0	0	2	4
2065	66	2	0	0	0	1	1	0	1	0	0	0	0	0	1	2	3

2067	70	1	0	0	1	0	1	1	0	1	0	0	0	0	0	3	4
2069	71	1	0	0	0	1	1	1	1	1	1	0	0	0	0	4	1
2070	70	2	0	0	0	1	1	1	0	0	0	0	0	0	0	2	4
2071	71	2	0	1	0	#NULL!	1	1	0	0	0	0	0	0	0	3	3
2073	70	2	0	0	1	1	1	1	0	0	0	0	0	0	0	2	3
2074	68	1	0	#NULL!	0	#NULL!	1	#NULL!	#NULL!	#NULL!	0	0	#NULL!	0	0	1	4
2075	66	1	0	1	0	1	1	2	0	1	0	1	0	0	1	1	4
2078	81	2	1	0	1	#NULL!	3	1	0	0	0	0	1	0	0	3	2
2085	69	1	0	0	0	0	3	0	0	1	0	0	0	0	1	1	4
2086	66	2	0	0	0	0	1	0	0	0	0	0	0	0	1	4	1
2093	65	2	0	0	0	1	1	0	0	0	1	0	0	0	0	2	1
2095	67	1	0	0	0	0	1	#NULL!	1	1	1	0	0	0	0	1	4
2102	70	2	0	0	0	1	1	0	0	0	0	0	0	0	0	4	1
2104	73	2	0	0	0	0	1	1	0	0	0	0	0	0	0	4	2
2109	83	2	0	1	0	0	1	0	0	1	1	1	0	1	1	3	4
2113	81	2	0	1	0	1	1	0	0	0	0	0	0	0	0	1	3
2114	86	2	0	1	#NULL!	1	1	0	0	0	0	0	0	0	0	1	4
2115	70	1	0	0	0	#NULL!	1	0	0	1	0	0	0	1	0	2	4
2116	66	2	0	0	0	#NULL!	1	1	0	0	0	0	0	0	0	2	2
2118	74	2	0	0	0	0	1	1	0	0	0	0	1	0	0	2	2
2119	68	1	0	0	1	1	3	1	0	1	0	0	0	0	1	3	4
2121	71	1	0	0	0	0	3	2	1	1	0	0	0	0	0	1	4
2123	73	1	0	0	1	1	3	0	0	0	0	0	0	0	0	3	4
2124	69	2	0	0	1	1	1	1	0	0	0	0	0	0	0	3	4
2125	69	2	0	1	0	1	1	1	0	0	0	0	0	0	0	2	4
2129	70	2	0	1	1	1	1	0	0	1	1	1	1	0	1	4	3
2130	79	2	1	1	0	1	1	2	0	1	0	0	1	0	1	2	1
2132	73	2	0	0	0	1	1	1	0	0	0	0	0	0	1	2	4
2133	73	1	0	0	1	1	3	1	0	1	0	0	0	0	1	3	4
2135	66	1	0	0	0	1	1	1	0	1	1	1	0	0	0	2	4
2138	71	2	1	1	1	1	1	1	0	0	0	0	1	0	0	4	1
2140	81	2	0	0	0	1	1	1	1	0	0	0	0	0	1	1	4
2141	75	1	0	0	1	1	1	1	1	1	1	0	1	1	0	1	4
2142	68	2	0	0	1	1	1	#NULL!	0	1	1	0	0	0	0	3	4
2144	67	1	0	0	0	1	1	0	1	1	1	0	0	0	0	2	4
2146	83	1	1	0	1	1	1	1	0	1	0	0	0	0	0	3	4
2147	79	2	0	0	1	1	1	1	0	0	0	0	0	1	0	3	4
2149	65	2	0	0	0	1	1	1	0	0	0	0	0	0	1	2	4
2154	71	1	0	0	0	1	1	1	0	0	0	1	0	0	1	1	4

2155	70	2	0	0	0	#NULL!	3	#NULL!	0	0	0	0	0	0	0	2	4
2157	75	1	0	0	0	1	1	2	1	1	0	0	0	0	0	2	4
2158	70	2	0	0	#NULL!	1	1	0	0	0	0	0	0	0	0	4	4
2163	73	2	0	1	0	1	1	#NULL!	0	0	1	1	1	0	0	1	3
2164	75	2	0	0	0	1	1	1	0	0	0	0	0	0	0	4	1
2165	73	1	0	0	0	1	1	1	1	1	1	0	0	0	0	3	4
2166	70	1	1	0	0	1	1	2	1	0	0	0	0	0	1	1	4
2167	70	2	0	0	0	1	1	#NULL!	0	0	0	0	0	0	0	2	2
2168	70	2	0	1	0	0	1	0	0	1	0	0	0	0	1	3	4
2169	65	1	1	1	0	1	1	1	1	0	0	1	0	0	0	2	4
2173	67	2	0	0	1	1	3	1	0	0	0	0	0	0	0	1	4
2175	73	2	0	1	1	#NULL!	1	0	0	0	0	1	1	0	0	4	4
2180	66	1	0	1	0	0	1	1	1	1	1	0	0	0	0	2	4
2192	75	2	0	0	1	1	1	1	0	1	0	0	0	0	0	3	4
2193	75	1	0	0	1	1	1	0	1	1	0	0	0	0	1	1	4
2194	66	1	0	0	1	1	1	0	0	1	0	0	0	0	0	3	4
2195	66	2	0	0	1	1	#NULL!	2	0	0	0	0	0	0	0	3	1
2199	72	1	0	0	1	1	1	1	0	0	0	0	0	0	0	4	4
2200	71	2	0	0	0	#NULL!	1	1	0	0	0	0	0	0	1	2	2
2202	66	1	0	0	0	1	1	1	1	1	0	0	0	0	0	1	4
2205	76	2	0	1	0	1	1	0	0	0	0	0	0	0	0	2	2
2207	65	2	0	0	0	1	2	#NULL!	0	0	1	1	0	0	0	2	2
2208	78	2	0	1	0	#NULL!	3	1	0	0	0	1	0	0	0	3	4
2210	74	1	0	0	1	1	1	0	1	1	1	0	0	0	0	1	4
2211	71	2	0	0	1	1	1	1	0	0	1	0	0	0	0	2	3
2212	79	1	0	0	0	1	#NULL!	0	0	1	1	0	0	#NULL!	0	2	4
2216	79	2	1	0	1	0	1	#NULL!	0	0	1	1	0	0	0	3	4
2222	73	2	1	0	1	1	3	0	0	0	0	0	0	0	0	3	1
2226	74	1	1	0	1	0	1	0	1	0	1	0	0	0	0	3	1
2227	68	2	0	0	1	0	1	0	0	0	1	0	0	0	0	3	4
2228	76	1	1	1	1	1	3	0	1	#NULL!	1	1	0	0	0	1	4
2230	82	2	0	0	0	0	#NULL!	1	1	0	0	0	0	0	1	1	4
2231	75	2	0	1	1	0	1	2	0	0	1	0	0	0	0	3	3
2232	65	1	0	0	0	0	1	1	0	1	0	0	0	0	0	1	4
2234	80	2	0	1	1	0	3	0	0	0	0	0	0	0	0	1	4
2246	70	1	0	0	0	1	3	0	1	1	0	0	0	0	0	1	4
2250	67	1	0	0	1	1	1	0	0	0	0	0	0	0	0	2	4
2254	65	2	0	0	1	1	1	1	0	0	1	0	0	0	0	3	3
2256	68	2	1	1	0	1	3	2	0	0	0	0	1	0	0	2	2

2257	73	1	0	0	1	1	1	0	1	1	0	1	0	0	1	2	4
2258	77	1	0	1	0	0	1	0	0	1	0	0	0	0	1	3	4
2260	76	1	0	#NULL!	0	#NULL!	#NULL!	#NULL!	#NULL!	#NULL!	0	0	#NULL!	0	0	3	4
2261	71	2	0	0	0	0	2	0	0	0	1	0	0	0	0	2	1
2262	69	2	0	1	1	1	1	0	0	0	1	0	0	0	0	3	3
2268	77	1	0	1	0	#NULL!	3	1	0	1	1	1	0	0	0	1	4
2269	71	2	0	1	1	#NULL!	1	2	0	0	0	0	0	0	0	4	1
2272	66	1	0	0	0	1	3	2	0	1	1	1	0	0	1	2	4
2282	69	1	0	1	1	1	1	1	0	1	0	0	0	0	0	2	4
2285	77	1	1	0	1	1	3	1	1	0	0	0	0	0	0	4	4
2289	73	1	0	0	1	1	3	2	0	1	1	0	0	1	1	3	4
2290	71	2	0	0	1	1	1	1	1	0	0	0	0	0	0	3	4
2294	83	2	1	1	1	1	1	0	0	1	0	0	1	0	0	2	4
2299	76	1	0	0	0	0	3	2	0	0	0	1	0	0	0	3	4
2300	73	2	0	0	0	0	1	0	0	0	0	1	0	0	0	2	4
2301	76	1	1	0	0	1	2	0	0	1	0	0	0	1	0	4	4
2302	67	1	0	0	1	1	1	1	1	0	1	0	0	0	0	2	4
2308	69	1	0	0	0	1	2	2	0	1	0	1	0	0	0	2	4
2312	72	1	1	0	1	0	1	1	1	1	0	0	0	0	1	3	4
2314	71	1	0	1	0	1	1	0	0	0	0	0	1	0	0	3	4
2317	69	1	0	0	0	0	1	1	1	1	0	0	0	0	1	1	4
2318	66	2	0	0	0	1	1	0	0	0	0	0	0	0	0	2	2
2320	82	2	0	0	1	1	1	1	0	0	0	0	0	0	0	4	1
2322	69	2	0	1	1	1	1	0	0	0	0	0	0	0	0	3	3
2323	81	1	0	0	1	1	1	#NULL!	0	0	0	0	0	#NULL!	1	1	4
2327	69	2	0	0	1	1	1	0	0	0	0	0	0	0	0	3	4
2328	66	2	0	1	0	1	1	0	0	0	0	0	0	0	0	2	3
2329	68	1	0	0	1	1	1	0	0	1	0	0	1	1	1	3	4
2332	66	2	0	0	0	1	1	0	0	1	0	0	0	0	0	4	2
2336	89	2	1	1	1	1	1	1	0	0	0	0	0	0	0	2	3
2337	75	1	1	0	1	0	#NULL!	2	0	1	1	1	1	0	0	2	1
2338	72	2	0	0	0	1	1	2	0	0	0	0	0	0	0	4	2
2346	74	2	0	0	0	0	2	0	0	0	0	1	0	0	0	2	4
2347	69	2	0	0	0	1	1	0	0	0	1	1	0	0	0	4	1
2348	69	1	0	0	0	1	3	1	1	1	1	1	0	0	1	1	4
2349	68	1	0	0	0	0	1	0	0	0	0	0	0	0	1	1	4
2350	68	2	0	1	0	0	1	0	0	0	0	0	0	0	0	1	2
2351	70	1	0	0	0	0	1	0	1	1	0	0	1	0	1	2	4
2352	65	2	0	0	0	0	1	0	1	0	0	0	0	0	1	3	1

2358	65	1	0	0	0	1	3	2	0	1	1	0	0	0	1	3	4
2359	69	2	0	1	0	1	3	#NULL!	0	0	0	0	0	0	0	4	1
2360	71	2	0	1	0	0	1	#NULL!	#NULL!	#NULL!	0	0	#NULL!	0	0	4	1
2363	65	2	0	0	1	1	1	0	0	1	0	0	0	0	0	2	2
2365	68	1	0	0	1	0	1	0	1	1	0	0	0	0	1	2	4
2369	90	1	0	0	1	1	1	1	1	1	1	0	0	0	0	1	4
2371	65	1	0	1	0	1	1	0	1	1	0	0	0	0	0	1	4
2372	74	1	0	0	1	1	1	1	1	0	1	0	#NULL!	1	0	1	4
2373	69	2	0	0	1	1	1	1	1	0	1	0	0	0	0	1	3
2374	66	1	0	0	0	1	3	0	1	0	1	0	0	0	0	2	4
2376	65	2	1	0	0	0	3	0	0	0	1	0	0	0	0	1	4
2383	71	1	0	0	0	1	1	0	0	1	0	0	0	0	1	1	4
2384	74	2	0	1	0	1	3	1	1	1	0	1	0	0	0	1	4
2385	66	1	1	0	0	1	1	0	0	1	0	0	0	0	1	2	4
2386	67	1	0	0	1	0	1	0	1	1	1	1	0	0	0	1	4
2387	66	2	0	0	0	1	1	0	0	0	1	0	0	0	0	2	4
2394	86	1	0	0	0	0	1	#NULL!	1	1	0	0	0	0	0	1	4
2395	79	2	0	0	0	0	3	1	0	0	1	1	1	0	0	2	2
2398	70	1	0	0	0	0	1	1	1	1	0	0	0	0	0	2	4
2400	76	1	0	0	1	1	2	0	1	1	0	0	1	1	0	2	4
2401	73	2	0	0	1	1	1	1	0	0	0	0	1	0	0	2	4
2402	73	1	0	0	1	1	1	1	1	0	1	0	0	0	0	2	4
2403	71	2	0	0	0	1	1	1	0	0	1	0	0	0	0	2	4
2404	77	1	0	0	1	1	1	2	0	1	0	0	0	0	0	2	4
2405	74	2	1	0	1	1	3	2	0	0	0	0	0	0	0	2	3
2407	67	2	0	0	0	0	3	2	0	1	0	0	0	0	0	2	4
2419	70	1	0	0	1	0	1	1	1	1	0	0	0	1	0	1	4
2420	77	2	0	#NULL!	1	#NULL!	#NULL!	0	0	0	0	0	0	0	0	3	2
2421	74	1	0	1	0	1	1	0	1	1	1	1	0	0	0	1	4
2423	67	2	0	0	0	1	3	0	0	0	1	0	0	0	0	2	3
2425	67	1	0	0	0	0	#NULL!	1	0	1	0	1	0	0	0	1	4
2426	77	1	0	0	1	0	1	1	1	0	0	0	#NULL!	0	0	3	4
2427	71	2	0	0	1	0	2	#NULL!	0	0	0	0	0	0	0	4	1
2430	65	1	0	0	0	1	1	0	0	1	0	0	0	0	1	2	4
2432	76	2	1	0	0	1	3	1	0	0	0	0	1	0	0	4	3
2435	68	1	0	0	0	1	1	1	1	0	0	0	0	1	1	3	4
2437	69	2	0	0	0	#NULL!	1	0	0	0	0	0	0	0	0	2	4
2439	74	1	0	0	1	1	3	1	1	1	0	0	0	0	0	3	4
2441	69	1	0	0	0	1	1	0	0	0	1	1	0	0	0	1	4

2442	75	1	0	0	0	1	1	1	0	1	1	0	0	0	1	2	4
2443	70	2	0	0	0	1	1	1	0	0	1	0	0	0	0	2	1
2444	74	2	0	0	0	0	1	0	0	0	0	0	0	0	0	1	4
2448	74	1	1	0	0	0	1	0	0	1	1	0	0	1	0	1	4
2449	73	2	0	0	0	0	1	0	0	0	1	0	0	0	0	1	1
2451	72	2	0	0	0	1	3	0	0	0	1	0	0	0	0	3	3
2454	76	2	1	0	1	0	1	1	0	1	1	0	0	0	0	1	3
2455	72	1	0	0	0	0	3	0	1	0	1	1	0	0	1	2	4
2456	76	1	0	0	0	0	1	0	0	0	1	0	0	0	0	1	4
2457	71	2	0	0	0	0	1	0	0	0	0	0	0	0	0	4	1
2458	73	1	0	1	0	1	1	0	0	1	0	0	1	0	0	1	4
2461	75	2	0	0	1	1	1	0	0	0	0	0	0	0	0	2	4
2464	73	1	0	0	0	0	1	1	1	1	0	0	0	0	1	1	4
2466	72	1	0	0	0	0	1	0	1	0	1	1	0	0	0	1	4
2469	76	2	0	0	0	0	1	0	0	0	0	0	1	0	0	2	3
2471	73	2	0	0	0	0	1	0	1	0	0	0	1	0	0	2	2
2472	70	1	0	0	0	1	1	0	0	1	1	1	0	1	0	2	4
2473	65	2	1	0	1	0	2	0	0	0	1	0	0	0	0	3	2
2474	70	1	0	0	0	0	1	#NULL!	1	1	1	1	0	0	1	1	4
2476	70	1	0	0	0	1	3	1	0	0	0	0	0	0	1	1	4
2478	74	2	0	1	0	1	1	1	0	0	0	0	0	0	0	2	2
2479	75	1	0	0	1	1	1	2	1	1	1	0	0	0	0	3	4
2480	71	2	0	0	1	1	1	2	0	0	0	0	0	0	0	1	4
2481	77	2	0	1	1	0	1	1	0	0	0	0	0	0	0	1	2
2483	69	2	0	0	0	1	1	0	0	0	0	0	0	0	0	4	1
2484	72	2	0	0	0	1	1	0	0	0	1	0	0	0	0	2	2
2486	72	1	0	0	0	0	1	0	0	1	0	0	0	0	0	2	4
2487	70	2	0	0	0	#NULL!	1	0	0	0	0	0	0	0	0	2	3
2488	68	1	0	0	0	0	1	1	1	1	1	0	0	0	1	1	4
2489	79	1	0	1	0	1	1	2	0	0	1	0	0	0	1	1	4
2490	70	1	0	0	0	1	1	2	1	1	1	0	#NULL!	0	0	1	4
2494	70	1	0	0	0	0	1	0	0	1	0	0	0	0	0	2	4
2495	68	2	0	1	0	1	1	0	0	0	1	0	#NULL!	0	0	2	4
2501	76	1	0	0	0	1	1	2	1	1	0	0	0	0	0	2	4
2504	75	2	1	0	1	0	1	0	0	0	1	1	0	0	0	4	1
2505	73	1	0	0	0	1	1	1	0	1	0	0	1	0	0	2	4
2506	70	2	0	0	0	1	1	#NULL!	0	0	0	0	0	0	0	2	2
2508	72	1	0	0	0	1	1	0	1	1	0	1	0	0	0	2	4
2509	66	2	0	0	0	1	1	0	0	0	1	1	0	0	0	2	1

2511	72	2	0	0	0	0	1	0	1	0	0	0	0	0	0	2	3
2512	72	1	0	0	0	0	3	#NULL!	#NULL!	#NULL!	1	0	#NULL!	0	0	1	4
2513	65	1	0	0	0	0	3	1	1	1	1	0	0	0	1	2	4
2514	65	1	0	0	0	0	3	1	0	1	0	1	0	0	1	1	4
2515	71	1	1	0	0	1	3	2	1	1	0	0	0	0	1	1	4
2516	70	2	0	0	0	0	3	1	0	0	1	1	0	0	0	2	4
2518	66	2	0	1	0	0	3	2	0	0	1	0	0	0	0	2	4
2519	81	1	0	0	0	0	1	1	1	1	1	0	0	0	0	1	4
2520	75	2	0	0	1	#NULL!	1	2	1	0	1	0	0	0	0	2	3
2521	71	2	1	0	1	1	1	1	0	0	1	0	0	0	1	3	4
2525	70	2	0	0	0	1	1	0	0	0	0	0	0	0	0	3	4
2527	80	2	1	0	0	0	1	0	0	0	0	0	1	0	0	2	2
2529	69	1	1	0	0	1	1	2	1	1	1	0	0	1	1	2	4
2530	69	2	1	0	1	1	1	0	0	0	1	0	1	0	1	4	2
2533	72	2	0	0	1	1	1	1	0	0	1	1	#NULL!	0	0	3	2
2534	74	2	0	1	1	1	1	#NULL!	1	0	1	1	0	0	0	3	1
2536	73	2	0	1	0	1	3	1	0	0	0	0	0	0	0	1	4
2538	77	2	0	0	1	1	1	0	0	0	0	0	0	0	0	3	3
2540	67	2	0	1	0	1	1	2	0	0	0	0	0	0	0	2	2
2542	70	2	0	0	1	1	1	1	0	0	0	0	0	0	0	3	2
2543	72	1	0	0	1	0	1	0	1	0	1	0	0	0	0	3	4
2545	72	1	0	0	1	1	3	2	1	1	1	1	0	0	0	1	4
2546	68	2	0	0	0	1	3	0	0	0	0	0	0	0	0	2	4
2549	68	2	0	0	0	1	1	1	0	0	1	0	0	0	0	4	1
2551	75	1	0	0	0	0	1	1	0	1	0	0	0	0	0	1	4
2552	73	2	0	0	0	0	2	1	0	0	0	0	0	0	0	2	1
2553	73	2	0	0	1	1	1	#NULL!	0	0	1	0	#NULL!	0	0	4	1
2554	73	1	1	0	1	0	1	0	0	0	0	0	0	0	0	3	4
2558	77	2	0	1	#NULL!	0	1	1	0	0	0	0	0	0	0	3	3
2559	67	2	0	0	0	0	1	0	0	0	0	0	1	0	0	3	2
2562	78	2	0	0	1	1	1	0	0	1	1	1	0	0	0	3	4
2564	70	2	0	0	0	1	1	0	1	0	0	0	0	0	0	1	4
2566	72	2	0	0	1	1	1	0	0	0	0	0	0	0	0	3	3
2567	70	1	0	0	0	0	1	1	1	1	0	0	0	0	1	1	4
2570	74	1	0	0	0	0	3	0	1	1	1	1	0	1	0	1	4
2571	70	2	0	0	0	0	2	0	0	0	1	1	0	0	1	1	3
2573	84	2	0	0	0	#NULL!	2	0	0	0	0	0	0	0	0	2	1
2574	76	1	0	0	1	0	1	1	1	1	#NULL!	#NULL!	0	0	1	3	4
2575	70	2	0	#NULL!	0	1	1	#NULL!	#NULL!	#NULL!	1	0	#NULL!	0	0	2	2

2579	65	2	0	1	0	0	1	0	0	0	1	1	0	0	0	2	3
2580	79	2	0	1	0	1	1	#NULL!	0	#NULL!	0	0	0	0	0	3	3
2581	77	1	0	0	0	1	1	0	0	1	0	1	0	1	0	2	4
2582	73	2	0	0	0	1	1	0	0	1	0	0	1	0	0	2	4
2583	69	1	0	0	0	1	1	1	0	1	0	0	0	0	1	3	4
2584	68	2	0	0	0	1	1	1	0	1	0	0	0	0	0	2	4
2586	68	1	0	0	0	0	1	1	1	1	0	0	0	0	0	1	4
2588	72	2	0	1	0	1	1	0	0	1	0	1	0	0	1	1	4
2589	76	1	0	0	0	1	1	1	1	1	1	1	0	0	1	3	4
2590	75	2	0	0	1	1	1	2	0	0	1	1	0	0	0	4	1
2591	66	2	0	0	0	1	1	#NULL!	0	0	0	0	0	0	0	2	4
2592	69	1	0	0	0	#NULL!	1	0	0	1	0	0	0	0	0	1	4
2593	67	2	0	0	0	0	1	1	0	0	0	0	0	0	0	2	2
2594	66	2	0	0	0	0	1	0	0	0	0	0	0	0	0	2	2
2596	66	1	0	0	0	1	1	0	0	1	0	0	0	0	1	2	4
2597	66	2	0	0	0	1	1	0	0	0	1	0	0	0	0	4	2
2598	84	1	1	0	0	1	1	0	1	0	1	0	1	1	0	1	4
2599	76	2	0	0	0	1	3	1	0	0	0	0	1	0	0	1	2
2601	65	2	0	0	0	1	1	0	0	0	1	1	0	0	0	2	2
2602	69	2	0	1	1	1	1	0	0	1	0	0	1	0	0	3	4
2603	86	2	1	1	0	0	1	0	0	0	1	1	1	0	0	1	2
2604	89	2	1	1	0	0	1	#NULL!	0	0	1	0	0	0	0	2	2
2606	77	1	0	0	0	1	1	2	0	1	1	1	0	1	1	4	4
2607	71	2	0	1	1	1	1	1	1	0	0	0	0	0	0	3	4
2610	75	1	0	0	1	0	1	1	1	1	0	0	0	0	1	1	4
2613	76	2	0	1	1	0	1	1	0	0	0	0	1	0	0	3	4
2615	73	1	0	0	0	#NULL!	1	0	0	0	0	0	0	1	0	1	4
2616	69	2	0	0	0	#NULL!	1	0	0	0	0	1	0	0	0	3	2
2617	67	1	0	0	0	1	1	1	1	0	0	0	0	0	0	3	4
2618	69	1	0	0	0	#NULL!	1	1	1	0	0	0	0	0	0	1	4
2620	78	1	0	0	0	1	1	1	0	0	0	0	0	0	0	2	4
2621	68	2	0	0	0	0	1	0	0	0	0	0	0	0	0	2	4
2622	83	2	1	1	0	1	3	1	0	0	0	0	0	1	0	3	1
2623	68	2	0	0	0	1	3	1	0	0	0	0	0	0	0	3	4
2624	72	1	0	0	1	1	1	0	1	1	1	1	0	0	0	3	4
2625	67	1	0	0	0	1	1	1	0	1	0	1	0	1	0	1	4
2630	77	1	0	1	0	1	1	0	0	0	0	0	0	0	0	1	4
2631	68	1	0	0	1	1	1	1	0	0	0	0	1	1	1	1	4
2632	65	2	1	0	0	1	1	0	0	0	0	1	1	0	0	4	1

2633	71	2	0	1	0	1	1	0	0	1	0	1	0	0	0	3	4
2634	70	2	0	0	0	1	1	0	0	0	0	0	0	0	0	2	2
2638	80	2	0	0	0	0	2	2	0	0	0	0	1	0	0	1	4
2639	79	1	0	0	0	0	1	0	0	0	0	0	0	0	0	1	4
2640	67	1	0	0	0	0	1	1	0	1	1	0	0	0	0	1	4
2641	70	1	0	0	0	1	1	0	0	1	0	0	0	0	0	2	4
2642	81	1	1	0	0	1	1	1	0	1	1	0	1	0	0	2	4
2643	74	1	0	0	0	1	1	1	1	1	1	0	0	0	1	1	4
2644	73	2	0	0	0	#NULL!	1	1	0	0	1	1	1	0	0	3	2
2654	76	2	0	0	0	#NULL!	1	0	0	0	0	0	0	0	0	1	4
2656	66	2	0	0	0	0	1	1	0	0	0	0	0	0	0	2	4
2657	74	1	0	0	0	1	1	0	1	1	0	1	0	0	1	1	4
2658	80	1	0	0	0	0	2	0	0	1	0	0	1	0	0	2	4
2659	79	2	0	1	0	1	1	1	0	0	0	0	0	0	0	2	1
2660	68	1	0	0	0	#NULL!	1	0	0	1	0	0	0	0	0	1	4
2672	74	1	0	0	0	1	1	0	0	1	0	0	0	0	0	2	4
2673	71	2	0	0	0	1	2	0	0	0	1	0	0	0	0	3	3
2674	69	2	0	#NULL!	1	1	1	#NULL!	#NULL!	#NULL!	0	1	#NULL!	0	0	2	1
2676	78	2	1	0	1	1	3	0	0	0	0	1	1	0	0	3	4
2679	67	1	0	0	0	1	1	1	0	1	0	0	0	0	1	1	4
2682	73	2	0	0	0	0	1	0	0	0	1	0	0	0	0	1	4
2684	66	2	1	0	0	1	1	1	0	0	0	0	0	0	1	3	4
2686	67	2	0	1	0	1	1	0	0	0	0	0	0	0	1	2	2
2688	77	2	1	1	1	1	1	1	1	1	0	0	0	0	1	1	4
2689	68	2	0	1	0	1	1	1	0	0	0	0	0	0	1	2	4
2691	70	2	0	1	0	0	1	0	0	0	0	0	0	0	0	2	4
2692	68	1	0	0	1	1	1	0	1	1	0	0	1	0	0	3	4
2694	71	2	0	0	1	1	1	0	0	0	0	0	1	0	0	3	2
2695	79	1	0	0	1	1	1	1	0	1	0	0	0	1	1	3	4
2696	71	2	0	0	0	1	1	1	0	0	0	0	0	0	1	3	4
2698	70	2	0	0	0	0	3	2	0	0	0	1	0	0	0	3	4
2700	65	2	0	1	0	1	1	1	0	1	1	0	0	0	0	2	3
2701	68	1	0	0	0	0	3	1	1	1	1	0	0	0	0	1	4
2702	66	1	0	0	0	1	1	1	0	1	0	0	0	0	0	3	4
2707	78	2	0	1	1	1	1	1	0	0	0	0	0	0	1	3	4
2708	69	1	0	0	0	1	1	0	0	1	0	0	0	0	1	2	4
2709	67	2	0	0	0	1	1	0	0	0	0	0	0	0	1	3	4
2716	84	1	0	0	1	0	1	0	1	1	0	0	0	0	0	2	3
2719	68	2	0	1	0	0	1	2	0	0	1	0	0	0	1	3	4

2721	72	2	0	0	0	0	1	1	0	0	0	1	0	0	0	4	4
2726	78	1	1	0	1	0	1	1	0	0	0	0	1	0	0	4	1
2727	76	2	1	0	1	0	1	1	0	0	0	0	0	0	0	2	2
2733	78	2	1	0	0	1	1	1	0	0	1	0	1	0	0	1	3
2743	75	1	0	0	0	1	1	0	0	1	1	1	0	0	1	3	4
2744	69	2	0	0	0	1	2	1	0	0	0	0	0	0	0	3	4
2745	67	2	0	1	0	0	1	0	0	0	1	1	0	0	0	1	4
2747	65	1	0	0	0	0	1	0	0	0	0	1	1	0	1	2	4
2750	79	2	1	0	0	#NULL!	3	1	0	0	1	1	0	0	1	1	1
2751	72	1	0	0	0	0	1	2	0	1	0	0	0	0	1	1	4
2752	69	2	0	0	0	0	1	1	0	0	0	1	0	0	0	2	1
2753	73	1	0	0	0	0	1	1	0	1	1	0	0	0	1	1	4
2754	71	2	0	0	0	0	1	0	0	0	0	0	1	0	1	1	4
2759	75	2	0	0	0	1	2	1	0	0	0	0	1	0	0	1	4
2765	78	1	0	0	0	0	1	2	0	1	1	0	0	0	1	2	4
2766	74	2	0	0	0	0	1	#NULL!	#NULL!	#NULL!	1	0	#NULL!	0	1	2	3
2770	86	2	0	1	0	#NULL!	1	#NULL!	0	0	0	0	0	0	0	3	1
2771	83	2	0	1	0	1	1	1	0	0	0	0	0	0	0	4	1
2773	88	2	1	1	1	0	#NULL!	#NULL!	0	#NULL!	0	0	0	0	0	3	2
2774	67	1	0	0	1	1	1	0	0	1	0	0	0	0	0	1	4
2776	75	2	0	1	0	0	1	0	0	0	1	1	0	0	0	3	4
2778	80	1	1	0	0	1	1	2	0	1	0	0	0	1	0	1	4
2781	69	1	1	0	1	1	1	#NULL!	0	0	0	1	0	0	0	2	4
2782	70	1	1	1	0	1	1	2	1	1	0	0	0	0	0	1	4
2783	66	1	0	0	0	0	1	0	1	0	0	0	0	1	0	1	4
2784	65	2	0	0	0	0	1	0	1	0	0	0	0	0	0	2	4
2787	84	2	1	#NULL!	#NULL!	0	#NULL!	#NULL!	0	0	0	0	1	0	0	4	2
2790	77	1	1	0	1	1	1	#NULL!	#NULL!	#NULL!	0	0	#NULL!	1	0	3	4
2791	71	2	0	0	1	1	1	1	0	#NULL!	0	0	0	0	0	3	2
2792	84	1	1	0	0	0	3	1	1	1	0	0	0	0	0	1	4
2793	77	2	0	0	0	0	1	0	0	0	0	0	0	0	0	1	4
2797	65	2	0	0	0	1	1	0	1	0	0	1	0	0	1	2	4
2806	92	2	0	1	1	0	1	1	0	0	1	1	0	0	0	4	1
2810	70	1	0	0	0	0	2	1	0	1	1	1	1	0	1	1	4
2819	84	2	0	0	1	0	1	2	0	0	0	0	0	0	0	2	3
2820	77	2	0	1	1	1	3	1	0	0	0	1	1	0	0	3	3
2822	76	1	0	0	0	1	3	1	0	1	1	1	0	0	1	3	4
2823	73	2	0	0	0	1	3	#NULL!	0	0	0	0	0	0	1	3	4
2825	67	2	0	0	0	0	1	0	0	0	0	0	1	0	1	3	4

2827	66	1	0	0	0	0	1	0	0	1	0	0	0	0	1	1	4
2830	76	2	0	0	0	0	1	1	0	0	0	0	0	0	1	1	4
2831	69	2	0	1	0	1	1	0	0	0	#NULL!	#NULL!	0	0	1	3	4
2833	70	1	0	1	1	1	3	2	1	1	0	0	0	0	1	3	4
2838	73	2	0	0	0	1	1	1	0	0	0	0	0	0	0	2	3
2839	73	1	0	0	0	1	3	2	1	1	1	1	0	0	0	1	4
2842	69	2	0	0	0	1	1	1	0	0	1	0	0	0	0	2	3
2845	71	2	0	1	0	1	1	0	0	0	0	0	0	0	1	2	4
2848	69	1	0	0	0	0	1	0	0	0	0	0	0	0	1	2	4
2850	67	2	0	1	0	0	1	0	0	0	1	0	1	0	0	4	1
2853	74	2	0	1	0	1	1	1	0	1	0	0	1	0	0	4	1
2854	75	1	0	1	0	0	3	2	0	1	0	0	1	1	0	1	4
2858	65	2	1	1	0	0	3	2	0	1	0	0	0	0	0	4	4
2861	79	1	0	0	0	0	3	1	1	1	0	0	0	0	0	1	4
2862	80	2	1	0	0	1	1	0	0	0	0	0	0	0	0	1	2
2869	67	2	0	0	0	1	1	#NULL!	#NULL!	#NULL!	0	0	#NULL!	0	0	2	2
2870	70	2	0	0	0	1	1	0	0	0	1	0	1	0	0	2	4
2871	69	1	0	0	0	1	1	1	0	0	0	0	0	0	0	2	4
2873	66	1	0	0	0	0	1	0	0	0	0	1	0	0	1	1	4
2876	81	1	0	0	0	0	1	2	1	0	1	0	0	0	1	1	4
2877	76	2	0	0	0	0	1	0	0	0	1	0	0	0	1	4	1
2880	89	1	1	0	0	0	1	2	0	1	0	0	0	1	0	1	4
2881	87	2	0	0	0	0	1	2	0	0	0	0	0	0	0	4	1
2882	65	1	0	1	0	1	1	0	0	1	0	0	1	0	0	2	4
2884	78	1	0	0	0	1	#NULL!	1	0	1	0	0	1	0	0	2	4
2885	74	2	0	0	1	1	1	1	0	0	0	0	0	0	0	1	4
2887	67	1	0	0	0	0	1	0	1	0	1	0	0	0	0	1	4
2890	80	2	1	1	0	1	3	0	0	1	0	0	1	0	0	2	2
2893	70	1	0	0	0	1	1	1	1	1	1	1	0	0	0	2	4
2895	81	1	0	0	0	1	1	1	0	1	0	1	1	1	0	3	4
2896	77	2	0	0	1	1	1	0	0	0	0	0	0	0	1	2	3
2903	72	2	0	0	0	0	1	1	0	0	1	0	0	0	1	1	4
2906	73	1	0	1	1	1	1	1	0	1	0	0	0	0	0	4	1
2908	66	1	0	0	0	0	1	0	0	0	0	0	0	0	0	4	1
2912	69	1	0	0	0	1	1	1	1	1	1	1	0	0	0	1	4
2913	69	2	0	0	0	0	2	0	0	0	1	0	1	0	1	2	4
2914	72	2	0	#NULL!	0	#NULL!	1	0	0	1	1	0	0	0	0	2	4
2915	83	2	0	1	0	1	1	0	0	0	1	0	0	0	0	4	1
2918	78	2	1	1	0	1	3	2	0	0	0	1	1	0	0	2	2

2919	70	1	0	0	0	1	1	0	0	1	0	0	0	0	0	2	4
2921	71	1	0	0	0	1	3	0	1	1	1	0	0	0	1	1	4
2922	68	2	0	0	0	#NULL!	1	2	0	0	1	0	0	0	1	3	1
2927	68	1	0	0	0	0	2	0	1	1	0	0	0	0	0	2	4
2928	67	2	0	0	0	#NULL!	3	0	0	0	0	0	0	0	0	2	1
2931	73	2	0	0	1	1	3	1	1	#NULL!	0	1	0	0	0	3	2
2932	90	1	1	0	1	1	1	1	#NULL!	1	0	0	0	1	0	4	4
2934	77	1	0	0	1	0	1	0	0	1	1	0	0	0	0	3	4
2935	73	2	0	0	0	0	1	0	0	0	1	0	0	0	0	2	2
2936	75	2	0	0	0	1	2	0	0	0	0	0	0	0	0	2	1
2937	75	1	0	0	0	1	1	1	0	1	1	0	1	0	0	1	4
2944	72	1	1	0	1	0	3	2	1	0	0	0	0	0	0	4	4
2945	69	2	0	0	0	1	1	#NULL!	0	0	1	0	0	0	0	3	2
2949	80	2	0	0	0	0	1	0	0	0	0	0	0	0	0	2	2
2951	79	2	0	0	1	#NULL!	3	2	0	0	1	1	0	0	0	2	2
2954	67	1	0	0	1	0	3	2	1	1	0	0	0	0	0	1	4
2961	69	2	0	0	0	1	1	1	0	0	0	0	0	0	0	2	4
2962	74	1	0	0	0	1	1	1	0	0	0	0	0	1	0	1	4
2963	70	2	0	0	0	1	1	0	0	0	0	1	0	0	0	4	3
2964	74	1	1	0	1	1	3	1	1	1	0	0	0	0	0	1	4
2965	72	2	0	0	0	1	1	1	0	0	0	1	0	0	0	2	2
2973	73	2	0	1	1	1	1	0	0	0	0	0	0	0	0	3	3
2976	71	1	0	0	0	1	3	1	1	1	0	0	0	0	0	1	4
2978	76	2	0	0	0	0	1	1	1	1	0	0	0	0	0	4	1
2980	68	2	0	0	0	0	1	1	0	0	1	0	0	0	0	1	1
2982	69	1	0	0	0	1	1	1	0	1	0	0	0	0	0	2	4
2983	77	1	0	0	1	1	2	2	0	1	0	0	1	0	0	3	4
2984	74	2	0	0	0	1	1	1	0	0	0	0	1	0	0	2	2
2987	69	2	0	1	1	#NULL!	#NULL!	0	#NULL!	1	0	1	0	0	0	4	1
2989	75	2	1	0	0	1	1	0	0	0	0	0	1	0	0	1	3
2990	81	1	1	0	1	1	1	1	1	1	0	0	0	0	0	3	4
2991	79	2	0	0	1	1	1	2	0	0	0	0	0	0	0	2	2
2993	81	1	1	0	0	1	1	1	0	0	0	0	1	0	0	3	4
2995	66	1	0	0	0	0	1	1	1	1	0	1	0	0	1	1	4
2996	65	2	0	0	0	0	1	0	0	0	1	0	0	0	0	2	2
2997	67	1	0	0	0	0	3	#NULL!	0	1	0	1	0	0	1	1	4
2999	77	2	1	0	1	1	3	2	0	0	0	0	0	0	0	4	1
3005	78	2	0	1	0	1	1	1	0	0	1	0	0	0	0	2	4
3006	87	1	1	1	1	#NULL!	3	1	0	1	0	0	0	0	0	3	4

3007	74	2	0	1	1	1	2	#NULL!	0	0	0	0	0	1	0	3	2
3012	79	1	0	0	0	0	1	1	0	1	0	1	0	0	0	1	4
3013	75	2	0	0	0	0	2	1	0	0	0	1	0	0	0	2	2
3014	77	1	0	0	0	1	1	1	0	1	0	0	0	0	0	1	4
3015	76	2	0	0	0	1	1	0	0	0	1	0	0	0	0	3	3
3016	68	1	0	0	0	1	3	1	1	1	0	0	0	0	0	1	4
3017	66	2	0	0	0	1	2	1	0	0	0	0	0	0	0	2	2
3020	69	2	0	0	0	1	3	0	0	1	1	0	0	0	0	2	4
3022	72	2	0	1	1	1	2	#NULL!	0	0	0	0	0	0	0	2	4
3026	88	2	0	0	0	0	1	1	0	1	0	0	0	0	0	2	3
3027	79	1	1	0	0	0	3	2	0	0	0	0	0	1	0	1	4
3028	68	1	1	0	0	0	1	1	0	1	1	0	0	0	0	1	4
3030	73	2	0	1	1	0	3	#NULL!	0	0	0	1	0	0	0	3	2
3033	70	1	0	0	0	1	3	2	0	1	0	0	1	0	1	1	4
3034	67	2	0	0	0	1	1	1	0	0	0	0	0	0	1	3	4
3039	80	1	0	0	0	1	1	1	0	1	1	0	0	0	0	1	4
3041	78	1	0	0	0	0	1	1	0	0	0	1	0	0	0	1	4
3042	77	2	0	0	0	1	1	0	0	0	0	0	0	0	1	2	2
3043	81	1	0	0	0	1	1	#NULL!	#NULL!	#NULL!	0	1	#NULL!	0	0	2	4
3044	77	2	1	0	0	1	1	#NULL!	#NULL!	0	1	0	#NULL!	0	0	4	1
3050	87	2	0	1	1	0	2	0	0	1	1	0	0	0	0	2	3
3052	67	1	0	#NULL!	0	#NULL!	#NULL!	#NULL!	#NULL!	#NULL!	0	1	#NULL!	0	1	2	4
3053	65	2	0	0	0	1	3	0	0	0	0	0	0	0	0	1	4
3057	75	1	1	0	1	1	1	1	1	1	0	0	0	1	0	3	4
3058	73	2	1	0	1	1	3	1	0	0	0	0	1	0	0	3	2
3061	70	2	0	0	1	0	1	0	0	1	1	0	0	0	0	3	3
3062	69	1	0	0	1	0	3	1	1	1	0	0	0	0	0	3	1
3064	77	1	0	0	0	0	1	0	1	0	1	0	1	0	0	2	4
3065	74	2	0	0	0	0	3	0	0	0	0	0	0	0	0	1	3
3069	77	2	1	1	1	1	1	0	0	0	0	0	0	0	0	4	1
3072	72	2	0	0	0	1	1	2	0	0	1	0	0	0	0	1	4
3073	76	1	0	0	1	1	1	0	0	0	0	0	0	0	0	2	4
3074	74	2	0	0	1	1	1	1	0	0	1	0	0	0	1	4	4
3076	74	1	0	0	0	0	3	2	1	0	1	0	0	0	0	1	4
3077	70	2	0	0	0	0	1	1	0	0	1	0	0	0	1	2	4
3078	72	2	0	0	0	0	3	0	1	#NULL!	0	1	#NULL!	0	0	4	1
3080	71	2	0	0	1	1	3	1	0	0	0	0	0	0	1	3	4
3081	70	1	0	0	0	1	1	1	0	1	0	1	0	1	0	3	4
3083	79	1	1	0	0	#NULL!	3	2	0	1	0	0	1	1	0	2	4

3086	67	1	0	0	0	1	1	1	0	1	1	0	0	1	1	1	4
3089	67	2	0	0	1	0	3	0	0	0	0	0	0	0	0	3	2
3091	66	1	0	0	0	0	1	0	0	1	1	1	0	0	0	1	4
3092	85	2	0	1	0	#NULL!	1	2	0	0	1	0	0	0	0	4	1
3099	74	2	0	0	1	1	1	0	0	0	0	0	0	0	0	1	4
3105	67	2	0	0	0	1	1	0	0	0	0	0	0	0	0	1	3
3106	66	1	0	0	0	1	1	0	0	1	0	0	1	0	0	3	4
3108	82	2	0	0	0	1	1	0	0	0	0	0	1	0	0	3	2
3110	65	1	0	0	1	#NULL!	1	2	1	1	0	1	0	0	0	2	4
3114	79	2	1	1	0	1	1	1	#NULL!	0	0	0	1	0	0	2	4
3117	76	2	0	0	1	1	3	1	0	0	0	0	0	0	0	3	2
3118	66	2	0	1	0	0	3	0	0	0	0	0	0	0	0	4	1
3120	73	2	0	0	0	1	#NULL!	1	0	1	0	0	0	0	0	2	2
3122	76	2	1	0	1	1	3	1	1	0	0	1	0	0	0	3	2
3123	70	1	0	0	1	0	3	1	0	1	1	0	0	0	0	2	4
3124	78	2	0	0	1	0	1	1	0	0	0	0	0	0	0	4	4
3125	76	1	1	0	0	0	1	0	0	1	1	0	0	0	0	1	4
3126	67	2	0	1	0	1	1	0	0	0	0	0	0	0	0	2	3
3127	84	2	0	1	1	0	2	1	0	1	0	0	1	0	0	2	3
3136	65	2	0	0	0	1	#NULL!	2	0	1	0	0	0	0	1	3	4
3137	71	1	1	0	1	1	3	1	1	0	0	0	1	0	1	3	4
3138	69	2	0	0	0	1	1	1	0	0	0	0	0	0	0	2	2
3144	73	2	0	0	1	1	1	0	0	0	0	0	0	0	0	2	4
3149	72	1	0	0	0	0	3	1	0	1	1	1	0	1	1	1	4
3150	68	2	0	0	0	0	3	1	0	0	0	0	0	0	0	3	4
3152	65	2	0	0	0	0	1	1	0	0	0	0	0	0	0	3	4
3153	68	2	0	1	0	1	1	0	0	0	1	0	1	0	0	1	4
3154	67	1	0	0	0	0	1	1	0	1	0	0	0	0	0	1	4
3155	67	1	0	0	0	0	1	1	0	0	0	0	0	0	1	1	4
3157	67	1	0	0	0	1	1	0	1	0	1	1	0	0	0	1	4
3159	71	2	0	1	0	0	1	#NULL!	0	0	0	0	0	0	0	2	3
3162	73	2	0	0	0	1	1	0	0	0	1	1	0	0	1	2	3
3164	68	2	0	1	0	0	1	1	0	0	0	0	0	0	0	2	4
3165	88	1	0	1	0	0	2	0	0	0	0	0	0	0	0	2	4
3167	75	2	0	0	1	1	1	1	0	0	0	0	0	0	0	2	1
3170	69	1	1	0	0	0	1	2	0	1	0	0	1	0	0	3	4
3171	67	2	0	0	0	#NULL!	#NULL!	1	1	0	0	0	0	0	0	3	3
3173	72	1	0	0	0	1	1	0	0	0	0	0	0	0	0	1	4
3174	66	2	0	0	0	1	1	#NULL!	0	0	0	0	0	0	0	2	3

3176	65	1	0	0	0	1	1	1	0	1	0	0	0	0	1	1	4
3182	69	1	0	0	0	1	1	0	0	1	0	0	0	0	0	1	4
3183	65	2	0	0	0	1	1	0	0	0	0	0	0	0	0	2	4
3188	81	1	1	0	0	0	1	1	0	0	0	0	0	0	0	2	4
3190	77	2	1	1	0	1	1	0	1	1	1	0	0	0	0	2	4
3193	72	1	0	0	0	0	3	2	0	1	1	1	0	0	0	1	4
3194	67	2	0	0	0	1	1	1	0	0	0	0	0	0	1	3	3
3195	76	2	0	1	0	1	1	1	1	1	0	0	1	0	0	2	4
3196	67	1	0	1	#NULL!	1	1	1	1	1	0	1	0	#NULL!	1	3	2
3198	72	2	0	0	0	0	3	1	0	0	0	0	0	0	0	2	1
3202	79	2	0	1	0	0	1	0	0	0	0	0	0	0	0	2	2
3203	81	2	0	1	0	0	1	1	0	0	0	0	0	0	0	1	2
3204	68	2	0	1	0	1	1	2	0	0	0	0	0	0	1	1	4
3206	75	2	0	1	0	1	3	2	0	0	0	0	0	1	0	2	2
3207	89	2	0	1	0	0	1	0	0	0	0	1	1	0	0	2	2
3210	67	2	0	0	0	1	1	0	0	0	1	0	0	0	0	2	2
3215	74	2	0	0	1	1	1	1	1	0	1	0	0	0	1	2	4
3216	66	1	0	0	0	1	1	#NULL!	0	0	0	0	0	0	1	2	4
3217	71	1	0	0	0	1	1	0	0	1	0	0	1	0	0	1	4
3220	75	1	0	0	0	1	1	2	1	1	0	0	1	0	0	1	4
3221	73	2	1	0	1	1	3	1	0	0	0	0	#NULL!	0	0	2	2
3222	71	1	0	0	0	1	1	1	1	0	0	0	0	0	0	3	1
3223	65	2	0	1	0	1	1	0	0	1	1	0	0	0	0	2	1
3224	77	2	0	#NULL!	1	#NULL!	3	#NULL!	#NULL!	#NULL!	0	0	#NULL!	0	0	2	4
3225	79	1	0	1	0	1	3	#NULL!	#NULL!	#NULL!	0	0	#NULL!	0	1	2	4
3227	74	2	1	1	0	1	3	1	0	0	0	0	1	0	0	4	1
3229	82	1	1	0	1	1	#NULL!	1	0	1	0	0	1	0	0	1	4
3233	78	2	0	1	0	1	1	2	0	0	0	0	0	0	0	2	3
3236	72	2	0	1	0	1	1	#NULL!	0	0	0	1	#NULL!	0	0	4	4
3237	65	1	0	0	0	1	1	0	0	1	1	0	0	0	1	1	4
3240	71	2	0	1	0	#NULL!	1	0	0	0	0	1	1	0	1	3	4
3244	71	2	0	0	0	#NULL!	1	2	0	0	0	0	0	0	0	4	1
3246	66	2	0	0	0	0	1	0	1	0	1	1	0	0	0	2	1
3247	74	1	1	0	0	1	1	1	1	1	0	0	0	0	0	3	4
3248	69	2	0	0	0	1	1	1	0	0	0	0	0	0	0	3	4
3250	73	1	0	0	0	1	1	1	0	0	0	1	0	0	0	1	4
3251	66	2	0	0	0	1	1	1	0	0	0	0	0	0	0	2	2
3253	71	1	0	0	0	1	1	#NULL!	0	1	1	1	0	0	0	1	4
3254	67	2	0	0	0	1	1	0	0	1	0	0	0	0	0	2	1

3257	78	1	0	0	0	1	3	1	0	1	1	0	1	0	0	1	4
3261	69	1	1	0	1	0	1	0	1	1	1	0	0	1	0	2	4
3262	78	2	0	1	0	0	1	0	0	0	0	0	0	0	0	4	4
3267	65	1	0	0	0	0	3	1	1	0	0	0	0	0	1	2	4
3271	69	1	0	0	0	1	3	0	1	1	0	1	0	0	0	4	4
3272	69	1	0	0	0	1	3	1	1	0	0	0	0	0	0	1	4
3274	83	2	1	1	1	1	2	0	0	0	0	0	1	0	0	3	3
3278	74	2	0	1	0	1	3	1	0	1	0	1	0	0	0	2	2
3279	72	1	0	0	1	1	1	#NULL!	#NULL!	#NULL!	0	0	#NULL!	0	1	1	4
3280	68	1	0	0	0	0	1	0	1	1	0	0	0	0	0	1	4
3281	66	2	0	0	0	0	1	0	1	0	1	0	0	0	0	2	1
3283	73	1	0	0	0	0	1	1	1	0	0	0	1	0	1	1	4
3284	66	1	1	1	1	1	3	0	1	1	0	0	1	0	0	2	4
3288	66	1	0	0	0	1	1	0	0	0	0	1	0	0	1	3	4
3289	65	2	0	0	0	1	1	#NULL!	0	0	0	0	0	0	1	4	4
3292	67	1	0	0	0	0	1	1	1	1	0	0	0	0	1	1	4
3294	66	2	0	0	0	0	3	2	0	0	0	0	0	0	1	2	4
3295	67	2	0	0	0	0	1	0	0	0	0	0	1	0	0	2	2
3297	67	1	0	0	0	1	1	0	1	1	0	0	0	0	1	2	4
3300	66	2	1	1	1	1	1	0	0	0	0	0	0	0	0	3	4
3302	72	1	0	0	1	1	3	#NULL!	1	1	0	0	#NULL!	0	1	2	4
3309	74	2	0	0	0	0	1	0	0	0	0	0	0	0	1	2	4
3312	79	1	1	0	1	1	3	1	0	0	0	0	0	0	0	3	4
3313	77	2	0	0	0	1	1	2	0	0	1	0	0	0	0	2	4
3315	86	2	1	0	0	1	1	1	0	0	0	0	1	0	0	4	1
3316	73	2	0	1	1	1	3	1	0	0	0	0	0	0	0	3	2
3318	69	2	0	1	1	1	1	1	0	0	1	1	0	0	1	2	4
3324	65	1	0	0	0	0	3	0	0	1	0	1	0	1	1	2	4
3334	65	2	0	0	1	1	3	0	0	1	0	0	0	0	0	2	3
3336	80	1	1	0	1	1	1	#NULL!	#NULL!	#NULL!	0	0	#NULL!	1	0	3	4
3342	79	2	0	0	0	1	1	1	0	0	0	0	1	0	0	4	1
3343	77	1	1	0	1	1	1	1	0	0	1	1	0	0	0	3	4
3349	65	2	0	1	1	1	3	1	0	0	0	0	0	0	0	3	4
3352	68	2	0	1	0	0	#NULL!	1	1	0	0	0	0	0	1	1	4
3353	79	2	0	1	1	1	3	1	0	0	0	1	0	0	0	2	2
3354	72	1	1	0	1	0	3	1	0	1	0	0	0	0	0	3	1
3355	70	2	0	0	0	0	1	1	0	1	0	0	0	0	0	1	2
3358	76	2	0	1	0	1	1	2	0	0	0	0	0	0	0	2	2
3360	77	2	0	1	1	1	#NULL!	#NULL!	#NULL!	#NULL!	0	0	#NULL!	0	0	4	4

3361	83	1	0	0	0	0	3	#NULL!	#NULL!	#NULL!	1	0	#NULL!	0	0	1	4
3362	76	2	0	0	0	0	3	0	0	0	0	0	0	0	0	4	1
3364	66	1	0	0	1	1	1	1	0	1	0	1	0	0	1	3	4
3367	69	1	0	0	0	0	1	1	0	0	0	0	0	0	1	1	4
3368	68	2	0	0	0	0	2	0	0	0	0	0	0	0	0	4	1
3370	65	2	0	0	0	0	2	0	0	0	0	0	0	0	0	4	1
3371	65	1	0	0	0	0	1	1	0	1	0	0	0	1	0	4	4
3372	76	2	0	#NULL!	0	#NULL!	1	#NULL!	#NULL!	#NULL!	1	1	#NULL!	0	0	4	1
3373	85	2	1	1	0	0	1	0	0	0	0	1	0	0	0	1	4
3374	75	1	0	0	0	0	1	#NULL!	#NULL!	#NULL!	0	0	#NULL!	0	1	1	4
3375	68	2	0	0	0	0	1	0	0	0	0	1	0	0	0	4	1
3376	82	2	0	1	1	0	2	0	0	0	0	0	0	0	0	4	1
3377	72	1	0	0	0	0	3	1	0	1	1	1	0	0	1	1	4
3378	66	2	0	0	0	0	1	0	0	0	0	0	0	0	0	4	1
3379	78	2	1	1	0	1	2	#NULL!	0	1	0	0	#NULL!	0	0	2	4
3382	67	1	0	0	0	1	1	1	0	0	0	0	1	1	1	2	4
3384	70	2	0	0	0	1	1	0	0	0	0	0	1	0	0	2	2
3385	69	1	0	0	0	0	1	0	0	1	0	0	0	0	1	2	4
3389	76	1	0	0	1	1	1	2	1	1	0	1	0	0	1	3	4
3391	73	2	0	1	1	1	1	#NULL!	0	1	1	0	0	0	0	4	4
3395	71	1	0	0	0	1	1	2	0	1	1	0	0	1	0	1	4
3396	66	2	0	0	0	1	3	1	0	0	1	1	0	0	0	2	1
3399	66	2	0	0	0	1	1	1	0	0	1	0	0	0	0	2	3
3400	65	1	0	0	0	1	3	0	0	1	1	0	0	0	1	1	4
3402	90	1	1	0	0	1	3	1	0	1	0	1	0	0	0	1	4
3403	82	2	1	0	0	1	3	1	0	#NULL!	0	0	0	0	0	2	4
3404	79	1	0	0	0	0	1	0	0	1	1	1	0	1	0	4	1
3405	72	2	0	0	0	0	1	0	0	0	0	0	0	0	0	4	1
3408	77	1	0	0	0	0	1	2	0	1	1	1	0	0	0	2	4
3409	74	2	0	0	0	0	1	2	0	0	0	1	0	0	0	1	4
3413	76	1	0	0	0	0	1	#NULL!	1	1	1	1	0	0	0	4	1
3417	69	2	0	1	0	#NULL!	1	0	0	0	0	0	0	0	0	3	4
3418	67	1	0	0	0	1	3	1	1	1	0	1	0	1	0	1	4
3419	75	2	0	1	0	1	1	0	0	0	0	0	0	0	0	4	1
3421	78	2	0	1	0	0	2	0	0	0	0	0	0	0	0	4	1
3423	72	2	0	1	1	1	1	0	0	0	0	0	0	0	1	3	4
3424	67	1	0	0	0	0	1	0	1	0	0	0	1	0	1	1	4
3426	76	2	0	0	0	0	1	0	0	0	1	0	0	0	1	1	4
3427	89	2	1	1	1	0	1	1	#NULL!	#NULL!	0	1	1	0	0	2	4

3430	69	1	0	0	1	1	3	2	0	0	0	0	0	0	1	3	4
3431	67	2	0	0	0	1	1	1	0	0	0	0	0	0	1	3	4
3433	85	1	1	0	1	1	1	1	0	1	1	0	0	#NULL!	0	3	4
3437	79	2	1	0	1	1	1	1	#NULL!	#NULL!	1	1	1	0	0	4	1
3438	82	2	0	1	1	0	1	#NULL!	0	0	0	0	0	0	0	4	1
3439	77	1	0	0	1	0	1	1	1	1	0	0	0	0	0	4	4
3440	74	2	0	0	1	0	1	0	0	0	1	1	0	0	0	2	4
3441	73	1	0	0	1	1	3	0	0	0	0	0	0	1	0	1	4
3442	65	2	0	0	1	1	3	1	0	0	0	0	0	0	0	1	2
3446	77	1	0	0	1	1	1	0	0	1	0	0	0	0	0	1	4
3451	65	1	0	0	0	1	1	1	0	1	1	1	0	0	1	1	4
3453	80	1	0	0	0	1	1	0	1	1	0	0	0	0	0	3	4
3454	77	2	0	0	1	1	1	0	0	0	0	0	0	0	0	3	4
3455	68	2	0	#NULL!	0	#NULL!	1	#NULL!	#NULL!	#NULL!	0	0	#NULL!	0	0	2	2
3456	82	1	1	0	1	1	#NULL!	#NULL!	0	1	0	0	1	1	0	3	4
3457	81	2	0	0	1	1	1	0	0	0	0	1	0	0	0	2	4
3458	70	2	0	0	0	0	1	2	0	0	1	0	0	0	0	2	2
3459	70	1	0	0	0	0	3	2	1	1	0	0	1	0	0	1	4
3464	74	2	0	1	0	1	1	1	0	1	1	1	0	0	0	2	4
3475	72	2	0	1	1	1	1	0	0	0	0	0	1	0	0	2	4
3476	66	1	0	1	1	#NULL!	3	1	0	1	0	0	0	0	1	3	4
3481	75	2	1	0	1	1	3	1	0	0	0	0	0	0	0	1	4
3487	69	2	0	1	0	1	1	1	0	0	0	1	0	0	0	2	4
3492	77	1	0	0	1	0	1	1	0	1	1	1	0	0	0	3	4
3493	72	2	0	0	1	0	1	#NULL!	0	0	1	1	0	0	0	3	4
3497	71	2	0	1	0	1	1	1	0	0	1	1	0	0	0	2	4
3498	65	1	0	1	0	0	1	2	0	1	1	1	0	0	0	2	4
3499	71	2	0	1	0	1	1	0	0	0	0	0	0	0	0	2	2
3502	65	1	0	0	0	0	3	2	1	1	1	0	0	0	0	1	4
3511	78	1	0	0	0	1	1	0	1	1	0	0	0	0	0	1	4
3512	76	2	0	0	0	#NULL!	2	0	0	0	0	1	0	0	0	4	1
3513	84	2	0	1	0	0	1	1	0	0	0	1	0	0	0	4	1
3518	70	2	0	1	0	0	2	0	0	0	1	1	0	0	1	2	4
3519	86	2	1	1	0	1	3	1	0	0	0	0	1	1	0	2	4
3521	67	1	0	1	0	0	1	1	0	0	0	1	0	0	0	1	4
3528	69	2	0	1	0	1	1	1	0	0	1	0	0	0	0	2	4
3529	66	1	0	0	1	1	1	0	0	1	0	0	1	0	0	1	4
3531	72	2	1	0	1	1	1	1	0	0	0	0	1	0	0	3	2
3532	85	2	0	1	0	#NULL!	1	1	0	0	0	0	0	0	1	3	2

3534	80	2	0	0	0	1	1	0	0	0	1	0	0	0	0	2	3
3535	71	2	0	1	0	1	1	0	0	0	0	0	0	0	0	4	1
3536	66	1	0	0	0	1	1	2	0	1	0	1	0	0	0	1	4
3538	71	1	0	1	1	1	3	0	0	1	0	0	0	0	1	3	4
3545	72	1	1	0	0	1	1	1	0	1	1	0	0	0	0	1	4
3546	65	2	0	0	0	1	3	1	0	0	0	0	0	0	1	1	2
3548	71	2	0	1	1	1	1	0	0	0	0	1	0	0	0	1	4
3549	78	1	0	0	0	1	1	#NULL!	#NULL!	#NULL!	0	1	#NULL!	0	1	3	4
3550	74	2	1	0	0	1	3	1	0	0	1	1	0	0	1	3	4
3552	66	2	0	0	0	1	1	1	0	0	1	0	0	0	0	4	1
3556	67	1	0	0	0	1	1	1	1	1	1	0	0	0	1	1	4
3557	66	2	0	0	0	1	1	0	0	0	0	0	0	0	0	2	1
3558	77	2	1	1	1	1	3	2	0	0	1	1	1	0	0	3	1
3559	73	2	0	1	0	1	1	0	0	0	1	0	0	0	0	3	2
3560	69	1	0	0	0	0	1	1	1	1	0	0	0	0	1	1	4
3561	74	1	0	1	0	1	1	1	0	0	0	0	1	0	0	2	4
3565	68	1	0	0	0	1	1	1	0	1	1	0	0	0	1	1	4
3566	69	1	0	0	0	1	1	1	1	1	0	0	0	0	1	2	4
3567	68	2	0	1	0	0	1	1	0	0	0	0	0	0	1	2	4
3568	87	2	0	0	0	1	2	#NULL!	#NULL!	#NULL!	0	0	#NULL!	0	0	3	4
3571	81	1	1	0	1	0	1	0	0	0	0	0	1	0	0	1	4
3574	71	1	0	0	0	1	1	0	0	1	0	0	0	0	0	2	4
3579	75	1	0	0	1	#NULL!	1	2	0	0	0	0	0	1	0	3	4
3580	69	2	0	0	0	1	2	0	0	0	0	0	0	0	0	2	4
3581	68	1	0	0	0	1	3	1	0	1	1	1	1	0	0	1	4
3582	67	2	0	0	0	1	1	0	0	0	1	1	0	0	0	3	3
3584	74	2	0	1	0	1	1	0	0	0	#NULL!	#NULL!	0	0	1	2	3
3588	73	2	0	1	0	1	2	0	0	0	0	0	1	0	1	2	4
3589	76	2	0	1	1	1	3	1	0	0	1	0	0	0	0	3	4
3590	79	1	1	0	0	#NULL!	1	2	0	0	1	0	1	0	0	1	4
3591	73	2	0	0	0	0	1	1	0	0	1	0	0	0	0	1	1
3593	77	2	0	0	0	1	1	1	0	0	1	0	0	0	0	2	3
3594	72	2	1	0	0	#NULL!	#NULL!	#NULL!	0	0	0	1	#NULL!	0	0	2	1
3595	71	1	1	0	0	1	3	1	0	1	0	0	0	0	0	1	4
3597	74	2	0	0	0	0	3	1	0	0	1	0	1	0	0	2	2
3598	76	2	0	1	0	1	1	1	0	0	0	0	0	0	0	3	4
3603	70	1	0	0	1	0	1	0	1	1	1	0	0	0	0	3	4
3604	65	2	0	0	1	0	3	1	0	0	0	0	0	0	1	1	3
3605	71	1	0	0	0	1	1	1	1	1	1	0	0	0	0	2	4

3606	74	2	0	0	0	#NULL!	1	2	0	0	0	0	0	0	0	2	3
3607	73	1	1	0	0	0	1	1	1	1	0	0	0	0	1	2	4
3611	73	2	0	0	0	1	1	0	0	0	1	1	1	0	0	2	2
3614	80	2	0	1	0	0	1	#NULL!	0	0	0	0	0	0	0	4	1
3616	80	2	0	1	1	1	1	0	0	0	0	0	0	0	0	3	3
3617	79	1	1	0	0	1	1	2	1	1	0	1	0	0	0	1	4
3618	76	2	0	0	0	1	#NULL!	0	0	0	0	0	0	0	0	4	1
3619	67	2	0	0	1	#NULL!	3	2	0	0	0	0	0	0	0	3	3
3622	70	1	0	0	0	1	3	0	0	1	1	0	1	0	0	2	4
3624	83	1	0	0	0	0	1	2	0	1	1	0	1	0	0	1	4
3625	77	2	0	0	1	0	1	0	0	1	0	0	0	1	0	2	2
3629	74	1	0	0	0	#NULL!	1	0	0	1	1	0	0	0	0	3	4
3630	71	2	0	0	1	1	3	#NULL!	0	0	0	0	0	0	0	2	2
3634	67	1	0	0	0	0	3	0	0	1	0	0	0	0	1	1	4
3636	82	2	0	1	1	0	3	1	0	0	0	0	1	1	0	4	1
3638	74	2	1	0	1	1	1	1	0	0	0	0	0	1	0	3	1
3644	70	2	0	0	0	0	1	0	0	0	1	1	1	0	0	2	2
3646	65	1	0	0	0	1	1	0	0	1	1	0	0	0	1	2	4
3647	87	1	1	0	1	1	#NULL!	1	1	1	1	0	0	0	0	3	4
3651	75	2	0	0	0	1	1	0	0	0	0	0	0	0	0	2	2
3654	80	1	0	0	1	#NULL!	#NULL!	#NULL!	0	0	0	0	#NULL!	0	0	3	1
3655	75	2	1	0	0	1	1	1	0	0	0	0	1	0	0	4	4
3657	66	1	0	0	0	0	3	2	1	1	1	0	0	0	0	2	4
3659	68	1	0	0	0	0	3	1	1	1	1	0	0	0	1	1	4
3664	73	1	0	0	1	#NULL!	1	#NULL!	1	0	0	0	0	0	0	4	4
3665	69	2	0	0	0	1	3	#NULL!	0	0	0	0	0	0	1	2	4
3672	74	2	1	0	0	0	1	1	1	1	1	0	0	0	0	3	3
3676	71	1	1	0	0	1	1	0	0	0	1	0	0	0	1	2	4
3677	69	2	0	0	0	1	1	0	0	0	0	0	0	0	0	2	3
3678	76	1	0	0	0	0	1	1	0	0	0	0	0	0	0	1	4
3681	66	2	0	0	1	0	3	#NULL!	0	0	1	0	0	0	1	1	2
3684	76	1	0	0	0	1	1	0	0	1	0	0	1	0	0	1	4
3685	75	2	0	0	1	1	3	1	0	0	0	0	1	0	0	3	4
3687	69	1	0	0	0	0	1	1	0	1	1	0	0	0	0	1	4
3688	66	2	0	0	0	0	1	2	0	0	0	0	0	0	0	2	1
3692	65	2	0	1	0	1	1	0	0	0	0	0	1	0	0	3	4
3693	75	1	0	0	0	1	1	0	1	1	0	0	0	0	0	1	4
3694	70	2	0	0	0	1	1	0	0	0	0	0	0	0	0	2	3
3697	67	2	0	1	0	1	1	1	0	0	0	0	0	0	0	2	1

3699	66	1	0	0	0	0	1	0	0	1	0	0	0	0	0	3	4
3702	71	1	0	0	0	1	1	1	0	0	0	1	0	0	0	1	4
3703	65	2	0	0	0	1	1	0	0	0	0	0	0	0	0	4	1
3704	66	1	0	0	0	1	3	1	1	1	1	1	0	0	0	2	4
3712	74	1	0	0	0	#NULL!	1	0	0	1	0	0	0	0	0	2	4
3713	73	2	0	0	1	1	1	0	0	0	0	0	0	0	0	2	2
3714	69	1	0	1	0	1	1	1	0	1	1	0	0	0	1	1	4
3716	70	2	0	0	0	0	3	0	0	0	0	0	0	0	0	4	3
3724	66	2	0	0	0	1	1	0	0	0	0	0	1	0	1	3	4
3730	79	2	0	0	0	1	1	1	1	#NULL!	0	0	1	0	0	2	2
3734	75	1	0	0	0	1	1	0	0	1	1	0	0	0	1	4	4
3735	68	2	0	0	0	0	2	1	0	0	0	1	0	0	0	2	4
3736	74	1	0	0	0	#NULL!	1	0	0	0	1	1	0	0	0	1	4
3737	69	2	0	0	0	0	1	0	0	0	0	1	0	0	0	1	1
3739	72	1	0	0	0	0	1	0	0	0	1	1	0	0	1	1	4
3740	66	2	0	0	0	0	1	1	0	0	1	0	0	0	0	3	3
3741	69	2	0	1	0	1	1	0	1	0	0	1	0	0	0	3	3
3742	80	2	0	0	0	#NULL!	3	0	0	0	0	0	0	0	0	2	4
3744	67	2	1	0	1	0	1	0	0	0	0	0	0	0	0	3	2
3745	72	2	0	1	0	0	1	0	0	0	1	0	0	0	0	2	1
3746	73	1	0	0	0	1	1	1	1	1	1	1	0	1	0	1	4
3747	69	2	0	0	0	1	1	0	0	0	0	1	0	0	0	2	3
3750	74	1	0	0	0	0	1	0	1	0	1	1	0	0	0	1	4
3751	74	2	0	0	0	0	1	0	0	0	0	0	0	0	0	2	4
3753	73	2	0	0	0	1	1	1	0	0	0	0	0	0	0	2	3
3754	72	1	0	0	0	1	3	#NULL!	#NULL!	#NULL!	1	1	#NULL!	0	0	2	4
3756	76	1	0	0	1	1	1	1	0	1	1	1	0	0	0	2	4
3757	65	2	1	0	1	0	3	1	0	0	1	1	0	0	1	3	1
3758	70	1	0	0	0	0	1	2	0	1	1	1	0	0	0	1	4
3763	74	1	0	0	0	1	1	0	0	1	1	0	0	1	0	1	4
3764	70	2	0	0	1	1	1	0	0	0	1	0	0	0	0	1	4
3766	67	1	0	0	0	1	3	0	0	1	0	1	0	1	1	2	4
3767	70	1	0	0	1	0	1	1	1	0	1	0	0	0	1	1	4
3768	67	2	0	0	0	0	1	0	0	0	0	1	0	0	1	2	4
3771	71	1	0	0	0	1	1	1	0	0	0	0	0	1	0	1	4
3772	65	2	0	0	0	1	1	1	0	0	0	0	1	0	0	1	2
3774	83	2	0	1	0	1	3	2	0	0	1	0	0	0	0	1	4
3776	70	1	0	0	0	0	1	1	1	1	1	1	0	0	1	1	4
3777	69	2	0	0	0	0	3	1	0	0	0	0	0	0	0	2	2

3779	65	2	0	0	0	#NULL!	1	0	0	0	1	1	0	0	1	4	4
3788	67	1	0	0	0	#NULL!	1	0	0	0	0	0	0	0	1	1	4
3789	68	1	0	0	0	0	1	0	1	1	0	0	0	1	1	1	4
3790	76	1	0	0	0	1	1	1	1	1	1	1	0	0	1	4	4
3792	81	1	0	0	0	1	2	0	0	0	1	0	1	0	0	1	3
3793	76	2	0	0	0	1	1	0	0	0	1	0	1	0	0	2	3
3801	72	1	0	0	0	0	1	2	0	0	1	1	0	0	0	2	4
3802	71	2	0	0	1	0	1	1	0	0	0	0	0	0	0	2	4
3803	82	2	0	1	0	0	1	1	0	0	0	0	1	0	0	3	3
3804	71	1	0	0	0	1	1	0	0	0	0	0	0	0	0	3	4
3807	77	2	0	0	0	0	1	1	0	0	1	0	0	0	0	1	1
3815	73	2	0	1	1	1	3	0	0	0	0	0	0	0	0	4	2
3819	68	2	0	0	0	1	1	1	0	0	1	0	0	0	0	3	4
3820	74	1	0	0	1	1	3	0	0	1	0	0	0	0	0	3	4
3821	70	2	0	0	0	1	3	1	0	0	0	0	0	0	0	3	4
3822	76	2	0	0	0	1	1	2	0	0	0	0	1	0	0	2	4
3823	74	1	0	0	0	0	1	0	0	1	0	0	0	0	0	2	4
3827	70	2	0	1	0	0	1	0	0	0	1	1	0	0	0	4	4
3831	82	2	1	0	1	1	1	1	0	0	0	0	0	0	0	4	4
3834	67	2	0	0	0	0	1	1	0	0	0	0	0	0	0	2	4
3835	79	1	0	0	0	1	2	1	1	1	0	0	0	0	0	3	4
3840	68	1	0	0	0	1	1	0	0	1	0	0	0	0	0	1	4
3841	66	2	0	0	0	1	1	0	0	0	1	0	0	0	0	2	3
3848	65	2	0	0	0	0	1	0	1	0	1	0	0	0	0	2	3
3854	74	2	0	0	1	0	3	1	0	0	0	0	0	0	0	2	4
3856	70	2	0	1	0	1	1	0	0	0	0	1	0	0	0	2	4
3858	71	1	0	0	0	0	1	2	#NULL!	#NULL!	0	0	#NULL!	0	1	3	2
3859	66	2	0	0	0	0	1	0	0	0	0	1	1	0	0	2	2
3861	74	2	0	1	1	0	1	1	0	0	0	0	0	0	1	3	4
3865	83	2	1	0	0	1	1	0	0	0	0	0	0	1	0	4	1
3866	73	1	1	0	1	1	3	1	1	0	0	0	0	1	0	3	4
3867	71	2	1	0	0	1	1	1	0	0	0	0	1	0	0	2	1
3868	73	1	0	0	0	0	1	1	0	1	0	0	0	1	0	2	4
3869	70	2	0	0	0	0	2	2	0	0	0	0	0	0	0	2	1
3871	68	1	0	0	1	1	1	0	1	1	0	0	1	0	1	3	4
3873	67	2	0	0	0	1	1	0	0	0	0	0	0	0	0	2	1
3875	73	1	0	0	0	0	3	1	1	1	0	0	0	0	1	1	2
3876	71	2	0	0	0	0	3	2	0	0	0	0	0	0	1	2	2
3878	69	1	0	0	1	0	3	1	1	1	1	0	0	0	0	3	4

3880	72	2	1	0	0	1	1	0	0	0	1	0	0	0	0	2	4
3881	80	1	1	0	1	1	3	0	0	1	0	0	1	0	0	2	4
3884	67	2	0	0	0	0	3	0	0	0	0	1	0	0	1	2	3
3885	82	1	0	0	0	1	1	1	1	0	0	0	1	0	0	3	1
3889	66	1	0	0	0	#NULL!	1	1	1	1	0	0	0	0	1	2	4
3890	74	1	0	0	0	0	3	1	0	0	0	0	1	1	1	1	4
3891	70	2	0	0	0	0	1	1	0	0	1	1	1	0	0	2	4
3893	71	1	0	0	0	1	1	1	0	0	1	0	0	0	0	1	4
3897	68	1	0	0	0	1	1	0	1	0	0	0	1	1	0	1	4
3899	70	1	0	0	0	0	1	1	1	1	1	0	0	0	0	2	4
3900	70	2	0	0	0	0	1	0	1	0	0	0	0	0	0	1	1
3901	67	1	0	0	0	1	1	1	0	1	0	0	0	0	0	2	4
3902	66	1	0	0	1	1	3	2	0	1	1	0	0	0	0	2	4
3904	90	2	1	1	0	1	1	1	0	1	0	0	1	1	0	4	1
3905	70	1	0	0	0	1	1	1	0	1	0	0	0	0	1	1	4
3906	75	1	1	0	1	1	1	2	0	1	0	0	1	0	0	3	4
3907	70	2	0	0	0	1	1	#NULL!	0	0	1	0	0	0	0	2	3
3908	66	1	0	0	0	1	1	0	0	1	0	0	0	0	0	1	4
3910	86	2	0	1	0	1	1	2	0	0	0	0	0	0	0	2	4
3912	70	2	0	0	0	0	1	0	0	0	1	0	0	0	0	2	3
3914	67	2	0	0	1	1	1	0	0	0	1	0	0	0	0	3	2
3916	76	1	1	0	0	1	3	#NULL!	0	1	0	0	0	0	0	2	4
3917	73	2	0	0	1	1	1	#NULL!	0	0	0	0	0	0	0	2	3
3920	67	1	1	0	0	0	1	0	0	0	0	0	1	1	0	1	4
3921	65	2	1	0	0	0	1	0	0	0	0	0	0	0	1	3	4
3922	65	1	0	0	0	0	1	0	0	0	0	0	0	0	0	1	4
3923	68	1	0	0	0	1	1	#NULL!	0	1	0	0	0	0	0	1	4
3930	67	1	0	0	0	#NULL!	1	0	0	0	0	0	0	0	0	1	4
3934	67	2	0	1	1	0	1	0	0	0	0	1	0	0	0	4	1
3935	65	1	0	0	0	0	1	0	0	1	0	1	0	0	1	1	4
3941	72	2	0	1	0	1	3	#NULL!	0	0	0	1	0	0	0	2	3
3942	74	2	0	1	0	0	1	0	0	0	0	0	1	0	0	4	1
3944	71	1	1	0	1	1	1	0	0	0	0	0	0	1	1	4	4
3946	65	1	0	0	0	1	1	1	1	1	0	0	0	0	0	2	4
3947	76	2	0	1	1	1	1	0	0	0	1	1	0	0	0	1	2
3949	77	2	1	#NULL!	1	#NULL!	1	2	0	#NULL!	1	1	1	0	0	2	4
3950	70	1	1	0	1	1	3	0	0	1	1	0	1	0	0	1	4
3951	67	2	0	0	0	1	1	0	0	0	1	0	0	0	0	3	4
3952	73	1	1	0	1	1	1	0	0	1	0	0	1	1	1	3	4

3955	71	1	0	0	0	1	3	1	0	0	0	0	0	0	1	4	4
3957	76	1	0	0	0	1	1	1	0	0	1	1	0	0	0	2	1
3958	69	1	0	0	0	1	2	0	0	1	0	0	0	0	0	3	4
3959	66	2	0	0	0	1	1	2	0	1	0	0	0	0	0	2	4
3967	69	2	0	0	0	1	3	1	0	0	0	1	0	0	1	2	4
3969	78	1	0	0	0	1	3	0	0	1	1	0	1	0	0	2	4
3970	74	2	0	0	0	1	1	1	0	0	0	1	0	0	0	2	4
3974	69	2	0	0	1	1	3	1	0	0	0	1	0	0	0	3	3
3976	66	1	0	0	0	0	1	0	1	1	0	1	0	0	0	1	4
3981	69	2	0	0	0	0	1	0	0	0	1	0	0	0	0	2	2
3983	79	1	0	0	0	1	1	#NULL!	0	0	0	1	0	0	0	2	4
3987	81	1	1	0	1	1	3	0	0	0	0	0	1	0	0	3	4
3988	71	2	0	0	#NULL!	#NULL!	3	1	0	0	#NULL!	#NULL!	0	0	1	2	4
3990	81	1	0	#NULL!	0	#NULL!	1	#NULL!	#NULL!	#NULL!	1	1	#NULL!	0	1	2	4
3991	74	2	0	0	0	0	1	#NULL!	0	0	1	1	0	0	0	3	3
3992	79	1	0	0	0	1	1	0	0	0	0	1	0	0	0	3	4
3993	74	1	0	0	0	1	1	2	0	0	0	0	#NULL!	0	0	4	4
3995	66	2	0	0	0	1	1	0	0	0	0	0	0	0	1	2	3
3996	72	1	1	1	0	1	1	0	0	0	0	0	1	0	0	1	4
3997	83	2	1	1	1	#NULL!	1	0	0	1	0	0	1	0	0	2	3
3998	69	1	0	0	0	0	1	1	0	1	1	0	0	0	0	1	4
4000	73	1	0	0	1	1	1	2	1	1	0	0	0	0	1	3	2
4001	69	2	0	0	1	1	1	1	0	0	1	0	0	0	0	3	2
4005	76	2	0	0	0	#NULL!	3	1	0	0	1	0	0	0	0	3	1
4006	76	1	0	0	1	1	3	1	0	0	0	0	0	0	1	3	4
4007	74	2	0	0	0	1	1	2	0	0	1	1	0	0	1	3	4
4008	77	1	0	0	0	0	1	0	1	1	0	0	0	0	0	4	1
4009	69	2	0	0	0	1	1	#NULL!	0	0	1	1	1	0	0	2	1
4010	68	1	0	0	0	0	1	0	1	1	1	0	0	#NULL!	1	1	4
4011	66	2	0	0	0	0	1	0	0	0	0	0	0	0	0	1	4
4016	67	2	0	0	1	1	3	0	0	0	0	0	1	0	0	3	4
4017	73	2	0	1	0	1	1	1	0	1	1	0	0	0	0	1	3
4018	69	2	0	1	0	1	1	0	0	0	0	0	0	0	0	2	4
4019	66	2	0	1	1	1	1	0	0	0	1	1	0	0	1	4	2
4025	77	2	0	0	0	0	3	#NULL!	#NULL!	#NULL!	1	0	#NULL!	0	0	2	1
4028	69	2	0	1	0	0	3	0	0	0	1	1	0	0	0	3	2
4029	67	1	0	0	0	1	1	1	0	1	1	1	0	0	1	2	4
4031	80	1	0	0	0	0	3	2	0	1	0	0	0	0	0	1	4
4032	79	2	0	0	0	0	1	0	0	0	1	0	1	0	0	2	1

4037	77	1	1	0	0	1	1	1	0	1	0	0	1	0	0	2	1
4041	75	2	0	0	0	#NULL!	#NULL!	0	0	0	1	1	1	0	0	2	2
4043	77	1	0	0	0	0	3	2	1	1	0	0	0	0	0	1	4
4048	68	1	0	0	0	1	1	1	0	1	0	0	0	0	0	3	4
4051	78	1	0	0	0	0	1	2	0	1	0	0	0	0	1	1	4
4053	71	2	0	0	0	0	3	0	0	0	0	1	0	0	1	1	2
4054	71	1	0	0	0	0	1	1	1	1	1	0	0	0	0	1	4
4059	84	1	0	0	0	0	1	0	0	1	0	0	0	0	0	3	4
4061	77	2	0	0	0	0	3	1	0	0	1	0	1	0	0	3	4
4062	79	2	0	1	0	0	#NULL!	#NULL!	#NULL!	#NULL!	0	0	#NULL!	0	0	4	3
4063	74	2	1	0	0	1	1	0	0	0	1	1	0	0	0	1	2
4064	72	1	0	0	0	#NULL!	1	1	0	1	1	1	0	0	1	2	4
4065	76	2	0	#NULL!	0	0	1	2	0	0	1	1	0	0	0	1	2
4066	77	2	0	1	0	#NULL!	1	#NULL!	0	0	1	1	0	0	1	3	4
4067	84	2	0	0	1	1	2	1	0	0	0	0	0	0	0	2	1
4068	79	1	0	0	1	1	1	1	1	1	0	0	0	1	0	1	4
4070	72	2	1	0	1	1	1	#NULL!	0	0	0	0	0	0	0	2	2
4072	66	2	0	0	0	1	1	0	0	0	0	0	0	0	0	2	2
4073	67	1	0	0	0	1	1	1	1	0	1	0	0	0	0	2	4
4075	70	2	0	1	0	1	1	0	1	0	1	1	0	0	0	1	3
4076	76	2	0	1	0	0	1	0	0	0	1	1	0	0	0	4	3
4078	75	1	0	0	0	1	1	2	0	1	0	1	0	0	0	1	4
4079	71	2	0	0	0	1	1	1	0	0	0	0	0	0	0	2	3
4080	72	1	1	0	0	0	1	1	1	1	0	0	0	1	0	1	4
4081	66	2	0	0	0	0	1	0	0	0	0	0	0	0	1	2	4
4082	75	1	0	0	0	1	3	2	0	1	1	0	1	0	0	1	4
4083	74	2	0	0	0	1	#NULL!	0	0	0	1	1	0	0	0	2	1
4086	66	2	0	0	0	0	1	0	0	0	0	1	0	0	0	2	2
4087	72	1	0	0	0	0	2	0	0	0	0	0	0	0	0	2	4
4088	72	2	0	0	0	#NULL!	1	1	0	0	1	1	0	0	0	2	3
4093	69	2	0	0	1	1	1	0	0	0	1	0	0	0	0	2	4
4094	72	2	0	1	0	0	1	0	0	0	0	1	0	0	0	2	2
4097	74	1	1	0	0	1	1	1	1	1	1	0	0	0	0	1	4
4098	69	2	0	0	0	0	1	0	0	0	1	0	1	0	0	2	2
4105	68	1	0	0	0	1	1	0	1	1	1	0	0	0	1	2	4
4107	75	1	0	0	0	1	1	0	0	0	0	0	0	0	1	2	4
4112	68	2	0	1	0	1	1	1	0	0	0	0	0	0	0	3	3
4114	78	1	0	0	0	0	1	1	1	0	0	0	0	0	0	2	4
4116	74	2	1	0	0	1	1	0	0	0	0	0	0	0	0	3	1

4119	66	1	0	1	0	0	3	1	0	0	0	0	0	0	0	1	4
4120	66	2	0	#NULL!	0	#NULL!	1	#NULL!	#NULL!	#NULL!	0	0	#NULL!	0	1	3	4
4122	65	1	0	0	0	0	3	2	0	1	0	0	0	0	0	1	4
4123	70	1	0	0	0	0	1	1	0	0	0	0	0	0	1	1	4
4124	68	2	0	0	0	0	3	0	0	0	0	0	0	0	0	3	2
4126	72	2	0	0	0	1	#NULL!	1	1	0	0	1	1	0	0	2	2
4127	69	1	1	0	0	1	1	1	0	0	0	0	0	0	0	1	4
4130	77	1	0	0	0	0	3	1	0	0	1	0	1	0	0	1	4
4131	76	2	0	0	0	0	1	0	0	0	1	0	0	0	1	2	4
4132	82	1	0	1	0	0	3	2	0	0	0	1	1	0	0	1	4
4133	71	1	0	0	0	#NULL!	3	1	0	1	1	1	0	0	0	1	4
4134	67	2	0	0	0	1	1	0	0	0	0	1	0	0	0	2	4
4135	71	1	0	0	0	0	3	1	0	1	1	0	0	0	0	2	4
4136	67	2	0	1	0	1	1	#NULL!	0	0	0	0	1	0	0	2	2
4139	77	1	1	0	0	0	1	1	0	1	1	1	0	0	0	3	4
4142	78	1	0	0	1	#NULL!	1	1	1	1	1	1	0	0	0	1	4
4145	66	2	0	0	1	1	1	#NULL!	1	0	0	0	0	0	0	3	2
4146	76	1	0	1	0	0	1	#NULL!	#NULL!	#NULL!	1	0	#NULL!	0	0	1	4
4147	74	2	1	0	0	0	1	1	0	0	1	1	0	0	0	4	1
4148	83	2	1	1	0	0	1	2	0	0	1	0	0	0	0	2	4
4149	75	1	0	0	0	1	1	0	0	0	0	0	0	0	0	2	4
4150	72	2	0	0	0	1	1	2	0	0	0	0	0	0	0	2	4
4156	65	2	0	0	0	0	1	0	0	0	1	0	0	0	0	2	3
4157	83	1	0	0	0	1	1	1	0	1	1	1	0	0	0	2	4
4158	77	2	1	0	0	1	1	#NULL!	0	0	1	0	0	0	0	2	4
4159	81	1	0	0	0	1	1	2	1	1	0	0	0	0	0	2	4
4160	72	2	0	0	0	1	1	1	0	0	0	0	0	0	0	1	3
4162	75	2	0	0	1	0	3	2	0	#NULL!	1	0	0	0	0	3	3
4163	74	2	0	1	1	1	1	0	0	0	0	0	1	0	0	4	3
4166	72	1	0	0	0	0	1	1	0	0	0	0	0	0	0	1	4
4167	69	2	0	0	0	0	1	0	0	#NULL!	1	1	0	0	0	4	1
4172	72	1	0	0	0	0	1	1	0	1	0	0	0	0	1	2	4
4173	70	2	0	0	0	0	1	1	0	0	1	0	0	0	0	3	4
4176	72	1	0	0	0	1	1	0	1	1	0	0	0	0	0	2	4
4177	70	2	0	1	0	1	3	1	0	0	0	0	0	0	0	3	4
4178	74	1	0	1	1	#NULL!	1	2	0	1	0	0	0	0	0	2	4
4180	75	2	0	0	1	0	#NULL!	1	1	0	0	0	0	0	0	3	4
4181	71	1	0	0	0	0	1	0	0	0	0	0	0	0	1	2	1
4185	72	1	0	0	0	1	1	2	0	1	0	1	1	0	0	3	4

4188	67	2	0	0	0	0	1	1	0	0	0	0	0	0	0	3	1
4189	65	1	0	0	1	1	1	0	1	1	0	1	0	1	1	3	4
4190	75	2	0	1	0	1	3	0	0	0	0	1	1	0	0	2	3
4191	68	2	0	1	0	1	1	1	0	0	0	0	0	0	0	3	3
4193	67	2	0	0	0	#NULL!	1	1	0	0	0	0	0	0	0	2	4
4201	65	1	0	0	0	1	1	1	1	1	0	0	0	0	0	2	4
4204	77	1	0	0	0	1	1	0	0	1	1	0	0	0	0	1	4
4208	69	1	0	0	0	0	1	1	1	1	1	0	0	0	0	2	4
4209	65	2	0	0	0	0	2	0	0	0	0	0	0	0	1	3	3
4210	72	2	0	#NULL!	0	#NULL!	1	#NULL!	#NULL!	#NULL!	1	1	#NULL!	0	0	3	4
4211	81	1	0	0	1	1	2	#NULL!	#NULL!	#NULL!	0	0	#NULL!	0	0	3	4
4212	75	2	0	0	0	1	1	0	0	0	1	1	#NULL!	0	0	3	4
4213	71	2	0	1	0	1	2	2	0	0	1	0	0	0	0	2	1
4216	68	1	0	0	1	1	1	1	0	1	0	0	0	0	1	2	4
4217	72	2	0	0	1	1	#NULL!	1	0	0	1	0	0	0	0	2	4
4221	86	1	1	#NULL!	1	#NULL!	3	1	0	1	1	1	0	0	0	3	4
4222	80	2	1	1	1	0	3	0	0	1	0	0	0	0	0	2	4
4225	80	1	0	0	0	1	1	2	0	1	0	0	0	0	0	2	4
4226	76	2	0	0	0	1	1	#NULL!	#NULL!	#NULL!	0	0	#NULL!	0	0	2	4
4229	87	2	0	1	0	1	1	1	0	0	1	0	0	0	0	4	3
4230	74	1	0	0	0	0	1	#NULL!	#NULL!	#NULL!	0	0	#NULL!	0	0	1	4
4231	70	2	1	0	0	0	3	#NULL!	0	0	1	0	1	0	0	3	4
4233	65	2	0	0	1	1	2	0	0	1	0	0	0	0	1	3	4
4236	76	2	0	0	0	1	1	0	0	0	0	0	1	1	0	4	1
4238	79	1	1	0	1	1	1	0	0	1	1	1	0	0	0	3	4
4242	74	2	1	0	1	1	1	0	0	1	0	1	0	0	0	4	1
4248	74	1	0	0	0	1	1	1	0	0	0	0	0	0	1	3	4
4249	72	2	0	0	1	#NULL!	1	1	0	0	1	0	0	0	0	3	3
4251	67	1	0	0	0	1	1	1	0	1	0	0	0	0	1	2	4
4252	79	2	1	0	0	1	3	2	0	0	0	0	1	0	0	2	3
4253	76	1	1	0	0	1	3	2	0	1	1	0	0	1	0	3	4
4255	70	1	0	0	0	0	3	#NULL!	0	1	0	0	0	0	0	1	4
4256	67	2	0	0	0	1	2	0	0	0	0	0	0	0	0	2	1
4257	68	1	0	0	0	1	1	0	0	1	0	0	0	0	1	2	4
4259	75	2	0	0	0	0	3	1	0	0	1	1	1	0	0	1	4
4260	66	1	0	0	0	0	1	1	1	1	1	0	0	0	0	2	4
4263	76	1	0	0	1	1	1	1	0	1	0	0	0	0	0	3	2
4264	73	2	0	0	0	1	1	1	0	1	0	0	0	0	0	2	1
4265	73	1	0	0	0	0	2	1	0	0	1	0	0	0	0	1	4

4266	69	2	0	0	1	0	1	0	0	0	1	1	0	0	0	3	3
4267	68	1	0	0	1	1	1	0	1	1	1	0	0	1	1	3	4
4268	66	1	0	0	0	0	1	0	0	0	1	0	0	0	0	1	4
4269	75	1	0	0	0	0	1	0	1	1	1	1	0	0	0	1	4
4277	71	2	0	0	1	1	3	1	0	0	0	0	1	1	0	3	4
4278	66	2	0	1	0	1	3	0	0	1	0	1	0	0	0	1	4
4281	77	2	0	1	1	1	1	0	0	0	1	0	0	0	0	1	4
4282	73	1	0	0	0	0	1	1	1	0	0	0	0	1	0	4	4
4283	72	2	0	0	0	0	1	0	0	0	0	0	0	0	0	2	3
4285	70	2	0	0	0	#NULL!	1	#NULL!	0	0	1	0	0	0	0	2	2
4286	71	2	0	1	1	1	1	0	0	0	1	1	0	0	0	3	4
4287	82	1	1	0	0	0	1	0	0	0	0	0	0	0	0	3	4
4288	79	2	0	0	0	0	1	1	0	0	0	0	0	0	0	3	2
4289	71	1	0	0	1	1	3	0	1	0	1	1	0	0	0	3	4
4290	67	2	0	0	0	1	1	0	0	0	0	0	0	0	0	2	1
4291	83	2	1	0	1	1	1	1	0	0	0	0	1	1	0	1	4
4292	83	1	1	0	0	1	1	#NULL!	0	0	0	0	0	0	0	1	1
4294	78	2	0	1	1	1	1	1	0	0	0	0	1	0	0	4	1
4295	72	2	0	1	1	1	1	1	0	1	1	0	0	0	1	3	4
4296	72	2	0	1	1	1	3	0	0	0	1	0	0	0	0	2	4
4297	74	1	0	0	1	1	1	0	#NULL!	1	0	1	0	0	0	3	4
4298	68	2	0	0	1	1	1	1	0	0	0	0	0	0	0	3	4
4300	69	1	0	0	0	1	1	1	0	0	1	1	0	0	0	3	4
4301	74	1	0	0	1	1	3	1	0	0	1	0	0	0	1	3	2
4302	71	2	0	0	0	1	1	0	0	0	1	0	0	0	0	2	4
4303	67	1	0	0	0	#NULL!	1	1	1	1	1	1	0	0	1	1	4
4307	72	2	0	0	1	1	1	0	0	0	0	1	0	0	0	4	3
4309	73	2	1	0	0	1	1	1	0	0	1	0	1	0	0	1	4
4310	78	1	1	1	0	1	1	0	0	1	0	0	0	0	0	1	4
4311	69	1	1	0	0	1	3	1	1	1	0	0	0	0	1	2	4
4312	73	1	0	0	0	0	1	0	1	1	1	1	0	0	0	1	4
4313	65	2	0	0	0	1	1	0	0	0	1	1	0	0	0	2	4
4314	65	1	0	0	0	1	1	0	1	1	0	0	0	0	1	2	4
4315	65	1	0	0	0	0	1	0	0	1	0	0	0	1	1	3	2
4317	69	1	0	0	0	0	1	1	0	1	1	1	0	0	0	1	4
4318	65	2	0	0	0	0	3	1	0	0	1	1	0	0	0	2	2
4319	65	2	0	1	0	1	1	0	0	0	1	0	0	0	0	1	4
4322	72	1	0	0	0	1	1	#NULL!	#NULL!	#NULL!	0	1	#NULL!	0	0	2	1
4328	73	1	0	0	0	0	1	1	1	1	1	0	0	0	0	1	4

4329	68	2	1	0	1	1	1	0	0	0	0	0	0	0	0	4	1
4330	78	1	0	0	0	0	1	0	0	1	0	0	0	0	0	1	4
4331	71	2	0	0	0	0	1	1	0	0	0	0	0	0	0	4	1
4332	67	1	0	0	0	0	3	1	1	0	1	0	0	0	0	1	4
4334	68	1	0	0	0	1	3	2	0	1	0	0	0	0	0	3	4
4335	71	1	0	0	0	1	1	0	1	0	0	0	1	0	0	2	4
4336	78	1	0	0	0	1	1	0	0	1	0	1	1	0	0	2	1
4337	70	2	0	0	0	1	1	1	0	0	1	0	0	0	0	2	1
4338	74	1	1	0	0	1	1	1	1	1	0	0	1	0	0	3	4
4339	74	2	0	0	0	#NULL!	1	0	0	0	0	0	0	0	0	2	1
4347	78	1	0	0	0	0	1	0	1	1	1	0	0	0	0	1	4
4349	74	2	0	1	0	1	1	1	0	0	0	1	0	0	0	1	4
4350	72	1	0	0	0	1	1	1	0	1	1	1	0	0	0	3	4
4351	68	2	0	1	#NULL!	1	#NULL!	#NULL!	0	0	0	0	0	0	0	4	3
4356	68	1	0	0	0	1	1	2	0	0	0	0	0	0	1	1	4
4360	70	2	0	0	0	0	1	0	0	0	1	1	0	0	0	1	2
4361	85	1	0	0	1	#NULL!	3	#NULL!	#NULL!	#NULL!	0	0	#NULL!	#NULL!	1	3	4
4369	87	1	1	1	1	0	1	0	0	1	0	0	0	0	0	1	4
4379	85	2	1	1	0	0	3	1	0	0	0	0	1	0	0	2	1
4382	77	1	1	0	0	1	1	1	0	0	0	0	0	0	1	2	4
4383	71	2	0	0	0	1	1	1	0	0	1	0	0	0	1	3	3
4387	69	1	0	0	0	1	3	2	1	1	1	0	0	0	0	2	4
4388	66	2	0	0	0	1	1	0	0	0	1	1	1	0	0	2	2
4389	70	2	0	0	1	1	#NULL!	1	0	1	1	0	0	0	0	1	3
4391	66	2	0	1	1	1	1	2	1	1	0	0	0	0	1	2	4
4392	66	1	0	0	0	0	3	2	1	1	0	1	0	0	0	3	4
4394	68	2	0	1	1	1	3	1	0	0	0	0	0	0	1	3	4
4395	70	1	0	0	0	1	1	1	1	1	1	0	1	0	0	2	4
4396	66	2	0	0	0	0	1	#NULL!	0	0	0	0	0	0	0	4	1
4397	71	2	1	0	1	0	1	1	0	0	0	0	1	0	0	3	4
4398	68	2	0	1	0	0	1	0	0	0	1	1	0	0	0	2	4
4400	80	2	0	1	0	1	1	1	0	0	1	1	0	0	0	2	2
4401	70	1	0	0	0	1	1	1	1	1	1	1	0	0	0	2	4
4407	71	1	0	0	0	0	1	0	0	0	1	0	0	0	0	1	4
4409	65	2	0	0	0	0	3	0	0	0	1	0	0	0	0	2	4
4411	71	2	0	0	0	1	3	0	0	0	0	0	0	0	1	2	4
4412	84	1	1	0	0	#NULL!	#NULL!	1	0	1	0	0	1	0	0	4	1
4414	74	1	0	0	0	0	1	0	1	1	1	1	0	1	0	1	4
4415	68	2	0	0	0	0	1	0	0	0	1	0	0	0	0	2	2

4416	67	2	0	0	0	0	1	0	0	0	1	0	0	0	0	1	2
4418	72	2	0	0	0	0	1	1	1	0	1	0	0	0	1	2	4
4419	69	1	0	0	0	1	1	1	0	0	1	0	0	0	0	1	4
4422	72	2	0	0	1	1	1	1	0	0	1	0	0	0	0	3	4
4423	86	1	1	0	0	1	1	0	0	0	0	0	0	1	0	2	4
4424	82	2	1	0	0	#NULL!	1	0	0	0	1	0	0	0	0	2	1
4426	72	1	1	0	0	0	3	0	0	0	1	0	0	0	1	1	4
4427	71	1	0	0	0	1	1	1	0	1	1	1	0	0	0	1	4
4428	65	2	0	0	0	1	#NULL!	1	0	0	0	1	0	0	0	1	3
4430	84	2	0	1	1	0	1	1	0	0	1	1	0	0	0	2	4
4433	69	2	0	1	0	0	1	0	0	0	0	0	0	0	0	2	3
4434	67	1	0	0	0	0	2	0	0	1	1	0	1	0	0	1	4
4436	65	1	0	0	0	0	1	#NULL!	1	1	1	0	0	0	1	1	4
4438	81	2	0	0	0	0	1	#NULL!	#NULL!	#NULL!	0	0	#NULL!	0	0	2	4
4440	73	2	0	0	0	0	1	0	0	0	0	0	0	0	1	1	4
4441	72	2	0	1	0	0	1	1	0	0	0	0	0	0	1	2	4
4442	70	1	0	0	0	1	1	1	1	0	1	0	0	0	0	1	4
4443	67	2	0	0	0	1	1	0	0	0	1	1	0	0	0	1	4
4444	69	1	0	0	1	0	1	1	1	1	0	0	0	0	0	3	3
4445	69	2	0	1	1	1	1	1	0	0	0	1	0	0	1	1	3
4448	73	2	0	0	0	#NULL!	2	#NULL!	0	0	1	0	1	0	0	3	3
4452	75	1	1	0	0	1	1	0	0	0	1	0	0	0	0	2	4
4453	66	2	0	0	0	1	1	0	0	0	1	0	0	0	0	2	3
4456	77	1	0	0	0	0	1	#NULL!	#NULL!	#NULL!	1	0	#NULL!	0	1	1	4
4457	75	2	0	0	0	1	1	0	#NULL!	0	1	1	0	0	1	3	3
4459	72	2	0	0	0	0	1	1	0	0	0	0	0	0	0	1	4
4461	67	2	0	1	0	0	1	0	0	0	1	0	0	0	0	4	1
4464	65	2	0	1	0	1	1	0	0	0	0	1	0	0	0	2	3
4468	72	1	0	0	0	0	1	0	1	1	1	0	0	0	0	3	4
4469	77	1	0	0	1	0	1	1	1	0	0	0	0	0	0	3	4
4470	71	2	0	0	0	0	1	0	0	0	0	0	0	0	0	1	3
4473	67	1	1	0	0	#NULL!	1	0	0	1	0	0	0	0	1	2	4
4474	67	1	0	0	0	0	1	0	1	1	1	0	0	0	1	1	4
4475	71	2	0	1	1	0	1	1	0	0	0	0	0	0	0	2	4
4478	72	2	0	0	1	1	1	1	0	0	1	0	0	0	0	3	1
4479	72	1	0	0	1	1	1	#NULL!	1	1	0	0	0	0	0	3	4
4480	68	2	0	0	1	1	1	1	0	0	1	0	0	0	0	3	2
4481	67	1	0	0	0	1	1	1	1	1	1	0	0	0	0	1	4
4482	76	2	0	1	1	1	1	0	0	0	1	0	0	0	0	3	4

4483	74	1	0	0	0	1	1	2	0	0	0	1	1	0	0	2	4
4484	73	2	0	0	1	1	1	1	0	0	1	0	0	0	0	2	2
4490	71	1	0	0	0	#NULL!	1	0	1	1	0	1	0	0	1	4	4
4491	69	2	0	0	0	1	1	1	0	0	1	0	0	0	0	2	1
4494	67	1	0	0	0	0	1	0	1	1	1	0	0	1	1	1	4
4495	66	2	0	0	0	0	1	2	0	0	1	0	0	0	0	2	3
4496	78	2	0	1	0	1	1	#NULL!	0	0	0	0	#NULL!	0	0	3	3
4501	70	2	1	1	0	1	1	1	0	0	1	0	0	0	1	2	4
4504	66	2	0	0	0	1	1	0	0	0	0	0	1	0	0	1	3
4506	70	2	0	0	0	1	3	1	0	0	0	0	1	0	0	2	1
4507	79	2	0	1	1	0	1	#NULL!	#NULL!	#NULL!	0	0	#NULL!	0	0	2	1
4508	76	1	0	1	0	1	3	#NULL!	#NULL!	#NULL!	1	1	#NULL!	0	1	2	4
4509	73	2	0	0	0	1	3	1	0	0	0	0	0	0	0	2	3
4510	65	2	0	0	0	1	3	0	0	0	0	0	0	0	0	2	4
4513	66	2	0	0	0	1	1	0	0	0	0	0	0	0	0	2	1
4514	66	1	0	0	0	1	1	#NULL!	1	1	0	1	0	0	0	3	4
4515	65	2	0	0	0	1	1	2	0	0	0	0	0	0	0	3	4
4517	76	1	0	0	1	0	3	0	0	1	0	0	0	1	0	3	2
4518	66	2	0	0	0	0	1	0	0	0	0	0	0	0	0	2	1
4520	73	1	1	0	1	0	1	1	0	1	1	1	0	0	1	2	4
4521	69	2	0	0	0	0	1	1	0	0	1	1	0	0	0	3	2
4531	67	1	0	0	0	0	1	1	0	1	0	0	0	0	0	2	4
4532	70	1	0	0	0	0	3	1	1	1	1	0	0	0	0	1	4
4534	74	1	0	0	0	1	1	2	0	0	0	1	0	0	0	2	4
4536	77	1	1	0	0	0	3	1	1	1	0	0	0	0	0	2	4
4537	77	2	0	0	0	#NULL!	1	1	0	1	0	0	1	0	0	2	2
4539	75	1	0	0	0	0	1	1	1	1	1	1	0	0	0	2	4
4540	69	2	0	0	0	0	1	0	0	0	1	0	0	0	0	2	3
4542	75	1	1	0	0	1	1	1	1	1	0	0	1	0	0	3	4
4543	70	2	0	0	0	1	#NULL!	0	0	0	0	0	0	0	0	2	1
4544	68	1	0	0	0	0	1	0	1	1	0	0	0	0	0	2	4
4550	80	1	1	0	0	0	1	2	1	1	0	1	0	0	1	2	4
4551	78	2	0	0	0	0	1	2	0	0	0	0	0	0	0	1	2
4553	66	2	0	1	0	1	1	0	0	0	0	0	1	0	0	2	4
4556	71	2	0	0	0	1	1	#NULL!	0	0	1	0	0	0	0	1	2
4558	66	2	0	0	0	0	1	1	0	0	0	0	0	0	1	1	4
4560	68	1	0	0	0	0	3	0	1	0	1	0	0	0	1	4	1
4561	65	2	0	0	0	0	1	0	0	0	1	0	0	0	1	1	1
4563	65	2	0	0	0	1	3	2	0	0	0	0	0	0	0	1	3

4565	65	2	0	1	0	1	2	0	1	0	0	0	0	0	0	2	2
4568	71	2	0	0	0	1	1	1	0	0	0	0	0	0	0	2	2
4569	71	1	0	0	1	0	1	1	1	1	0	0	0	0	0	3	4
4572	78	2	1	0	1	0	1	1	0	0	1	1	0	0	0	3	2
4574	72	2	0	0	0	1	3	1	0	1	1	1	0	0	0	4	1
4579	71	2	0	0	0	1	2	0	0	0	0	1	0	0	0	2	2
4588	70	2	0	0	1	0	1	1	#NULL!	0	0	0	0	0	0	2	2
4591	78	2	0	1	1	#NULL!	1	1	0	0	1	1	0	0	0	2	3
4592	76	1	1	0	0	1	1	#NULL!	0	0	1	0	0	0	1	1	4
4596	79	1	0	0	0	1	1	0	1	1	1	1	0	0	0	4	1
4597	72	2	0	0	0	1	1	0	0	0	1	1	0	0	0	3	4
4599	70	2	1	0	0	1	1	1	0	0	0	1	0	0	0	3	2
4600	73	2	0	0	0	0	1	0	0	0	1	0	0	0	0	2	2
4601	73	1	0	0	0	0	1	0	0	1	1	1	0	0	0	1	4
4603	79	1	0	0	0	0	1	1	1	1	1	1	0	0	1	1	4
4604	72	2	0	1	0	0	1	0	1	0	1	0	0	0	0	4	1
4605	67	2	0	0	0	1	1	1	0	0	0	0	0	0	0	2	2
4607	74	1	0	0	0	#NULL!	1	1	0	1	0	0	0	0	1	1	1
4608	67	2	0	0	1	1	3	0	0	0	0	0	0	0	1	1	4
4609	78	2	0	1	0	0	2	0	0	0	0	1	0	0	0	2	3
4611	70	2	0	1	0	0	1	0	0	0	1	0	0	0	0	2	4
4615	70	2	0	1	0	1	2	0	0	0	1	0	0	0	1	2	3
4618	69	2	0	0	0	1	1	0	0	1	1	0	0	0	0	1	3
4619	65	1	0	0	0	1	1	2	1	1	0	0	0	0	0	3	4
4622	70	1	0	0	0	1	1	0	0	1	0	0	0	0	1	1	4
4623	67	2	0	0	0	1	1	1	0	0	0	0	0	0	1	4	4
4624	67	2	0	0	0	0	1	0	0	1	0	0	0	0	0	3	4
4625	65	1	0	0	0	0	3	#NULL!	#NULL!	#NULL!	1	0	#NULL!	0	0	1	4
4626	77	1	0	0	0	1	1	1	0	0	0	0	0	0	0	3	4
4628	83	2	1	1	1	1	1	1	0	0	0	0	1	0	0	2	4
4631	67	1	0	0	0	1	3	0	1	1	0	0	0	0	1	3	4
4632	67	1	0	0	1	0	3	1	1	1	0	0	0	0	0	1	4
4633	82	1	1	0	0	1	1	1	0	1	0	0	1	0	0	1	4
4636	67	1	0	0	0	0	1	0	1	1	0	0	0	0	0	1	4
4639	66	1	0	0	0	0	1	0	1	1	1	0	0	0	0	2	4
4640	76	1	0	0	0	0	1	1	0	0	0	1	0	0	1	3	4
4642	70	1	0	0	0	0	1	1	1	1	1	0	0	0	0	2	4
4643	66	2	0	0	0	1	1	0	0	0	1	1	0	0	1	2	2
4644	71	2	0	1	0	0	1	1	0	0	1	1	0	0	1	1	1

4646	77	1	0	0	1	0	1	1	0	0	0	0	0	0	0	3	4
4647	71	2	0	0	1	0	1	1	0	0	1	0	0	0	0	3	1
4648	65	2	0	0	0	1	3	1	0	0	0	0	0	0	0	2	1
4651	68	2	0	0	0	#NULL!	#NULL!	0	0	0	1	0	0	0	0	4	1
4653	67	1	0	0	0	0	3	2	1	1	0	0	0	0	0	1	4
4654	66	2	0	0	0	1	1	0	0	0	1	0	1	0	0	2	2
4657	70	1	0	0	0	1	1	2	0	1	0	0	0	0	0	2	4
4658	68	2	0	0	1	1	1	0	0	0	0	0	0	0	0	3	3
4661	65	2	0	1	0	#NULL!	1	0	#NULL!	0	0	0	0	0	1	2	3
4662	68	1	0	0	1	0	1	0	0	1	0	0	0	1	1	2	4
4668	66	1	0	0	1	1	1	0	0	1	0	0	1	0	0	1	4
4669	68	2	0	1	0	1	1	1	0	0	0	1	0	0	0	2	1
4670	67	1	0	0	0	1	3	0	1	0	1	0	0	0	1	3	4
4671	65	1	0	0	0	1	1	0	1	1	0	0	0	0	0	1	4
4672	70	2	0	1	0	1	3	1	0	0	0	0	#NULL!	0	1	3	4
4674	78	2	0	0	#NULL!	1	1	1	0	0	0	0	0	0	0	4	2
4678	70	1	1	0	0	0	1	1	0	1	1	0	1	0	0	1	4
4679	68	2	0	0	0	0	3	0	0	0	1	0	0	0	0	2	1
4680	72	1	0	0	0	1	3	1	1	1	1	0	0	0	0	1	4
4681	71	2	0	0	0	1	1	1	0	0	0	0	0	0	0	3	2
4682	70	1	0	1	0	0	1	1	1	1	1	1	0	0	0	1	4
4684	77	2	0	1	0	0	3	2	#NULL!	#NULL!	0	1	#NULL!	0	0	2	1
4686	79	1	0	0	1	1	1	0	1	0	0	0	0	0	1	3	4
4687	75	2	0	0	1	#NULL!	1	#NULL!	0	0	0	0	0	0	1	1	4
4688	72	1	0	0	0	1	1	0	0	1	1	0	0	0	1	3	4
4689	71	2	0	1	1	0	1	1	0	0	0	0	0	0	1	2	4
4692	67	1	0	0	0	1	1	1	0	1	0	0	0	0	0	3	4
4693	80	1	0	0	0	#NULL!	3	1	0	1	0	0	1	0	0	2	4
4694	74	2	0	0	0	#NULL!	3	1	0	0	0	0	0	0	0	2	3
4698	80	1	0	0	1	1	1	1	0	1	1	0	0	0	0	3	4
4699	76	2	0	0	1	1	3	1	0	0	0	0	0	0	0	4	1
4701	80	1	1	0	0	0	1	2	0	1	0	0	0	1	0	1	4
4703	79	2	0	1	0	0	1	0	0	0	1	0	0	0	1	1	4
4705	68	2	0	0	1	1	1	1	0	0	0	0	0	0	1	3	4
4706	79	2	0	1	1	#NULL!	3	0	0	0	1	0	0	0	0	3	1
4708	70	1	0	0	0	0	1	1	1	1	0	0	0	0	1	1	4
4709	67	2	0	0	0	0	1	0	0	0	0	0	0	0	0	1	4
4710	73	2	1	1	0	1	1	0	0	0	0	0	1	0	0	4	1
4716	68	2	0	0	0	0	2	1	#NULL!	0	1	0	0	0	0	2	3

4720	74	1	0	1	0	0	1	1	1	0	0	0	1	0	0	2	4
4723	66	1	0	0	0	1	3	1	1	0	0	0	1	0	1	3	4
4725	70	1	0	0	1	1	1	1	0	1	0	0	1	0	0	2	4
4726	66	2	0	0	0	1	1	1	0	0	0	0	1	0	0	2	2
4727	83	2	0	1	0	0	1	1	0	0	0	0	0	0	1	2	4
4731	90	2	0	1	0	0	3	1	0	0	0	0	0	0	0	2	1
4733	74	2	1	0	1	0	1	0	0	0	1	1	0	0	0	2	1
4736	67	2	1	0	1	1	2	1	0	1	0	0	1	0	1	2	4
4737	66	1	0	0	1	1	3	2	1	1	0	0	0	0	0	3	4
4743	78	1	0	0	1	1	3	1	1	1	0	0	0	1	0	3	4
4744	73	2	0	0	1	1	#NULL!	0	0	0	1	0	1	0	1	3	4
4745	68	1	0	0	0	0	1	1	0	0	1	0	0	0	0	1	4
4749	68	2	0	0	0	#NULL!	1	#NULL!	0	1	0	0	0	0	0	1	3
4750	77	2	0	1	1	0	3	1	0	0	1	0	1	0	0	3	3
4751	70	1	0	0	0	1	1	1	0	1	1	0	0	0	1	4	4
4756	65	2	0	0	0	0	1	0	0	0	0	0	0	0	0	3	4
4759	76	1	0	1	1	1	1	1	0	1	0	1	0	0	1	1	4
4760	73	1	0	0	0	1	1	1	1	1	1	0	0	0	0	1	4
4762	69	1	0	0	1	1	1	1	1	1	0	0	0	1	1	2	4
4763	68	2	0	0	0	1	3	0	0	1	0	0	0	0	0	3	2
4764	73	1	0	0	0	1	1	2	1	1	1	0	1	0	0	1	4
4768	67	1	0	0	0	1	1	0	1	1	0	0	0	0	1	3	4
4769	66	2	0	0	0	1	1	1	0	0	0	0	1	0	0	1	2
4774	74	2	0	0	0	0	2	1	0	0	0	1	0	0	0	2	2
4778	72	2	1	0	0	1	1	0	0	0	1	1	0	0	0	2	2
4780	70	2	0	1	1	1	2	0	0	1	0	0	0	0	0	2	4
4785	69	1	0	0	0	1	1	0	1	0	0	0	0	0	0	1	4
4787	73	1	0	0	0	0	3	1	1	1	1	0	0	0	0	1	4
4789	75	2	0	1	1	0	1	0	0	0	0	0	1	0	0	2	4
4791	74	2	0	0	0	#NULL!	3	1	0	0	0	0	1	0	0	4	1
4794	75	1	1	0	0	1	1	0	1	0	0	1	0	0	1	2	4
4795	73	2	0	0	0	1	1	0	0	0	0	0	0	0	0	3	3
4798	70	1	0	0	0	0	1	1	0	1	0	0	0	0	0	3	4
4799	66	2	0	0	0	0	3	1	0	0	0	0	0	0	0	3	4
4800	67	2	1	0	0	1	3	0	0	1	0	0	0	0	1	2	3
4802	74	2	0	0	0	#NULL!	3	1	0	0	0	1	0	0	0	1	2
4806	68	1	0	0	0	0	2	0	1	1	1	1	0	0	0	3	4
4811	77	2	0	0	1	1	1	1	0	0	0	0	0	0	0	3	3
4814	93	2	1	1	#NULL!	#NULL!	#NULL!	1	0	0	0	0	1	0	0	4	1

4815	66	1	1	0	0	1	1	1	1	1	0	0	0	0	1	2	4
4816	65	2	0	0	0	1	1	0	0	0	0	0	0	0	1	1	4
4817	67	1	0	0	0	0	3	0	1	1	0	0	0	0	0	1	4
4828	67	1	0	0	0	1	1	1	0	1	1	0	0	1	1	1	4
4829	71	2	0	0	0	1	1	#NULL!	0	0	0	0	0	0	0	2	2
4834	66	2	0	0	0	1	1	0	0	0	0	0	0	0	1	2	4
4835	68	1	1	0	0	1	1	0	0	0	0	0	0	0	0	3	4
4836	72	1	0	0	0	1	1	0	1	1	0	0	0	0	1	1	3
4838	76	2	0	1	1	#NULL!	1	1	0	0	0	0	0	0	0	3	1
4839	68	1	0	0	0	0	1	#NULL!	1	1	0	0	0	0	0	1	4
4840	66	2	0	0	0	1	3	2	0	0	0	0	0	0	0	2	1
4843	71	1	0	0	0	1	1	1	0	1	0	0	0	0	0	1	4
4845	77	1	0	0	0	1	3	0	1	1	0	0	0	1	0	2	4
4846	70	2	0	0	0	1	1	0	0	0	0	1	0	0	0	3	3
4850	72	1	0	0	0	0	3	1	0	0	0	0	1	0	1	2	4
4851	68	2	0	0	0	1	1	1	0	0	1	0	0	0	0	4	1
4852	78	2	0	1	0	1	3	#NULL!	#NULL!	#NULL!	0	0	#NULL!	0	0	4	4
4854	76	1	0	0	0	1	3	1	1	1	0	0	0	0	0	2	4
4856	73	1	0	0	0	1	3	1	0	1	0	0	0	0	0	3	4
4857	72	2	0	0	0	1	1	1	0	0	0	1	0	0	0	2	3
4858	68	1	0	0	1	1	1	1	1	1	0	1	0	0	0	3	4
4862	72	2	0	0	1	1	1	0	0	1	0	0	0	0	0	2	4
4868	68	1	0	0	0	0	1	0	0	1	0	0	1	0	1	3	4
4869	75	1	1	0	0	0	3	0	1	0	1	0	0	0	0	1	4
4872	75	1	0	0	0	1	2	1	0	0	0	0	0	1	0	3	4
4873	73	2	0	0	1	1	2	0	0	0	0	0	0	0	0	3	2
4879	67	2	0	0	0	1	2	1	0	0	0	0	0	0	0	3	4
4880	77	2	0	1	0	1	2	0	0	0	1	1	0	0	0	1	4
4882	80	1	1	0	0	1	1	0	1	1	1	0	0	1	0	1	4
4883	73	2	0	0	0	1	1	1	0	0	1	0	0	0	0	2	2
4890	74	2	0	0	0	1	1	1	0	0	1	0	0	0	0	4	1
4895	67	2	0	1	1	#NULL!	1	0	0	0	0	0	0	0	0	2	3
4896	73	2	0	0	0	#NULL!	3	1	0	0	0	0	0	0	0	1	3
4897	69	1	0	#NULL!	0	#NULL!	1	#NULL!	#NULL!	#NULL!	1	0	#NULL!	0	0	1	4
4898	66	1	0	0	0	1	3	1	1	1	0	0	0	0	1	3	4
4901	66	1	0	0	0	0	1	0	0	1	1	0	0	0	0	1	4
4902	66	1	1	0	1	1	1	0	0	0	0	0	0	0	0	2	4
4903	84	1	1	0	0	1	1	1	0	0	0	0	0	0	0	2	4
4904	80	2	0	0	0	1	1	1	0	0	0	0	0	0	0	1	3

4906	68	1	0	1	1	1	1	0	1	1	0	0	0	0	0	3	4
4912	81	2	0	1	1	1	1	1	0	0	0	0	0	0	0	2	2
4913	66	1	0	0	1	1	1	0	1	0	0	0	0	0	0	3	4
4914	79	1	1	0	0	0	1	0	0	1	0	0	0	0	0	2	4
4915	75	2	0	0	1	0	1	0	0	0	1	1	0	0	0	4	2
4916	66	1	0	0	0	1	1	1	0	0	0	0	0	0	0	2	4
4919	68	2	0	0	1	0	1	0	0	0	0	0	0	0	1	2	4
4920	85	1	0	0	0	1	1	1	0	1	0	0	1	0	0	3	4
4921	72	2	0	0	0	1	1	1	0	1	0	0	1	0	1	1	4
4922	67	1	0	0	0	1	1	0	0	1	1	1	0	0	1	2	4
4925	82	2	1	0	0	1	1	1	0	0	0	0	1	0	0	2	2
4926	76	1	0	0	0	1	1	1	0	1	0	0	1	0	0	1	4
4927	70	1	0	0	0	1	1	0	1	1	0	0	0	0	0	2	4
4929	70	2	1	1	0	#NULL!	1	0	0	0	1	0	0	0	0	2	2
4930	67	2	0	1	0	#NULL!	2	1	0	0	0	0	0	0	0	2	4
4932	74	1	0	0	0	0	1	0	1	1	0	0	0	0	0	4	1
4933	72	2	0	0	1	0	1	0	0	0	0	0	0	0	0	2	2
4934	78	2	0	1	0	0	1	1	0	0	0	0	0	0	0	1	2
4939	79	2	0	1	0	1	1	1	0	0	0	0	1	1	0	2	4
4945	70	2	0	0	1	1	1	1	0	0	0	0	0	0	1	3	4
4947	77	2	0	1	0	0	1	1	0	0	1	0	1	0	0	2	3
4950	76	1	0	0	0	0	1	0	1	0	0	0	0	0	0	1	4
4954	76	2	0	0	0	0	2	2	0	0	0	0	1	0	0	4	1
4956	70	1	0	0	1	0	1	0	1	0	0	0	0	0	0	2	4
4957	68	2	0	0	1	0	1	1	0	0	1	0	0	0	1	2	4
4959	73	2	0	1	0	1	1	0	0	0	1	1	0	0	1	2	3
4962	86	1	1	1	0	1	1	2	0	0	0	0	0	1	0	3	4
4965	68	1	0	0	0	#NULL!	2	1	1	1	0	0	0	0	1	1	4
4967	66	1	0	0	0	0	1	0	0	0	1	0	0	0	1	1	4
4972	67	1	0	0	0	0	1	1	1	1	0	0	0	0	1	2	4
4974	86	2	0	0	0	0	1	1	0	0	0	0	1	0	1	1	2
4975	74	1	0	1	0	1	3	2	0	1	0	0	0	0	0	1	4
4976	73	1	0	0	0	0	1	1	1	1	1	0	0	0	0	1	4
4977	73	2	0	0	0	1	1	0	0	0	0	0	0	0	0	1	4
4978	75	2	0	1	0	1	1	0	0	0	1	0	0	0	1	3	3
4983	86	2	0	1	0	1	1	1	0	0	0	0	1	1	0	2	1
4985	71	2	0	0	1	1	1	1	0	0	1	0	0	0	0	2	4
4987	72	1	0	0	1	1	1	1	0	1	0	0	0	0	1	2	1
4989	83	2	0	1	1	0	3	2	0	0	0	0	1	0	0	4	3

4992	76	2	0	1	0	1	#NULL!	0	0	0	0	0	0	0	0	2	4
4993	69	1	0	0	0	1	1	2	0	0	0	0	0	0	0	2	4
4996	70	2	0	0	1	1	3	1	0	0	0	0	0	0	0	4	3
4998	70	1	0	0	0	0	1	1	1	1	1	1	0	0	0	1	4
4999	76	1	0	0	0	1	1	#NULL!	1	1	0	1	0	0	0	2	4
5004	68	1	0	0	0	1	1	1	0	1	0	0	1	1	0	1	4
5005	65	2	0	0	1	1	1	#NULL!	0	0	0	0	0	0	0	3	4
5008	70	2	0	0	0	1	1	1	0	1	0	0	0	0	0	2	3
5012	74	2	0	1	0	1	1	#NULL!	0	0	0	0	#NULL!	0	0	3	4
5018	74	2	0	0	1	0	1	0	1	1	0	0	0	0	0	2	4
5019	72	1	0	0	0	0	1	0	1	1	0	0	0	0	0	2	4
5020	70	1	0	0	0	0	1	2	0	1	0	0	1	0	0	2	4
5023	66	2	0	0	1	1	1	0	0	0	0	0	1	0	1	4	2
5025	77	1	0	0	0	#NULL!	1	2	0	0	0	0	0	0	0	1	4
5026	73	2	0	0	0	0	1	0	0	0	1	0	0	0	0	3	2
5029	69	1	0	0	1	1	1	0	0	1	1	0	1	1	0	1	4
5030	72	1	0	0	0	#NULL!	1	2	0	1	1	0	0	0	0	1	4
5034	70	1	0	0	0	1	3	1	1	0	1	0	0	0	0	1	4
5036	74	2	0	0	0	#NULL!	3	1	0	0	1	0	0	0	0	2	3
5042	70	1	0	0	0	0	1	0	0	1	1	0	0	0	1	1	4
5044	71	1	1	0	0	1	1	0	1	1	1	0	0	1	0	1	4
5045	68	2	0	0	0	1	1	1	0	0	1	0	0	0	0	2	1
5051	78	1	0	1	1	0	#NULL!	1	0	1	0	0	0	0	0	1	4
5052	70	1	0	1	0	1	1	#NULL!	#NULL!	#NULL!	0	0	#NULL!	0	0	2	4
5057	81	2	0	0	1	1	3	2	0	0	0	0	1	0	0	3	4
5061	70	1	0	0	0	#NULL!	1	1	1	0	0	1	0	1	0	1	4
5064	72	2	0	0	1	1	3	1	0	0	0	0	0	0	0	3	3
5065	66	1	1	0	0	#NULL!	1	2	1	1	0	0	0	0	0	3	4
5067	72	2	0	1	0	0	3	0	0	0	0	0	0	0	0	2	4
5072	79	1	0	0	1	0	3	1	1	1	1	1	0	0	0	1	1
5073	75	2	0	0	1	0	3	0	0	0	1	1	0	0	0	3	1
5074	68	2	0	1	1	1	3	2	0	0	0	0	0	0	0	4	1
5076	70	2	0	1	1	1	2	1	0	0	0	0	0	0	0	1	3
5079	85	1	0	1	0	0	1	0	1	1	0	0	0	0	0	1	4
5081	76	2	0	0	1	1	3	1	0	0	0	0	0	0	0	2	3
5083	74	2	0	0	0	1	1	0	0	0	1	1	0	0	0	4	1
5085	82	2	0	1	0	#NULL!	1	1	0	0	0	0	1	0	0	2	4
5086	66	2	1	0	0	1	1	#NULL!	0	1	0	0	0	0	0	2	4
5088	66	1	0	0	0	1	3	1	0	1	0	0	0	0	0	1	4

5091	72	2	1	0	1	1	1	2	0	0	1	0	1	0	0	4	1
5092	72	1	0	0	1	0	1	2	1	1	0	0	0	0	0	1	4
5100	69	2	0	0	0	0	1	0	0	0	0	0	0	0	0	2	1
5101	66	2	0	1	1	0	1	#NULL!	#NULL!	#NULL!	0	0	#NULL!	0	1	3	4
5102	77	1	0	0	0	0	3	1	0	1	0	1	0	0	0	1	4
5103	69	2	0	0	1	#NULL!	1	0	0	0	1	1	0	0	0	4	1
5104	71	1	0	0	0	0	1	1	1	1	1	0	0	0	1	2	4
5105	73	2	1	1	0	1	1	0	0	0	0	0	1	0	0	1	4
5107	84	1	0	0	1	1	1	0	0	1	0	0	1	0	0	2	4
5108	76	2	1	0	1	1	1	1	0	0	0	0	1	0	0	4	1
5109	71	1	0	0	1	1	1	2	0	0	0	0	1	1	0	3	4
5110	65	2	0	0	1	0	1	0	0	0	0	0	1	0	1	2	4
5111	80	1	0	1	0	0	1	1	1	1	0	0	0	0	1	1	4
5116	66	1	0	0	1	1	1	0	0	1	1	0	0	0	1	3	4
5117	74	2	0	0	1	1	1	0	0	0	1	0	0	0	0	3	2
5118	74	1	0	0	1	1	1	1	1	1	0	0	0	0	0	1	4
5120	67	2	0	0	0	1	1	0	0	0	0	0	0	0	0	4	1
5125	71	1	0	0	0	1	1	1	0	1	1	0	0	0	1	3	4
5126	68	1	0	0	0	1	1	0	1	1	0	0	1	0	0	2	4
5127	66	2	0	0	0	0	1	1	0	0	0	1	0	0	1	1	4
5130	80	2	0	1	1	0	1	0	0	0	0	0	0	0	0	1	4
5135	67	1	0	0	0	1	1	1	1	1	1	0	0	1	0	1	4
5138	83	2	0	1	1	1	1	0	0	0	1	0	#NULL!	0	0	3	2
5143	71	2	1	0	1	#NULL!	1	1	0	0	1	0	0	0	0	3	3
5144	72	2	0	0	0	1	#NULL!	1	0	1	0	0	0	0	0	2	4
5146	67	1	0	0	0	0	#NULL!	0	0	0	1	1	0	0	1	3	4
5148	67	2	0	0	0	1	1	0	0	0	0	0	0	0	0	2	1
5152	71	1	0	1	0	1	1	1	1	1	0	0	0	0	0	3	4
5155	85	1	0	0	0	1	1	1	0	1	0	0	0	0	0	1	4
5156	82	2	0	0	0	1	1	1	0	0	0	0	0	0	0	1	4
5157	75	2	0	1	1	1	2	0	0	0	0	0	0	0	0	3	2
5159	79	1	0	0	0	0	1	0	0	1	0	0	0	0	0	1	4
5160	77	2	0	0	1	0	1	0	0	0	0	0	0	0	0	3	4
5161	65	1	0	1	0	0	1	2	1	1	1	0	0	0	1	2	4
5162	76	1	0	0	0	1	1	0	0	1	1	1	0	0	0	1	4
5165	71	1	0	0	0	1	1	0	0	1	0	1	0	0	0	1	1
5166	70	2	0	0	0	1	1	0	0	0	1	0	0	0	0	4	1
5171	77	1	0	0	1	1	1	0	1	1	0	1	0	0	0	2	4
5173	71	2	0	0	0	#NULL!	1	0	0	0	0	0	1	0	0	4	2

5176	84	2	0	0	1	1	1	1	0	0	0	0	#NULL!	0	0	4	4
5178	66	1	0	0	0	1	1	2	1	1	1	0	0	0	0	2	4
5180	75	1	0	0	1	1	1	0	0	1	0	0	0	0	0	1	4
5182	66	2	0	1	0	1	1	1	0	0	0	0	1	0	0	2	2
5185	75	2	0	0	0	1	3	1	0	0	0	0	1	0	0	2	4
5187	73	2	0	0	0	1	1	0	0	0	0	0	0	0	0	4	2
5188	74	1	0	0	0	1	1	1	0	1	0	0	0	0	0	1	4
5189	71	2	0	0	0	1	1	1	0	0	0	1	0	0	0	4	1
5190	69	1	0	0	1	1	3	1	1	1	0	1	0	0	0	1	4
5192	67	1	0	0	0	1	1	0	1	1	0	0	0	0	0	2	4
5193	66	2	0	0	0	1	1	1	0	0	0	0	1	0	0	3	2
5195	84	2	1	1	0	1	1	0	0	0	1	1	1	0	0	4	1
5201	73	1	0	0	1	1	3	1	0	0	1	0	0	0	0	3	4
5202	69	2	1	0	1	1	1	0	0	0	0	0	0	0	0	3	4
5207	78	1	0	1	0	1	1	1	0	1	1	0	1	0	0	3	4
5209	66	1	0	0	0	0	1	1	1	1	1	0	0	0	1	3	2
5211	73	1	0	0	1	0	1	0	1	0	0	0	0	0	0	2	4
5212	70	2	0	0	0	0	3	1	0	0	0	0	0	0	0	3	2
5213	72	1	0	0	0	#NULL!	1	1	0	1	0	0	0	1	0	2	4
5215	65	1	1	0	0	#NULL!	1	#NULL!	0	1	1	0	0	1	1	1	4
5216	72	1	0	0	0	0	3	2	1	1	0	1	0	1	1	1	4
5217	68	2	0	0	0	0	3	0	0	0	1	0	0	0	1	2	1
5220	79	2	0	0	0	0	1	1	0	0	0	0	0	0	0	2	4
5222	66	1	0	1	0	0	1	1	0	0	0	1	0	0	0	1	4
5223	73	2	0	0	1	1	1	1	0	0	1	0	0	0	0	3	4
5224	72	1	0	0	1	1	3	0	1	0	0	0	0	0	1	3	4
5229	66	2	0	1	0	0	2	0	0	1	0	0	0	0	1	2	4
5231	73	2	0	1	0	0	1	1	0	1	0	0	0	0	1	2	3
5234	87	1	0	0	0	1	3	2	0	1	0	0	0	0	0	3	4
5235	73	1	1	0	0	0	3	#NULL!	#NULL!	#NULL!	0	0	#NULL!	0	0	1	4
5237	89	2	0	1	0	#NULL!	1	1	0	0	0	0	0	0	0	4	1
5238	66	1	0	0	0	#NULL!	1	0	0	0	1	0	1	1	0	2	4
5240	70	2	0	0	0	0	3	0	0	0	0	0	0	0	0	2	2
5241	79	2	0	#NULL!	0	#NULL!	2	0	0	0	0	1	0	0	0	3	1
5242	90	2	0	#NULL!	0	#NULL!	1	#NULL!	#NULL!	#NULL!	0	0	#NULL!	0	0	2	1
5243	77	2	0	1	1	1	1	0	0	0	1	0	0	0	0	2	1
5245	66	1	0	0	1	1	1	0	1	1	1	0	0	0	1	1	4
5246	66	1	0	0	0	0	1	1	1	1	1	0	0	0	0	1	4
5248	83	1	1	1	1	0	2	1	1	1	0	0	0	0	0	1	4

5250	85	2	1	1	1	1	1	1	0	0	0	0	1	0	0	1	1
5252	71	1	0	0	1	0	1	1	1	1	1	0	0	0	0	1	4
5253	66	2	0	0	0	0	1	0	1	0	1	0	0	0	0	2	2
5257	73	2	1	0	1	1	3	1	0	0	0	1	0	0	0	3	4
5258	70	2	0	1	0	1	1	#NULL!	#NULL!	#NULL!	0	1	#NULL!	0	0	3	4
5260	77	2	1	0	0	1	1	1	0	0	1	0	1	0	0	4	1
5261	74	2	0	1	0	1	1	0	0	0	0	1	0	0	0	4	1
5263	71	2	0	0	0	1	3	1	#NULL!	0	0	0	0	0	0	4	4
5269	67	1	0	0	0	0	1	0	1	1	0	1	0	0	1	2	4
5270	65	2	0	0	0	0	1	#NULL!	#NULL!	#NULL!	1	1	#NULL!	0	1	2	1
5271	67	2	0	0	0	0	3	1	0	0	0	0	0	0	0	1	3
5274	69	2	0	1	1	1	3	0	0	0	1	0	0	0	0	3	3
5277	77	1	0	0	0	#NULL!	1	1	0	1	0	0	0	1	0	1	4
5278	71	2	0	0	0	0	1	0	0	0	1	1	0	0	0	1	1
5279	75	1	0	0	0	0	1	#NULL!	#NULL!	#NULL!	0	1	#NULL!	0	0	4	1
5280	71	2	0	0	0	1	1	0	0	0	0	0	0	0	0	2	4
5281	77	1	0	0	0	1	1	1	1	1	0	0	1	0	0	1	4
5282	67	2	1	0	0	1	1	0	0	0	0	0	0	0	0	2	1
5283	89	1	1	0	0	#NULL!	1	2	1	1	0	0	0	0	0	4	1
5284	88	2	1	0	0	#NULL!	1	2	0	0	0	0	0	0	0	2	1
5285	72	1	0	0	0	1	1	0	0	1	1	0	0	0	0	1	4
5286	66	2	0	0	0	1	1	0	0	0	1	0	0	0	0	2	2
5288	75	2	0	0	0	1	3	0	0	0	1	1	0	0	0	2	3
5290	67	2	0	1	0	1	2	1	0	0	0	0	1	0	0	1	2
5291	74	2	0	1	0	0	1	1	0	0	0	0	0	0	0	2	1
5292	78	2	1	1	0	1	1	0	0	0	1	0	0	0	0	4	1
5293	76	1	0	0	0	0	1	#NULL!	1	1	1	1	0	1	0	1	4
5296	73	1	0	0	0	0	1	1	0	0	1	1	0	0	0	1	4
5297	68	2	0	0	0	0	3	0	0	0	1	0	1	0	0	2	1
5299	69	1	0	0	0	1	1	0	1	1	1	1	0	0	0	1	4
5300	67	2	1	0	0	1	1	1	0	0	0	1	0	0	0	4	1
5304	70	2	0	0	1	1	1	#NULL!	#NULL!	#NULL!	0	1	#NULL!	0	0	2	2
5305	70	1	1	0	1	1	1	#NULL!	#NULL!	#NULL!	1	0	#NULL!	0	1	2	4
5306	79	2	0	1	0	0	1	0	0	0	0	0	0	0	0	2	4
5307	66	1	0	0	1	0	1	0	1	1	1	0	0	0	0	1	4
5309	70	1	0	0	0	1	1	0	0	1	1	0	0	0	0	3	4
5310	66	2	0	0	0	1	1	0	0	0	0	0	0	0	0	2	1
5313	74	1	0	0	0	1	1	0	0	1	0	1	0	0	0	1	4
5314	66	2	0	0	0	1	1	0	0	0	0	0	1	0	0	2	2

5316	82	2	0	0	1	0	1	0	0	0	0	0	1	0	0	4	1
5318	73	1	0	0	1	1	1	0	1	1	1	1	0	1	0	3	4
5321	76	2	0	0	1	1	2	0	0	0	0	0	1	0	0	4	4
5322	65	2	0	1	1	1	1	#NULL!	#NULL!	#NULL!	0	0	#NULL!	0	0	3	4
5323	75	2	1	#NULL!	1	#NULL!	3	#NULL!	#NULL!	#NULL!	0	1	#NULL!	#NULL!	0	4	4
5330	66	2	0	0	0	0	1	0	0	0	0	0	0	0	0	1	2
5333	70	1	0	0	0	0	1	0	1	1	1	1	0	0	0	3	4
5335	72	1	0	0	0	0	1	2	1	1	0	0	0	0	1	3	4
5336	66	2	0	0	0	0	1	1	0	0	0	0	0	0	0	2	1
5337	83	2	0	1	0	1	1	1	0	0	0	1	0	0	0	2	3
5338	85	2	1	1	0	0	3	1	1	0	0	0	1	1	0	4	1
5339	73	2	1	0	0	1	3	1	0	0	0	0	0	0	0	2	4
5341	72	2	0	#NULL!	0	#NULL!	1	#NULL!	#NULL!	#NULL!	1	0	#NULL!	0	0	4	1
5345	72	1	0	0	1	1	3	2	1	1	0	1	0	0	1	1	4
5346	66	2	0	0	0	1	1	#NULL!	#NULL!	#NULL!	0	0	#NULL!	0	1	2	3
5350	65	2	0	1	0	0	2	0	0	0	0	1	0	0	1	1	4
5351	71	2	0	1	0	1	1	0	0	1	0	0	0	0	1	2	4
5354	69	1	0	0	0	1	1	0	1	1	1	0	0	0	0	1	4
5355	69	2	0	1	0	0	2	1	0	0	0	1	0	0	1	2	4
5357	79	1	0	0	0	0	3	2	1	1	0	0	0	0	0	2	4
5358	79	2	0	0	0	0	1	2	0	0	0	0	0	0	0	2	1
5360	69	1	0	0	1	0	1	1	0	0	0	1	0	0	1	1	4
5361	66	2	0	0	0	0	3	0	0	0	0	1	0	0	0	2	3
5366	74	1	0	0	0	0	1	1	0	1	1	0	0	0	0	1	4
5367	71	2	0	0	0	0	1	0	0	0	1	1	0	0	0	1	2
5370	67	1	0	0	0	1	1	1	1	1	0	0	0	0	0	3	4
5371	75	1	0	0	0	1	1	0	1	1	1	0	0	0	0	1	4
5372	72	2	0	0	0	0	1	2	0	0	1	0	0	0	0	2	4
5373	67	2	0	1	0	1	1	0	0	0	0	0	0	0	1	1	2
5375	73	1	0	0	0	1	1	1	0	0	0	1	0	0	0	1	4
5376	69	2	0	0	0	1	1	2	0	0	0	1	0	0	0	2	2
5381	73	2	0	0	0	1	1	2	0	0	1	1	1	0	0	1	2
5382	71	1	0	0	0	0	3	0	0	0	1	0	0	0	1	1	4
5383	66	2	0	0	0	0	3	1	0	0	1	0	0	0	0	2	2
5390	81	2	0	#NULL!	0	#NULL!	1	1	0	0	1	0	0	0	1	1	3
5391	76	1	0	0	0	0	1	#NULL!	#NULL!	#NULL!	1	0	#NULL!	0	0	1	4
5393	79	2	0	0	0	1	3	1	1	0	0	0	0	0	0	1	4
5394	77	1	1	0	0	1	1	2	1	1	0	0	1	1	0	1	4
5400	66	2	0	1	0	0	1	1	0	0	0	0	0	0	0	2	3

5403	67	2	0	1	0	1	1	1	0	0	0	0	0	0	1	2	4
5404	75	1	0	0	0	1	1	2	1	1	1	0	0	0	0	2	4
5406	69	2	1	1	0	1	1	0	0	0	1	1	0	0	1	2	2
5407	67	1	1	0	0	1	3	2	0	1	0	0	0	1	1	2	4
5408	78	1	0	0	1	1	1	2	0	0	0	0	1	0	0	1	4
5409	72	2	0	0	1	1	1	0	0	0	0	0	1	0	0	3	2
5410	85	2	0	1	1	1	1	1	0	0	0	0	0	0	0	2	4
5411	70	1	1	0	0	1	3	2	0	1	0	0	0	0	0	2	4
5412	66	2	0	0	0	1	1	0	0	0	0	0	0	0	0	2	3
5423	75	2	0	0	#NULL!	0	2	0	0	0	0	0	0	0	0	4	1
5427	65	2	0	1	1	1	1	1	0	0	0	0	0	0	1	4	1
5431	68	1	0	0	0	1	1	1	0	0	0	0	0	0	0	3	4
5435	67	1	1	0	0	1	1	0	1	1	0	0	0	0	1	2	4
5438	66	2	1	0	0	0	1	0	0	0	1	0	1	0	1	1	3
5440	70	2	1	#NULL!	1	#NULL!	1	0	0	0	1	0	0	0	1	2	4
5442	67	2	0	1	1	1	3	1	0	0	0	0	0	0	0	4	1
5447	71	2	0	0	0	1	1	1	0	0	0	1	0	0	0	3	4
5454	71	2	0	0	0	1	2	2	0	0	1	1	0	0	0	2	2
5456	66	1	1	0	0	1	1	1	0	1	0	0	0	1	0	3	4
5457	65	2	0	0	0	1	2	0	0	0	0	0	1	0	0	2	4
5461	72	1	0	0	1	0	1	0	0	0	1	1	0	0	1	3	4
5462	71	1	0	0	1	1	1	1	0	1	1	1	0	0	0	1	4
5465	72	1	0	0	0	0	1	1	1	1	1	1	1	0	1	1	4
5467	77	1	0	0	0	0	1	1	0	1	1	1	0	0	1	1	4
5468	73	2	0	0	0	0	2	0	0	0	0	0	0	0	0	1	4
5469	70	2	0	1	0	0	1	0	1	0	1	0	0	0	1	2	4
5470	87	2	0	0	0	#NULL!	1	1	0	0	0	1	1	1	0	3	1
5471	90	1	0	1	0	0	1	0	0	1	0	1	0	0	1	1	4
5475	84	1	0	0	0	0	1	#NULL!	#NULL!	#NULL!	0	0	#NULL!	0	1	1	2
5476	80	2	0	0	0	#NULL!	1	#NULL!	#NULL!	#NULL!	0	0	#NULL!	0	0	4	1
5477	94	2	0	1	0	0	#NULL!	1	0	0	0	1	0	0	1	1	4
5482	72	1	0	0	0	1	1	0	1	1	1	1	0	0	1	1	4
5483	69	2	0	0	0	1	3	0	0	1	1	1	0	0	1	2	4
5485	73	1	0	0	0	0	1	1	0	1	0	0	0	0	0	3	4
5486	69	2	0	0	0	0	1	1	0	0	1	0	0	0	0	4	4
5487	85	1	1	0	0	0	3	2	1	1	0	0	1	0	0	3	4
5488	81	2	0	0	1	0	1	#NULL!	0	0	1	0	1	1	0	2	4
5490	66	2	0	0	1	1	1	1	0	1	0	0	0	0	0	3	2
5491	74	2	0	1	0	1	1	0	0	0	0	0	0	0	0	3	3

5492	77	1	0	0	1	1	1	1	1	1	1	0	0	0	0	3	4
5493	74	2	1	0	1	1	1	1	0	0	1	0	0	0	0	3	3
5494	77	1	0	0	1	1	1	0	0	1	0	0	0	0	0	3	3
5495	75	2	0	0	1	1	1	1	0	0	0	0	0	0	1	2	4
5497	80	2	0	1	1	0	1	0	0	0	1	0	0	0	0	3	4
5498	85	2	1	#NULL!	1	#NULL!	1	2	0	0	0	0	0	0	0	4	1
5499	73	1	1	0	0	#NULL!	3	1	1	1	0	1	0	1	0	2	1
5503	77	2	1	0	0	1	1	#NULL!	0	0	0	0	0	0	0	4	4
5505	66	1	0	0	0	1	3	1	0	1	0	0	0	0	1	1	4
5508	86	1	1	0	1	0	1	2	1	1	1	0	1	0	0	1	4
5509	84	2	0	0	1	0	1	1	0	0	0	0	1	0	0	2	3
5511	73	1	0	0	1	0	1	0	0	1	0	0	0	0	1	3	4
5512	71	2	0	0	0	0	1	0	0	0	0	0	0	0	1	4	4
5513	69	1	0	0	0	1	3	1	1	1	0	1	0	0	0	1	4
5514	67	2	0	0	0	1	1	0	0	0	1	0	0	0	0	2	2
5519	68	1	0	1	1	1	1	1	1	1	0	0	0	0	0	3	4
5520	69	1	0	0	#NULL!	1	1	1	1	1	0	0	1	0	1	3	4
5521	65	2	0	0	0	1	1	0	0	0	1	0	1	0	0	3	4
5523	74	1	0	0	1	1	1	1	0	1	0	0	0	0	0	3	4
5524	69	2	0	0	0	1	1	#NULL!	0	1	0	0	0	0	0	2	1
5529	86	2	1	1	0	1	3	1	0	0	0	0	1	0	0	3	3
5530	83	2	0	1	1	1	3	1	0	0	0	0	0	0	0	2	4
5531	70	2	0	0	0	#NULL!	1	#NULL!	0	0	0	0	0	0	0	3	3
5532	80	2	0	0	0	1	3	2	0	0	0	1	0	0	1	2	4
5533	74	1	0	0	0	1	1	0	0	0	0	0	0	0	1	1	4
5536	73	2	0	1	0	0	1	0	0	0	0	0	0	0	0	2	2
5537	69	1	0	0	0	1	1	0	1	1	0	0	0	0	1	2	4
5539	78	2	0	1	#NULL!	1	3	#NULL!	0	0	0	0	0	0	0	2	3
5540	82	1	0	0	1	1	1	2	0	1	0	0	1	0	0	3	4
5542	65	2	0	1	0	1	1	0	0	0	0	0	1	0	1	2	4
5553	67	1	0	0	0	0	1	1	1	1	1	1	0	0	1	3	4
5555	82	2	1	1	1	1	1	2	0	0	0	0	1	0	0	1	4
5559	68	1	0	0	0	0	1	0	0	0	0	0	0	0	0	3	4
5560	66	1	0	0	0	1	1	1	0	1	1	0	0	0	0	2	4
5564	70	2	0	1	0	0	2	0	0	0	1	1	0	0	0	1	2
5567	73	2	0	0	0	1	3	2	0	0	0	0	0	0	0	2	2
5568	73	1	0	0	0	1	3	2	0	1	0	0	0	0	0	1	4
5570	78	2	1	0	0	1	3	2	0	0	0	0	1	0	0	2	2
5571	78	2	0	1	1	1	1	0	1	0	0	0	0	0	1	1	4

5574	76	1	0	1	1	1	3	#NULL!	#NULL!	#NULL!	1	1	#NULL!	0	0	3	4
5575	68	1	0	0	0	1	1	1	1	1	0	0	0	0	0	2	4
5579	73	2	1	0	1	0	1	0	0	0	1	0	0	0	0	3	4
5580	79	2	0	1	0	1	1	1	1	#NULL!	0	1	#NULL!	0	0	3	4
5583	73	1	0	0	0	#NULL!	1	0	0	0	0	0	0	0	0	1	4
5584	66	2	0	1	1	1	1	0	0	0	1	0	0	0	0	4	4
5586	71	2	1	0	1	1	3	1	0	0	0	0	0	0	0	4	3
5589	71	1	0	0	0	1	3	0	1	1	1	0	0	1	1	1	2
5590	66	2	0	0	0	1	1	0	0	0	0	0	0	0	0	2	2
5591	66	1	0	0	0	0	1	2	0	1	0	0	0	1	0	2	4
5593	68	1	0	0	0	0	1	0	0	0	1	0	0	0	1	1	4
5594	66	2	0	0	0	0	2	#NULL!	0	0	0	0	0	0	0	2	2
5595	65	2	0	0	0	1	1	1	0	0	0	0	1	0	0	4	2
5599	75	2	0	1	1	1	#NULL!	#NULL!	0	0	0	1	0	0	0	4	2
5603	69	1	0	0	0	1	1	1	1	1	0	0	0	0	0	2	4
5605	80	2	0	0	0	#NULL!	2	2	0	0	0	0	1	0	0	2	1
5607	68	1	0	0	0	0	1	0	1	0	0	1	0	0	1	1	4
5608	65	1	0	0	0	1	1	0	0	1	0	0	0	0	1	3	4
5615	70	2	0	0	0	0	3	1	0	0	0	0	0	0	0	2	4
5616	66	1	0	0	0	0	1	1	0	0	0	0	0	0	0	1	4
5617	73	1	0	0	1	#NULL!	1	0	1	1	1	0	0	0	0	3	4
5618	72	2	0	0	1	0	2	#NULL!	#NULL!	#NULL!	1	0	#NULL!	0	0	3	4
5620	65	2	0	0	1	1	1	1	0	0	0	0	0	0	0	2	3
5621	70	2	0	1	0	0	1	0	1	0	0	0	0	0	0	2	3
5624	65	2	0	0	0	0	1	#NULL!	0	0	0	1	0	0	0	4	1
5626	66	1	1	0	0	1	1	1	0	1	0	0	0	0	1	2	4
5628	69	1	0	0	0	1	1	0	1	1	1	0	0	0	1	2	4
5629	67	2	0	0	0	1	1	0	0	0	0	0	0	0	0	2	3
5630	67	2	0	1	0	1	1	1	0	1	0	0	0	0	1	2	4
5634	76	1	0	0	0	1	#NULL!	1	1	0	1	1	0	0	1	2	4
5635	72	2	0	0	0	1	#NULL!	0	0	0	1	0	0	0	1	2	4
5637	73	1	0	0	0	1	3	1	1	1	0	0	0	0	0	1	4
5638	67	2	0	0	0	1	3	1	0	0	0	1	0	0	0	4	4
5643	67	1	0	0	0	1	1	1	1	1	0	1	0	0	0	2	4
5647	74	2	0	0	0	0	1	0	0	0	0	0	0	0	0	2	2
5649	69	1	0	0	0	1	3	2	1	1	0	0	1	0	1	3	4
5650	66	2	1	0	0	1	1	1	0	1	0	0	0	0	0	2	3
5651	69	1	0	0	0	0	3	0	1	0	0	0	0	1	1	2	4
5654	75	1	0	1	0	0	1	0	0	0	1	1	0	0	0	3	4

5656	76	2	0	0	0	1	1	0	0	0	0	0	0	0	0	2	3
5658	76	1	0	0	0	0	1	#NULL!	#NULL!	#NULL!	0	0	#NULL!	0	1	1	4
5664	84	2	1	1	0	1	1	1	0	0	1	1	0	0	0	2	4
5665	83	1	0	0	0	#NULL!	#NULL!	0	0	1	0	0	0	1	0	1	4
5666	82	2	0	0	0	0	1	0	0	0	0	1	0	0	0	4	2
5669	73	1	0	0	1	0	1	0	1	0	1	0	0	0	0	2	4
5670	79	1	0	0	0	0	3	1	0	1	0	0	0	0	0	2	4
5671	72	2	0	0	0	1	1	1	0	0	1	0	0	0	0	2	3
5672	66	1	0	0	0	1	1	#NULL!	0	0	1	1	0	0	0	2	4
5674	79	2	0	#NULL!	1	1	1	1	0	1	0	0	1	0	0	4	1
5675	78	1	0	0	1	0	1	1	1	1	0	0	0	0	0	3	4
5679	78	2	0	0	0	#NULL!	#NULL!	0	0	0	0	0	1	0	0	2	2
5680	76	1	0	0	1	0	1	0	1	0	0	0	0	0	0	2	4
5682	83	2	0	1	1	1	1	1	0	0	0	0	0	0	0	2	3
5685	82	1	0	1	0	0	1	2	0	1	0	0	0	0	0	4	1
5686	71	1	0	0	0	1	1	2	1	1	0	0	1	0	1	3	4
5694	82	2	0	1	0	0	3	2	0	0	0	0	0	0	0	3	4
5698	65	1	0	0	0	1	3	0	0	0	0	0	0	0	1	3	4
5704	74	1	1	0	0	0	1	1	0	1	0	0	0	0	0	1	4
5705	69	2	0	0	0	#NULL!	1	0	0	0	1	0	0	0	0	2	4
5708	80	2	0	1	0	1	1	1	0	0	0	0	1	0	0	2	4
5709	66	1	0	1	0	0	3	1	0	1	1	0	0	0	0	2	4
5710	71	1	1	0	1	1	1	1	1	1	0	0	0	0	0	1	4
5711	67	2	0	0	1	1	3	0	0	0	1	1	0	0	0	2	3
5713	70	1	0	1	1	1	3	1	0	0	1	1	0	0	1	1	4
5714	70	1	0	0	0	0	3	1	1	1	1	1	0	0	0	2	4
5715	67	2	0	0	0	1	1	1	0	0	1	1	0	0	0	1	4
5717	69	1	0	0	0	1	1	1	1	1	1	1	0	0	0	2	4
5721	73	1	0	0	0	1	1	1	0	1	0	0	1	1	0	1	4
5724	65	1	0	0	1	1	1	1	0	1	0	0	0	0	1	3	4
5728	76	1	1	0	1	1	3	2	0	1	0	0	0	1	0	3	4
5729	75	2	0	0	0	1	1	0	0	1	0	0	1	0	0	1	4
5730	77	1	1	#NULL!	1	#NULL!	1	1	1	0	1	1	0	0	0	3	4
5733	73	1	0	0	0	1	1	1	0	0	1	1	0	1	1	2	4
5735	72	1	0	0	1	1	1	1	0	1	0	0	0	1	0	3	4
5736	69	2	0	0	1	1	3	1	0	0	0	0	0	0	0	4	2
5737	81	2	0	1	0	1	1	1	0	0	1	0	0	0	0	2	1
5739	66	2	0	0	1	0	3	0	0	0	0	0	0	0	1	2	4
5740	65	1	0	0	1	0	3	1	1	1	0	0	0	0	1	2	4

5741	72	2	0	1	1	1	3	1	0	0	0	0	0	1	1	4	4
5744	76	1	0	0	1	0	1	1	0	1	0	0	0	0	0	1	4
5745	75	2	0	0	1	0	1	1	0	0	0	0	0	0	0	2	1
5746	70	1	0	0	1	1	1	1	0	1	0	0	0	0	1	1	4
5747	76	1	0	0	0	1	3	1	1	1	1	0	0	0	1	2	4
5748	70	2	0	0	0	0	1	1	0	0	1	0	0	0	1	2	4
5749	74	1	0	0	1	#NULL!	1	0	1	0	0	1	0	0	1	3	4
5750	69	2	0	0	0	#NULL!	1	0	0	0	0	0	0	0	1	3	4
5751	77	1	0	0	0	0	1	1	1	1	1	0	0	0	0	1	4
5752	73	2	0	0	1	1	1	1	0	0	0	0	0	0	0	3	2
5753	69	2	1	1	0	1	1	1	0	0	0	0	1	0	1	2	4
5754	71	2	0	1	0	1	3	1	0	0	0	0	0	0	0	2	3
5756	74	2	0	0	1	1	#NULL!	1	0	0	1	1	1	0	0	3	4
5757	79	2	0	1	0	1	1	0	0	0	0	0	1	0	0	3	4
5758	84	2	1	1	0	#NULL!	3	0	0	1	0	0	1	0	0	1	4
5759	81	1	0	0	1	0	1	0	0	0	0	1	0	0	0	3	4
5761	65	2	0	0	0	0	3	1	1	1	1	0	0	0	0	4	1
5763	79	2	0	0	0	0	1	0	0	#NULL!	0	0	0	0	0	4	1
5769	79	1	1	0	1	1	1	1	0	1	0	0	1	0	0	3	4
5774	71	2	0	0	0	#NULL!	1	0	0	0	0	0	0	0	0	2	2
5775	69	1	0	0	0	0	1	0	1	1	0	1	0	1	1	1	4
5776	69	1	0	0	0	#NULL!	1	0	0	1	1	0	0	0	0	2	4
5777	73	1	0	0	0	1	1	0	1	1	0	0	0	0	0	2	4
5778	66	2	0	0	0	1	1	1	0	0	0	0	0	0	0	3	4
5779	70	1	1	0	1	0	1	0	0	1	1	0	0	1	1	1	4
5780	65	2	0	0	0	0	3	0	0	0	0	0	0	0	1	2	4
5783	73	1	0	1	0	0	1	2	0	1	1	1	1	0	0	1	4
5784	71	2	0	0	0	0	3	0	1	0	1	1	1	0	0	2	1
5785	70	1	0	0	0	1	3	2	1	1	0	0	0	0	1	2	4
5786	65	2	0	0	0	1	1	0	1	0	1	1	0	0	1	2	2
5788	66	2	0	0	0	#NULL!	1	1	0	0	0	0	0	0	0	3	2
5789	66	2	0	0	0	1	3	1	0	0	0	0	0	0	1	3	2
5790	65	1	0	0	0	1	#NULL!	1	1	1	0	0	0	0	1	3	3
5791	74	1	0	0	0	#NULL!	1	0	1	0	1	0	0	0	1	3	2
5794	66	2	0	1	1	0	1	0	1	1	1	1	0	0	1	2	4
5795	75	1	0	0	0	1	1	1	0	1	0	1	0	1	0	2	4
5796	70	2	0	0	0	1	1	2	0	1	1	0	0	0	0	2	4
5797	66	1	0	0	0	1	3	#NULL!	#NULL!	#NULL!	0	0	#NULL!	0	0	1	4
5798	65	2	0	0	0	1	3	1	0	0	1	1	0	0	0	2	2

5801	68	1	1	0	0	1	1	0	1	0	1	0	0	0	1	3	4
5806	73	1	0	0	1	1	1	0	0	1	0	0	0	0	0	3	4
5807	73	2	0	0	1	1	1	0	0	0	0	0	0	0	0	3	2
5808	74	2	0	1	0	0	1	1	0	0	1	0	0	0	0	1	4
5810	69	2	0	0	0	0	1	1	0	0	1	0	0	0	0	3	4
5812	74	1	0	1	0	0	1	0	0	0	0	0	1	0	0	3	4
5813	73	2	0	1	0	1	3	#NULL!	0	0	1	1	1	0	0	2	4
5815	68	2	0	0	0	1	1	#NULL!	0	0	0	0	0	0	0	3	4
5818	67	2	0	0	0	0	3	0	0	0	1	1	0	0	0	2	1
5819	66	1	0	0	0	0	1	1	1	1	0	1	0	0	1	1	4
5823	66	1	0	0	0	1	3	0	0	1	0	0	1	0	0	2	4
5824	70	1	0	0	0	0	1	0	0	0	0	0	0	0	0	1	4
5825	68	2	0	0	0	0	2	#NULL!	#NULL!	#NULL!	1	0	#NULL!	0	1	1	2
5831	65	2	0	0	0	0	1	1	0	0	0	1	0	0	0	1	3
5834	69	1	0	0	0	0	1	2	0	0	1	1	0	0	0	1	4
5835	84	1	1	0	0	1	1	2	0	1	0	0	0	0	0	3	4
5836	82	2	1	0	1	1	3	1	0	#NULL!	1	0	0	0	0	4	4
5837	80	2	0	1	0	0	1	#NULL!	0	0	1	0	1	0	0	4	4
5838	86	1	0	0	1	1	1	0	0	0	1	0	1	0	0	4	1
5840	75	2	0	0	#NULL!	1	1	0	0	0	0	0	0	0	0	4	2
5841	74	1	1	0	1	1	3	1	0	1	1	0	0	1	0	3	4
5842	85	2	0	1	0	0	1	1	0	0	0	1	0	0	0	3	4
5843	69	2	0	0	0	0	1	1	1	0	1	0	1	0	1	2	2
5846	80	1	0	0	1	0	1	#NULL!	0	0	1	0	0	0	0	1	4
5850	69	2	0	0	0	0	1	2	0	0	1	1	#NULL!	0	0	2	2
5854	78	1	0	0	0	0	3	2	1	1	1	1	0	0	0	3	2
5855	78	2	0	0	0	1	1	1	0	0	1	0	1	0	0	2	2
5857	69	2	0	0	0	1	3	1	0	0	1	0	0	0	0	4	1
5858	79	2	1	1	1	0	1	0	0	0	1	0	0	0	0	2	1
5859	76	1	0	0	0	0	1	0	0	1	1	1	0	1	0	1	4
5860	76	2	0	0	0	0	1	0	0	0	0	0	0	0	1	3	2
5863	74	1	1	0	0	0	1	1	1	0	0	0	0	0	0	1	4
5864	70	2	0	0	0	1	1	1	0	0	1	1	1	0	0	1	4
5866	66	1	0	0	0	1	2	0	0	0	0	0	0	1	1	3	4
5867	76	2	1	1	0	0	1	0	0	0	0	0	1	0	0	2	3
5868	82	1	0	0	1	0	1	1	0	1	1	1	0	0	0	3	4
5869	79	2	1	0	1	0	1	0	0	0	1	0	0	0	0	3	4
5870	73	1	0	0	1	1	1	0	0	1	0	0	1	0	0	3	4
5871	69	2	0	0	1	1	1	0	0	0	0	0	0	0	0	3	4

5873	76	2	0	0	1	1	1	1	0	0	0	0	0	0	0	3	1
5874	81	2	0	0	0	0	1	0	0	0	1	0	0	0	0	2	4
5877	88	2	0	1	0	1	1	0	0	0	0	0	1	0	0	2	3
5880	71	1	0	0	0	1	1	1	1	1	0	0	0	0	0	2	2
5881	67	1	0	0	0	0	1	0	0	1	0	0	0	0	1	1	4
5882	68	1	0	0	1	1	1	0	1	0	1	1	0	1	0	1	4
5883	86	1	0	1	1	0	1	0	0	1	1	0	0	0	0	3	4
5885	69	2	0	1	0	1	1	0	0	0	1	0	0	0	0	1	4
5890	76	1	0	0	1	1	1	0	0	0	0	0	0	0	1	3	4
5891	72	2	0	0	1	1	2	0	0	0	0	1	0	0	1	1	4
5892	65	2	0	1	1	1	1	0	0	0	0	0	0	0	1	1	1
5895	76	1	0	0	0	1	3	1	0	0	1	1	0	0	0	2	4
5896	73	2	0	0	0	#NULL!	1	1	0	0	1	1	0	0	0	1	1
5898	65	1	0	0	0	1	1	2	1	1	0	0	0	0	1	2	4
5900	81	1	0	0	0	0	3	1	0	0	1	0	0	0	0	1	4
5901	80	2	0	0	0	0	3	1	0	0	1	0	0	0	0	4	1
5902	69	1	0	0	0	0	3	0	1	1	1	0	0	0	0	1	4
5903	66	2	0	0	0	0	1	0	0	0	0	0	0	0	0	4	1
5905	69	1	0	0	0	0	1	1	0	1	1	0	0	0	1	4	4
5906	67	2	0	0	0	0	1	1	0	0	0	0	0	0	0	2	1
5907	73	1	0	0	0	1	1	0	0	0	0	1	0	0	0	1	4
5908	66	2	0	0	0	1	1	0	0	0	0	0	1	0	0	2	2
5910	66	2	0	0	0	0	1	0	1	1	1	0	0	0	0	1	4
5911	73	2	0	1	1	1	1	1	0	0	0	0	0	0	0	4	1
5916	80	2	1	0	0	1	1	0	0	1	0	0	0	0	0	2	3
5917	68	1	0	0	0	1	1	0	0	1	0	0	0	0	0	2	4
5918	66	2	0	0	0	1	1	0	0	0	0	0	1	0	1	1	4
5919	67	1	0	0	0	1	1	0	1	1	1	0	0	0	1	1	4
5921	74	1	0	0	0	1	1	0	0	0	1	0	1	0	0	1	4
5923	66	1	0	0	0	1	1	1	0	0	0	0	0	1	0	1	4
5925	67	1	0	0	0	1	3	0	0	1	0	0	0	0	0	2	4
5928	73	2	0	0	0	1	1	0	0	0	0	0	1	0	0	3	4
5930	66	2	0	0	0	1	3	0	0	0	1	0	0	0	0	2	2
5931	72	1	0	1	0	0	1	0	1	0	0	1	0	0	0	2	4
5932	87	2	1	1	1	0	1	2	0	0	0	0	1	1	0	3	3
5936	72	1	1	0	0	0	3	1	0	1	0	1	1	0	1	3	4
5938	77	1	1	0	0	1	3	2	1	1	0	0	0	0	0	1	4
5939	77	2	0	0	0	0	1	1	0	0	0	1	0	0	0	2	2
5940	69	1	0	0	0	1	1	0	1	1	1	1	0	1	1	2	4

5944	72	1	0	0	0	#NULL!	3	1	0	1	1	1	0	0	0	3	4
5945	71	1	1	0	1	1	1	1	1	1	0	0	0	0	0	1	4
5946	67	2	0	0	1	1	1	0	1	0	0	0	0	0	0	2	4
5947	66	2	0	1	0	1	3	2	0	0	0	0	0	0	1	4	4
5948	73	1	0	0	0	0	1	1	1	1	1	0	0	0	0	2	4
5949	70	2	0	0	0	0	1	2	0	0	1	0	0	0	0	2	3
5950	72	1	0	0	0	0	1	1	1	1	1	1	0	0	1	2	2
5951	71	1	1	0	0	0	2	0	1	1	1	0	0	0	0	3	4
5952	70	2	0	0	1	0	1	#NULL!	#NULL!	#NULL!	1	1	#NULL!	0	0	3	3
5953	82	2	1	1	0	1	1	1	0	0	1	0	1	0	0	3	2
5955	66	2	1	0	1	0	1	0	0	0	0	0	1	0	0	2	1
5956	73	1	0	0	0	0	1	1	0	1	1	0	0	0	0	4	4
5957	72	2	1	0	0	1	1	0	0	0	1	1	0	0	0	2	1
5962	72	2	0	0	1	1	1	0	0	0	0	0	0	0	0	3	3
5964	86	1	0	0	0	0	1	1	0	1	0	0	0	0	0	3	4
5967	67	2	0	0	0	1	1	0	0	0	1	0	0	0	0	2	1
5971	69	2	0	1	0	1	1	1	1	1	0	0	0	0	0	3	2
5974	76	1	0	1	0	1	1	0	1	0	0	0	0	0	1	2	4
5977	67	1	0	0	0	1	3	2	1	1	0	0	0	0	0	3	4
5978	67	2	0	0	1	1	1	2	0	0	0	0	0	0	0	2	2
5982	73	2	0	0	0	0	1	#NULL!	0	0	1	0	0	0	0	4	1
5983	71	1	0	0	0	#NULL!	1	0	1	1	1	1	0	0	0	1	4
5984	69	1	0	0	1	#NULL!	1	1	1	1	0	1	0	0	0	2	4
5985	66	2	0	0	1	1	3	1	0	1	0	1	0	0	0	2	2
5988	67	2	1	0	0	1	1	1	0	0	0	1	0	0	0	1	2
5992	68	2	0	0	1	1	1	0	0	0	1	0	0	0	0	2	4
5993	66	1	0	0	0	1	1	0	0	1	1	0	0	0	1	1	4
5995	71	1	1	0	0	0	3	2	1	1	0	1	0	0	0	2	4
5997	74	1	0	0	0	1	1	2	1	1	0	0	0	0	0	2	4
5998	67	2	0	0	1	1	1	0	0	0	0	0	0	0	0	2	4
5999	66	1	0	0	0	1	1	0	1	1	0	0	0	0	1	4	4
6000	67	1	0	0	0	1	1	1	0	1	0	0	1	0	0	2	4
6004	67	2	0	0	0	0	1	0	0	0	1	0	0	0	0	2	3
6005	70	1	0	0	0	0	3	2	1	1	0	0	0	0	1	2	4
6006	69	2	1	0	0	0	1	0	0	0	0	0	0	0	1	2	4
6007	78	2	0	1	0	1	1	#NULL!	0	#NULL!	0	0	0	0	0	2	4
6009	67	1	0	0	0	1	1	1	1	1	1	0	0	0	0	1	4
6012	70	2	0	0	1	1	2	0	0	0	0	1	1	1	0	2	4
6013	70	1	0	0	1	0	1	#NULL!	1	1	1	0	0	0	0	1	4

6014	85	2	0	1	0	0	1	1	0	0	0	1	0	0	0	2	3
6017	78	1	0	0	0	0	3	1	1	1	1	1	0	0	0	2	4
6018	76	2	0	0	0	0	1	2	0	0	1	0	0	0	0	3	4
6019	66	2	0	1	0	0	1	1	0	0	1	1	0	0	0	2	3
6020	76	1	0	0	0	0	1	0	1	1	1	1	0	0	0	1	4
6021	75	2	0	0	0	0	1	#NULL!	0	0	1	1	0	0	0	4	1
6022	66	2	0	1	0	0	1	0	1	0	1	0	0	0	0	2	1
6023	82	1	0	0	0	0	1	1	0	0	0	0	0	0	0	4	1
6024	79	2	0	0	1	0	1	1	0	0	1	1	0	0	0	3	2
6028	73	2	0	1	0	1	1	0	0	0	0	0	1	0	0	2	1
6030	66	1	0	0	0	0	3	1	1	1	1	0	0	0	1	1	4
6033	75	2	0	0	0	0	1	#NULL!	0	0	0	0	1	0	1	2	4
6036	72	1	0	0	0	0	1	2	1	1	1	0	0	0	0	1	4
6039	66	1	0	0	0	0	1	0	1	1	1	1	0	0	0	2	4
6040	66	2	0	0	0	0	2	#NULL!	0	0	1	1	0	0	0	1	1
6046	83	2	0	0	0	0	1	#NULL!	0	0	1	0	0	0	0	1	4
6049	68	1	0	0	0	1	1	0	1	1	0	1	1	0	0	1	4
6052	71	2	0	0	1	0	3	1	0	0	1	1	0	0	0	2	1
6055	86	2	0	1	1	0	2	0	0	0	1	1	0	0	0	3	3
6056	65	1	0	0	0	0	1	#NULL!	0	1	1	0	0	0	0	1	4
6059	89	1	0	0	0	0	1	2	0	1	1	1	0	0	1	1	4
6061	69	1	0	0	#NULL!	1	1	1	1	0	1	0	0	0	0	3	4
6062	66	2	0	0	0	0	1	#NULL!	#NULL!	#NULL!	1	0	#NULL!	0	0	2	2
6064	87	2	1	1	0	1	1	0	0	0	0	1	0	0	0	3	4
6068	84	1	1	0	0	0	2	1	0	1	1	0	1	0	0	1	4
6070	86	1	1	0	0	0	1	2	1	1	0	0	1	0	0	1	4
6073	73	1	0	0	0	1	1	1	1	1	1	0	0	1	0	2	4
6074	68	2	0	0	0	1	2	1	0	0	1	1	0	0	0	2	2
6075	74	2	0	0	0	1	3	1	0	0	1	0	0	0	0	3	3
6083	72	1	0	1	0	0	3	2	1	0	0	1	0	0	0	1	4
6084	71	1	1	1	0	1	3	2	0	1	0	0	1	0	0	3	4
6090	72	1	0	0	0	1	1	0	1	0	0	1	0	0	0	1	4
6091	69	2	0	#NULL!	0	#NULL!	1	#NULL!	#NULL!	#NULL!	0	0	#NULL!	0	0	2	2
6095	69	1	0	0	0	0	1	1	0	0	1	1	0	1	0	3	4
6096	67	2	0	0	0	#NULL!	1	0	0	0	0	0	0	0	0	2	4
6098	71	2	0	1	1	1	3	#NULL!	#NULL!	#NULL!	1	1	#NULL!	0	1	3	4
6099	77	2	0	1	1	1	3	0	0	0	0	0	0	0	0	2	4
6100	67	2	0	1	0	1	1	0	0	0	1	1	0	0	0	3	2
6101	75	2	1	1	0	1	1	0	0	0	1	1	0	0	0	2	3

6103	69	1	0	0	0	1	3	0	0	1	1	0	0	0	0	1	4
6107	73	2	0	0	0	0	1	0	0	0	1	0	0	0	0	2	4
6108	66	1	0	0	0	#NULL!	1	0	1	1	0	0	0	0	1	3	4
6110	71	2	0	0	0	1	1	0	0	0	1	1	0	0	0	1	2
6111	67	1	0	0	0	1	1	0	0	1	0	0	0	0	0	1	4
6112	65	1	0	0	0	1	1	#NULL!	#NULL!	#NULL!	1	1	#NULL!	0	0	2	4
6113	70	1	0	0	0	0	1	1	1	1	1	1	0	0	0	1	4
6114	68	2	0	0	0	0	3	0	0	0	0	0	0	0	0	1	4
6115	82	1	0	0	0	1	1	2	0	0	0	0	1	0	0	1	4
6119	84	1	0	0	0	0	1	1	0	0	0	0	0	1	0	1	4
6120	75	2	0	0	0	0	1	1	0	0	1	0	0	0	0	4	1
6121	78	1	0	0	0	1	1	2	0	1	1	1	0	0	0	1	4
6122	70	2	0	0	0	0	3	#NULL!	0	0	1	0	0	0	0	2	3
6126	66	1	0	0	0	0	3	0	1	1	0	1	0	0	0	1	4
6127	79	1	1	0	0	1	1	1	1	1	1	1	1	0	0	1	4
6128	73	2	1	0	0	1	2	1	0	0	0	0	1	0	0	4	1
6130	81	2	0	1	0	0	1	0	0	0	1	0	0	0	0	2	2
6131	73	1	0	0	0	0	3	0	1	1	0	0	0	0	0	1	4
6132	68	2	0	0	0	0	1	0	0	0	0	0	0	0	0	2	2
6133	73	1	0	0	0	1	1	1	1	1	0	0	0	0	0	1	4
6134	68	2	1	0	0	#NULL!	1	0	0	0	0	1	0	0	0	2	2
6139	66	2	0	0	0	0	3	0	0	0	0	0	0	0	0	2	1
6140	66	1	0	0	0	0	1	2	0	1	0	0	0	0	1	3	4
6141	70	1	0	0	0	0	1	0	1	0	1	0	0	0	0	1	4
6143	70	1	0	0	0	0	3	2	0	1	1	0	0	0	0	2	4
6144	69	2	0	0	0	0	1	0	0	0	0	0	0	0	0	4	1
6145	73	2	0	1	1	0	3	1	0	0	1	1	0	0	0	3	2
6146	76	1	0	0	0	1	1	1	0	1	0	1	0	0	0	3	4
6148	80	1	0	0	1	1	1	2	0	1	1	1	0	0	0	3	4
6149	77	2	0	0	1	0	#NULL!	#NULL!	0	0	1	0	0	0	0	2	3
6151	72	1	0	0	0	0	1	0	0	0	0	0	0	1	0	1	4
6152	69	2	0	0	0	0	1	0	0	0	0	0	0	0	0	2	2
6153	73	2	0	1	0	1	3	0	0	0	1	0	0	0	0	2	4
6154	76	2	1	1	1	0	1	1	0	0	0	0	0	0	0	3	2
6155	69	1	0	0	0	0	1	1	1	1	1	1	0	1	0	1	4
6156	80	2	0	1	0	0	#NULL!	1	1	0	0	0	0	0	1	3	4
6159	69	2	1	1	0	1	1	1	0	0	1	0	1	0	0	4	1
6164	77	2	1	0	0	1	1	1	0	0	1	0	1	0	0	3	3
6165	77	1	0	0	0	1	1	1	1	1	0	0	0	0	1	1	4

6166	71	1	0	0	0	0	1	1	0	0	1	0	0	0	0	1	4
6168	67	2	0	1	0	1	1	0	0	0	0	0	1	0	0	2	3
6169	78	2	0	1	1	0	1	#NULL!	0	0	0	0	0	0	0	3	4
6170	82	2	0	0	1	1	3	1	0	0	0	0	0	0	0	1	4
6171	76	1	0	0	1	1	1	1	0	1	0	0	0	0	0	3	4
6172	69	2	0	0	0	0	3	1	0	0	0	0	0	0	0	3	4
6173	71	2	0	1	0	1	1	1	0	0	1	1	0	0	1	2	4
6175	67	2	0	0	0	1	1	0	0	0	0	0	1	0	1	2	4
6180	66	1	0	0	0	1	1	0	1	1	0	0	0	0	0	2	4
6182	82	2	0	1	0	1	1	2	0	0	0	0	1	0	1	2	4
6184	67	2	0	0	0	0	2	0	0	1	0	0	0	0	1	2	4
6185	66	1	0	0	0	0	3	0	0	0	1	1	0	1	0	3	4
6187	73	2	0	0	0	1	1	0	0	0	0	0	0	0	0	2	3
6188	80	2	0	1	0	1	1	0	0	0	0	1	0	0	0	2	4
6189	66	2	0	1	1	#NULL!	2	0	0	0	1	0	0	0	0	3	4
6193	76	2	0	#NULL!	0	#NULL!	1	0	0	0	1	0	1	0	1	2	4
6195	78	1	0	0	1	1	3	1	0	0	0	0	0	0	0	2	4
6199	71	1	0	0	1	#NULL!	1	2	1	1	1	1	0	0	1	3	4
6200	69	2	0	0	0	0	1	1	0	0	1	1	0	0	0	2	2
6205	73	2	0	0	0	0	1	1	0	0	0	0	0	0	0	2	4
6206	82	1	1	1	0	1	1	2	0	0	0	1	0	0	0	1	4
6207	81	2	1	0	0	1	1	0	0	#NULL!	0	1	1	0	0	3	3
6208	70	1	0	0	0	1	1	0	0	1	0	0	0	0	0	1	4
6209	73	2	0	1	0	1	1	1	0	0	0	0	1	0	0	2	3
6211	71	1	0	0	1	1	3	1	1	1	1	0	0	0	0	3	4
6213	75	1	1	0	0	1	1	2	1	0	0	0	0	1	0	2	4
6223	71	2	0	0	1	#NULL!	3	1	0	0	0	0	0	0	0	2	3
6224	81	1	0	0	0	1	1	0	0	1	0	0	1	0	0	1	4
6225	80	2	0	0	0	1	2	#NULL!	#NULL!	#NULL!	0	0	#NULL!	0	0	2	2
6228	65	2	1	0	0	1	1	1	1	0	0	0	0	#NULL!	0	4	2
6231	82	2	1	1	1	0	1	1	0	0	1	0	0	0	0	2	4
6232	80	1	0	0	1	0	1	#NULL!	0	0	0	0	0	0	0	3	4
6233	80	2	0	0	0	0	#NULL!	1	0	#NULL!	0	1	0	0	0	2	3
6234	70	1	0	0	1	1	1	1	1	1	0	1	0	1	0	2	4
6238	69	2	1	0	0	1	1	1	0	0	0	0	0	0	0	4	1
6239	69	1	0	0	0	1	3	2	0	1	0	0	0	0	0	2	4
6242	75	2	0	0	0	1	3	0	0	0	1	0	0	0	0	3	4
6243	83	1	1	0	1	0	1	1	0	1	0	0	0	0	0	1	4
6244	78	2	0	0	1	0	2	0	0	0	0	0	0	0	0	3	2

6247	65	2	0	0	0	1	1	0	0	0	0	0	0	0	1	2	3
6249	71	1	0	1	0	1	1	2	0	1	0	0	0	0	0	1	3
6251	67	1	0	0	0	1	1	1	1	1	0	0	0	0	0	3	4
6252	66	2	0	0	1	1	1	1	0	0	0	0	1	0	1	3	4
6253	72	2	0	1	1	0	1	1	0	0	0	1	0	0	0	4	4
6254	75	2	0	0	1	0	3	0	0	0	1	0	0	0	0	2	2
6255	72	1	0	0	0	0	1	1	1	1	1	1	0	1	0	1	4
6256	80	1	0	0	0	0	1	2	1	1	1	1	0	0	0	2	4
6257	77	2	0	0	0	0	3	1	0	0	0	1	0	1	0	4	1
6260	79	1	1	0	1	#NULL!	1	1	0	#NULL!	1	0	1	0	0	4	1
6263	67	2	0	1	0	1	1	0	0	0	0	0	0	0	0	4	1
6268	70	1	0	0	0	1	2	0	1	1	0	0	0	0	0	2	4
6269	78	1	0	0	0	1	3	0	1	1	1	0	0	0	0	3	4
6272	65	1	0	0	0	1	1	2	1	1	0	0	0	0	1	2	4
6274	74	2	0	1	0	1	1	0	0	0	0	0	0	0	0	3	1
6275	72	1	1	0	0	0	3	1	0	0	1	0	1	1	0	3	4
6277	79	2	1	0	1	1	1	2	0	0	0	0	1	0	0	4	1
6278	70	2	0	0	0	1	1	#NULL!	0	0	1	0	0	0	0	3	4
6279	69	1	0	0	0	1	1	0	0	1	1	0	0	0	0	1	4
6280	66	1	0	0	1	1	1	1	1	1	0	0	1	0	1	3	4
6282	68	1	1	0	0	1	1	1	0	1	0	1	1	1	0	3	4
6285	66	1	0	0	0	1	1	0	1	1	0	0	0	0	1	1	4
6286	72	1	0	0	1	1	3	#NULL!	#NULL!	#NULL!	0	0	#NULL!	0	0	2	4
6287	68	2	0	0	1	1	1	0	1	0	0	0	1	1	0	2	2
6288	75	1	0	0	1	1	1	1	1	1	1	0	0	0	0	4	4
6289	74	2	1	0	1	1	3	0	0	0	0	0	#NULL!	0	0	2	4
6293	82	1	0	0	1	0	1	1	0	1	1	0	0	0	0	2	4
6295	73	2	0	1	#NULL!	1	1	#NULL!	0	0	1	0	0	0	0	3	4
6296	78	1	0	0	0	1	1	1	1	1	0	0	0	0	0	2	4
6297	67	2	0	0	0	1	1	1	0	0	1	0	1	0	0	2	4
6299	65	2	0	0	1	1	1	0	0	0	1	0	0	0	1	2	4
6302	67	1	0	0	0	1	1	1	1	1	0	0	0	0	0	1	4
6305	73	1	0	1	1	0	1	1	1	1	0	0	0	0	0	1	4
6306	83	2	0	#NULL!	0	0	3	#NULL!	0	0	0	0	0	0	0	3	4
6307	87	2	0	1	0	0	1	1	0	0	0	0	1	0	0	2	4
6308	69	1	0	1	1	1	1	0	1	1	0	0	0	0	0	1	4
6310	74	2	0	0	0	1	1	#NULL!	0	0	1	1	0	0	0	2	4
6315	72	1	0	0	1	0	1	0	0	0	0	0	0	0	1	2	4
6317	80	1	0	1	0	1	1	0	0	0	0	1	0	0	0	1	4

6320	77	1	0	0	0	#NULL!	1	1	0	1	1	1	1	0	0	2	4
6321	76	2	0	0	1	1	1	0	0	0	1	0	0	0	0	3	4
6326	71	1	0	0	1	#NULL!	1	1	1	1	0	0	0	0	1	3	4
6327	70	2	0	1	1	1	1	1	0	0	0	1	0	0	1	2	2
6330	77	2	1	1	0	#NULL!	1	2	0	0	1	0	1	0	1	4	4
6333	78	2	1	0	1	1	3	2	0	0	0	0	0	1	0	2	1
6336	70	2	0	0	1	1	1	1	0	0	1	0	0	0	0	2	4
6342	70	1	0	0	1	1	2	#NULL!	1	0	0	0	0	0	1	3	4
6344	85	2	0	1	0	0	2	0	0	0	0	0	1	0	0	4	1
6348	75	1	0	0	1	1	1	0	1	1	0	0	0	0	0	4	1
6349	72	2	0	0	1	#NULL!	1	0	0	0	0	0	0	0	0	2	2
6352	67	1	0	1	1	1	1	1	0	1	0	0	0	0	0	3	4
6357	74	2	1	1	1	#NULL!	#NULL!	#NULL!	0	1	0	0	0	0	0	3	4
6362	75	2	0	0	0	0	1	0	1	0	1	1	0	0	0	2	1
6375	80	1	0	1	1	0	3	1	1	1	1	1	0	0	1	3	4
6383	65	2	0	1	0	1	2	0	0	0	0	0	0	0	1	1	4
6385	71	1	0	0	1	#NULL!	1	0	1	1	0	0	0	0	0	3	4
6388	67	2	0	0	1	1	1	1	0	1	0	0	0	0	0	2	2
6389	66	1	0	0	0	1	1	1	1	1	0	0	0	0	0	1	4
6391	82	2	0	1	0	#NULL!	#NULL!	#NULL!	#NULL!	#NULL!	0	0	#NULL!	0	0	2	4
6392	78	1	1	1	0	1	#NULL!	1	0	1	0	0	0	0	0	3	4
6395	73	1	0	1	0	#NULL!	3	#NULL!	#NULL!	#NULL!	1	0	#NULL!	0	0	1	1
6396	69	2	0	1	0	1	1	0	1	0	0	0	1	0	0	3	2
6399	69	1	1	0	0	0	1	0	1	1	0	0	0	0	1	2	4
6400	70	2	0	0	0	1	1	0	0	0	0	1	0	0	1	2	4
6402	79	2	1	1	1	0	1	#NULL!	0	0	1	0	1	0	0	3	2
6404	81	1	0	0	0	#NULL!	1	0	1	1	1	1	0	0	0	1	4
6405	75	2	0	0	0	1	1	0	0	0	0	0	0	0	0	3	2
6406	70	1	1	0	#NULL!	0	1	0	1	1	0	0	1	0	1	1	1
6407	70	2	0	0	1	0	3	1	0	0	1	0	0	0	0	3	4
6408	74	1	0	0	0	1	1	0	1	0	1	0	0	0	1	3	4
6415	67	2	0	1	0	0	1	0	0	0	0	0	0	0	1	1	4
6417	72	2	0	0	0	0	1	0	0	0	1	1	0	0	0	2	1
6419	74	1	0	0	0	1	1	0	0	0	1	0	0	0	0	1	4
6420	66	2	0	0	0	#NULL!	1	0	0	0	1	1	1	0	0	2	4
6423	69	1	0	0	0	1	1	1	1	1	0	0	1	0	0	2	4
6424	66	2	0	0	0	1	#NULL!	0	0	0	0	0	0	0	0	2	4
6429	67	1	0	0	1	1	1	0	1	1	0	0	0	0	0	3	1
6430	66	2	0	0	1	1	3	1	0	0	0	0	0	0	0	2	2

6437	78	2	0	1	#NULL!	1	1	1	0	1	1	1	0	0	0	4	4
6441	74	1	0	1	1	1	1	#NULL!	0	0	0	0	1	0	0	2	4
6442	78	2	0	1	0	1	1	0	0	0	1	1	0	0	0	1	4
6444	78	1	0	0	0	0	1	1	0	1	0	0	1	1	0	1	4
6445	75	2	0	0	0	0	1	1	0	0	0	0	0	0	0	4	1
6446	74	1	0	0	1	0	1	0	1	1	0	0	0	0	0	2	4
6447	72	2	0	0	1	0	3	1	0	0	1	0	0	0	0	3	3
6448	77	1	0	1	0	1	1	1	0	1	0	0	0	0	0	4	1
6452	80	2	0	0	1	1	3	1	0	0	0	0	1	0	0	2	3
6453	65	1	0	0	1	#NULL!	1	0	0	1	0	0	0	0	0	1	4
6454	78	2	1	0	0	0	1	0	0	0	0	0	0	0	0	2	2
6459	71	2	1	0	0	1	1	1	0	0	1	1	0	0	0	2	4
6460	71	2	0	0	0	0	1	1	0	0	1	0	1	0	0	3	3
6462	80	2	0	0	0	0	1	1	0	0	0	0	0	0	0	3	3
6466	74	2	0	0	1	0	1	0	1	0	1	0	0	0	0	2	2
6467	73	1	0	0	1	1	1	1	1	1	1	0	0	1	0	1	4
6471	67	2	0	1	0	1	1	0	0	1	0	0	0	0	0	2	3
6472	72	2	0	1	0	0	3	1	0	0	0	1	1	0	0	2	2
6474	66	1	0	1	0	0	1	0	1	1	0	0	0	0	0	1	4
6477	76	2	0	0	0	0	3	0	0	0	0	0	0	0	0	2	2
6478	79	1	0	0	0	0	1	1	0	0	0	0	0	1	0	2	4
6480	65	1	0	0	0	0	3	1	1	1	0	0	0	0	1	2	4
6490	76	1	1	#NULL!	1	1	#NULL!	2	0	1	0	0	1	1	0	3	1
6492	73	1	0	0	0	1	3	0	1	#NULL!	0	0	1	0	0	1	4
6493	70	2	0	0	0	1	1	1	0	0	0	0	0	0	0	3	3
6495	75	2	0	1	0	1	1	#NULL!	0	0	1	0	0	0	0	2	1
6497	65	1	0	0	0	1	1	1	0	1	1	1	1	0	0	1	4
6499	73	2	0	0	1	1	3	0	0	0	1	0	0	0	0	2	4
6503	71	1	0	0	0	1	1	1	1	1	0	0	0	0	0	1	4
6504	70	2	0	0	0	#NULL!	1	0	0	0	0	0	1	0	0	1	4
6505	69	1	0	0	1	0	1	1	0	1	0	0	#NULL!	0	0	1	4
6506	71	1	0	0	0	1	1	1	0	1	0	0	0	0	0	1	4
6507	71	2	0	0	0	1	1	1	0	0	1	0	0	0	0	2	1
6508	70	1	0	0	0	1	1	2	1	1	1	0	0	0	0	1	4
6510	73	2	0	0	1	0	1	0	0	0	0	0	0	0	0	3	4
6512	73	2	0	1	0	0	1	0	0	0	0	0	1	0	0	4	1
6513	71	1	0	0	1	1	1	0	1	1	0	0	0	0	0	2	4
6514	65	1	0	0	1	0	1	2	1	1	1	1	0	0	1	3	4
6516	76	2	0	0	0	0	2	0	0	0	1	0	0	0	0	4	1

6517	74	1	0	0	0	0	1	#NULL!	1	0	0	0	0	0	0	3	4
6518	83	1	1	0	0	1	1	1	0	1	0	0	0	0	0	3	4
6520	66	2	0	0	0	1	2	0	0	0	0	1	0	0	1	3	4
6521	78	2	1	1	1	1	1	1	0	0	1	0	0	0	1	3	4
6522	69	2	0	1	1	1	1	1	0	0	0	0	1	0	0	4	1
6524	83	1	0	0	0	0	1	1	0	0	1	1	0	0	0	4	1
6534	65	1	0	0	0	1	1	0	1	0	0	0	0	0	0	3	4
6535	70	1	0	0	0	0	1	#NULL!	#NULL!	#NULL!	0	0	#NULL!	0	1	3	4
6537	69	1	0	0	0	0	1	1	0	0	0	0	0	0	1	1	4
6540	91	2	1	#NULL!	1	#NULL!	#NULL!	0	0	0	0	1	0	0	0	2	3
6541	67	1	1	0	0	1	1	1	0	1	0	0	0	0	0	4	1
6548	67	2	0	0	0	1	3	0	0	0	0	0	0	0	0	4	1
6550	73	2	1	1	0	0	1	1	0	0	0	0	0	0	0	2	2
6552	75	1	0	0	0	0	1	1	0	1	1	1	0	0	0	1	4
6558	68	1	0	0	1	0	1	1	1	1	0	0	0	0	1	1	4
6561	83	2	0	1	0	0	3	#NULL!	#NULL!	#NULL!	0	0	#NULL!	0	0	2	1
6562	66	1	0	0	0	0	1	2	1	1	0	0	0	0	1	1	4
6563	77	1	1	0	1	1	2	0	0	0	1	0	0	0	1	3	4
6564	72	2	0	0	1	1	3	1	0	0	1	1	0	0	0	2	3
6567	71	2	0	1	0	1	1	1	1	1	1	0	0	0	0	2	4
6568	85	1	0	0	0	1	3	2	0	1	0	0	0	0	0	1	4
6569	82	2	0	0	0	1	1	1	0	0	0	0	1	0	0	2	4
6570	73	1	0	0	1	0	3	0	0	1	0	0	0	1	1	1	4
6571	72	2	0	0	1	0	1	1	0	0	0	0	0	0	1	3	4
6572	81	2	0	1	0	1	3	1	0	#NULL!	0	0	0	0	0	3	4
6573	81	2	0	1	0	0	2	1	0	0	0	0	0	0	0	4	1
6576	75	2	1	1	0	1	1	1	0	0	0	0	0	0	0	2	3
6578	72	1	0	0	0	1	1	0	0	1	1	0	0	0	0	2	4
6579	71	2	0	0	0	1	1	#NULL!	#NULL!	#NULL!	0	0	#NULL!	0	0	4	2
6580	85	1	0	0	1	1	1	0	0	1	0	0	1	0	0	2	4
6585	66	1	1	0	0	0	1	1	1	1	1	1	0	0	0	2	4
6589	73	1	0	0	0	1	1	1	0	0	0	1	1	1	0	1	4
6591	70	1	0	1	0	1	3	0	1	0	0	0	1	1	0	3	4
6592	75	2	0	1	0	1	3	1	0	0	0	1	0	0	0	3	2
6593	70	2	0	0	0	1	3	0	0	0	0	0	0	0	0	4	4
6594	79	1	0	0	0	0	1	0	0	1	0	0	0	0	0	1	4
6597	65	1	1	0	0	0	#NULL!	1	1	0	0	0	1	1	0	1	4
6599	70	1	0	0	0	0	1	0	0	1	1	1	0	0	0	2	4
6600	76	1	1	0	0	1	1	0	0	1	1	1	1	0	0	2	4

6601	71	2	0	0	0	1	#NULL!	0	0	0	0	0	0	1	0	4	1
6607	71	1	0	0	0	1	3	1	0	1	0	0	0	0	0	1	4
6608	74	2	0	0	1	1	2	1	0	0	1	0	1	0	0	3	4
6609	74	1	1	0	0	1	1	2	1	1	1	0	1	1	0	1	4
6613	77	2	0	1	0	1	1	#NULL!	0	0	1	0	0	0	0	3	4
6614	82	2	0	1	1	1	1	0	0	0	1	0	1	0	0	3	3
6618	69	2	0	0	0	1	2	1	0	0	1	0	0	0	0	2	2
6620	66	1	0	0	1	0	#NULL!	0	1	1	0	0	0	0	1	1	4
6621	65	2	0	0	0	1	2	0	0	1	1	0	0	0	0	2	4
6622	77	2	1	1	1	1	1	0	0	0	0	1	1	0	0	3	2
6624	73	2	0	0	0	1	3	2	0	0	1	1	0	0	0	2	2
6625	74	2	0	0	0	1	3	1	0	0	0	0	1	0	0	2	2
6626	72	1	1	0	0	1	1	1	1	1	0	0	0	1	0	3	4
6627	67	1	0	0	0	1	1	1	0	0	0	0	0	0	1	2	4
6628	68	1	0	0	0	1	3	2	0	1	0	0	1	1	0	2	4
6629	68	2	0	0	0	0	1	0	0	0	0	0	0	0	0	2	3
6630	66	2	0	1	0	1	1	0	0	0	1	0	0	0	0	2	4
6631	90	2	0	1	0	0	1	#NULL!	0	0	0	0	0	1	0	2	2
6632	81	1	0	1	0	1	3	1	1	1	1	0	0	0	0	3	4
6635	71	1	0	0	0	1	1	2	0	1	0	0	0	1	0	2	4
6641	72	2	0	0	1	1	1	0	0	0	0	0	0	0	0	2	4
6643	67	2	0	1	0	1	1	0	0	0	0	0	1	0	1	2	4
6644	72	1	0	0	1	1	1	1	1	0	1	1	0	0	0	2	4
6645	67	2	0	0	0	1	1	1	0	0	0	0	0	0	0	2	2
6647	73	1	0	0	0	0	1	0	1	0	1	0	0	0	0	2	4
6648	70	2	0	0	0	0	1	1	0	0	0	0	0	0	1	2	1
6649	72	1	0	#NULL!	0	#NULL!	1	#NULL!	#NULL!	#NULL!	0	0	#NULL!	0	0	1	4
6650	71	2	0	0	0	0	1	0	0	0	0	0	0	0	0	2	2
6656	71	1	0	0	0	0	1	0	1	1	1	0	0	0	0	1	4
6657	66	2	0	0	0	0	3	0	0	0	0	0	0	0	0	2	2
6663	78	2	0	0	0	1	3	0	0	1	0	1	1	0	0	3	2
6664	76	1	0	0	0	#NULL!	1	2	0	1	0	0	#NULL!	0	0	2	4
6669	75	2	1	0	0	0	2	1	0	0	0	0	0	0	0	2	2
6673	72	2	0	0	1	1	1	1	0	0	0	0	1	0	0	3	2
6674	74	2	0	1	0	0	1	1	0	0	1	1	0	0	0	4	1
6677	71	2	0	0	0	1	3	1	0	0	1	0	1	0	0	2	2
6678	70	1	0	0	0	1	1	1	0	1	0	0	0	0	0	1	4
6679	74	1	0	0	0	1	3	0	1	0	0	1	1	0	0	3	4
6680	67	2	0	0	1	1	1	0	0	0	0	1	0	0	0	2	4

6684	72	2	1	1	1	1	1	1	0	1	0	0	0	0	0	2	3
6685	68	2	0	1	0	0	1	#NULL!	#NULL!	#NULL!	0	0	#NULL!	0	0	2	2
6686	77	1	0	0	1	0	#NULL!	0	0	1	0	1	0	0	1	3	4
6687	70	2	0	#NULL!	1	#NULL!	1	#NULL!	#NULL!	#NULL!	0	1	#NULL!	0	0	2	3
6688	77	1	1	0	1	1	2	0	0	1	0	0	1	0	0	1	4
6689	74	2	0	0	1	#NULL!	3	#NULL!	#NULL!	#NULL!	0	0	#NULL!	0	0	3	1
6690	65	1	0	0	0	0	1	#NULL!	#NULL!	#NULL!	0	1	#NULL!	0	1	1	4
6692	66	2	0	0	0	1	2	1	0	0	0	0	1	0	0	4	1
6693	66	2	0	1	0	0	1	0	0	0	0	0	0	0	1	3	4
6696	79	2	0	0	1	0	3	#NULL!	0	0	0	0	0	0	0	4	1
6698	70	2	0	1	0	1	1	1	0	0	0	0	0	0	1	1	4
6699	68	1	0	0	1	0	1	0	0	0	0	0	0	1	0	3	4
6701	74	2	1	0	0	1	1	1	0	0	1	0	0	0	0	4	1
6703	79	2	0	1	0	1	3	#NULL!	0	0	0	1	0	0	0	3	4
6704	72	1	0	0	0	#NULL!	1	1	0	0	0	0	0	0	0	1	4
6705	66	2	0	0	0	0	2	0	0	0	0	0	0	0	0	1	1
6706	83	2	0	1	0	0	2	1	0	0	1	1	0	0	0	4	1
6708	74	2	0	1	0	0	2	0	0	0	0	0	0	0	0	4	4
6709	73	2	0	1	1	0	3	0	1	0	0	0	0	0	0	3	4
6710	74	1	0	0	0	1	1	0	1	0	0	1	0	1	1	1	4
6712	67	2	0	1	1	1	1	0	0	0	1	0	0	0	0	3	2
6713	67	2	0	1	1	0	2	#NULL!	#NULL!	#NULL!	#NULL!	#NULL!	#NULL!	0	1	2	4
6717	74	2	0	0	1	1	3	2	0	1	0	0	1	0	0	1	4
6720	78	2	0	1	0	0	1	1	0	0	0	0	0	0	0	2	3
6721	68	1	0	1	0	1	1	0	1	1	1	0	0	0	1	2	4
6722	71	1	0	0	0	1	1	2	0	1	0	0	0	0	0	2	4
6723	68	2	0	0	0	0	1	0	0	0	1	0	0	0	0	2	1
6724	69	2	0	0	0	0	3	1	0	0	0	0	1	0	0	2	4
6725	69	1	0	0	0	0	1	0	1	1	0	0	0	0	1	1	4
6726	69	1	0	0	0	1	3	2	1	1	1	0	0	0	0	2	4
6731	65	1	0	0	1	1	1	1	1	1	1	1	0	0	0	1	4
6732	70	1	0	0	0	0	3	2	0	1	0	0	0	0	0	2	4
6734	72	2	0	0	1	1	1	1	0	0	0	0	0	0	0	3	2
6743	82	2	0	1	0	0	1	1	0	0	0	0	0	0	0	1	4
6745	71	2	0	1	0	0	3	1	0	0	0	0	0	0	0	2	1
6748	86	1	1	0	1	1	1	0	0	1	0	0	1	1	0	1	4
6749	77	2	0	0	1	1	1	0	0	0	0	0	1	0	0	3	1
6750	70	1	0	0	0	1	1	0	0	0	0	0	1	0	0	1	4
6751	66	2	0	0	0	1	2	0	0	0	0	0	1	0	0	2	1

6752	68	2	0	0	0	0	1	0	1	0	0	0	0	0	0	4	1
6756	77	2	0	1	0	1	1	0	0	1	1	0	0	0	0	2	3
6759	65	1	0	0	0	1	2	#NULL!	1	1	0	0	0	0	0	3	4
6761	73	1	0	0	0	0	2	0	0	0	1	0	0	0	1	2	4
6762	70	1	0	0	0	0	1	#NULL!	#NULL!	#NULL!	0	0	#NULL!	0	0	1	4
6764	71	1	0	0	1	1	3	1	1	0	1	1	0	0	0	2	4
6765	67	2	0	0	0	0	1	2	0	0	0	0	0	0	1	2	4
6766	75	1	1	0	1	1	3	#NULL!	0	0	0	0	0	0	1	1	4
6768	78	2	0	1	0	1	1	2	0	0	0	0	0	0	0	2	4
6771	76	1	0	0	1	1	3	0	1	1	0	0	0	0	0	3	4
6775	66	2	0	0	0	1	1	0	0	0	1	1	0	0	1	2	1
6776	72	1	0	0	0	#NULL!	1	0	0	1	1	0	0	0	0	1	4
6778	66	2	0	0	0	1	1	1	0	0	0	0	1	0	0	2	2
6779	65	1	0	0	0	1	1	2	1	1	0	0	0	0	1	2	4
6780	87	1	0	0	1	0	1	#NULL!	#NULL!	#NULL!	1	0	#NULL!	0	0	3	1
6782	81	2	0	1	0	0	1	1	0	0	1	1	0	0	0	1	4
6786	74	1	0	0	0	1	3	1	1	1	0	0	0	0	0	1	4
6787	71	2	0	0	1	1	1	1	0	0	0	0	0	0	0	3	3
6789	65	2	0	1	1	1	1	0	0	1	0	0	0	0	1	3	4
6790	66	2	1	0	1	1	3	0	0	0	0	1	0	0	1	3	2
6791	72	1	0	0	0	1	2	0	1	1	0	0	0	0	0	1	4
6792	71	2	0	0	1	1	2	#NULL!	0	#NULL!	0	0	0	0	0	4	4
6795	66	1	0	0	0	1	1	0	0	0	0	0	1	1	0	1	4
6797	68	2	1	0	0	1	3	2	0	0	1	0	1	0	0	2	4
6799	67	2	0	0	0	#NULL!	1	0	0	0	0	0	0	0	0	2	3
6803	65	1	0	0	0	0	1	0	0	1	0	1	0	0	1	2	4
6804	72	2	0	1	1	1	1	1	0	0	0	0	0	0	0	3	1
6805	76	2	0	1	0	1	1	1	0	0	0	0	0	0	0	2	3
6806	78	1	0	0	0	0	1	0	0	1	0	1	0	0	0	1	4
6807	73	2	0	0	0	0	1	1	0	0	0	1	0	0	0	2	2
6812	73	2	0	0	1	1	2	0	0	0	1	0	0	0	0	2	4
6815	75	2	0	1	0	1	1	0	0	0	0	0	1	0	0	1	3
6816	75	1	0	0	0	1	3	2	0	0	0	0	0	0	1	1	4
6817	72	2	0	0	0	1	3	1	0	0	0	0	0	0	0	3	4
6819	72	2	0	0	0	1	1	0	0	0	0	0	0	0	0	2	3
6821	71	1	0	0	0	1	1	1	1	1	1	0	0	1	0	3	4
6825	85	1	0	0	1	1	3	1	1	1	1	1	0	0	0	3	4
6827	71	1	0	0	1	1	1	1	0	0	1	1	0	0	0	4	1
6828	66	2	0	0	0	1	#NULL!	0	0	0	0	1	0	0	0	1	4

6829	89	2	1	1	0	1	1	0	0	0	0	0	1	0	0	2	4
6832	80	1	0	0	0	0	1	#NULL!	0	0	0	0	0	0	0	3	4
6833	77	2	0	0	#NULL!	0	1	0	0	0	1	0	0	0	0	4	4
6836	77	1	1	0	0	1	1	0	1	1	0	1	0	0	0	3	2
6837	74	2	0	0	0	1	1	0	0	0	1	0	0	0	0	2	2
6838	73	1	0	0	0	1	1	1	0	1	0	0	0	0	0	1	4
6841	81	2	0	0	0	0	1	2	0	1	0	0	0	0	0	2	4
6843	65	1	1	0	1	1	1	1	0	1	0	0	0	0	1	3	4
6847	65	1	0	0	0	1	1	1	0	0	0	0	0	0	0	1	4
6848	66	1	0	0	0	1	1	0	0	1	0	1	0	0	1	4	4
6850	76	2	0	1	0	1	1	1	0	1	0	0	1	0	0	1	4
6851	74	2	0	1	0	1	2	0	0	1	0	1	1	0	0	2	2
6855	66	1	0	0	0	0	1	0	0	1	0	1	0	0	0	2	4
6856	65	1	0	0	0	1	1	0	0	0	0	0	0	0	1	2	4
6857	66	2	0	0	0	1	1	0	0	0	0	0	0	0	1	3	4
6858	66	1	0	0	0	1	1	0	1	1	1	0	0	1	1	1	4
6860	71	1	0	0	0	0	1	0	0	1	1	1	0	1	0	2	4
6861	66	2	0	0	0	0	1	1	0	0	1	0	0	0	0	2	2
6862	80	2	1	1	1	0	3	1	0	0	1	0	0	0	0	2	3
6864	76	1	0	0	0	1	3	1	1	0	1	0	1	0	0	1	4
6865	70	2	0	1	0	1	1	1	0	1	0	1	0	0	0	1	3
6867	81	2	1	0	0	0	1	1	0	0	0	0	0	0	0	2	2
6868	79	1	0	0	1	0	1	0	0	1	0	0	0	0	0	1	1
6869	67	2	0	1	0	0	1	1	1	1	1	0	0	0	0	2	4
6871	76	2	0	0	0	1	1	0	0	0	0	0	1	0	0	3	3
6874	84	1	0	0	0	0	3	1	0	1	0	0	1	1	0	1	4
6875	82	2	0	0	0	0	1	1	0	0	0	0	0	0	0	2	4
6878	69	2	0	0	0	#NULL!	1	0	0	0	1	1	1	0	0	2	2
6879	65	1	0	0	0	0	1	1	1	1	0	0	0	0	1	1	4
6880	69	1	0	0	0	#NULL!	1	1	1	1	1	1	0	0	0	1	4
6881	67	2	1	0	0	#NULL!	1	1	1	0	0	0	1	0	0	4	2
6884	68	1	0	0	0	1	1	0	1	1	1	0	0	0	1	1	4
6886	67	2	0	1	0	1	3	1	0	0	0	0	0	0	0	4	1
6889	73	2	1	0	0	1	1	0	0	0	0	0	1	0	0	2	3
6891	72	2	0	0	0	1	1	1	0	0	1	1	0	0	0	2	4
6892	65	1	0	1	0	1	1	0	0	1	1	0	1	0	0	1	4
6893	87	1	0	0	0	1	1	1	1	1	0	0	0	0	0	2	4
6896	68	2	0	1	0	0	1	0	0	0	0	0	0	0	0	2	3
6897	78	1	1	0	0	1	1	2	0	1	0	0	0	0	0	4	1

6898	75	2	0	0	0	1	1	1	#NULL!	#NULL!	0	0	#NULL!	0	1	2	4
6901	69	1	0	0	0	1	1	1	1	1	1	1	0	0	1	3	4
6902	66	2	0	0	0	1	1	0	1	0	0	0	0	0	0	3	3
6904	72	1	0	0	1	1	3	0	0	0	0	0	0	1	0	1	4
6907	67	1	0	0	0	0	1	0	1	1	0	0	0	0	1	1	4
6908	65	2	0	0	0	#NULL!	1	0	0	0	0	0	0	0	1	2	4
6909	84	1	0	1	0	0	3	2	0	0	0	0	1	0	0	1	4
6910	79	2	0	1	1	#NULL!	#NULL!	#NULL!	0	0	0	1	0	0	0	4	2
6917	71	2	0	#NULL!	0	1	3	0	0	0	0	0	0	0	0	2	1
6919	70	1	0	0	1	1	1	0	1	1	0	0	1	0	0	2	4
6920	65	2	0	0	1	1	1	0	0	0	0	0	0	0	0	2	4
6922	76	2	1	0	#NULL!	1	#NULL!	#NULL!	#NULL!	#NULL!	0	1	#NULL!	1	0	2	2
6924	76	1	1	0	1	1	1	1	1	1	1	1	0	0	0	3	1
6925	70	2	0	0	1	1	1	0	0	0	0	0	1	0	1	4	4
6926	65	2	0	1	0	1	1	0	0	0	0	0	1	0	0	2	2
6927	75	2	0	0	0	1	1	0	0	0	0	0	1	0	0	4	1
6928	74	1	0	0	0	1	1	1	1	1	1	1	0	0	0	2	4
6932	74	2	0	1	1	0	1	2	0	0	1	0	0	0	0	4	1
6934	78	2	0	0	1	1	1	#NULL!	0	0	0	1	0	0	0	3	3
6935	71	1	0	0	1	0	1	0	0	1	0	0	0	0	0	3	4
6936	73	1	0	0	1	1	1	0	0	1	0	1	0	0	0	3	4
6937	71	2	0	0	0	#NULL!	1	2	0	0	0	0	0	0	0	3	4
6939	79	1	1	0	0	1	3	1	1	1	1	1	0	0	0	1	4
6940	76	2	0	0	0	1	1	1	0	0	1	1	0	0	0	3	3
6941	85	1	0	0	0	0	3	0	1	1	1	1	0	0	0	1	4
6945	73	2	0	0	0	1	2	0	0	0	0	0	0	0	0	4	1
6947	68	2	0	0	0	1	1	1	0	0	0	1	0	0	0	2	2
6954	66	1	0	0	#NULL!	1	1	0	0	1	1	0	0	0	1	2	4
6956	68	1	0	0	0	0	1	0	0	1	1	0	0	0	1	1	4
6957	65	2	0	0	0	1	1	0	0	0	1	0	0	0	0	2	3
6959	80	2	1	0	0	1	1	2	0	0	0	0	1	0	0	2	1
6960	80	1	0	0	1	1	1	1	1	0	0	0	1	0	0	3	4
6961	86	1	0	0	1	1	1	#NULL!	#NULL!	#NULL!	1	1	#NULL!	0	0	3	4
6962	81	2	1	0	1	1	3	1	0	0	0	0	1	0	0	2	4
6963	78	1	1	0	1	1	1	1	0	0	0	0	0	0	1	3	4
6965	73	2	0	#NULL!	0	#NULL!	1	0	0	0	0	0	1	0	0	4	1
6967	68	1	0	0	0	0	1	0	1	1	1	1	0	0	1	3	4
6971	71	2	0	0	0	1	1	1	1	1	1	1	0	0	0	1	1
6972	78	1	0	1	0	1	1	0	1	1	0	0	0	0	0	3	4

6974	77	2	0	0	0	0	3	1	0	0	0	0	0	0	0	3	4
6975	70	1	1	0	0	1	1	1	1	1	0	0	1	0	0	3	4
6977	66	1	0	1	1	1	1	0	0	1	0	0	1	0	0	2	4
6982	79	2	1	0	1	1	1	1	0	#NULL!	0	0	0	0	0	3	3
6985	69	1	0	0	0	1	1	1	0	0	0	0	0	0	1	1	4
6986	85	2	1	#NULL!	1	#NULL!	3	2	0	0	0	0	1	0	0	4	2
6987	72	1	0	0	0	#NULL!	1	0	1	0	0	1	1	0	0	3	3
6988	70	2	0	0	0	1	1	#NULL!	0	0	1	0	0	0	1	2	2
6989	79	2	0	1	0	0	1	0	0	0	1	0	0	0	0	2	2
6990	66	2	0	1	0	1	1	1	0	0	1	0	0	0	1	4	1
6994	72	2	0	1	0	1	1	1	0	0	0	0	0	0	0	3	4
6995	71	2	0	1	0	0	1	0	0	0	1	0	0	0	0	2	1
6999	72	1	0	0	0	1	1	1	0	1	1	1	0	0	0	2	4
7002	76	2	0	1	0	1	#NULL!	0	0	0	1	0	0	0	1	2	3
7003	66	1	0	0	0	0	1	1	0	1	1	0	0	0	1	2	4
7004	66	2	0	0	0	0	1	1	0	0	0	0	0	0	0	3	4
7005	73	1	1	0	0	1	1	2	0	0	0	0	1	1	0	2	4
7006	71	2	0	0	0	1	1	1	0	0	0	0	0	0	0	3	4
7007	84	1	0	0	0	0	1	0	1	1	0	1	0	1	0	2	4
7008	77	2	0	0	0	1	1	0	0	0	1	0	1	0	0	4	1
7011	80	2	0	1	0	1	2	0	0	1	0	0	1	0	0	3	1
7012	70	1	0	0	0	0	1	0	1	0	0	1	0	0	0	2	4
7013	84	2	0	1	0	0	1	1	0	0	0	0	#NULL!	0	0	2	4
7016	82	1	0	0	1	0	1	2	1	1	0	1	1	1	0	1	4
7018	77	2	1	1	0	0	1	#NULL!	#NULL!	#NULL!	0	0	#NULL!	0	0	4	3
7021	82	2	1	0	1	1	1	0	0	0	0	0	1	1	0	1	3
7022	75	1	1	0	0	1	1	0	0	1	0	0	0	0	0	2	4
7023	78	1	0	0	0	0	1	1	0	1	1	0	0	0	0	1	4
7024	71	2	0	0	0	0	2	0	0	0	0	0	0	0	0	4	1
7027	69	2	0	0	1	0	3	1	0	0	0	0	0	0	0	3	2
7030	69	2	0	0	1	#NULL!	1	#NULL!	0	0	1	0	0	0	1	1	4
7033	72	2	0	1	0	1	1	0	0	0	1	0	0	0	0	2	2
7034	67	1	0	0	0	1	1	0	0	0	0	1	0	0	1	3	4
7036	68	2	0	0	0	0	1	1	0	0	0	0	0	0	0	4	1
7037	77	1	0	0	0	1	1	1	0	1	0	0	0	0	0	1	4
7038	70	2	0	0	0	1	1	2	0	0	0	0	0	0	0	2	2
7039	76	1	0	0	0	0	1	1	1	1	0	0	0	0	0	1	4
7040	73	2	0	0	0	#NULL!	1	1	0	0	1	0	0	0	0	2	1
7041	68	2	0	1	1	1	1	0	0	0	0	0	1	0	1	3	4

7042	73	1	1	0	0	1	3	0	0	0	0	1	0	0	0	4	1
7043	84	2	1	1	0	0	3	1	0	0	0	0	0	0	0	3	2
7047	83	2	0	1	#NULL!	0	1	1	0	0	1	0	0	0	0	3	3
7048	72	1	1	0	0	1	1	2	0	1	1	1	0	1	0	1	4
7050	82	1	0	1	0	0	1	1	1	0	1	1	0	0	0	1	4
7054	75	1	0	#NULL!	0	#NULL!	1	#NULL!	#NULL!	#NULL!	0	0	#NULL!	0	0	2	4
7055	72	2	0	0	0	0	3	1	0	0	0	0	0	0	0	2	1
7060	79	2	0	0	0	0	3	1	0	0	1	0	0	0	0	3	4
7065	65	1	0	0	1	1	2	0	0	1	1	0	0	0	0	3	4
7068	74	1	0	1	0	1	1	1	0	1	0	0	0	0	0	2	4
7069	75	2	0	0	0	1	1	0	0	0	0	0	0	0	0	4	3
7072	73	2	0	0	1	1	1	0	0	0	0	0	0	0	0	3	2
7073	65	1	0	0	1	1	2	0	0	0	0	0	0	0	0	3	4
7074	78	2	0	1	0	1	1	0	0	0	1	0	0	0	0	2	4
7078	66	2	0	0	0	1	3	#NULL!	0	0	1	0	0	0	0	2	2
7080	71	2	0	1	0	0	1	0	0	0	1	0	0	0	1	2	3
7083	80	2	0	0	0	0	#NULL!	0	0	0	1	0	0	0	0	2	3
7086	87	1	0	0	1	1	1	1	0	1	1	1	0	0	0	1	4
7088	75	1	1	0	0	0	3	2	0	0	0	1	0	0	0	3	4
7089	73	2	0	0	0	0	1	1	0	0	0	1	0	0	0	3	3
7092	86	2	0	0	0	1	2	2	0	0	1	1	1	0	0	4	1
7093	71	1	1	0	0	1	3	1	1	1	0	0	0	0	1	3	4
7094	70	1	0	0	1	1	1	2	0	1	0	0	0	0	0	2	4
7095	69	2	0	0	0	1	1	0	0	0	0	0	0	0	1	1	4
7098	75	1	0	0	0	1	3	2	0	0	0	0	0	0	0	3	4
7099	69	2	0	0	0	1	1	0	0	0	0	1	#NULL!	0	0	2	1
7100	84	2	0	1	0	0	1	2	0	0	0	0	0	0	0	2	4
7101	66	1	0	0	0	1	3	0	1	0	1	0	0	0	1	2	4
7107	67	2	0	0	0	1	1	1	1	0	0	0	1	0	0	2	4
7114	80	2	0	1	0	0	1	#NULL!	0	0	0	0	0	0	0	2	3
7116	67	2	0	0	1	1	3	1	0	0	0	0	0	0	0	3	2
7117	71	1	0	0	0	1	3	#NULL!	#NULL!	#NULL!	0	1	#NULL!	0	1	2	4
7119	73	2	0	0	0	0	1	1	0	0	1	1	0	0	0	2	4
7120	73	1	0	0	0	0	1	0	0	0	1	1	0	0	0	1	4
7121	81	1	0	0	0	0	1	0	#NULL!	#NULL!	1	0	#NULL!	0	0	1	4
7122	75	2	0	#NULL!	0	#NULL!	1	#NULL!	#NULL!	#NULL!	1	0	#NULL!	0	0	4	1
7123	79	1	0	0	0	1	1	1	1	1	0	0	0	0	0	1	4
7130	69	1	0	0	0	1	1	0	1	1	0	0	1	0	0	1	4
7133	78	2	0	0	0	#NULL!	3	1	0	0	0	0	0	0	0	1	1

7134	70	2	1	1	1	1	1	1	0	0	0	0	0	0	0	3	3
7135	66	2	0	1	0	1	2	0	0	1	0	1	0	0	0	2	4
7136	72	2	0	1	1	0	1	0	0	0	1	0	0	0	0	3	2
7137	66	2	0	1	1	1	#NULL!	#NULL!	0	0	0	0	0	0	1	3	4
7138	67	2	0	0	0	1	1	1	0	0	1	0	0	0	0	2	2
7140	66	2	1	0	0	1	3	2	0	0	0	0	0	0	1	2	3
7141	66	2	0	1	0	1	3	1	1	0	0	1	1	0	0	2	4
7142	93	2	0	1	1	0	1	2	0	0	1	0	0	0	0	1	4
7143	66	2	0	1	0	0	1	2	0	0	0	0	0	0	0	2	3
7144	65	2	0	0	0	0	1	0	0	0	0	0	0	0	0	2	4
7145	78	1	1	0	1	1	1	2	0	0	0	0	#NULL!	0	0	1	1
7147	89	2	0	1	0	0	1	2	0	0	0	0	0	0	0	2	2
7149	66	2	0	0	0	0	1	0	0	0	0	0	0	0	0	3	4
7150	65	1	1	0	0	1	1	1	0	1	1	0	1	1	0	1	4
7153	81	1	0	0	0	0	3	2	1	1	1	0	0	0	0	1	4
7158	74	1	1	0	1	1	1	1	1	1	0	1	0	0	1	1	4
7159	70	2	0	0	0	1	1	0	0	0	1	0	0	0	1	2	4
7160	83	2	0	1	0	0	1	1	0	0	0	0	0	0	0	2	4
7161	75	2	0	1	0	1	1	0	0	1	0	0	0	0	0	2	2
7165	75	2	0	1	0	0	1	1	0	0	0	0	0	0	0	2	2
7167	65	2	0	1	0	1	1	0	1	1	0	0	0	0	0	1	3
7168	73	1	0	0	1	0	1	1	1	1	1	1	0	0	0	3	4
7169	70	2	0	0	1	0	1	1	0	0	1	0	0	0	0	3	4
7170	72	1	1	0	0	#NULL!	1	2	0	1	0	1	0	0	0	1	4
7171	72	1	0	0	0	1	1	0	0	1	1	1	0	0	0	1	4
7173	75	1	0	0	0	1	3	0	1	0	0	0	0	0	0	2	4
7174	71	2	0	0	0	1	3	0	1	0	0	0	0	0	0	1	1
7178	79	1	0	0	0	1	1	0	0	0	0	0	0	0	0	1	4
7182	66	2	0	1	0	1	1	2	0	0	0	0	1	0	0	2	4
7187	73	2	0	1	1	1	2	0	0	1	0	0	0	0	1	2	4
7194	74	1	0	0	1	1	3	2	0	0	1	0	0	0	0	1	4
7195	72	2	1	0	1	1	1	0	0	0	0	0	0	0	0	1	3
7196	80	2	0	1	1	0	1	1	0	0	0	0	0	1	0	3	2
7197	70	1	0	0	0	1	1	0	1	1	0	0	0	0	1	2	4
7198	65	2	0	1	0	#NULL!	1	0	0	1	0	0	0	0	0	2	4
7199	71	2	0	0	0	1	2	0	0	0	1	0	0	0	0	3	3
7205	72	1	0	0	0	0	2	0	1	1	0	0	1	0	1	1	4
7207	66	1	0	0	0	0	3	#NULL!	0	0	1	0	0	0	0	1	4
7208	74	1	0	0	0	1	1	2	0	1	0	0	1	0	0	1	4

7209	68	2	0	0	0	1	1	1	0	0	0	0	0	0	1	1	4
7210	66	1	0	1	0	1	1	1	0	1	0	0	1	0	1	1	4
7213	72	2	0	1	0	1	1	0	0	0	0	0	0	0	1	2	4
7216	69	1	0	0	1	1	1	0	0	1	0	0	0	0	1	1	4
7218	72	1	0	0	1	1	1	1	0	1	0	0	0	0	1	3	4
7219	71	2	1	0	1	1	1	0	0	0	0	0	1	0	0	1	3
7224	74	2	0	0	1	1	3	1	0	0	0	0	0	0	0	3	2
7225	72	1	0	0	1	1	1	1	1	0	1	0	1	0	0	2	4
7230	72	2	0	0	1	0	1	0	0	0	1	1	0	0	0	3	3
7233	75	2	0	1	0	0	1	0	0	0	1	0	0	0	0	1	4
7235	66	1	0	1	0	1	1	1	1	1	0	0	0	0	0	1	4
7237	74	2	1	0	0	1	1	1	0	0	0	1	0	0	0	2	3
7238	70	1	0	0	1	1	1	1	0	0	0	0	0	0	0	1	4
7239	68	2	0	0	0	#NULL!	1	0	0	0	0	0	1	0	0	3	4
7247	65	1	0	0	0	1	1	1	0	0	1	1	1	0	1	1	4
7248	86	1	0	0	0	0	3	1	0	1	1	1	0	0	0	1	1
7254	70	1	0	0	0	1	3	1	0	1	0	1	1	1	0	1	4
7255	65	2	0	0	0	1	2	0	0	0	0	0	0	0	0	1	4
7258	67	1	0	0	1	1	#NULL!	1	0	1	0	0	1	0	0	1	3
7259	90	1	1	0	1	1	1	0	0	1	0	0	1	0	0	3	4
7260	84	2	1	0	0	1	1	0	0	0	0	1	1	0	0	3	3
7264	75	1	0	0	0	1	1	0	0	0	0	0	0	0	0	2	4
7265	73	2	0	0	1	#NULL!	3	0	0	0	1	0	1	0	0	4	1
7266	70	2	0	0	0	0	3	1	0	0	0	0	1	0	0	2	3
7267	66	1	0	0	0	1	3	1	0	0	1	0	0	0	1	3	4
7268	78	2	0	1	1	0	1	1	0	0	0	0	1	0	0	2	3
7269	84	2	0	#NULL!	0	1	1	2	0	0	0	0	1	1	0	3	3
7270	76	1	0	0	0	1	1	2	0	0	0	0	0	1	0	3	4
7271	70	2	0	0	0	1	3	1	0	0	0	0	0	0	0	3	4
7278	78	2	0	1	0	0	1	#NULL!	#NULL!	#NULL!	0	1	#NULL!	0	0	4	1
7279	73	2	1	0	0	#NULL!	#NULL!	0	0	0	0	0	1	0	0	4	1
7281	70	2	0	1	1	0	1	0	0	0	0	1	0	0	0	3	3
7283	78	1	0	1	1	1	3	1	0	1	0	1	1	0	0	1	1
7289	69	1	0	0	0	1	1	0	0	1	0	0	0	0	0	3	4
7290	66	2	0	0	0	1	1	0	0	0	1	0	0	0	0	2	2
7292	68	2	0	0	0	0	1	0	0	0	1	1	0	0	0	3	2
7296	71	1	0	0	1	1	1	1	1	0	1	0	0	0	0	3	4
7297	70	2	0	0	0	1	1	1	0	0	0	0	0	0	0	2	2
7298	66	1	0	0	0	1	1	0	0	1	0	0	1	1	0	2	1

7299	65	2	0	0	0	0	1	1	0	0	0	0	0	0	0	4	1
7300	70	2	0	#NULL!	0	#NULL!	1	0	0	0	1	1	0	0	0	2	4
7304	67	1	0	0	0	1	1	1	0	1	0	1	0	0	1	3	4
7312	71	1	0	0	0	0	1	1	0	1	0	0	0	1	0	1	4
7313	66	2	0	0	0	0	1	1	0	0	1	1	1	0	0	2	1
7314	82	2	1	1	1	1	1	#NULL!	#NULL!	#NULL!	0	0	#NULL!	0	0	4	1
7318	66	2	0	0	0	0	1	0	0	0	1	0	0	0	0	2	4
7319	66	1	0	0	0	#NULL!	3	0	0	1	1	0	0	0	0	2	4
7320	73	1	0	0	0	0	3	0	1	0	0	0	0	0	1	3	3
7324	65	2	0	1	0	0	2	#NULL!	#NULL!	#NULL!	0	0	#NULL!	0	0	1	4
7325	73	1	0	0	1	1	3	1	1	1	0	0	0	0	0	3	4
7332	71	2	1	0	1	1	1	1	1	1	0	1	0	0	0	2	4
7335	87	2	0	1	0	#NULL!	#NULL!	0	0	0	0	1	0	0	0	3	4
7337	67	2	0	0	0	#NULL!	1	#NULL!	#NULL!	#NULL!	0	0	#NULL!	0	0	2	4
7338	66	2	0	0	1	1	1	0	0	0	0	0	0	0	1	1	4
7339	70	1	1	0	0	1	1	1	1	1	0	1	0	0	0	1	4
7340	69	2	0	0	0	1	1	1	0	0	0	0	0	0	0	3	3
7342	73	1	0	0	1	1	1	0	1	1	0	1	1	0	0	1	4
7343	72	2	0	0	0	1	1	2	0	1	0	1	1	0	0	3	4
7346	71	1	0	0	1	1	#NULL!	#NULL!	1	1	0	0	0	0	0	3	4
7353	77	2	0	1	0	1	1	0	0	0	1	0	0	0	0	2	4
7360	69	1	0	0	0	1	3	2	0	1	0	0	0	1	0	3	4
7361	67	2	0	0	0	1	1	0	0	0	0	0	0	0	0	2	2
7362	78	2	0	1	0	1	1	2	0	0	0	0	0	0	0	2	4
7364	68	1	0	0	0	1	1	1	1	1	1	0	0	0	0	1	4
7365	67	2	0	0	1	1	1	0	0	0	0	0	0	0	0	3	3
7366	75	1	0	0	1	1	1	#NULL!	0	1	0	0	1	0	0	3	4
7367	73	2	0	0	1	1	3	2	0	0	0	0	1	0	0	2	4
7368	80	1	0	1	1	1	1	#NULL!	#NULL!	#NULL!	0	0	#NULL!	0	0	3	4
7369	81	1	1	0	0	1	1	1	1	1	0	0	#NULL!	1	0	2	4
7370	78	2	0	0	0	1	1	0	0	0	0	1	1	0	0	2	1
7371	92	2	0	#NULL!	1	0	#NULL!	1	#NULL!	0	0	0	0	0	0	4	4
7373	82	2	0	1	0	0	1	1	0	0	0	0	0	0	0	2	1
7374	65	1	0	1	0	0	1	0	1	1	1	1	0	0	0	1	4
7375	72	1	0	#NULL!	0	0	3	#NULL!	#NULL!	#NULL!	1	1	#NULL!	0	1	2	2
7378	69	1	0	0	0	1	1	2	1	1	1	1	0	0	1	1	4
7382	76	2	0	1	0	1	1	#NULL!	#NULL!	#NULL!	0	1	#NULL!	0	0	3	1
7383	71	2	0	1	1	1	1	2	0	0	0	0	0	0	0	2	3
7384	69	2	0	1	0	0	1	0	0	1	0	0	0	0	0	2	3

7386	67	1	0	0	0	1	1	0	1	0	0	0	0	1	1	2	4
7387	65	2	0	0	0	1	1	1	0	0	0	0	0	0	0	2	2
7388	87	2	0	0	0	0	2	1	0	0	0	0	0	0	0	2	4
7389	78	1	0	0	0	0	2	1	1	0	0	0	1	0	1	1	4
7390	86	1	1	0	0	0	1	0	0	1	0	0	#NULL!	1	0	3	4
7391	83	2	0	0	0	0	1	1	#NULL!	#NULL!	0	0	#NULL!	0	0	4	1
7392	68	1	0	0	0	0	1	0	0	1	1	1	1	0	0	1	4
7394	66	1	0	0	0	1	1	0	0	1	0	0	0	0	0	2	4
7395	66	2	1	0	0	1	1	2	0	0	0	0	0	0	0	3	2
7397	78	2	0	0	1	0	1	1	0	0	0	0	0	0	0	2	4
7398	77	1	0	1	0	0	1	2	1	1	1	1	0	0	0	3	4
7401	72	1	0	0	0	1	1	1	1	1	1	0	0	0	0	3	4
7403	78	2	0	1	0	0	1	2	0	0	0	0	1	0	0	3	4
7404	71	2	0	0	1	0	1	1	0	0	1	0	0	0	0	3	4
7405	71	1	0	0	1	1	1	1	0	1	1	1	0	0	0	1	4
7406	91	2	1	1	0	0	2	0	0	0	0	0	1	0	0	4	1
7410	71	1	1	0	0	1	2	0	0	1	0	0	1	0	0	4	1
7411	69	2	0	0	0	1	3	1	0	0	0	1	0	0	0	3	3
7415	65	1	1	0	0	1	1	1	1	1	0	0	0	1	1	1	4
7416	80	1	0	0	1	0	1	1	0	1	0	0	0	0	0	1	4
7417	75	2	1	0	1	0	1	1	0	0	0	0	0	0	0	2	3
7419	73	1	0	0	0	0	1	0	0	1	0	0	0	0	0	1	4
7429	73	1	0	0	0	1	1	0	0	0	1	0	0	0	0	2	4
7430	65	2	0	0	0	1	1	1	0	0	1	0	0	0	0	2	4
7432	78	1	0	0	1	1	1	2	1	1	1	1	0	0	0	2	4
7433	71	2	1	0	1	1	1	2	0	0	0	0	1	0	0	3	2
7436	69	1	0	#NULL!	0	#NULL!	3	#NULL!	#NULL!	#NULL!	0	0	#NULL!	0	1	1	4
7439	81	2	0	#NULL!	1	#NULL!	1	2	0	0	0	0	1	0	0	3	4
7442	79	1	0	0	0	1	1	1	1	1	0	0	0	0	1	1	4
7443	77	2	1	0	0	0	1	1	0	0	0	0	1	0	0	2	3
7445	83	2	0	0	0	0	3	2	1	0	1	1	0	0	1	1	4
7447	72	2	0	1	1	#NULL!	1	1	0	0	0	1	0	0	0	3	4
7450	70	2	0	0	1	1	1	#NULL!	#NULL!	#NULL!	0	0	#NULL!	0	0	2	1
7451	72	1	1	1	0	1	3	1	0	1	1	0	0	0	0	1	4
7452	76	1	1	0	1	0	1	1	1	0	0	0	1	0	1	4	1
7454	77	1	0	1	0	#NULL!	1	0	0	0	0	0	0	0	0	2	4
7455	74	2	0	1	0	1	1	1	0	0	1	0	0	0	0	2	4
7456	79	2	0	0	0	1	3	1	0	0	0	0	0	0	0	2	3
7458	66	1	0	0	0	1	3	0	0	1	1	0	0	0	1	3	4

7463	71	1	0	0	1	1	1	1	0	1	0	0	0	0	0	3	4
7464	66	2	0	0	0	1	3	2	0	0	0	0	0	0	0	3	3
7465	77	1	0	0	1	1	2	2	0	1	0	0	0	0	0	3	4
7466	76	2	0	#NULL!	1	1	2	#NULL!	#NULL!	#NULL!	0	1	#NULL!	0	0	4	3
7467	70	1	0	0	0	1	1	2	1	1	0	0	0	0	1	2	4
7471	78	2	0	0	0	0	1	0	0	0	0	0	0	0	0	4	1
7472	70	1	1	0	0	0	1	#NULL!	#NULL!	#NULL!	0	0	#NULL!	0	0	4	4
7475	68	2	0	1	1	1	1	#NULL!	#NULL!	#NULL!	0	0	#NULL!	0	1	2	3
7477	80	1	0	0	0	0	1	2	0	1	0	0	1	0	0	1	4
7478	76	2	0	0	1	0	3	1	0	0	0	0	0	0	0	2	3
7483	73	2	0	0	0	0	1	1	0	0	1	1	0	0	0	2	4
7484	72	1	0	0	0	#NULL!	1	2	1	1	0	0	1	0	0	4	1
7485	73	1	0	0	0	1	1	1	1	1	1	0	0	1	0	1	4
7487	67	1	0	0	0	0	1	#NULL!	1	1	0	1	0	0	1	1	4
7489	75	2	0	1	0	1	1	2	0	0	1	0	1	0	0	2	3
7494	76	1	0	0	0	#NULL!	1	0	0	0	0	0	0	0	0	3	4
7495	72	2	0	0	0	#NULL!	1	0	0	0	0	0	0	0	0	3	3
7498	68	1	0	0	0	0	1	1	1	1	0	1	0	0	1	3	4
7501	72	2	0	1	0	1	1	0	0	0	1	0	0	0	0	2	4
7503	83	2	0	0	0	1	1	1	0	0	0	0	0	0	1	2	4
7505	77	1	0	0	0	1	3	1	1	1	0	1	0	0	0	1	4
7506	76	2	0	0	0	1	1	1	0	0	0	0	0	0	0	4	1
7507	67	1	0	0	1	1	1	0	0	1	0	0	0	0	1	1	4
7508	78	2	0	1	0	0	2	1	0	0	0	0	0	0	0	4	1
7511	70	1	0	0	0	1	1	0	0	0	0	0	0	0	0	1	4
7512	66	2	0	0	0	1	3	1	0	0	0	0	0	0	0	2	1
7513	66	1	0	0	0	0	1	2	0	1	0	0	0	0	0	2	4
7515	73	1	1	0	1	1	3	1	#NULL!	1	0	0	0	0	1	4	4
7516	66	2	0	0	0	1	1	0	#NULL!	#NULL!	1	1	#NULL!	0	1	1	4
7517	76	2	0	1	0	1	3	1	0	0	0	0	0	0	0	3	3
7521	65	1	0	0	0	0	3	0	0	1	1	1	0	0	1	1	4
7522	75	1	0	0	0	0	3	0	0	1	0	1	0	0	0	2	4
7529	67	1	0	0	0	0	1	#NULL!	#NULL!	#NULL!	0	0	#NULL!	0	1	4	4
7530	69	1	0	0	0	0	1	0	0	1	1	0	0	0	0	1	4
7531	67	2	1	0	0	0	1	1	0	0	1	0	0	0	0	2	2
7532	72	1	0	0	0	#NULL!	1	1	0	0	0	0	0	0	0	1	4
7533	68	2	0	0	0	0	1	0	0	0	0	0	0	0	0	4	1
7534	74	1	0	1	0	0	1	#NULL!	#NULL!	#NULL!	1	0	#NULL!	0	0	1	4
7537	84	1	1	0	1	1	1	0	1	0	0	0	1	0	0	3	1

7539	78	1	0	0	1	#NULL!	1	2	0	0	0	0	0	0	0	4	4
7540	78	2	0	0	1	0	2	#NULL!	0	0	0	0	0	0	0	2	4
7542	66	1	0	0	0	1	1	1	1	1	0	0	0	0	0	1	4
7543	65	2	0	0	0	1	1	1	0	0	0	0	0	0	0	3	4
7546	70	2	0	1	0	1	1	1	0	0	0	0	0	0	1	4	4
7548	65	1	0	0	0	0	3	2	0	1	0	1	0	0	1	1	4
7549	68	1	0	0	1	1	1	1	1	1	0	0	1	1	0	3	4
7550	71	2	0	1	1	1	3	1	0	0	0	1	0	0	0	3	4
7555	90	1	1	#NULL!	1	#NULL!	1	0	0	#NULL!	1	1	0	0	0	3	4
7558	75	1	0	0	0	0	#NULL!	1	1	0	0	0	1	0	0	1	4
7563	73	1	0	0	0	1	1	1	0	1	0	0	0	0	0	1	4
7566	79	2	0	0	0	1	1	2	0	0	0	0	1	0	1	2	4
7567	76	1	0	0	0	1	1	2	0	1	0	0	0	0	0	4	3
7570	68	2	0	0	0	1	1	0	0	0	0	0	0	0	0	2	1
7576	69	1	0	0	0	1	1	1	0	1	0	0	0	1	1	1	4
7578	72	2	0	0	0	0	1	2	0	0	0	0	0	0	0	4	1
7580	77	2	0	1	0	1	3	0	0	0	0	0	0	0	0	4	1
7584	86	1	0	0	0	1	1	1	0	1	0	0	1	0	0	1	4
7585	84	2	1	0	0	1	1	2	0	0	0	0	0	0	0	1	4
7588	67	2	0	1	0	1	3	0	0	0	0	1	0	0	0	2	1
7591	72	2	0	0	1	0	3	2	0	0	1	0	0	0	0	2	1
7593	78	2	0	1	0	1	1	0	0	0	0	1	0	0	0	1	4
7595	69	2	0	0	1	1	1	0	0	0	0	0	1	0	1	2	4
7596	68	1	0	0	0	1	3	1	0	1	1	1	1	0	1	1	4
7598	73	2	0	0	0	0	1	1	1	0	1	0	0	0	1	2	4
7600	68	1	0	0	0	1	1	#NULL!	#NULL!	#NULL!	0	1	#NULL!	0	1	1	4
7601	67	2	0	0	0	1	3	0	0	0	0	0	0	0	0	2	2
7603	66	2	0	1	1	1	1	0	0	0	0	0	0	0	0	4	1
7604	65	1	1	0	0	1	1	#NULL!	0	0	1	1	1	0	0	3	4
7605	80	1	1	0	1	1	3	1	0	1	0	0	0	0	0	3	4
7607	74	1	0	0	0	1	3	2	0	1	0	0	0	0	0	2	4
7608	70	2	0	0	0	1	#NULL!	0	0	0	0	0	1	0	0	1	2
7610	66	2	0	0	0	1	1	0	0	0	0	0	0	0	0	1	3
7614	70	1	0	0	0	1	1	2	1	1	0	0	1	0	0	2	4
7616	77	1	0	0	1	1	1	1	0	1	0	0	0	0	0	1	4
7617	65	2	0	0	0	1	3	1	0	1	0	0	0	0	0	2	2
7618	90	1	0	0	0	1	1	0	0	1	0	0	0	1	0	3	4
7620	66	1	0	0	0	1	3	#NULL!	#NULL!	#NULL!	1	0	#NULL!	0	1	2	4
7621	73	1	0	0	0	1	1	1	0	1	0	0	0	0	0	1	4

7622	71	2	0	0	0	1	1	2	0	0	0	0	0	0	0	2	3
7623	85	1	0	0	1	0	1	1	0	1	1	0	0	0	0	1	4
7625	75	1	1	0	0	1	3	1	1	1	0	0	1	1	0	2	4
7626	75	2	0	0	0	1	1	1	1	0	1	0	1	0	0	1	3
7627	83	2	0	0	0	1	2	1	0	0	0	0	0	1	1	1	4
7628	73	2	0	1	0	1	2	1	0	0	1	0	0	0	1	1	4
7629	67	2	0	0	0	0	1	0	0	1	1	1	0	0	1	3	4
7634	74	2	0	0	0	1	3	2	0	0	0	0	1	0	0	1	2
7635	70	1	0	0	0	#NULL!	1	0	1	1	0	0	0	0	0	1	4
7637	70	1	0	0	0	0	1	#NULL!	1	1	0	1	0	0	0	1	4
7638	69	2	0	0	0	0	1	1	0	0	0	0	0	0	0	2	2
7639	78	2	0	0	0	0	3	0	0	0	0	0	0	0	0	2	2
7642	65	2	0	0	0	0	1	0	0	0	1	0	0	0	0	2	2
7643	75	1	1	0	0	#NULL!	1	0	1	1	1	0	1	0	0	1	4
7646	70	1	0	0	0	0	1	2	1	0	1	0	0	0	0	2	4
7647	67	2	0	0	0	0	1	#NULL!	#NULL!	#NULL!	1	0	#NULL!	0	0	2	4
7649	81	2	0	1	0	0	1	0	0	0	1	1	0	0	1	2	3
7654	73	1	0	0	0	1	3	0	0	1	0	0	0	0	0	2	4
7658	82	1	0	1	0	1	1	0	0	1	1	0	0	0	0	1	4
7660	68	2	0	0	0	#NULL!	1	#NULL!	0	0	0	0	0	0	0	1	2
7661	75	1	1	0	0	#NULL!	3	#NULL!	#NULL!	#NULL!	0	0	#NULL!	0	0	2	4
7662	84	2	0	1	0	0	1	2	0	0	0	1	0	0	0	1	4
7665	79	2	0	1	0	0	1	1	0	0	0	0	0	0	0	1	1
7667	69	1	0	0	0	0	1	0	1	1	0	0	1	0	0	2	4
7668	76	1	1	0	0	1	1	1	0	0	0	0	1	0	0	1	4
7669	69	2	0	0	0	1	1	0	0	0	0	0	0	0	0	2	3
7671	73	2	0	1	1	1	1	1	1	0	0	0	0	0	0	3	3
7679	86	2	0	0	0	1	1	0	0	0	0	0	1	0	0	2	4
7681	71	1	0	0	0	0	1	0	0	1	0	0	0	0	1	4	4
7682	67	2	0	0	0	0	1	2	0	0	0	0	0	0	1	2	2
7683	65	1	0	1	0	1	3	1	1	1	0	0	0	0	0	1	4
7684	72	1	0	0	0	0	1	0	0	0	1	1	0	0	0	3	4
7685	68	2	0	0	0	0	1	0	0	0	0	0	0	0	0	2	1
7687	71	2	0	0	0	1	1	1	0	0	0	1	0	0	0	3	3
7690	74	2	0	1	0	0	3	#NULL!	#NULL!	#NULL!	1	1	#NULL!	0	0	1	4
7691	66	1	0	0	0	0	1	2	1	1	0	0	0	0	1	2	4
7692	72	1	1	0	1	1	1	0	1	1	0	0	0	0	0	1	4
7699	85	1	1	0	0	0	#NULL!	0	0	1	0	0	0	1	0	1	4
7703	77	2	0	1	0	1	2	1	0	1	1	0	0	0	0	2	3

7707	71	2	0	0	0	1	1	#NULL!	0	0	0	0	0	0	0	3	2
7710	76	2	0	0	0	0	1	1	0	0	0	0	1	0	0	2	2
7712	80	2	0	0	1	0	1	0	0	0	1	1	0	0	0	2	4
7714	65	1	0	0	1	1	1	0	1	1	1	1	0	0	0	3	4
7716	79	1	0	0	0	0	3	2	1	1	0	0	0	0	0	2	4
7717	74	2	0	0	0	1	1	0	0	0	1	0	0	0	0	2	2
7718	66	1	0	0	0	0	1	1	0	1	1	0	0	0	0	1	4
7719	65	2	0	0	0	1	1	1	0	0	0	0	0	0	0	2	2
7723	78	1	0	0	0	1	1	2	1	1	0	0	0	0	1	2	4
7724	75	2	1	0	0	1	1	1	0	0	0	0	0	0	1	2	4
7725	74	1	0	0	0	1	1	1	0	1	0	0	0	1	0	2	4
7726	72	2	0	0	0	0	1	1	0	0	1	1	0	0	0	2	2
7728	74	1	0	0	0	1	1	1	0	1	0	0	0	0	0	4	1
7729	69	2	0	0	0	1	1	1	0	0	1	1	0	0	0	2	2
7730	71	1	0	0	0	1	1	0	0	0	0	0	0	0	0	1	4
7731	66	2	0	0	0	1	1	0	0	0	1	0	0	0	0	2	3
7733	69	1	1	0	0	1	3	2	0	0	1	1	1	0	0	3	1
7736	77	2	0	0	0	1	3	1	0	0	0	0	0	0	0	2	3
7739	85	2	0	1	1	0	2	1	0	0	0	0	0	0	0	2	4
7741	73	2	0	0	0	1	1	0	0	0	0	0	0	0	1	2	4
7742	66	1	0	0	0	0	1	1	0	1	0	0	0	0	0	1	4
7743	66	2	0	0	0	0	1	1	0	0	1	1	0	0	1	1	2
7744	68	1	0	0	0	0	1	1	1	1	1	1	0	0	0	1	4
7745	80	1	0	0	0	0	1	1	1	1	1	1	0	0	0	3	4
7746	76	2	0	0	1	0	1	0	0	0	0	1	0	0	0	2	4
7747	72	1	0	0	0	0	1	1	1	1	0	0	0	0	1	1	4
7748	67	2	0	0	0	1	2	1	0	0	0	0	0	0	0	3	2
7755	78	2	0	0	0	1	1	0	0	0	0	0	0	0	0	4	1
7756	72	1	0	0	0	1	1	1	0	0	1	0	0	1	1	1	4
7757	71	2	0	0	0	1	1	0	0	0	1	1	0	0	1	2	3
7763	69	1	0	0	0	0	1	2	0	1	1	0	0	0	1	2	4
7764	72	1	0	0	0	1	1	1	0	1	0	0	0	0	0	2	4
7765	77	2	0	1	1	0	1	1	0	0	0	0	0	0	1	4	4
7766	80	2	1	1	1	1	3	1	0	0	0	0	1	0	0	3	2
7767	71	2	0	1	0	1	3	2	0	1	0	0	1	0	0	2	2
7769	68	2	0	0	0	1	3	1	0	0	0	0	0	0	1	2	2
7772	70	2	1	0	#NULL!	0	3	1	0	0	0	1	0	0	0	2	4
7775	86	2	1	1	0	0	1	1	0	0	0	0	0	0	0	4	1
7776	67	1	0	0	0	1	1	1	0	1	1	1	0	0	0	2	4

7777	76	1	0	0	0	1	3	1	0	1	1	1	0	0	0	1	4
7778	73	2	0	0	0	1	3	1	0	0	1	1	0	0	0	2	3
7781	86	2	0	1	0	0	1	0	0	0	0	0	0	0	0	1	2
7783	69	1	0	0	0	0	1	0	0	1	0	0	0	0	1	1	4
7786	76	2	0	0	0	1	1	1	0	0	1	1	0	0	0	2	3
7787	74	2	0	1	0	1	1	0	0	0	1	0	0	0	0	2	2
7790	65	1	1	0	0	1	1	0	0	0	0	0	0	0	0	1	4
7791	76	2	0	1	1	0	1	2	0	0	0	0	1	0	0	1	2
7794	80	2	1	1	0	1	3	1	0	0	1	0	1	0	0	2	3
7796	81	2	0	1	0	0	3	0	0	0	0	1	0	0	0	1	4
7797	74	2	0	1	0	0	1	0	0	0	0	1	0	0	0	1	4
7798	77	2	0	1	0	1	1	0	0	0	0	0	1	0	0	2	1
7804	69	2	0	1	0	1	1	0	0	1	1	0	0	0	1	2	4
7805	66	2	0	0	0	#NULL!	1	0	0	0	1	1	1	0	1	2	2
7806	66	1	1	0	0	1	3	0	1	1	0	0	0	1	1	2	4
7808	69	1	1	0	1	0	1	2	1	1	0	0	0	0	1	3	4
7812	78	2	0	1	0	0	3	1	0	0	0	1	1	0	0	2	4
7813	69	1	0	1	0	1	1	1	0	0	0	0	0	0	1	1	4
7814	70	2	0	1	0	1	1	0	0	0	1	0	0	0	1	2	2
7816	65	1	0	0	0	1	2	1	1	1	0	0	0	0	0	1	4
7819	75	1	0	0	0	0	1	1	1	1	1	1	0	0	0	1	4
7823	90	1	0	1	0	0	1	0	0	0	0	0	0	0	0	1	4
7828	72	1	1	0	0	1	3	2	1	1	0	0	1	1	0	2	4
7829	70	2	0	0	0	1	1	1	0	0	0	0	0	0	0	2	2
7830	67	2	0	0	0	0	1	0	0	1	0	1	0	0	0	4	1
7831	65	1	1	0	0	1	1	0	0	1	0	1	0	0	1	1	4
7834	76	2	0	0	1	1	1	1	0	0	0	0	0	0	0	3	2
7835	81	2	1	1	#NULL!	1	1	#NULL!	0	0	0	0	1	0	1	4	1
7839	66	1	0	0	0	1	1	0	1	1	0	0	0	0	1	3	4
7841	69	2	0	1	0	1	1	#NULL!	1	0	1	0	0	0	0	2	1
7846	79	1	0	0	0	1	3	2	1	0	0	0	0	0	0	4	1
7847	72	2	0	0	0	1	3	0	0	0	1	0	0	0	0	1	4
7848	65	2	0	1	0	0	1	1	0	1	1	0	0	0	1	2	4
7850	69	2	0	0	1	1	3	0	0	0	0	0	1	0	0	2	2
7854	66	2	0	1	1	0	1	#NULL!	0	1	#NULL!	#NULL!	0	0	1	1	4
7858	71	2	0	0	0	0	1	#NULL!	0	0	0	0	0	0	0	2	4
7859	77	1	0	0	0	#NULL!	1	1	0	1	0	0	#NULL!	0	0	1	4
7860	73	2	0	1	1	#NULL!	3	#NULL!	0	0	0	0	0	0	0	2	4
7862	84	2	1	0	1	1	3	1	0	0	0	0	#NULL!	0	1	3	1

7867	67	1	1	0	0	1	1	1	0	1	1	0	#NULL!	1	1	2	4
7869	84	1	1	0	1	1	1	1	0	1	0	0	0	1	0	1	4
7870	75	2	0	0	1	0	1	1	0	0	0	0	0	0	0	2	3
7871	76	1	0	0	1	0	1	1	0	1	0	0	0	0	1	1	4
7872	71	2	0	0	1	0	1	2	0	0	0	0	0	0	1	4	1
7873	72	1	0	0	0	1	1	0	0	1	1	0	0	0	1	4	4
7874	67	2	0	0	0	1	1	0	0	0	0	0	0	0	1	1	4
7875	74	1	0	0	1	1	1	2	0	1	0	0	0	0	0	2	4
7876	67	2	0	0	0	1	3	0	0	0	0	0	0	0	1	3	4
7879	67	1	0	0	0	1	1	1	0	0	1	0	0	0	0	1	4
7880	77	1	0	0	1	0	1	0	1	1	0	0	0	0	0	3	4
7881	74	2	0	0	0	1	1	1	0	0	1	1	0	0	0	2	2
7882	85	1	0	0	0	0	1	1	1	1	0	0	1	0	0	1	4
7883	80	2	1	0	1	0	2	1	0	0	0	0	0	0	0	2	1
7884	67	1	0	0	0	1	1	1	1	1	0	0	0	0	0	2	4
7885	73	1	0	0	1	1	1	0	0	1	0	0	0	0	0	1	4
7886	72	2	0	0	0	1	1	0	0	0	1	0	0	0	0	2	3
7888	85	2	1	1	0	1	1	1	0	1	1	0	0	0	0	1	2
7890	69	2	0	0	0	0	1	2	1	1	0	0	1	0	0	2	2
7891	65	1	0	1	0	1	1	0	1	1	0	0	0	0	0	2	4
7893	73	1	0	0	1	1	1	0	0	1	0	0	1	0	0	3	4
7896	72	2	0	0	0	0	1	0	0	0	1	0	1	0	0	2	3
7898	66	1	0	0	0	0	1	1	1	1	1	1	0	0	0	1	4
7905	73	1	0	0	1	1	1	1	0	1	1	0	0	1	0	3	4
7906	71	2	0	0	1	1	1	0	0	0	0	0	0	0	0	2	4
7907	67	2	0	#NULL!	0	1	1	1	0	0	0	0	1	0	0	2	4
7910	71	2	1	1	1	0	1	0	0	0	0	0	0	0	0	2	1
7911	71	2	0	0	0	1	3	1	0	1	0	0	1	0	0	2	3
7912	66	1	0	0	0	0	3	0	1	0	0	0	0	0	1	2	4
7913	68	1	0	1	0	0	3	1	1	0	1	0	0	0	0	1	4
7915	70	1	0	0	1	0	3	2	1	1	1	0	0	0	0	3	4
7916	67	2	0	0	1	0	1	0	1	0	0	0	1	0	0	2	4
7919	76	2	0	0	0	0	2	0	0	0	0	1	0	0	0	2	3
7920	67	1	0	0	0	0	2	0	0	1	0	0	0	1	1	2	4
7921	76	2	0	1	0	0	3	1	1	0	0	0	0	0	0	2	3
7923	70	2	0	0	0	1	1	#NULL!	0	0	1	0	1	0	0	2	1
7924	75	1	0	0	0	0	1	1	0	1	1	0	0	0	0	2	4
7925	69	2	0	0	0	0	1	1	0	0	1	0	0	0	0	2	2
7926	65	2	0	0	0	0	1	0	0	0	1	0	0	0	0	2	4

7928	73	2	0	#NULL!	0	#NULL!	3	#NULL!	#NULL!	#NULL!	1	0	#NULL!	0	0	2	3
7929	68	1	0	0	0	1	1	0	0	1	0	1	0	0	1	1	4
7930	70	1	0	0	1	1	3	2	0	1	0	0	0	0	0	3	4
7931	65	2	0	0	1	1	2	0	0	0	0	0	0	0	1	3	4
7934	67	1	0	0	0	0	1	0	0	1	0	0	0	0	0	3	4
7936	67	2	0	0	0	0	1	0	0	0	0	0	0	0	1	2	2
7937	77	2	1	1	0	1	2	1	0	0	0	0	1	0	0	2	3
7939	69	2	0	0	0	#NULL!	1	1	0	0	0	0	0	0	0	4	1
7940	73	2	0	1	1	1	1	0	1	1	0	0	0	0	0	2	4
7941	86	2	0	1	1	1	1	0	0	0	0	0	1	1	0	2	2
7943	72	2	0	1	1	1	1	#NULL!	0	0	1	0	1	0	0	2	2
7944	69	2	0	0	1	1	1	0	1	1	0	0	0	0	0	2	2
7947	77	1	1	0	0	1	1	1	0	0	0	1	0	1	0	2	4
7949	70	1	0	0	0	1	1	0	1	1	0	0	0	0	1	3	4
7957	73	2	0	0	1	1	1	0	0	0	0	0	1	0	0	4	1
7958	72	1	1	0	0	1	1	0	0	1	0	0	1	0	0	1	4
7963	72	2	1	1	1	#NULL!	1	1	0	1	1	0	0	0	0	4	1
7969	70	1	0	0	0	1	1	1	0	0	0	0	0	0	0	1	4
7970	86	2	1	1	1	0	1	0	0	0	1	0	0	0	0	2	2
7974	70	1	1	0	0	1	3	#NULL!	#NULL!	#NULL!	0	0	#NULL!	0	0	1	4
7975	78	2	0	1	1	1	1	2	0	0	0	1	1	0	0	3	4
7976	72	1	1	0	1	0	1	1	0	1	0	1	0	0	0	2	3
7977	67	2	0	0	0	1	#NULL!	1	0	0	0	0	0	0	0	2	2
7980	72	2	0	1	1	1	1	#NULL!	#NULL!	#NULL!	0	0	#NULL!	0	0	3	3
7981	70	2	0	1	0	1	3	1	0	0	0	0	0	0	0	2	3
7982	66	2	0	1	0	1	3	0	0	0	0	0	0	0	1	2	4
7983	65	1	0	1	0	1	1	2	0	0	1	0	0	0	1	2	4
7984	68	1	0	0	0	1	1	2	1	1	0	0	0	0	0	2	4
7985	70	1	0	0	0	1	1	0	0	1	0	0	0	0	0	1	4
7993	72	2	0	1	0	1	1	1	0	1	0	0	0	0	0	1	4
7994	76	1	0	0	0	1	2	#NULL!	0	0	0	0	#NULL!	0	0	3	4
7996	65	1	0	0	0	0	3	2	0	1	0	0	0	0	1	1	4
7998	79	1	0	0	0	1	1	0	1	1	0	0	0	0	0	2	4
8001	84	2	1	1	1	0	2	0	0	0	0	0	1	0	0	3	4
8003	75	2	1	1	0	1	1	2	0	0	1	0	0	0	0	3	4
8009	74	1	1	1	1	1	1	0	1	0	0	0	1	0	0	1	4
8014	78	1	0	0	0	0	2	0	0	1	0	0	0	0	1	1	4
8020	73	1	0	0	1	0	1	#NULL!	#NULL!	#NULL!	0	1	#NULL!	0	1	1	4
8029	78	1	1	0	1	0	1	0	1	0	1	1	0	0	1	1	4

8030	74	2	0	0	1	0	1	0	0	0	0	0	1	0	0	3	1
8031	65	1	0	0	1	1	3	1	0	1	0	0	0	0	1	3	4
8032	79	1	0	0	0	1	3	1	1	0	0	0	0	0	0	4	1
8033	74	2	0	0	0	1	3	1	0	0	0	0	0	0	0	4	2
8042	74	2	0	1	1	0	2	0	1	0	1	0	0	0	0	2	4
8043	90	2	0	1	0	1	1	1	0	0	0	0	1	0	0	4	1
8044	65	1	0	0	0	1	1	0	1	0	1	1	0	0	0	1	4
8046	72	2	0	1	0	0	1	0	0	0	1	1	0	0	0	2	3
8047	67	2	0	0	0	0	3	1	0	0	1	1	0	0	0	2	4
8048	81	1	0	1	1	0	3	1	0	0	0	0	1	0	0	1	4
8049	77	1	0	0	0	0	1	0	0	1	1	1	0	0	0	1	4
8050	71	2	0	0	0	0	1	1	0	0	1	0	0	0	0	2	4
8051	66	1	1	0	0	1	3	1	1	1	1	0	1	0	1	1	4
8053	70	1	0	0	0	1	1	0	0	0	0	0	0	0	0	2	4
8057	66	2	0	1	0	1	1	0	0	0	0	0	0	0	0	3	4
8058	79	2	0	1	0	0	1	1	0	0	0	0	0	0	0	2	3
8061	66	2	0	0	1	1	1	0	1	0	0	0	0	0	0	3	2
8064	75	1	0	0	0	1	1	2	0	1	0	0	1	0	0	2	4
8072	75	2	1	0	1	1	1	0	0	0	1	1	0	0	0	4	1
8073	66	2	0	0	1	0	1	0	1	0	0	0	0	0	0	2	2
8074	72	2	0	1	0	0	1	1	0	0	0	0	0	0	0	2	1
8075	73	1	0	0	0	0	1	1	0	0	0	0	0	0	0	2	4
8076	72	2	1	0	0	0	1	1	0	0	0	0	0	0	0	2	2
8078	72	2	0	0	1	0	1	1	1	1	1	0	0	0	0	3	4
8079	71	1	1	0	1	1	#NULL!	1	0	1	0	0	0	0	0	3	1
8080	67	2	0	1	0	1	3	1	0	0	0	0	1	1	0	3	4
8081	78	1	0	0	0	1	1	1	0	1	1	0	0	0	0	1	4
8083	70	1	0	0	0	0	1	0	0	1	0	0	0	0	1	2	4
8087	66	2	0	1	0	0	1	1	0	1	0	0	1	0	0	1	2
8089	76	2	0	0	1	0	1	#NULL!	0	0	0	0	1	0	1	2	4
8091	77	1	0	0	0	1	1	1	1	1	0	1	0	0	0	2	4
8092	65	2	0	0	0	1	1	0	0	0	1	0	0	0	0	2	3
8094	68	2	0	0	0	0	1	0	1	1	1	0	1	0	1	2	4
8095	84	2	0	1	0	0	1	#NULL!	#NULL!	#NULL!	1	1	#NULL!	0	0	1	2
8097	79	2	0	0	0	0	1	1	0	0	0	1	1	0	0	2	1
8098	74	1	1	0	0	0	1	1	1	0	1	0	0	0	1	1	4
8099	70	2	0	0	0	0	2	0	0	0	1	0	1	0	0	2	1
8101	70	1	0	0	0	0	3	0	1	1	0	0	0	0	0	2	2
8103	74	1	0	0	0	1	1	1	0	0	0	0	1	0	1	2	4

8104	71	2	0	0	0	1	1	0	0	0	0	1	0	0	0	2	2
8105	83	2	0	1	0	0	1	#NULL!	0	0	1	0	0	0	0	1	4
8107	80	2	0	1	0	0	1	0	0	0	1	0	0	0	0	2	2
8111	77	2	1	0	0	0	1	1	0	0	0	0	1	0	0	4	1
8112	70	1	0	0	0	1	3	2	0	1	0	0	0	1	1	2	4
8114	66	2	0	0	0	0	1	0	0	0	1	1	1	0	0	4	1
8115	77	1	0	0	0	0	3	0	1	0	1	0	1	0	1	1	4
8116	74	2	0	0	0	0	1	0	0	0	0	0	1	0	0	2	2
8118	68	1	0	0	0	#NULL!	1	1	1	0	0	0	0	0	1	3	4
8119	81	1	0	0	0	1	1	2	0	0	1	1	1	0	0	4	4
8120	72	2	0	0	0	1	3	1	0	0	0	0	1	0	0	2	1
8122	67	2	0	0	0	#NULL!	1	0	0	0	1	0	1	0	0	2	2
8124	66	2	0	0	0	0	3	1	0	0	1	1	0	0	1	1	4
8126	65	1	0	0	0	#NULL!	1	0	1	1	1	0	0	0	0	2	4
8136	75	2	0	1	0	0	1	2	0	0	0	0	0	0	0	4	2
8137	83	2	0	1	0	0	1	1	0	0	0	0	0	0	0	2	1
8138	87	2	0	1	0	1	1	1	0	0	1	1	0	0	0	4	1
8144	85	1	1	0	0	0	1	#NULL!	#NULL!	#NULL!	0	0	#NULL!	0	0	1	4
8147	70	2	1	0	0	1	1	1	0	1	0	0	1	0	1	1	4
8149	79	2	0	0	0	0	3	2	0	1	0	0	1	0	0	2	3
8150	70	1	0	0	0	0	3	1	1	1	1	0	0	0	0	1	4
8151	76	2	0	1	0	0	1	0	0	0	0	0	0	0	0	4	1
8154	68	1	0	0	0	1	3	1	0	1	1	1	0	0	0	1	4
8156	72	2	0	0	0	0	1	1	0	0	0	0	0	0	1	4	1
8157	88	1	0	1	0	0	3	1	0	1	0	0	1	0	0	1	4
8158	79	2	0	0	0	0	1	0	0	0	0	0	0	0	1	2	2
8159	89	1	0	0	0	1	1	1	1	1	1	1	0	0	1	1	4
8161	84	2	0	1	0	0	2	1	0	0	0	0	0	0	0	2	1
8163	68	1	0	0	0	0	1	#NULL!	#NULL!	#NULL!	0	0	#NULL!	1	0	4	4
8167	67	2	0	0	1	0	3	1	0	1	1	0	0	0	1	2	4
8169	79	2	0	0	1	0	3	1	0	0	0	0	0	0	0	4	1
8174	67	2	0	0	0	0	1	1	0	0	1	0	0	0	1	2	4
8176	89	1	0	1	1	0	3	2	1	0	0	0	0	0	0	3	4
8177	77	1	0	0	0	0	1	1	0	0	1	0	0	1	0	1	4
8178	74	2	0	0	0	0	1	0	0	0	0	1	0	0	0	1	4
8180	67	1	0	0	0	0	1	1	1	1	1	0	0	0	1	1	4
8182	72	1	0	0	0	0	1	1	1	1	1	0	0	0	0	1	4
8185	84	2	0	1	0	0	1	0	0	1	0	0	1	0	0	4	1
8187	65	2	0	0	0	0	1	0	0	0	1	0	0	0	0	2	1

8189	69	1	0	0	0	1	1	2	0	0	1	0	1	0	1	2	4
8191	82	1	0	1	0	0	1	1	1	1	1	0	0	0	0	1	4
8193	71	2	0	1	0	0	1	1	0	0	0	0	0	0	0	2	2
8197	73	2	0	1	0	0	1	2	0	0	0	0	0	0	0	2	2
8199	83	2	0	1	1	0	1	#NULL!	#NULL!	#NULL!	0	1	#NULL!	0	0	2	2
8200	70	1	0	0	0	0	3	#NULL!	#NULL!	#NULL!	0	0	#NULL!	0	1	2	4
8204	77	1	0	0	1	0	1	1	1	1	0	0	0	0	1	1	4
8205	71	2	0	0	0	0	1	1	0	0	0	0	0	0	0	2	4
8207	77	2	1	0	0	0	1	1	0	0	1	1	1	0	0	2	1
8210	66	2	0	1	0	1	3	2	0	1	1	0	0	0	0	2	2
8213	79	2	0	1	0	0	2	0	0	0	0	0	0	0	0	2	1
8215	90	1	1	0	0	0	1	0	1	1	1	0	0	0	1	1	4
8216	80	1	1	0	0	#NULL!	1	0	0	1	0	0	0	0	0	1	4
8217	77	2	0	0	0	1	1	0	0	0	1	1	0	0	0	2	1
8219	66	1	0	0	0	0	1	#NULL!	0	1	0	0	0	0	1	1	4
8221	65	2	0	0	0	1	1	0	0	0	0	1	0	0	0	4	3
8223	67	2	0	0	0	0	3	1	0	0	0	1	0	0	1	2	4
8227	75	1	0	0	0	0	3	0	0	1	1	1	0	0	0	2	4
8229	83	1	1	0	0	0	1	2	1	1	0	1	0	0	0	1	4
8230	79	2	0	0	0	0	1	0	0	0	1	1	0	0	0	2	2
8231	72	1	0	0	0	0	1	0	0	1	0	1	1	0	0	1	4
8232	68	1	0	0	0	0	1	2	0	1	0	0	1	0	1	2	4
8233	69	1	0	0	0	0	1	0	0	1	1	1	0	0	1	1	4
8234	65	2	0	0	0	0	2	0	0	0	0	1	0	0	1	2	2
8239	88	1	1	0	0	1	1	1	1	0	1	1	0	0	0	2	4
8244	80	2	1	0	0	0	2	0	0	0	1	1	0	0	0	4	1
8245	72	1	0	1	0	0	1	2	0	1	1	1	0	0	1	1	4
8248	69	1	0	0	#NULL!	1	1	1	1	1	0	0	0	0	1	1	4
8249	67	1	0	0	0	0	1	0	0	1	0	0	0	0	0	4	4
8253	74	1	0	0	0	1	3	1	1	1	0	0	0	0	0	1	4
8254	69	2	0	0	0	1	1	0	0	0	0	0	0	0	1	2	4
8256	88	2	1	1	0	#NULL!	2	0	0	0	0	1	1	0	0	4	2
8257	78	2	1	1	0	0	1	0	0	#NULL!	1	0	0	0	0	4	1
8260	74	1	1	0	0	1	1	0	0	1	0	1	0	0	0	1	4
8261	71	2	0	0	0	1	1	0	0	0	0	0	0	0	0	4	1
8269	84	1	0	0	0	0	1	0	1	1	0	1	0	0	1	1	4
8270	70	2	0	1	0	0	1	0	0	0	1	0	0	0	0	2	4
8272	77	2	1	1	0	1	1	0	0	0	1	0	1	0	0	2	2
8275	66	1	0	1	0	1	1	1	0	1	0	0	0	0	1	2	4

8279	65	2	1	0	0	1	3	1	0	0	0	1	1	0	0	4	1
8280	75	1	0	0	0	1	3	1	1	1	1	1	0	0	1	4	4
8281	71	2	0	0	0	1	1	1	1	1	1	1	0	0	0	4	1
8283	66	1	0	0	0	0	1	#NULL!	1	1	1	1	0	0	0	3	4
8284	65	2	0	0	0	0	1	0	0	0	1	0	0	0	0	1	3
8286	68	1	0	0	0	0	1	2	0	1	0	0	0	1	0	3	4
8287	69	2	1	1	0	0	3	0	0	0	1	1	0	0	0	2	2
8291	80	2	0	0	1	1	1	1	0	0	1	0	#NULL!	0	1	2	3
8292	81	1	0	0	0	0	1	2	1	1	1	0	0	0	1	1	4
8293	77	2	0	0	0	0	2	0	1	0	1	0	0	0	1	2	4
8294	82	1	0	0	1	#NULL!	1	#NULL!	0	1	0	0	0	0	1	3	4
8296	71	2	0	0	0	0	1	0	1	0	0	0	0	0	0	1	4
8298	67	2	0	0	0	0	1	1	0	1	1	0	0	0	1	2	4
8300	66	2	0	1	0	1	1	#NULL!	0	0	1	0	0	0	0	2	2
8302	82	2	0	1	0	0	1	1	0	0	0	0	0	0	0	4	3
8304	75	2	0	1	0	0	1	1	0	1	0	0	0	0	0	2	3
8306	66	2	0	0	0	1	1	#NULL!	1	0	0	0	0	0	1	1	4
8307	71	2	1	1	0	0	3	0	0	0	1	0	0	0	1	2	2
8310	66	1	0	1	0	1	1	0	1	1	1	0	1	0	1	1	4
8315	68	2	0	0	1	1	1	1	0	0	0	0	0	0	0	3	4
8316	67	1	0	0	0	1	2	1	1	1	0	0	0	0	0	3	4
8317	66	1	0	0	0	#NULL!	1	0	1	1	0	0	0	0	0	1	4
8320	69	2	0	1	0	1	3	0	0	0	0	0	0	0	0	2	2
8321	82	2	0	1	0	1	1	2	0	0	0	0	1	1	0	4	1
8322	76	2	0	1	0	1	1	2	0	0	0	1	0	0	0	2	4
8326	70	2	1	1	0	0	3	0	0	0	0	0	0	0	0	4	1
8329	69	2	0	1	0	0	1	0	0	0	0	0	0	0	1	1	4
8331	66	1	0	0	0	0	3	#NULL!	#NULL!	#NULL!	0	0	#NULL!	0	1	1	4
8333	71	1	0	0	0	0	1	0	1	1	0	0	1	0	0	1	4
8336	80	2	0	0	0	0	1	1	0	0	1	0	#NULL!	0	1	1	4
8337	77	1	0	0	0	0	1	1	0	1	0	1	0	0	0	4	1
8338	77	1	0	0	0	0	1	1	1	1	1	0	0	0	0	1	4
8339	76	2	0	0	0	1	#NULL!	1	0	0	1	1	0	0	0	1	4
8342	73	2	1	1	0	1	1	0	0	0	0	1	1	0	0	4	1
8346	75	1	0	0	0	#NULL!	1	0	0	0	0	0	0	0	0	2	4
8348	76	1	0	0	0	0	1	1	1	1	0	0	0	0	0	1	4
8349	71	2	0	0	0	1	1	1	0	0	0	1	0	0	0	2	1
8351	80	2	0	0	0	#NULL!	1	0	0	0	0	0	1	0	0	2	1
8353	77	2	0	#NULL!	0	#NULL!	1	2	0	0	0	0	1	0	1	1	4

8364	78	2	0	0	0	0	1	0	1	0	0	0	0	0	1	2	4
8365	78	1	1	0	0	0	1	0	1	0	1	0	0	0	1	2	4
8368	74	2	1	0	0	1	1	0	0	0	0	0	1	0	0	1	4
8369	86	2	0	1	0	0	1	0	0	0	0	0	0	0	0	1	4
8370	81	2	1	1	1	#NULL!	#NULL!	1	0	0	1	1	0	0	0	2	1
8371	82	1	0	1	0	0	1	1	1	1	1	1	0	0	0	1	4
8375	74	1	0	0	0	1	1	0	0	0	1	1	0	0	0	2	4
8378	78	1	0	1	0	0	1	0	0	1	0	1	0	0	0	1	4
8379	82	1	1	1	1	1	1	1	0	0	1	0	0	0	0	1	4
8383	65	1	0	0	0	#NULL!	1	0	0	1	0	0	0	1	0	1	4
8384	68	2	0	1	0	1	1	0	0	0	0	1	0	0	0	4	1
8388	85	2	0	1	0	1	1	1	0	0	0	0	1	0	0	2	2
8392	70	1	0	0	0	0	1	0	1	1	0	0	0	0	1	2	4
8393	67	2	1	0	0	1	2	1	0	0	0	1	0	0	0	4	1
8394	73	2	0	#NULL!	0	#NULL!	1	1	0	0	1	0	0	0	0	2	4
8397	77	1	0	0	0	0	1	0	0	0	0	0	0	0	1	1	4
8398	73	2	0	0	0	0	1	1	0	0	1	1	0	0	0	4	1
8399	69	2	0	0	0	0	1	0	0	0	0	0	0	0	0	2	3
8401	68	1	0	0	1	0	1	1	1	1	1	0	0	0	0	3	4
8402	87	1	0	0	0	0	#NULL!	0	0	1	0	0	0	0	1	4	1
8403	86	2	0	0	0	0	#NULL!	0	0	0	1	1	0	0	0	3	1
8406	75	2	1	1	0	1	3	2	0	0	0	0	1	0	0	2	2
8407	71	1	0	0	0	1	1	1	0	0	0	0	0	0	1	1	4
8408	66	2	0	0	0	1	1	0	1	0	1	1	0	0	0	1	2
8411	66	1	0	0	0	0	1	0	1	1	0	0	0	0	0	1	4
8415	73	1	0	0	0	1	3	#NULL!	#NULL!	#NULL!	1	0	#NULL!	1	1	4	4
8416	67	2	0	0	0	1	1	1	0	0	1	0	0	0	1	2	4
8417	73	1	0	0	0	0	3	1	1	0	1	0	0	0	0	1	4
8421	80	2	0	0	0	#NULL!	1	0	0	0	1	0	0	0	1	2	3
8424	73	1	0	#NULL!	0	#NULL!	3	1	1	1	0	0	0	0	1	2	1
8433	83	1	0	0	0	0	1	1	0	1	0	1	0	0	0	2	4
8434	80	2	0	0	0	0	2	0	0	0	0	0	#NULL!	0	0	2	4
8436	81	2	0	1	0	1	1	0	0	0	0	0	0	0	0	2	3
8440	68	2	0	1	0	1	1	0	0	0	1	0	0	0	0	2	4
8441	75	1	0	0	0	1	1	0	0	1	0	0	1	0	0	2	4
8442	68	2	0	0	0	1	1	1	0	0	0	0	0	0	0	2	4
8443	73	1	0	0	0	0	1	2	1	0	0	0	0	1	1	1	4
8444	69	2	0	0	0	0	1	0	0	0	1	0	0	0	0	2	2
8445	80	1	1	0	0	0	1	1	1	1	0	0	0	0	0	1	4

8450	71	1	0	0	0	#NULL!	1	0	1	1	1	1	0	0	1	4	1
8451	69	2	0	0	0	0	2	0	0	0	1	0	0	0	0	2	1
8453	77	2	0	0	0	1	1	1	0	0	1	1	0	0	0	2	1
8455	71	2	0	0	0	0	1	1	0	0	0	0	0	0	0	4	1
8457	76	2	0	0	0	0	1	1	0	0	0	1	0	0	0	2	2
8458	67	2	0	1	0	0	3	1	0	0	1	0	0	0	1	2	4
8459	66	1	0	0	0	0	3	0	1	0	1	0	0	0	0	1	3
8460	65	2	0	0	0	0	1	0	1	0	1	0	0	0	0	4	1
8461	65	1	0	0	0	1	1	0	0	0	0	0	0	0	1	2	4
8462	75	1	0	0	0	0	3	0	1	1	1	1	0	0	0	1	4
8463	71	2	0	0	0	0	1	0	0	0	0	1	0	0	0	4	1
8464	77	1	0	0	0	0	1	2	1	0	0	1	1	0	0	1	4
8465	73	2	1	0	0	0	2	1	0	0	1	1	0	0	0	4	1
8466	76	1	0	0	0	0	1	0	0	0	0	1	0	0	0	2	4
8468	75	2	0	1	0	0	1	0	0	0	0	0	0	0	0	2	2
8469	79	1	0	0	0	0	1	0	1	0	0	1	0	0	1	1	4
8470	79	2	0	0	0	0	1	1	0	0	1	1	0	0	0	2	1
8471	68	1	0	0	0	0	1	1	0	1	0	0	0	0	0	1	4
8473	66	2	0	0	0	0	1	#NULL!	1	0	1	0	0	0	0	2	1
8474	65	1	0	0	0	0	1	0	0	1	1	0	0	0	1	1	4
8475	81	1	0	0	0	1	1	2	1	1	0	0	0	0	0	1	4
8476	70	2	0	1	0	0	1	0	0	0	1	1	0	0	0	4	1
8477	76	1	0	0	0	0	1	1	0	0	0	0	0	0	0	1	4
8478	72	2	0	0	0	0	1	0	0	0	0	0	0	0	0	4	1
8479	84	2	0	1	0	1	3	1	0	0	0	0	0	0	0	4	1
8480	66	2	0	1	0	0	1	1	0	0	0	1	0	0	0	1	4
8481	84	2	0	1	0	1	1	1	0	0	0	0	0	0	0	4	2
8486	84	1	0	0	0	#NULL!	1	1	0	1	0	1	1	0	0	2	4
8489	69	1	0	0	0	1	1	0	0	1	1	0	0	1	0	2	4
8490	69	2	0	0	0	0	1	0	0	0	0	0	0	0	1	1	4
8493	76	1	1	0	0	0	1	0	0	0	0	0	0	0	1	2	4
8494	69	2	0	1	0	0	1	1	0	0	0	0	0	0	0	2	1
8496	66	1	0	0	0	0	1	1	0	1	0	0	0	0	0	1	4
8497	71	1	0	1	0	0	3	0	0	1	1	0	0	0	1	1	4
8498	66	1	0	0	0	0	1	0	0	0	0	0	0	0	1	1	4
8499	74	2	0	0	0	0	1	1	0	0	0	0	0	0	0	1	4
8501	66	1	1	0	0	0	2	0	0	0	0	1	0	0	0	1	4
8504	75	1	0	0	1	1	1	1	1	1	0	0	1	0	0	1	4
8505	72	2	0	0	1	0	1	1	0	0	0	0	1	0	1	1	4

8507	68	1	0	0	0	0	1	1	1	1	1	0	0	0	1	1	4
8508	65	2	0	0	0	0	1	0	0	0	1	0	0	0	0	1	4
8509	80	1	0	0	0	0	1	0	0	0	0	0	1	0	0	1	4
8510	74	2	0	0	0	0	1	0	0	0	0	1	0	0	0	4	1
8511	81	2	1	1	0	1	1	1	0	0	0	0	0	0	0	4	1
8512	69	2	0	1	0	1	3	1	0	0	1	1	0	0	0	2	4
8513	76	2	0	0	0	0	1	1	0	0	1	0	0	0	0	2	2
8515	65	1	0	0	0	0	1	1	1	1	1	1	0	0	1	1	4
8516	87	1	0	0	1	0	3	0	0	0	0	0	0	0	1	4	4
8517	79	2	0	0	0	0	1	0	0	0	0	0	0	0	1	2	4
8518	82	2	0	0	0	0	1	1	0	0	0	0	0	0	0	2	1
8519	81	1	0	0	0	0	1	0	0	1	0	0	0	0	0	3	4
8520	69	1	0	0	0	1	1	1	0	1	0	0	1	0	0	1	4
8521	70	1	0	0	0	0	1	2	1	1	0	0	0	0	0	1	4
8524	65	2	0	1	0	0	1	0	0	0	1	0	0	0	0	1	4
8525	68	1	0	0	0	1	1	2	1	0	1	1	0	0	0	1	4
8528	85	1	0	0	0	1	1	0	0	0	0	0	0	0	0	3	4
8529	83	2	0	0	0	1	3	#NULL!	0	0	0	0	1	0	0	1	4
8532	69	2	0	0	0	0	1	1	0	1	0	0	1	0	0	2	1
8534	81	2	0	0	0	1	1	1	0	0	0	0	1	0	0	2	4
8536	84	2	1	1	1	0	1	0	0	0	1	0	0	0	0	1	4
8537	77	1	1	0	0	0	3	2	1	0	0	0	0	0	0	3	4
8538	74	2	0	0	0	0	1	0	0	0	0	0	0	0	0	2	2
8539	70	2	0	1	0	0	1	0	0	0	1	0	0	0	0	2	4
8543	80	1	0	1	0	0	3	1	1	0	0	0	0	0	1	1	4
8544	74	1	0	1	0	1	1	1	0	1	1	0	0	0	0	2	4
8545	72	1	0	0	0	1	1	2	0	1	0	0	1	1	0	1	4
8547	75	2	0	0	0	1	1	1	0	0	0	0	1	0	0	2	4
8548	74	1	1	0	0	1	3	2	1	0	0	0	0	0	0	2	4
8549	66	2	0	0	0	1	3	0	0	0	1	1	0	0	0	4	1
8554	68	1	0	0	0	#NULL!	1	1	1	1	1	0	0	0	0	1	4
8557	71	1	0	1	0	0	3	2	1	0	1	0	0	0	1	1	4
8564	84	2	0	1	0	1	1	0	0	0	1	0	0	0	0	2	3
8566	82	2	1	1	0	1	1	0	0	0	0	0	0	1	0	2	1
8567	82	1	1	0	0	0	3	2	1	1	0	0	0	0	1	1	4
8568	73	2	0	0	1	0	1	0	0	0	0	0	0	0	1	2	4
8569	73	2	0	1	1	0	1	1	0	0	0	0	0	0	0	4	4
8571	70	2	1	0	0	0	1	0	0	0	1	0	0	0	0	4	1
8572	68	1	0	0	0	1	1	2	0	1	0	0	1	0	0	3	4

8574	67	2	0	0	0	0	1	0	1	0	1	0	0	0	0	1	2
8576	73	2	0	0	0	0	1	0	0	0	1	0	0	0	0	2	1
8581	74	1	0	0	0	0	1	2	0	1	1	0	0	0	0	1	4
8582	69	2	0	0	0	0	1	0	0	0	1	1	0	0	0	2	2
8583	79	1	0	0	1	1	1	2	1	0	0	0	1	0	0	1	4
8584	73	2	0	0	0	1	1	0	0	0	1	1	0	0	0	2	1
8585	73	1	0	0	0	0	1	1	1	1	1	0	0	0	1	1	4
8586	71	2	0	0	0	#NULL!	1	0	0	0	0	0	0	0	0	4	1
8587	70	2	0	1	0	0	1	1	0	0	1	1	0	0	0	2	1
8589	72	2	0	0	0	0	1	#NULL!	#NULL!	#NULL!	1	0	#NULL!	0	0	4	1
8592	81	1	0	0	0	0	1	1	1	1	0	1	0	0	0	1	4
8593	76	2	0	0	0	0	1	#NULL!	0	0	1	1	0	0	0	2	1
8594	90	1	1	0	0	0	1	0	0	0	0	0	0	0	1	1	4
8595	88	2	0	0	0	0	1	1	0	0	1	0	0	0	0	2	2
8596	74	2	0	1	0	0	1	1	1	0	1	1	0	0	0	2	1
8597	66	2	0	1	0	0	1	0	0	0	1	0	0	0	0	1	4
8598	83	1	1	0	0	1	2	1	0	1	0	0	0	0	0	2	4
8599	78	2	0	0	0	1	1	0	0	0	0	1	1	0	0	4	1
8600	74	2	0	1	1	1	#NULL!	0	0	0	0	0	1	0	0	4	1
8603	67	2	0	1	0	1	2	0	0	0	0	0	0	0	0	2	4
8604	70	1	0	0	0	0	1	1	1	1	0	0	0	0	0	1	4
8605	70	2	0	0	0	0	2	0	0	0	0	1	0	0	0	2	2
8606	74	2	0	0	0	0	2	0	0	0	1	1	0	0	0	1	3
8608	69	2	0	0	0	0	1	0	0	0	1	1	0	0	0	2	2
8610	88	1	0	0	0	0	3	1	0	0	1	0	0	0	0	2	4
8611	84	2	0	0	0	1	1	1	0	0	1	0	0	0	0	2	2
8616	83	2	0	0	0	1	2	1	0	0	0	0	0	0	0	2	3
8617	70	2	0	1	0	1	1	0	0	0	0	0	0	0	0	2	2
8618	70	2	1	0	0	1	1	1	0	0	1	1	0	0	0	2	3
8620	78	1	0	0	0	0	1	0	1	0	1	1	0	0	0	2	4
8621	73	2	0	0	1	0	1	0	0	0	1	0	0	0	0	4	1
8622	74	1	0	0	0	0	1	0	0	0	1	1	1	0	0	2	4
8623	71	2	0	0	0	0	1	0	0	0	1	0	0	0	0	2	1
8625	85	1	0	0	0	#NULL!	1	0	0	0	0	0	0	0	0	1	4
8627	78	2	0	1	0	0	1	2	0	0	0	0	0	0	1	2	1
8629	74	1	1	0	0	0	1	1	0	1	0	0	0	0	0	2	4
8635	72	2	1	0	0	1	1	1	0	0	0	0	0	0	1	3	4
8638	74	2	0	0	1	1	1	0	0	0	0	0	0	0	0	3	2
8642	74	2	0	0	0	1	1	0	0	0	0	0	0	0	0	2	4

8644	75	1	0	1	1	0	3	1	1	1	0	0	0	0	1	1	4
8646	71	2	0	0	0	0	1	1	1	0	0	0	0	0	0	4	1
8648	69	1	0	0	0	1	3	#NULL!	#NULL!	#NULL!	0	0	#NULL!	0	0	2	4
8663	70	2	0	0	0	0	1	1	0	1	0	0	0	0	1	2	3
8666	70	1	0	0	0	1	1	2	0	1	0	0	0	1	1	1	4
8669	73	1	0	0	0	0	1	1	0	1	1	0	0	0	1	1	4
8670	73	2	0	0	0	0	1	0	0	0	0	1	0	0	0	1	2
8672	73	2	0	0	0	0	2	0	0	0	0	0	0	0	0	2	3
8673	69	1	0	0	0	0	1	0	0	1	1	0	0	0	0	2	4
8674	69	1	0	0	0	0	3	2	1	1	0	1	0	0	0	1	4
8678	77	1	0	0	0	1	3	1	0	1	0	0	0	0	0	1	4
8679	77	2	0	0	1	1	3	0	0	0	0	0	0	0	0	1	4
8682	78	2	0	1	0	1	1	#NULL!	#NULL!	#NULL!	1	0	#NULL!	0	1	1	4
8685	72	1	0	0	0	0	1	1	1	1	1	0	1	0	0	2	4
8686	65	2	0	0	0	1	1	1	0	0	0	0	0	0	0	4	3
8687	79	2	1	1	0	0	3	0	0	0	1	0	1	0	0	2	4
8688	70	1	0	0	0	1	1	1	0	1	0	0	0	1	0	2	4
8689	67	1	0	0	0	1	1	0	0	1	0	0	0	0	1	3	4
8690	67	2	0	0	0	1	1	2	0	0	0	0	0	0	1	2	4
8693	68	1	0	1	0	1	1	0	0	1	1	1	0	0	0	1	4
8694	68	2	0	1	0	0	1	0	0	0	1	0	0	0	0	1	4
8695	75	2	0	1	0	1	2	0	0	0	0	0	0	0	0	3	2
8697	72	1	0	0	0	1	1	2	1	1	0	0	0	0	0	2	4
8699	67	1	0	0	0	#NULL!	1	1	1	0	0	0	0	0	1	2	4
8700	65	2	0	0	0	#NULL!	1	1	1	0	1	1	0	0	1	2	2
8704	73	1	0	0	0	0	1	#NULL!	1	1	0	0	0	0	0	1	4
8706	66	1	0	0	0	0	1	0	1	0	0	0	0	0	0	1	4
8707	71	1	0	0	0	0	1	1	0	0	1	0	0	0	0	4	1
8708	67	2	0	0	0	0	1	1	0	0	0	0	0	0	0	4	1
8709	66	2	0	0	0	0	1	1	0	0	0	0	0	0	0	2	2
8711	77	2	0	1	0	1	1	0	0	0	0	0	0	0	1	2	3
8712	66	2	0	0	0	1	1	0	1	1	0	0	1	0	0	4	1
8713	65	1	0	0	0	1	1	0	1	1	0	0	0	0	1	1	4
8714	77	1	0	0	0	0	1	1	0	1	0	1	0	1	0	2	4
8715	75	2	0	0	0	0	1	#NULL!	#NULL!	#NULL!	0	0	#NULL!	0	0	2	1
8716	74	1	0	0	0	1	3	1	1	1	1	1	1	0	0	1	4
8717	71	2	0	0	1	1	2	0	0	0	1	0	0	0	0	2	3
8720	67	1	0	0	0	1	1	0	0	0	1	0	0	0	1	1	4
8727	71	1	0	#NULL!	0	#NULL!	1	#NULL!	#NULL!	#NULL!	1	0	#NULL!	0	0	1	4

8729	72	2	0	0	0	0	1	0	0	0	1	0	0	0	0	4	1
8730	70	2	0	0	0	0	1	0	0	0	1	1	0	0	0	2	1
8731	82	2	0	1	0	0	1	0	0	0	1	0	0	0	0	2	2
8733	72	1	0	0	1	0	1	0	0	0	1	0	#NULL!	0	0	3	4
8735	70	2	0	1	0	1	1	0	0	0	1	0	0	0	1	2	4
8737	70	2	0	0	0	1	1	0	0	0	0	0	0	0	0	2	4
8738	83	1	0	0	1	1	1	2	0	1	1	1	0	0	0	4	1
8741	85	2	0	1	0	0	2	0	0	0	0	0	0	0	1	2	4
8744	67	1	0	0	0	#NULL!	1	2	0	1	0	0	0	0	1	2	4
8745	82	2	0	1	0	1	1	1	0	0	0	0	0	0	1	3	4
8747	85	2	0	#NULL!	1	1	1	#NULL!	#NULL!	#NULL!	1	0	#NULL!	0	0	2	4
8750	73	2	0	1	0	0	1	0	0	0	0	0	0	0	0	1	4
8751	82	1	0	0	0	0	1	1	1	1	0	0	0	0	0	1	4
8759	77	2	0	1	0	1	1	1	0	0	0	1	0	0	0	1	3
8760	78	1	0	1	0	0	1	1	0	1	1	0	0	0	1	1	4
8762	65	2	0	0	0	0	3	0	1	1	0	0	0	0	0	2	4
8763	69	2	0	0	1	0	3	2	0	0	0	0	0	0	0	3	2
8764	68	1	0	0	0	0	3	1	0	0	0	0	0	0	1	1	4
8765	67	1	0	0	0	0	1	0	1	1	0	0	0	0	1	3	4
8768	66	1	0	0	0	0	1	0	1	1	0	0	0	0	0	1	4
8769	68	2	0	1	0	0	1	0	0	0	1	0	0	0	1	3	2
8772	74	1	0	0	0	1	3	2	0	1	0	0	1	0	0	1	4
8774	84	1	0	0	0	1	3	0	1	1	1	1	1	0	0	3	4
8777	79	2	0	1	0	0	1	1	0	0	0	0	0	0	0	4	1
8779	89	2	1	1	1	0	1	1	0	0	0	0	0	1	0	2	1
8780	73	1	0	0	0	0	1	#NULL!	#NULL!	#NULL!	1	0	#NULL!	0	0	1	4
8781	70	2	0	0	0	0	1	0	0	0	1	0	0	0	0	2	1
8784	76	2	0	1	0	1	3	2	0	0	0	0	0	0	0	2	4
8785	72	1	0	0	0	0	1	0	0	0	1	0	0	0	0	1	4
8786	67	2	0	0	0	0	2	0	0	0	1	0	0	0	0	4	1
8787	67	1	0	0	0	0	1	0	0	0	0	0	0	0	1	1	4
8791	77	2	0	1	0	0	2	1	0	0	0	0	1	0	0	1	3
8792	73	1	0	0	0	0	1	1	1	1	1	1	0	0	0	2	4
8796	67	1	0	0	0	1	1	0	0	0	1	0	0	0	0	1	2
8800	66	1	0	0	0	1	3	1	0	1	0	0	0	0	0	1	4
8805	86	2	0	1	1	1	1	1	0	0	0	0	0	0	0	4	1
8808	72	1	0	0	0	1	1	1	1	1	1	0	0	0	0	3	4
8809	68	2	0	0	1	1	3	#NULL!	1	0	0	1	0	0	0	3	2
8810	72	1	0	0	1	1	1	#NULL!	#NULL!	#NULL!	0	0	#NULL!	1	0	3	4

8811	65	2	0	0	0	0	1	0	0	0	1	0	0	0	1	1	4
8815	71	1	0	0	1	1	1	1	0	1	0	0	0	1	1	3	1
8819	76	1	0	0	0	0	1	0	1	1	1	0	0	0	1	1	4
8820	71	2	0	0	0	0	1	1	0	0	1	0	0	0	0	4	1
8821	65	1	0	0	1	1	1	#NULL!	0	1	0	0	0	0	1	3	4
8823	65	1	0	0	0	0	3	1	0	1	0	1	0	0	0	1	4
8826	72	2	1	1	1	1	1	1	0	0	1	1	0	0	0	3	4
8832	76	2	0	0	0	0	1	0	0	0	0	0	0	0	0	3	4
8835	66	2	0	0	0	0	3	0	0	0	0	1	0	0	0	1	4
8836	75	1	1	0	1	1	3	0	0	1	0	0	1	1	0	1	4
8837	74	2	0	0	0	1	1	#NULL!	#NULL!	#NULL!	1	0	#NULL!	0	0	2	2
8841	72	2	0	1	1	1	3	1	0	0	0	0	0	0	0	2	4
8842	65	1	0	0	0	0	3	1	1	1	0	0	0	0	1	1	4
8843	73	1	0	0	0	1	3	#NULL!	#NULL!	#NULL!	0	0	#NULL!	1	0	3	1
8846	69	2	0	1	0	0	1	#NULL!	0	0	0	0	0	0	1	4	1
8851	83	1	1	0	0	0	1	2	1	1	0	0	0	0	0	2	3
8855	72	1	1	0	0	0	1	0	1	1	1	1	0	0	0	2	4
8856	68	2	0	0	0	0	2	0	0	0	1	0	0	0	1	4	1
8858	81	2	0	0	1	0	1	1	0	0	0	0	1	0	0	3	1
8859	73	2	0	#NULL!	1	#NULL!	1	1	0	0	0	0	1	0	0	3	4
8866	81	1	0	0	0	1	2	0	0	1	0	0	0	0	1	1	4
8867	81	2	0	0	0	1	1	1	0	0	0	0	0	0	0	1	1
8870	72	1	0	0	0	1	1	2	1	1	1	0	0	0	0	2	4
8871	68	2	0	0	1	1	1	1	0	0	0	0	0	0	0	3	2
8872	78	1	0	0	0	1	1	1	1	1	0	0	0	1	0	1	4
8873	74	2	0	0	0	1	2	1	0	0	0	0	0	0	0	2	2
8876	72	2	0	1	1	0	1	1	0	0	0	0	0	0	0	3	4
8882	81	2	0	0	0	1	2	#NULL!	0	0	1	0	#NULL!	0	0	1	3
8888	87	1	1	1	0	1	1	1	0	0	0	0	0	0	0	2	4
8890	79	2	0	0	0	1	1	0	0	0	0	0	0	0	1	4	1
8891	66	1	0	0	0	1	1	0	0	1	1	0	0	1	1	1	4
8894	66	1	0	1	1	0	1	0	1	1	0	0	0	0	1	2	4
8895	65	1	0	1	1	1	1	0	1	1	1	1	0	0	1	1	4
8899	70	1	0	0	0	#NULL!	1	1	0	1	0	0	1	0	1	1	4
8900	80	1	0	0	1	0	3	#NULL!	1	1	0	1	0	0	0	3	4
8902	78	1	0	1	0	0	1	1	1	1	0	0	0	0	0	1	4
8905	80	1	1	1	0	1	1	2	0	0	0	1	0	0	0	1	4
8906	77	2	0	0	0	1	1	1	0	0	1	1	0	0	0	1	2
8908	69	2	0	0	0	1	1	#NULL!	0	1	0	0	0	0	1	4	1

8910	72	1	0	0	0	1	3	2	0	1	0	0	0	0	0	3	4
8911	71	2	0	0	1	1	3	0	0	0	0	0	1	0	0	2	4
8917	84	1	0	1	1	0	3	1	0	1	0	0	0	0	0	3	4
8921	76	2	1	1	1	1	3	2	0	0	0	0	1	1	0	4	1
8923	84	1	0	0	1	1	1	0	0	1	0	0	1	1	1	1	4
8927	71	1	0	0	0	1	1	1	1	1	0	0	0	0	1	1	4
8929	67	1	0	0	0	1	1	1	0	1	0	0	1	0	0	1	4
8930	93	1	0	1	1	1	3	2	0	1	0	0	0	0	0	3	4
8932	69	1	0	0	0	0	1	0	1	1	1	1	0	0	0	2	4
8934	70	2	0	0	1	1	1	0	0	1	1	0	0	0	0	2	3
8935	70	1	1	0	1	0	1	0	1	1	1	1	0	0	1	3	4
8936	71	1	0	0	0	1	1	2	1	1	1	0	0	0	0	2	3
8937	71	2	0	0	0	1	1	1	0	0	0	0	1	0	0	2	2
8940	78	2	0	1	0	1	1	1	0	0	0	0	0	0	0	1	4
8941	77	2	1	1	0	1	1	0	0	0	1	0	0	0	0	3	4
8942	69	1	0	0	0	0	1	1	0	0	0	0	0	0	0	2	4
8945	65	1	0	0	1	1	1	#NULL!	0	1	0	0	0	0	0	1	4
8947	68	1	0	0	1	0	3	1	0	1	1	0	0	0	1	3	4
8948	66	1	0	0	1	1	3	1	0	1	0	0	0	0	0	1	4
8950	69	1	0	0	0	0	1	1	0	1	1	0	0	0	0	1	4
8952	72	2	0	1	0	1	1	0	1	0	1	0	1	0	0	2	2
8953	65	1	0	0	0	1	1	0	0	1	0	0	0	0	0	1	4
8957	68	1	0	0	0	1	3	#NULL!	#NULL!	#NULL!	0	0	#NULL!	0	1	4	1
8958	66	2	0	0	0	1	3	1	0	0	0	1	0	0	0	2	1
8961	69	2	0	1	0	1	1	0	0	0	0	0	0	0	0	2	2
8962	67	1	0	0	0	1	1	0	0	1	1	1	0	0	0	2	4
8963	78	2	0	1	0	1	1	1	0	0	1	1	0	0	1	2	4
8965	78	1	0	0	1	0	1	2	1	1	0	0	0	0	0	3	4
8967	75	1	0	0	0	0	1	1	0	1	1	0	0	1	0	1	4
8968	70	2	0	0	0	0	1	0	0	0	0	1	0	0	0	2	2
8972	71	1	0	0	0	1	1	1	0	1	0	0	0	0	0	1	4
8973	67	2	0	0	0	1	3	2	0	0	0	1	0	0	0	3	4
8975	69	1	0	1	0	0	1	0	0	1	0	0	0	0	0	1	4
8976	70	2	1	0	1	1	3	#NULL!	0	1	0	0	1	0	0	3	3
8977	71	1	0	0	0	0	1	1	1	1	1	0	0	0	0	2	4
8978	67	2	0	#NULL!	0	#NULL!	1	#NULL!	#NULL!	#NULL!	0	0	#NULL!	0	1	2	4
8979	70	1	0	0	0	1	1	2	0	0	0	1	0	0	0	2	4
8981	67	2	0	0	0	1	1	0	0	0	1	0	0	0	0	2	4
8987	68	2	0	1	0	1	1	1	0	0	0	0	1	0	1	2	4

8988	85	2	0	1	1	0	1	1	0	0	0	0	0	0	0	2	4
8990	81	1	0	0	0	1	1	1	0	0	1	0	0	0	0	2	4
8991	76	2	1	0	0	1	1	0	0	1	0	0	0	0	0	2	4
8992	82	2	0	0	0	1	1	1	0	0	0	1	0	0	0	2	3
8993	81	1	0	0	0	0	#NULL!	2	0	0	0	0	1	0	0	1	4
8999	65	2	0	0	0	1	1	0	0	0	1	0	0	0	0	4	1
9000	85	2	0	0	#NULL!	1	1	0	0	0	0	0	0	0	0	3	1
9002	66	2	0	0	0	#NULL!	1	0	0	0	1	0	1	0	0	2	2
9005	70	1	0	0	0	1	1	1	0	1	0	0	0	1	0	1	4
9006	72	1	0	0	0	1	1	1	0	0	1	0	0	0	0	2	4
9007	70	2	0	0	0	1	3	2	0	0	0	0	0	0	0	1	2
9008	82	2	1	1	0	0	1	1	0	0	1	0	1	0	0	4	2
9009	73	2	0	1	0	1	3	1	0	0	1	0	1	0	0	2	1
9014	67	2	0	1	0	1	1	1	0	0	0	0	0	0	1	3	4
9015	72	1	0	1	1	0	1	1	1	0	1	0	0	0	0	3	4
9018	88	2	0	1	0	0	1	1	0	0	0	0	0	0	0	1	2
9019	74	1	1	0	1	1	3	1	0	1	0	1	0	0	0	1	4
9020	72	2	0	0	0	1	1	1	0	0	0	0	0	0	0	2	2
9021	80	1	0	0	0	0	1	#NULL!	0	1	0	0	0	1	0	1	4
9022	79	2	1	0	0	0	1	#NULL!	#NULL!	0	0	0	0	0	0	4	1
9023	74	2	0	1	0	1	1	#NULL!	#NULL!	#NULL!	1	0	#NULL!	0	1	3	4
9024	77	1	0	1	1	1	1	0	0	0	0	0	1	0	0	3	4
9028	66	2	0	0	0	1	1	1	0	0	1	0	0	0	0	4	1
9029	85	2	0	1	1	#NULL!	1	0	0	0	0	0	0	0	0	4	2
9030	83	2	0	1	0	0	1	0	0	0	0	0	0	0	0	1	4
9032	68	2	0	0	0	1	1	2	0	0	1	0	0	0	0	2	3
9033	70	1	1	0	0	1	1	0	1	1	1	0	0	1	0	1	4
9034	73	1	1	0	0	1	1	0	1	1	1	0	0	1	0	2	4
9035	68	2	0	0	0	0	1	0	0	0	1	0	0	0	0	2	4
9036	80	1	0	0	0	1	1	1	0	1	1	1	0	0	1	2	4
9037	77	2	0	0	1	1	3	1	0	0	0	0	0	0	1	2	4
9039	65	2	0	1	0	1	1	1	0	0	0	0	0	0	1	2	3
9041	84	2	0	#NULL!	0	#NULL!	1	1	1	1	0	0	0	0	0	3	4
9042	66	1	0	0	0	1	3	1	1	1	0	0	1	0	1	4	4
9047	75	1	1	0	0	1	3	1	0	1	0	0	0	0	0	3	4
9048	73	2	0	0	0	#NULL!	1	1	0	0	0	0	0	0	0	2	2
9049	86	2	0	1	1	0	1	1	1	0	0	0	0	1	0	3	4
9050	87	2	1	1	0	1	1	1	0	0	0	0	1	0	0	2	3
9052	86	1	0	1	0	1	1	1	0	0	0	0	0	0	1	3	4

9053	72	2	1	1	1	1	3	0	0	0	0	1	0	0	1	3	4
9054	78	1	0	1	0	0	1	1	1	1	0	0	0	0	0	1	4
9058	66	1	0	0	0	1	1	1	#NULL!	#NULL!	0	1	#NULL!	0	1	1	4
9059	74	2	0	1	0	0	1	1	0	0	0	0	0	0	0	3	3
9070	66	1	0	0	0	0	2	0	1	1	0	0	0	0	0	1	4
9071	71	1	0	0	0	0	1	2	0	0	1	0	0	0	0	1	4
9072	65	2	0	0	0	0	1	0	0	0	0	0	0	0	0	4	1
9075	66	1	0	0	0	1	1	#NULL!	0	0	0	0	0	0	0	2	4
9076	76	1	0	0	0	1	1	1	0	1	0	1	0	0	0	1	4
9077	70	2	0	0	0	1	1	0	0	0	0	0	0	0	0	2	2
9079	67	2	0	0	0	1	3	1	0	0	1	0	0	0	0	2	2
9081	66	1	0	0	0	0	1	2	0	1	1	0	0	0	1	1	4
9082	70	2	0	1	0	0	2	0	0	0	0	0	0	0	0	2	3
9085	76	1	0	0	0	1	1	1	1	0	0	1	0	0	0	1	4
9090	67	1	0	0	1	0	1	0	1	1	1	0	0	0	1	1	4
9094	67	2	0	1	0	0	1	0	0	0	1	0	0	0	1	2	4
9096	70	1	0	0	0	0	1	0	1	1	0	0	0	0	0	2	4
9097	69	1	0	0	0	0	1	2	0	0	0	0	0	0	0	1	4
9098	65	2	1	0	0	0	1	0	0	0	0	0	0	0	0	2	2
9099	70	2	0	1	1	0	1	0	0	1	0	0	0	0	0	2	4
9101	66	1	0	0	0	0	1	1	0	1	1	0	0	0	0	1	4
9103	66	1	0	0	0	0	1	1	1	1	1	0	0	0	1	1	4
9104	66	2	0	0	0	0	1	0	0	0	0	0	0	0	0	1	2
9109	69	1	0	0	0	0	3	1	0	1	1	0	1	0	0	3	4
9111	77	2	1	0	0	0	3	0	0	0	0	0	0	0	0	1	4
9113	65	1	0	0	0	1	1	1	1	0	0	0	0	0	0	2	4
9117	77	1	0	0	0	1	1	1	1	1	0	0	1	0	0	1	4
9121	65	1	0	0	0	1	1	#NULL!	#NULL!	#NULL!	0	0	#NULL!	0	0	3	4
9122	75	2	0	0	0	1	1	1	0	0	0	0	1	0	0	3	2
9123	75	1	1	0	0	1	1	2	0	1	0	0	0	0	0	1	2
9127	66	1	0	0	0	0	1	0	0	1	0	0	0	0	0	1	4
9129	72	2	0	0	0	#NULL!	1	1	0	0	1	1	0	0	0	4	1
9132	66	1	0	0	0	1	3	2	1	1	0	0	0	0	1	2	4
9137	73	1	0	0	0	0	2	0	1	0	0	0	0	0	0	3	4
9138	68	2	0	0	0	0	1	#NULL!	0	0	1	0	0	0	1	2	1
9145	70	2	0	0	0	1	1	0	0	0	0	0	0	0	0	2	4
9148	68	2	0	#NULL!	0	1	1	0	0	0	0	0	0	0	1	2	4
9150	74	2	0	0	1	0	1	#NULL!	0	0	1	0	0	0	1	1	3
9157	66	1	0	0	0	0	1	1	0	1	0	0	0	0	0	2	4

9158	74	1	0	0	0	0	1	0	0	0	0	0	0	0	0	1	4
9159	71	2	0	0	0	0	3	1	0	0	0	0	0	0	0	2	4
9162	65	2	0	0	0	1	1	0	0	0	0	0	0	0	1	2	4
9163	69	1	0	0	0	0	1	0	0	1	0	0	0	0	1	1	4
9164	65	2	0	0	0	1	1	0	0	0	0	0	0	0	0	2	2
9165	82	1	0	0	1	0	1	1	0	0	0	0	0	0	0	3	4
9166	77	2	0	0	0	0	1	0	0	0	1	0	0	0	0	2	4
9167	71	1	0	0	#NULL!	#NULL!	1	2	0	1	0	0	0	0	1	1	2
9168	70	2	0	0	0	0	1	0	0	0	1	0	0	0	0	2	2
9169	81	1	0	0	0	1	1	#NULL!	#NULL!	#NULL!	1	1	#NULL!	0	0	1	4
9170	75	2	0	0	0	0	1	0	0	0	1	0	0	0	0	1	4
9172	72	1	1	0	0	1	3	2	1	0	0	0	1	1	0	1	4
9173	66	2	0	0	0	1	1	1	0	0	0	0	0	0	0	3	1
9174	65	1	0	0	0	1	#NULL!	1	0	0	0	0	#NULL!	0	1	2	4
9178	70	2	0	0	0	1	1	0	0	0	0	0	0	0	0	3	4
9182	67	1	0	0	0	0	1	1	1	0	1	0	0	0	1	3	4
9183	66	1	0	0	0	1	1	1	0	1	0	0	0	1	1	2	4
9184	67	1	0	0	0	1	1	1	1	1	0	0	0	0	1	1	4
9185	66	2	0	0	0	1	1	1	1	0	0	0	0	0	0	1	2
9186	65	1	0	0	0	1	1	0	0	0	1	0	0	0	1	2	4
9187	74	2	0	0	1	1	1	0	0	0	0	0	0	0	0	4	1
9188	70	1	0	0	1	1	1	1	0	1	0	1	0	0	0	4	1
9190	65	2	0	0	0	1	1	0	0	0	1	0	0	0	0	2	3
9191	66	1	0	0	0	0	3	0	1	1	0	0	0	0	1	3	4
9192	76	2	0	0	0	1	1	0	0	0	0	0	0	0	0	1	4
9193	71	2	0	1	0	1	1	0	0	0	1	0	0	0	0	2	4
9194	69	1	0	0	1	0	1	2	0	1	0	0	0	0	0	2	4
9197	65	1	0	0	0	0	1	0	0	1	1	0	1	1	0	1	4
9201	65	2	0	1	0	0	3	0	0	0	1	0	0	0	1	2	2
9203	66	1	0	0	0	1	1	1	0	0	0	1	0	0	1	1	4
9204	66	2	0	1	0	1	1	0	0	0	1	0	0	0	0	2	4
9209	70	1	0	0	0	0	1	0	1	1	1	1	0	0	1	1	2
9210	66	2	0	0	0	1	3	1	0	0	1	0	0	0	0	2	2
9211	74	1	0	0	0	0	1	1	0	1	0	0	1	1	0	1	4
9214	66	1	0	0	0	1	3	0	0	0	0	0	1	1	0	1	4
9219	86	1	1	0	0	#NULL!	1	0	0	1	0	0	0	1	0	1	4
9220	84	2	0	0	0	#NULL!	1	#NULL!	0	0	0	0	0	0	0	2	3
9222	72	2	1	0	1	1	#NULL!	1	0	0	0	1	1	0	1	2	4
9224	77	2	0	0	0	1	1	1	0	0	1	0	0	0	0	4	1

9226	80	2	0	0	0	0	1	1	0	0	0	0	0	0	0	4	1
9228	69	1	0	0	0	1	1	0	0	0	0	0	0	0	0	3	4
9231	74	1	0	0	0	1	1	2	0	1	0	0	1	0	0	2	4
9232	72	2	1	0	0	1	1	0	0	0	1	0	0	0	0	3	2
9235	80	2	0	0	1	0	1	1	0	0	0	0	0	0	0	1	3
9236	65	1	0	0	0	0	1	1	0	1	1	0	0	1	1	1	4
9239	74	1	1	0	0	1	1	0	1	1	0	0	0	0	0	1	4
9240	83	2	1	0	0	0	1	#NULL!	0	0	0	0	0	0	0	4	1
9245	76	1	0	0	1	1	1	1	0	1	0	0	0	0	0	1	4
9246	74	2	0	0	1	0	3	0	0	0	0	0	0	0	0	3	3
9251	73	1	1	0	0	0	3	1	0	0	1	0	0	0	0	2	4
9253	67	2	0	0	0	0	1	1	0	0	0	1	0	0	0	3	2
9258	90	2	1	1	0	1	2	0	0	0	0	0	1	0	0	4	2
9261	65	2	0	0	0	1	1	1	0	0	1	0	0	0	0	1	3
9263	74	2	0	0	0	0	3	1	0	0	0	0	0	0	0	2	3
9264	72	2	0	1	0	1	3	1	0	0	0	0	0	1	0	4	1
9265	76	1	0	0	0	0	1	1	1	1	0	0	0	0	0	1	4
9267	71	1	0	0	0	0	1	2	1	1	1	0	0	0	0	2	4
9269	65	1	0	0	0	0	1	1	0	1	1	1	0	0	0	1	4
9273	71	1	0	0	1	1	1	1	1	1	0	0	0	0	0	3	4
9275	65	1	0	0	1	0	1	1	1	1	0	0	0	0	1	3	4
9277	69	1	0	0	0	0	3	0	0	0	0	0	0	0	1	1	4
9278	80	1	1	0	#NULL!	0	#NULL!	2	1	1	1	0	0	0	0	1	4
9279	77	2	1	0	1	1	3	1	0	0	1	0	0	0	0	4	1
9280	67	1	0	0	0	1	3	1	0	1	0	0	0	0	0	1	4
9281	66	2	0	0	0	1	1	2	0	0	0	0	0	0	0	4	1
9282	74	2	0	1	0	1	2	1	0	0	1	0	0	0	0	2	2
9289	73	2	1	0	1	#NULL!	#NULL!	1	0	0	1	0	1	0	0	2	2
9290	79	2	1	1	1	1	1	2	0	0	0	0	1	0	0	2	4
9293	82	2	0	#NULL!	1	1	#NULL!	0	0	1	0	0	#NULL!	0	0	1	4
9297	71	1	0	0	0	0	1	0	1	0	0	0	0	0	1	3	4
9303	79	1	0	0	0	0	1	0	1	1	1	0	1	0	0	1	4
9304	74	2	0	0	0	0	1	1	0	0	0	1	1	0	0	2	3
9305	80	1	1	1	0	0	1	0	0	1	1	0	1	0	0	1	4
9306	75	1	0	0	0	1	1	0	1	0	1	1	0	0	0	3	4
9307	67	1	0	1	0	1	3	0	0	1	0	0	0	0	1	3	4
9310	67	2	0	1	0	1	1	1	0	0	0	0	0	0	0	2	4
9311	83	2	1	1	1	1	1	0	0	0	0	0	0	0	0	4	1
9313	65	2	0	1	0	0	1	1	0	0	0	1	0	0	0	2	4

9318	77	1	0	0	1	0	1	1	1	1	1	1	0	0	0	3	4
9319	72	2	0	0	1	0	1	0	0	0	1	1	0	0	0	3	2
9321	74	1	0	0	0	1	2	1	1	1	0	0	0	0	0	2	4
9323	86	2	0	0	1	0	1	1	0	0	0	1	1	0	0	2	4
9324	66	1	0	0	1	1	3	2	1	1	0	0	0	0	1	3	4
9325	65	2	0	1	1	0	1	#NULL!	0	0	0	0	0	0	0	2	3
9327	72	1	0	0	1	1	3	0	0	1	0	0	0	0	0	1	4
9328	69	2	0	0	1	1	1	0	0	0	0	0	0	0	1	1	4
9333	76	2	1	#NULL!	0	#NULL!	1	#NULL!	#NULL!	#NULL!	0	0	#NULL!	0	1	3	3
9334	70	1	0	0	0	1	1	#NULL!	1	1	1	0	1	0	0	1	4
9336	67	1	0	0	0	1	1	1	1	1	0	0	0	0	0	1	4
9342	69	1	0	0	0	1	1	2	0	1	1	0	0	0	0	2	4
9344	68	1	0	0	0	1	1	1	1	1	1	0	0	0	0	2	4
9345	67	2	0	0	0	1	1	1	0	0	1	0	0	0	0	2	4
9348	67	1	0	0	0	1	1	1	0	1	0	0	0	0	1	1	4
9349	67	2	1	0	0	1	1	#NULL!	#NULL!	#NULL!	0	0	#NULL!	0	1	2	2
9350	73	2	0	0	1	1	3	0	0	1	1	0	1	0	0	2	2
9358	72	1	0	0	1	0	1	1	1	1	1	1	0	0	0	2	4
9359	66	2	0	0	0	1	1	0	0	0	0	0	0	0	0	2	2
9361	72	2	0	0	0	1	1	0	0	0	1	0	1	0	0	2	2
9365	72	1	0	0	0	1	1	#NULL!	#NULL!	#NULL!	1	0	#NULL!	0	1	2	4
9366	77	2	0	1	0	1	1	1	0	1	1	1	1	0	1	3	2
9367	76	1	0	0	0	1	1	2	1	1	0	1	0	0	1	1	4
9368	68	2	0	0	0	1	3	2	0	0	0	0	0	0	1	3	2
9369	75	1	0	0	0	1	1	0	0	0	0	0	0	0	0	3	4
9370	70	2	0	0	1	#NULL!	1	0	0	0	1	0	0	0	0	2	4
9371	77	1	0	0	0	0	1	1	0	1	1	1	0	0	0	1	4
9372	71	2	0	0	1	0	1	1	0	0	1	1	0	0	0	2	2
9375	70	1	0	0	0	1	1	0	0	0	0	0	1	0	0	1	4
9376	76	1	0	0	0	0	3	1	1	1	1	1	0	0	1	1	4
9377	75	2	0	0	0	0	1	0	0	0	1	1	0	0	0	2	2
9378	72	1	0	0	0	1	1	#NULL!	0	1	0	0	0	0	1	1	4
9379	71	2	0	0	0	1	1	2	0	0	0	1	0	0	0	2	2
9381	68	2	0	1	0	0	3	2	0	0	0	0	1	0	0	4	1
9383	66	1	0	0	0	1	1	0	0	1	1	1	0	0	0	3	4
9385	70	1	0	0	0	1	1	1	0	0	0	0	0	0	1	1	4
9386	67	2	1	0	0	1	3	2	0	0	0	0	0	1	0	2	2
9396	70	2	0	0	1	1	1	1	0	0	0	0	0	0	0	2	4
9397	65	1	1	0	0	1	3	1	1	1	0	0	0	1	1	1	4

9398	65	2	0	1	0	1	1	0	1	0	0	0	0	0	0	1	3
9400	68	2	0	1	1	1	1	0	0	1	0	0	0	0	0	3	4
9402	73	1	0	0	1	0	1	1	1	1	0	0	0	0	1	3	4
9403	68	2	0	1	1	#NULL!	3	#NULL!	0	0	0	0	0	0	0	4	1
9405	69	2	0	0	1	1	1	1	0	0	1	0	0	0	0	4	3
9409	76	2	0	0	0	0	2	1	1	0	1	0	0	0	0	3	2
9410	86	2	0	1	#NULL!	#NULL!	1	1	0	0	0	1	1	0	0	4	1
9411	74	1	1	0	0	1	1	0	0	1	0	0	1	0	0	1	4
9412	70	2	0	0	0	0	2	0	0	1	0	0	0	0	0	4	4
9413	89	2	0	0	0	1	1	1	0	0	0	0	1	0	0	2	3
9417	78	2	1	1	0	1	3	0	0	1	0	0	1	1	0	2	4
9419	78	2	0	0	#NULL!	1	#NULL!	1	0	0	0	0	0	0	0	3	2
9420	78	2	1	0	0	1	3	0	0	0	0	1	1	0	0	1	4
9421	77	1	1	0	1	1	1	1	0	1	1	0	1	0	0	2	4
9427	76	1	0	0	1	1	1	1	1	1	0	0	0	0	0	1	4
9428	70	2	0	0	1	1	1	0	0	0	1	1	0	0	1	3	4
9431	81	1	1	0	0	0	3	0	1	1	1	1	1	0	1	1	4
9432	81	2	1	0	0	0	1	0	0	0	1	0	0	0	0	2	2
9433	73	1	0	0	0	1	1	1	1	1	0	0	0	0	1	1	4
9436	67	2	0	1	0	1	1	0	0	0	0	0	1	0	0	4	1
9437	65	1	0	0	0	1	1	#NULL!	1	1	0	0	0	0	0	2	4
9438	74	2	0	1	0	1	1	2	0	0	0	0	1	0	0	3	2
9442	76	2	0	0	0	1	3	1	0	0	0	1	1	0	0	4	1
9443	74	1	0	0	0	1	1	0	0	0	0	1	1	0	0	3	4
9446	78	2	0	1	0	0	1	0	0	0	1	1	0	0	0	2	2
9447	67	1	0	0	0	0	1	0	0	1	1	1	0	0	0	1	4
9452	83	2	0	0	0	0	#NULL!	1	0	0	0	0	0	0	0	2	1
9454	65	1	0	0	0	1	1	2	0	1	0	0	1	0	0	2	4
9455	70	2	1	1	1	1	3	0	0	0	0	0	0	0	0	2	4
9457	74	2	0	0	0	0	3	1	0	0	0	1	1	0	0	4	1
9460	81	1	0	1	1	1	1	2	1	1	1	0	0	0	0	1	4
9465	76	1	0	0	0	1	3	1	0	1	1	0	0	0	0	2	4
9466	74	2	0	0	0	1	1	1	0	0	0	1	0	0	0	2	1
9467	72	1	0	0	0	1	1	#NULL!	1	1	0	0	0	0	1	2	4
9468	68	2	0	0	0	1	1	0	1	1	0	0	1	0	0	2	4
9469	70	2	0	1	0	0	1	1	0	0	0	0	0	0	0	4	1
9471	81	2	0	1	0	#NULL!	1	#NULL!	0	0	0	1	0	0	0	1	3
9474	66	1	0	0	0	0	1	1	0	0	0	0	0	0	1	1	4
9477	67	2	0	0	0	1	2	0	0	0	0	0	0	0	0	2	2

9479	79	1	0	0	0	#NULL!	1	1	0	1	0	1	0	0	0	1	4
9480	74	2	0	0	0	1	2	1	0	0	0	0	0	0	0	2	2
9483	68	2	0	0	0	0	1	1	0	0	0	0	0	0	0	1	2
9484	71	2	0	1	0	1	3	2	0	0	#NULL!	#NULL!	0	0	1	1	4
9486	81	2	0	#NULL!	0	#NULL!	1	#NULL!	#NULL!	#NULL!	0	0	#NULL!	1	0	4	1
9487	72	1	0	0	0	0	3	1	0	1	1	1	0	0	1	2	4
9488	67	2	0	0	1	#NULL!	1	0	#NULL!	0	1	1	0	0	1	1	4
9489	78	1	1	0	1	1	1	1	0	1	1	0	0	0	1	3	4
9490	74	2	0	0	0	1	1	0	0	0	1	0	0	0	0	3	3
9491	82	2	0	1	0	1	1	1	0	0	0	0	0	0	0	4	1
9492	73	2	0	0	0	#NULL!	3	#NULL!	0	0	1	0	0	0	1	2	2
9493	72	1	0	0	0	0	3	1	1	1	0	1	0	0	0	1	4
9495	71	1	0	0	0	0	1	0	1	1	1	1	0	1	1	1	4
9497	82	2	0	1	1	0	1	0	0	0	0	0	0	1	0	1	4
9498	74	1	0	0	0	1	1	1	1	1	0	0	0	0	1	4	4
9499	72	2	0	0	0	1	1	#NULL!	0	#NULL!	0	0	0	0	0	4	1
9500	77	1	0	0	1	1	3	#NULL!	#NULL!	#NULL!	0	0	#NULL!	0	0	3	4
9501	75	2	0	0	0	1	1	1	0	0	0	0	0	0	0	1	3
9502	79	2	0	1	0	1	1	0	0	0	0	0	0	0	0	2	2
9506	73	2	0	1	0	0	3	0	0	0	0	0	0	0	1	2	4
9507	80	2	0	1	#NULL!	0	#NULL!	1	0	0	0	0	0	0	0	2	1
9513	87	2	0	1	0	1	2	2	1	1	0	0	1	0	0	2	4
9518	82	2	0	0	1	1	1	1	0	0	0	0	1	0	0	2	4
9519	81	1	0	0	1	1	1	0	0	1	1	0	1	0	0	2	3
9520	67	2	0	1	1	1	1	0	0	0	0	0	0	0	1	2	2
9523	65	2	0	0	0	1	1	0	0	0	0	1	1	0	0	2	3
9525	70	2	1	0	0	0	1	0	0	0	0	0	0	0	0	1	4
9526	65	1	0	0	0	1	1	1	1	1	0	0	0	0	0	1	4
9527	71	1	0	0	0	1	1	1	0	1	1	0	0	0	1	1	4
9528	66	2	0	0	0	1	1	0	0	0	1	0	0	0	0	3	2
9530	70	1	0	#NULL!	0	#NULL!	1	#NULL!	#NULL!	#NULL!	0	0	#NULL!	0	0	1	4
9531	65	2	0	0	0	1	1	0	0	0	1	0	1	0	0	2	3
9532	73	1	0	0	0	0	1	0	1	0	1	0	0	0	0	2	4
9534	76	2	0	0	0	0	1	1	0	0	0	0	0	0	0	2	2
9535	75	1	0	0	0	0	1	1	1	1	1	1	0	0	0	1	4
9539	70	1	1	0	0	0	1	1	0	1	1	1	0	1	0	2	4
9540	70	2	0	0	0	0	1	0	0	0	1	1	0	0	0	2	2
9543	72	2	0	1	0	1	1	1	0	0	0	1	0	0	0	3	4
9547	69	1	0	0	0	1	3	1	1	1	0	0	0	0	0	1	4

9548	65	2	1	0	0	1	1	#NULL!	#NULL!	#NULL!	1	0	#NULL!	0	0	3	4
9549	78	2	1	1	0	0	1	1	0	0	1	0	0	0	0	2	3
9550	81	2	0	0	1	#NULL!	1	1	0	0	1	0	0	0	0	4	1
9552	67	2	1	1	0	1	1	1	0	0	0	0	1	0	0	4	1
9555	74	1	0	0	0	1	1	0	0	0	#NULL!	#NULL!	0	0	1	2	4
9558	71	2	0	#NULL!	0	1	1	#NULL!	#NULL!	0	0	0	#NULL!	0	0	4	4
9560	77	2	1	0	0	1	1	1	0	0	0	0	0	0	0	2	4
9562	84	1	0	0	0	0	1	2	0	0	1	0	0	0	0	1	4
9565	77	1	0	0	1	1	3	1	0	1	1	0	0	0	0	1	4
9566	75	2	0	0	1	1	3	1	0	0	0	0	1	0	0	4	3
9575	67	1	0	0	0	0	1	1	0	0	0	0	1	0	0	2	4
9578	67	1	0	0	0	0	3	1	0	1	1	1	0	0	0	3	4
9579	66	2	0	0	0	0	1	1	0	1	0	1	0	0	0	2	3
9580	72	1	0	0	0	1	1	1	0	1	0	1	0	1	0	1	4
9581	66	2	0	0	0	0	3	1	0	0	0	0	0	0	0	2	2
9583	70	2	0	0	0	0	3	1	0	0	0	0	0	0	1	2	4
9584	65	1	0	0	0	1	3	0	0	1	0	0	1	0	0	2	4
9585	82	2	0	1	0	0	1	1	0	0	0	0	#NULL!	0	0	2	4
9589	72	1	0	0	1	1	1	0	0	1	1	1	0	0	0	2	4
9590	69	2	1	0	0	1	1	0	0	0	1	1	0	0	0	2	2
9591	71	1	1	0	1	#NULL!	3	1	#NULL!	1	0	0	0	0	0	3	4
9592	71	2	0	0	0	0	1	2	0	0	0	0	0	0	0	2	4
9593	70	2	0	0	0	0	1	1	1	1	0	0	1	1	1	1	4
9594	70	1	0	0	0	#NULL!	1	1	1	1	1	0	0	0	1	2	4
9597	81	1	0	0	0	1	1	#NULL!	#NULL!	#NULL!	1	0	#NULL!	0	0	2	4
9598	73	2	0	0	0	1	1	2	1	0	1	1	0	0	1	2	2
9601	74	1	0	0	0	1	1	0	1	1	1	1	0	0	1	3	4
9602	73	2	0	0	0	0	1	1	0	0	1	1	0	0	0	2	4
9609	75	1	0	0	0	1	3	2	1	1	0	1	0	0	0	2	4
9612	67	2	0	0	0	0	3	0	0	0	0	0	0	0	0	2	2
9618	73	1	0	0	0	0	1	0	0	1	1	1	0	0	0	1	4
9619	70	2	0	0	0	0	1	0	0	0	0	1	0	0	0	1	4
9621	77	2	0	0	0	0	1	#NULL!	#NULL!	#NULL!	0	0	#NULL!	0	0	2	1
9622	80	2	0	1	0	0	1	0	0	0	1	0	0	0	0	1	2
9625	70	2	0	0	0	#NULL!	1	0	0	0	0	1	0	0	0	2	4
9626	68	1	0	0	0	#NULL!	3	2	0	1	1	1	0	0	0	3	4
9628	77	2	0	0	0	0	3	0	0	0	0	0	1	0	0	2	2
9630	67	1	0	0	0	1	1	#NULL!	1	1	1	0	0	0	0	2	4
9635	69	2	0	1	0	1	1	0	0	0	0	0	0	0	1	2	4

9636	71	2	0	1	0	1	1	1	0	0	1	0	0	0	1	2	4
9642	67	1	0	0	1	1	1	1	1	1	1	0	0	0	0	4	1
9645	74	1	0	0	0	1	3	1	0	0	1	1	0	1	0	1	4
9647	74	1	0	0	0	0	1	1	1	1	0	1	0	0	0	1	4
9648	72	2	0	0	0	0	3	1	0	0	0	0	0	0	0	2	1
9652	72	2	1	0	1	1	1	0	0	1	0	0	1	1	0	2	1
9655	66	1	0	0	0	1	3	0	1	1	0	0	0	0	1	3	4
9660	72	2	0	0	0	0	1	2	0	0	0	0	0	0	0	2	1
9664	67	1	1	0	0	0	1	0	0	1	0	0	0	0	1	1	4
9665	91	2	1	1	0	0	1	1	0	0	0	0	1	1	0	4	1
9666	66	1	0	1	0	0	1	2	0	1	0	1	0	0	0	1	4
9670	82	2	1	0	1	0	1	1	0	0	0	0	0	0	0	2	4
9673	68	1	0	0	0	0	2	#NULL!	0	0	0	0	0	0	1	2	4
9675	65	1	0	0	0	#NULL!	2	0	0	1	0	1	0	0	1	2	4
9676	85	2	1	0	0	0	3	1	0	#NULL!	0	0	0	0	0	2	2
9677	84	1	0	0	0	0	3	1	1	1	1	0	0	0	0	1	4
9679	69	2	0	1	0	1	1	0	0	0	0	0	0	0	0	2	1
9683	73	2	0	0	0	0	3	1	0	0	1	1	0	0	1	2	2
9684	72	1	0	0	0	0	1	0	0	1	1	0	0	0	0	2	4
9685	71	1	0	#NULL!	0	#NULL!	3	#NULL!	#NULL!	#NULL!	1	1	#NULL!	0	1	2	4
9686	65	2	0	0	0	1	1	0	0	0	0	0	0	0	0	2	4
9688	76	2	0	0	1	1	1	2	0	0	1	0	0	0	0	1	4
9690	68	1	0	0	0	0	1	0	0	1	0	0	0	0	0	2	4
9693	67	1	0	1	0	1	1	0	1	1	1	0	0	0	0	2	4
9695	70	2	0	0	0	0	1	0	0	0	1	1	0	0	0	1	4
9696	78	2	0	1	1	1	1	1	0	0	1	0	0	0	0	2	3
9701	65	2	0	0	0	0	1	#NULL!	1	0	0	1	0	0	0	2	4
9702	68	1	0	0	0	1	1	0	1	0	1	1	0	0	0	1	4
9703	67	2	0	0	0	1	1	0	0	0	1	1	0	0	0	2	2
9704	66	2	0	1	#NULL!	1	1	0	0	0	0	0	0	0	0	2	4
9705	79	1	0	0	0	1	1	0	1	1	1	0	0	0	0	2	3
9706	68	2	0	0	1	1	3	0	1	1	0	0	0	0	0	2	2
9707	86	1	0	0	0	0	3	0	0	1	0	0	0	0	0	1	4
9709	75	2	1	1	1	1	3	1	0	0	0	0	0	0	0	3	4
9710	66	2	0	0	0	0	1	1	0	0	0	0	0	0	0	2	3
9711	65	1	0	0	0	0	1	0	0	0	0	0	0	0	1	1	4
9716	69	1	0	0	0	#NULL!	1	0	0	1	0	0	0	0	1	1	4
9717	67	2	0	0	0	#NULL!	3	0	0	0	0	0	0	0	1	2	4
9718	70	1	0	0	1	1	3	1	1	1	0	0	0	0	0	2	4

9722	77	1	0	0	0	1	1	1	0	0	0	0	0	0	1	1	4
9723	69	2	0	0	0	#NULL!	1	2	0	0	0	0	0	0	0	2	1
9725	72	2	0	0	1	0	1	1	0	0	0	0	0	0	0	4	1
9726	77	1	0	1	1	0	1	1	1	0	1	0	0	0	0	3	4
9728	66	2	0	0	0	1	1	1	0	0	0	1	0	0	0	2	3
9729	72	1	1	0	0	#NULL!	1	0	1	1	1	0	0	0	0	1	4
9730	68	2	0	0	0	1	1	0	0	0	1	0	1	0	0	2	2
9732	81	2	1	0	1	0	1	1	0	0	1	1	0	0	0	2	4
9733	72	2	0	0	0	1	1	2	0	0	0	0	0	0	0	1	2
9735	84	2	0	0	0	0	1	1	0	0	0	0	0	0	0	3	4
9736	84	1	1	0	0	1	3	1	0	0	0	0	1	0	0	4	1
9737	78	2	0	0	0	1	2	1	0	1	1	0	1	0	0	2	2
9739	73	2	0	0	0	#NULL!	3	1	0	#NULL!	0	0	0	0	0	2	2
9740	70	1	0	0	0	0	1	2	1	1	1	0	0	0	1	1	4
9741	66	1	0	0	0	0	2	0	1	0	1	0	0	0	0	1	4
9742	72	1	0	0	0	1	3	1	0	1	1	1	0	0	0	1	4
9743	69	1	0	0	0	0	2	1	1	1	0	0	0	0	1	1	4
9744	70	1	0	1	1	1	3	0	0	1	0	0	1	0	0	2	4
9745	70	2	0	0	0	0	1	0	0	0	0	1	0	0	0	2	3
9748	75	2	0	0	0	0	3	0	0	0	0	0	0	0	1	3	3
9749	65	1	0	0	0	0	1	1	1	1	0	0	0	0	1	1	4
9751	80	1	1	0	1	0	1	1	0	1	#NULL!	#NULL!	0	0	1	3	4
9752	77	2	0	0	1	0	1	2	0	0	1	1	0	0	1	3	1
9753	68	1	0	0	0	1	1	0	1	0	0	0	0	0	0	2	4
9754	66	1	0	0	0	0	1	1	0	0	0	0	0	0	0	2	4
9755	72	2	0	0	0	0	1	2	0	0	1	1	0	0	0	2	4
9759	72	2	0	0	0	1	2	1	0	0	1	1	0	0	0	4	1
9760	75	1	0	0	0	1	3	0	0	1	0	0	0	1	1	3	4
9761	72	2	0	0	0	1	1	0	0	0	0	0	0	0	0	4	1
9762	75	2	0	1	1	0	3	1	0	1	0	0	0	0	0	3	2
9763	80	1	1	1	0	1	1	1	0	1	0	0	0	1	0	1	4
9764	67	1	0	0	0	0	3	0	1	1	1	1	0	1	0	1	4
9765	66	2	0	0	0	0	1	0	0	0	1	1	0	0	1	1	3
9770	80	2	1	1	1	0	2	0	0	0	0	0	1	1	0	3	1
9772	80	1	0	0	0	0	3	#NULL!	0	0	1	1	0	0	0	1	4
9773	76	2	0	0	0	0	1	#NULL!	#NULL!	#NULL!	1	0	#NULL!	0	0	1	4
9774	73	1	0	0	0	1	1	2	0	1	0	0	1	0	0	2	4
9776	70	1	1	0	0	1	1	1	1	1	0	1	0	0	0	2	4
9777	65	2	0	1	0	0	1	0	0	0	0	1	0	0	1	2	2

9778	66	1	0	0	0	0	1	0	1	1	0	0	0	1	0	1	4
9780	81	2	0	1	1	0	1	1	0	0	1	0	0	0	0	3	4
9781	69	1	0	0	0	1	3	2	0	1	0	0	0	0	1	1	4
9783	67	2	0	0	0	#NULL!	1	0	0	0	1	0	0	0	1	3	2
9784	75	1	0	1	1	0	1	2	1	1	0	0	1	0	0	3	4
9786	66	1	0	1	0	1	1	0	1	0	0	0	0	0	0	2	4
9793	67	1	0	0	0	0	1	1	1	0	0	0	0	0	1	1	4
9795	71	1	0	0	0	0	2	0	0	0	0	0	1	0	0	4	4
9798	77	1	0	0	1	1	3	1	0	0	0	0	0	0	0	3	4
9800	68	1	0	0	0	1	1	2	0	0	0	0	0	0	1	3	4
9801	65	2	1	0	0	#NULL!	2	1	0	0	1	1	0	0	0	2	2
9804	88	2	1	1	0	#NULL!	1	#NULL!	0	1	1	0	0	0	0	1	2
9805	66	1	0	0	0	1	1	0	1	1	1	1	0	0	0	3	4
9810	69	2	1	1	1	1	1	2	0	1	0	0	1	0	0	2	4
9818	76	1	0	0	0	0	1	1	1	0	1	1	0	0	1	1	4
9819	75	2	0	0	0	0	1	1	1	#NULL!	0	0	0	0	1	2	4
9820	66	1	0	0	0	0	1	0	1	1	1	0	0	0	0	2	4
9823	65	1	0	0	1	1	1	0	0	0	0	0	1	0	1	4	4
9824	72	1	0	1	1	0	3	2	0	1	0	0	0	0	0	3	4
9825	76	1	1	0	1	1	1	0	0	1	0	0	1	1	0	3	4
9826	69	2	1	0	0	1	1	0	0	0	0	0	0	0	0	4	1
9827	67	1	0	0	1	1	1	1	0	1	0	0	1	0	1	3	4
9831	66	1	0	0	0	1	2	0	0	1	0	1	0	0	0	2	4
9836	69	1	0	1	0	0	1	1	0	0	1	0	0	0	0	3	2
9837	65	1	0	0	0	0	1	1	1	1	1	0	0	0	1	4	4
9840	73	1	0	0	1	0	#NULL!	0	0	0	1	1	0	0	0	2	4
9841	67	1	0	0	0	1	3	1	0	1	0	0	0	0	0	3	4
9843	77	1	0	0	0	1	1	0	1	1	0	1	0	0	0	1	4
9844	75	2	0	0	1	1	1	1	0	0	0	1	1	0	0	2	2
9845	72	2	0	0	1	1	#NULL!	0	0	0	0	0	1	0	0	4	3
9847	73	1	1	0	1	1	1	0	1	0	1	1	0	0	0	3	4
9848	70	2	1	0	1	1	1	2	1	#NULL!	0	0	0	0	0	1	2
9849	68	1	0	1	0	1	1	1	0	0	0	0	0	0	0	2	4
9850	69	2	0	0	0	1	1	2	1	0	0	0	1	0	0	1	4
9851	67	1	0	0	0	1	1	0	0	1	0	0	0	0	0	1	4
9852	73	1	0	0	0	1	1	1	0	1	0	1	0	1	0	2	4
9854	77	2	0	0	0	1	1	0	0	1	1	1	0	0	0	1	3
9860	71	1	0	0	0	1	3	0	0	1	0	0	0	0	0	1	4
9868	67	2	0	0	0	1	1	#NULL!	0	0	0	0	0	0	0	2	3

9872	66	1	0	0	0	0	1	0	0	0	0	0	0	0	1	1	4
9873	66	2	0	0	0	1	1	0	0	1	1	1	1	0	0	2	2
9878	77	1	0	0	0	1	1	0	0	1	0	0	0	0	0	2	4
9880	66	2	0	1	0	#NULL!	1	0	0	0	0	0	0	0	1	1	3
9882	67	1	0	0	0	0	3	#NULL!	#NULL!	#NULL!	0	0	#NULL!	0	1	4	4
9883	78	1	0	0	0	1	1	1	1	1	0	0	0	0	0	2	4
9884	77	2	0	0	0	1	2	1	#NULL!	#NULL!	0	0	0	0	0	2	4
9885	85	1	0	1	1	0	1	0	1	1	0	0	0	0	0	1	4
9887	70	2	0	0	0	1	2	0	0	0	0	0	0	0	0	2	2
9890	67	2	0	0	0	1	3	2	0	0	0	0	0	0	0	1	3
9893	76	1	0	0	1	0	1	2	1	1	0	0	0	0	0	3	4
9894	71	2	0	0	0	0	3	0	1	1	0	0	1	0	0	3	4
9896	73	2	0	0	1	1	3	1	0	0	0	0	0	0	0	3	4
9898	65	1	0	0	0	0	1	0	1	1	0	1	0	0	1	1	4
9902	83	1	1	0	1	0	1	1	0	1	0	0	0	0	0	3	4
9904	72	2	0	0	1	1	1	1	0	0	0	1	0	0	0	3	3
9906	72	2	0	0	1	1	3	#NULL!	#NULL!	#NULL!	0	0	#NULL!	0	0	3	4
9908	71	1	0	0	0	0	1	0	0	1	0	1	0	0	1	1	4
9911	82	2	0	0	0	0	1	0	1	0	0	1	0	0	1	2	2
9913	73	1	0	0	1	#NULL!	1	1	1	1	0	1	0	0	0	1	4
9916	70	2	0	1	1	0	3	1	0	0	1	0	0	0	1	3	3
9918	66	1	0	1	1	1	3	2	0	1	0	0	0	0	0	2	4
9920	65	2	0	0	0	1	1	0	0	1	1	0	1	0	1	2	3
9921	65	1	0	0	1	1	1	0	1	1	1	1	1	0	1	1	4
9922	76	1	1	0	0	1	2	0	0	0	0	1	1	1	1	2	4
9927	67	2	0	0	1	0	1	#NULL!	0	0	1	1	0	0	1	3	4
9932	83	1	0	0	0	0	2	1	0	0	0	0	0	0	0	1	4
9933	70	2	0	1	0	1	1	0	0	#NULL!	0	0	1	0	1	1	4
9934	75	1	0	0	0	0	1	0	1	1	0	1	0	0	1	2	4
9935	73	2	1	0	1	0	1	0	0	0	0	0	0	0	0	2	1
9940	78	1	0	0	0	#NULL!	1	1	0	1	0	0	1	0	0	1	4
9941	77	2	0	0	0	1	3	1	0	0	0	0	1	0	0	4	1
9942	75	1	0	0	0	1	1	0	0	0	0	1	0	0	0	1	4
9943	68	2	0	0	0	1	1	0	0	0	0	1	0	0	0	1	2
9944	65	2	0	0	0	1	1	0	1	0	0	0	0	0	1	2	3
9945	67	2	0	1	0	1	1	0	1	1	0	1	0	0	1	2	4
9948	67	2	0	0	1	1	1	0	0	0	0	0	0	0	1	3	3
9949	82	1	0	0	1	0	3	1	1	0	1	0	0	0	0	1	4
9950	82	2	0	0	0	0	1	0	#NULL!	#NULL!	0	0	0	0	0	2	4

9951	78	2	1	1	1	1	2	0	0	0	0	0	#NULL!	0	0	3	4
9955	72	1	1	0	1	1	1	0	1	1	1	1	0	1	1	3	4
9956	68	2	0	0	0	1	1	0	0	0	0	1	0	0	1	2	4
9962	79	1	1	1	1	1	#NULL!	1	0	1	0	0	0	0	0	1	1
9965	72	1	0	0	0	1	1	0	1	1	0	0	0	0	0	2	4
9966	69	2	0	0	0	#NULL!	1	1	0	0	0	0	0	0	0	2	2
9969	68	1	0	0	0	1	1	0	1	1	0	0	0	0	0	1	4
9970	66	2	0	0	0	1	1	0	0	0	0	0	#NULL!	0	1	3	3
9972	69	1	0	0	0	1	#NULL!	#NULL!	1	1	1	1	0	0	0	3	1
9973	65	2	0	0	0	1	3	1	0	0	1	0	0	0	0	3	1
9977	72	1	1	0	0	0	1	2	1	1	1	0	1	0	0	2	4
9978	71	2	1	0	0	1	1	1	0	0	0	1	0	0	0	2	2
9979	66	1	0	0	0	1	1	0	1	1	1	0	0	0	0	2	4
9980	66	2	0	0	0	1	1	0	1	0	1	0	0	0	0	2	4
9982	71	2	0	1	0	0	3	0	0	0	0	1	0	0	1	2	4
9983	70	1	0	0	0	1	1	1	0	1	0	0	0	1	0	2	4
9984	66	2	0	0	0	1	1	0	0	0	0	0	1	0	0	2	2
9985	71	1	0	1	0	0	1	0	1	1	1	0	0	0	0	1	4
9986	65	1	0	0	1	0	1	0	1	1	1	0	0	0	1	3	4
9989	74	2	0	1	0	0	1	1	0	1	1	1	0	0	0	2	4
9992	66	1	0	0	1	0	1	0	0	1	1	0	0	0	1	4	4
9995	75	2	0	1	1	1	3	1	0	0	0	0	1	0	1	4	2
9998	78	2	0	1	1	0	1	0	1	0	0	0	0	0	0	3	4
10001	68	2	1	0	1	1	3	1	0	0	0	0	0	0	0	3	3
10002	68	1	0	0	1	1	1	2	0	1	1	1	0	0	1	3	4
10003	71	1	0	0	0	1	1	2	1	1	0	0	1	0	0	1	4
10004	70	2	0	0	0	1	1	0	0	0	0	0	0	0	0	2	2
10007	70	2	0	0	1	1	1	0	0	0	0	0	0	0	0	2	3
10008	84	2	0	0	1	1	3	1	1	0	0	0	0	0	0	1	3
10011	65	2	0	1	1	1	3	2	1	0	0	0	0	0	0	2	2
10013	72	2	0	1	1	0	1	1	0	0	0	0	1	0	0	3	4
10015	68	2	0	1	1	1	1	0	1	0	0	0	0	0	1	2	4
10016	71	2	0	1	0	0	1	#NULL!	0	0	1	1	0	0	0	3	3
10017	71	1	0	0	0	1	1	0	0	1	1	0	0	0	0	1	4
10022	76	1	0	0	0	#NULL!	#NULL!	1	0	1	1	0	0	0	1	1	4
10023	75	2	0	0	0	0	1	1	0	0	1	1	0	0	0	4	1
10027	76	1	1	0	1	1	2	0	0	1	0	0	0	0	0	2	4
10029	68	2	0	1	1	1	3	#NULL!	#NULL!	#NULL!	0	0	#NULL!	0	0	3	4
10030	66	1	0	0	0	1	1	#NULL!	#NULL!	#NULL!	1	0	#NULL!	0	0	2	4

10033	72	1	0	0	0	1	1	1	0	1	0	0	0	0	1	3	4
10034	75	2	1	1	0	0	1	0	0	0	1	1	0	0	0	2	4
10035	73	1	0	0	1	1	1	0	0	1	1	0	0	1	0	3	4
10036	72	2	1	0	0	1	1	#NULL!	0	0	1	1	0	0	0	1	3
10037	68	1	0	0	0	1	2	1	0	1	0	0	0	1	0	1	4
10038	65	1	0	0	0	1	3	1	0	0	0	0	0	0	0	3	4
10045	66	2	0	1	0	1	1	0	0	0	0	0	0	0	1	4	1
10053	80	2	0	0	0	0	1	0	0	0	0	0	0	1	0	2	4
10054	82	1	0	0	0	1	3	2	1	0	0	0	1	1	0	3	4
10055	81	2	0	0	0	0	1	1	0	0	0	1	0	0	0	3	4
10058	86	1	1	1	1	0	#NULL!	1	0	0	0	0	0	0	0	4	1
10062	99	1	0	1	0	1	2	#NULL!	#NULL!	#NULL!	1	0	#NULL!	1	0	1	4
10066	66	2	1	1	1	1	1	0	1	1	0	0	0	0	0	2	4
10067	76	2	0	1	1	1	3	1	0	0	0	1	0	0	0	3	1
10068	70	1	0	0	1	1	#NULL!	#NULL!	#NULL!	#NULL!	0	0	#NULL!	1	1	3	4
10069	66	2	0	0	1	0	1	0	0	0	0	0	0	0	0	4	1
10070	74	1	0	0	0	1	2	2	0	0	1	0	0	1	0	1	4
10073	80	1	0	0	1	1	1	#NULL!	#NULL!	#NULL!	1	1	#NULL!	0	1	3	3
10078	84	2	1	1	0	1	1	0	0	0	0	1	0	0	0	2	4
10079	66	2	0	1	1	1	3	1	0	0	1	0	0	0	0	4	1
10081	67	2	0	0	0	1	1	0	0	#NULL!	0	1	0	0	0	2	4
10083	69	2	0	0	1	1	3	0	0	0	0	0	0	0	0	1	4
10084	75	2	0	1	0	0	1	0	0	0	1	1	0	0	0	2	4
10085	70	2	0	1	0	0	1	1	0	0	1	1	0	0	0	4	1
10086	71	1	1	0	1	0	1	1	1	1	0	1	0	0	1	2	1
10087	66	2	0	0	0	0	1	0	0	0	0	1	0	0	0	2	2
10088	70	1	0	0	0	0	3	1	1	1	1	0	0	0	0	2	4
10089	65	2	0	0	0	0	1	0	0	0	1	0	0	0	0	2	2
10092	70	2	0	1	0	1	1	0	0	0	0	1	0	0	0	2	2
10093	69	2	0	1	0	1	1	0	0	0	1	1	1	0	0	2	4
10094	86	1	0	0	0	#NULL!	1	0	1	1	1	0	0	0	0	1	4
10095	75	2	0	1	1	0	1	1	0	0	0	0	1	0	1	3	3
10097	85	1	0	0	0	0	2	1	0	1	0	0	0	0	0	1	4
10098	77	2	0	0	0	0	1	0	1	0	0	0	0	0	0	2	1
10102	71	2	1	0	1	1	1	1	0	0	0	0	0	1	0	3	3
10103	74	1	1	1	0	1	1	1	0	0	0	0	0	0	0	3	4
10105	77	1	1	0	0	1	1	1	1	0	0	0	1	1	0	3	4
10108	73	2	0	0	0	0	3	1	0	1	0	0	0	0	1	2	3
10113	65	1	0	0	0	#NULL!	1	2	0	1	0	0	0	0	0	2	4

10114	68	1	1	0	0	0	1	1	1	1	1	1	0	0	1	1	4
10115	67	2	0	0	0	1	1	1	0	0	1	1	0	0	0	2	3
10116	76	1	1	0	1	1	3	2	1	1	0	0	1	1	0	3	4
10120	83	1	0	0	0	1	1	1	1	1	0	0	0	1	0	1	4
10121	80	2	0	0	0	1	1	1	0	0	1	0	0	0	0	2	2
10123	70	1	0	0	0	1	1	0	1	1	0	1	0	0	0	1	4
10126	68	1	0	0	0	1	1	0	0	1	0	0	0	0	0	1	4
10128	67	2	0	0	0	#NULL!	3	1	0	0	0	0	1	0	0	1	2
10129	66	1	0	1	0	1	1	0	1	0	1	1	0	0	0	1	4
10130	73	2	0	0	0	1	1	#NULL!	#NULL!	0	0	0	0	0	0	2	2
10131	72	1	1	0	0	1	3	2	0	0	1	0	1	1	0	1	4
10132	65	1	0	0	0	0	3	1	1	1	0	0	0	0	0	1	4
10134	73	2	0	0	1	1	1	1	0	1	1	0	0	0	0	3	4
10135	65	1	0	1	0	1	3	1	1	1	1	0	1	0	0	3	4
10137	68	1	0	0	0	1	1	0	1	1	1	0	0	0	0	1	4
10139	68	1	0	0	0	0	2	0	0	0	0	0	0	0	1	1	4
10140	65	2	0	0	0	1	1	0	0	0	1	0	0	0	0	2	2
10141	75	1	0	0	0	#NULL!	1	0	1	1	1	1	0	0	0	2	4
10142	74	2	0	0	0	1	3	1	0	0	1	0	0	0	0	3	2
10144	81	2	0	0	0	1	3	1	0	0	1	0	0	0	0	2	2
10148	67	1	0	0	0	1	1	1	1	0	0	0	0	0	1	2	4
10150	74	2	0	1	0	1	1	1	0	0	0	0	0	0	1	1	4
10151	73	1	0	0	0	1	1	1	1	0	1	1	0	0	0	3	4
10155	70	2	0	0	#NULL!	#NULL!	1	0	0	0	1	0	0	0	0	2	3
10156	75	2	0	1	0	1	1	1	0	0	0	0	0	0	0	2	4
10157	67	1	0	0	0	1	1	2	1	1	0	0	0	0	0	1	3
10158	65	2	0	0	0	1	1	0	0	0	0	1	0	0	1	2	4
10159	70	2	0	0	0	1	2	0	1	0	0	0	0	0	0	2	4
10164	67	1	0	0	0	0	3	1	1	1	0	0	0	0	0	1	4
10167	71	1	0	1	0	1	3	0	0	1	0	0	0	0	0	1	4
10169	67	1	0	0	0	0	1	1	0	0	1	1	0	1	1	1	4
10170	65	2	0	0	0	0	1	0	0	0	0	1	0	0	1	4	4
10174	70	1	0	0	0	1	1	1	1	1	0	1	0	0	0	3	4
10176	74	1	0	0	0	1	1	1	1	0	1	1	0	1	0	1	4
10178	74	2	0	1	0	1	1	0	1	0	0	0	0	0	0	1	1
10179	67	2	0	1	0	1	1	0	0	0	0	0	0	0	1	1	4
10182	81	1	0	0	1	1	1	2	0	1	0	0	0	0	0	2	4
10183	78	2	1	0	1	1	1	0	0	0	1	0	0	0	0	2	1
10185	69	2	0	1	0	1	1	0	0	0	1	0	1	0	0	3	3

10186	69	1	0	0	1	1	1	1	0	1	0	0	0	0	0	2	4
10187	75	1	0	1	1	#NULL!	1	0	0	1	1	1	0	0	0	2	4
10190	66	2	0	1	0	1	1	0	0	0	0	1	0	0	0	1	4
10191	71	1	0	0	1	1	3	1	0	0	0	0	1	0	0	2	4
10192	66	2	0	0	0	1	1	0	0	0	0	0	0	0	1	2	4
10193	68	2	0	1	0	0	2	1	1	0	0	0	1	0	1	1	4
10195	66	1	0	0	0	0	1	1	0	1	0	0	0	0	1	1	4
10196	66	1	0	0	0	1	2	0	0	1	0	1	0	1	0	4	1
10197	65	2	0	0	0	1	1	0	0	0	1	0	0	0	0	2	3
10198	69	1	0	0	0	1	1	0	0	0	1	0	0	0	0	1	4
10199	75	1	0	0	0	1	1	1	1	1	1	0	1	1	0	2	4
10203	66	2	0	0	0	1	1	0	0	0	0	0	0	0	0	2	4
10205	65	1	0	0	0	1	1	0	1	0	0	1	0	0	1	1	4
10212	82	2	0	1	0	1	3	1	0	0	0	0	1	0	0	1	4
10213	76	1	0	0	0	0	1	0	0	1	0	0	0	0	0	3	4
10214	72	2	0	0	0	#NULL!	3	1	0	0	0	0	0	0	0	2	2
10217	77	1	0	0	0	1	1	0	1	0	0	0	0	1	0	3	1
10218	82	1	0	0	0	1	1	1	1	0	0	0	1	0	0	2	4
10219	79	2	0	0	0	1	1	1	0	0	0	0	0	0	0	2	2
10221	66	1	0	0	0	1	1	1	0	1	0	0	0	0	0	2	4
10222	81	1	1	0	1	1	1	0	1	1	0	0	1	0	0	3	4
10226	70	2	0	1	0	0	3	2	0	0	0	0	0	0	0	2	3
10228	65	2	0	0	0	1	1	0	0	0	1	1	0	0	0	2	2
10229	66	1	0	0	1	1	1	0	1	0	0	0	0	0	0	3	4
10230	69	1	0	0	1	1	1	1	1	1	0	0	0	0	0	1	4
10231	68	2	0	0	0	1	1	0	0	0	0	0	0	0	0	2	3
10232	81	2	0	1	1	0	1	1	0	0	1	0	0	0	0	4	1
10234	78	1	0	0	0	1	1	#NULL!	0	1	0	0	0	0	0	3	4
10235	74	2	0	0	0	1	3	#NULL!	0	0	0	0	0	0	0	2	4
10236	74	1	0	1	0	0	1	1	1	1	1	0	0	0	1	2	4
10237	76	1	0	1	0	0	1	1	1	0	0	0	0	0	1	4	4
10240	69	2	0	0	0	1	1	#NULL!	#NULL!	#NULL!	0	1	#NULL!	0	0	2	2
10242	83	2	0	1	0	1	1	#NULL!	0	0	0	0	0	0	0	2	2
10243	74	1	0	0	0	1	1	0	0	1	0	0	0	0	0	2	4
10244	69	2	1	0	0	1	1	0	0	0	1	0	0	0	1	2	4
10246	73	1	0	0	0	1	1	1	0	0	0	0	0	0	1	2	4
10247	70	2	0	0	0	0	1	1	0	0	0	0	0	0	0	2	3
10252	87	2	0	1	0	0	1	1	0	0	0	0	0	0	0	2	3
10253	68	1	0	1	0	1	1	0	1	1	0	0	0	0	1	4	4

10257	80	2	0	1	1	1	3	1	0	0	0	0	0	0	0	3	2
10259	72	2	1	0	1	1	1	0	0	0	0	1	#NULL!	0	0	4	3
10262	72	2	0	0	0	1	1	#NULL!	0	0	1	0	0	0	0	2	3
10263	73	2	0	1	0	1	1	0	0	0	1	0	0	0	0	2	4
10264	79	1	1	0	1	#NULL!	1	0	1	1	1	0	0	0	1	1	4
10265	76	2	0	0	0	0	2	1	0	0	1	0	0	0	0	1	4
10270	69	1	0	0	0	0	1	0	0	1	0	0	0	1	1	1	4
10271	77	1	0	0	0	0	1	1	1	1	0	0	0	0	1	1	4
10278	65	1	0	0	0	1	1	0	1	1	0	0	0	0	1	1	4
10279	70	1	0	0	0	0	1	#NULL!	#NULL!	#NULL!	0	1	#NULL!	0	0	1	4
10281	74	2	0	0	0	1	1	0	0	0	1	0	0	0	0	3	2
10288	68	1	0	0	0	1	1	#NULL!	#NULL!	#NULL!	1	0	#NULL!	0	0	2	4
10291	82	2	1	0	0	0	1	0	0	0	1	1	0	0	1	1	4
10294	76	2	0	1	1	1	1	0	0	0	0	0	0	0	0	3	3
10295	75	2	0	1	1	0	1	#NULL!	0	0	0	0	0	0	0	4	1
10296	76	1	0	1	0	0	1	1	0	0	0	0	0	0	0	2	4
10302	68	2	0	0	0	1	1	0	0	0	0	0	0	0	0	1	4
10303	74	1	0	0	0	0	1	1	0	1	0	0	0	0	0	2	4
10304	73	2	0	0	0	1	3	1	0	0	1	0	1	0	0	4	1
10308	77	2	0	0	0	#NULL!	1	#NULL!	0	0	1	1	0	0	0	2	3
10315	67	1	0	0	0	#NULL!	1	2	1	1	0	0	0	0	1	2	4
10316	66	2	0	0	0	0	1	2	0	0	0	0	0	0	1	1	2
10318	72	1	0	0	0	0	1	1	0	1	1	0	0	0	1	3	1
10319	67	2	0	0	1	1	2	1	0	0	1	0	0	0	0	2	1
10321	68	1	0	0	0	1	3	0	1	1	1	0	0	0	0	1	4
10334	65	2	0	1	0	1	1	0	0	0	0	1	0	0	0	1	2
10337	84	2	0	0	0	1	1	1	0	0	1	1	1	0	0	2	1
10338	72	1	0	0	0	1	3	2	0	1	0	0	0	0	1	1	4
10343	78	2	1	0	1	1	1	1	0	0	0	0	1	0	0	3	2
10349	71	2	1	0	1	0	1	1	0	0	1	0	0	0	0	4	2
10350	66	1	0	1	0	0	1	0	0	1	1	0	0	0	1	1	4
10352	66	1	0	0	0	0	1	0	1	1	0	0	0	0	1	2	4
10357	68	2	0	1	0	#NULL!	1	0	0	0	1	1	0	0	0	3	3
10360	66	1	1	0	1	1	1	1	1	1	1	1	0	1	0	3	4
10363	76	2	0	0	0	1	1	1	0	0	1	1	0	0	0	2	4
10364	70	2	1	1	1	1	1	0	0	0	0	0	1	0	0	3	4
10365	71	2	0	0	1	0	1	1	0	0	0	0	0	0	0	2	3
10366	70	1	0	0	1	1	1	0	0	1	0	1	0	1	0	3	4
10372	67	1	0	0	1	1	1	2	1	0	0	0	0	0	1	3	4

10374	70	1	0	0	1	1	1	2	1	1	1	0	0	0	0	2	4
10376	76	1	0	0	0	1	2	0	0	0	1	1	0	0	0	2	4
10378	74	2	0	1	1	0	3	1	0	0	0	1	0	0	0	3	2
10380	78	1	0	0	1	0	1	0	1	1	0	0	0	0	0	3	4
10381	80	2	0	0	0	0	1	0	0	0	1	1	0	0	1	1	4
10382	79	1	0	0	0	0	1	1	1	1	1	1	0	0	0	1	4
10384	67	2	0	#NULL!	#NULL!	1	#NULL!	1	0	0	0	0	0	0	0	4	1
10388	73	1	0	1	0	0	1	0	0	1	0	0	0	0	0	3	4
10390	72	1	0	0	0	0	1	2	1	1	1	1	0	0	0	1	4
10391	71	2	0	0	0	0	3	1	0	0	0	1	0	0	0	2	2
10392	69	2	0	0	0	1	1	0	0	0	1	0	0	0	0	2	4
10393	66	1	0	0	0	1	2	0	0	0	0	0	0	0	0	2	4
10397	78	2	0	1	0	0	1	0	0	0	0	1	0	0	0	2	2
10398	71	1	0	0	0	0	3	1	0	1	0	0	1	0	0	1	4
10399	68	2	0	0	0	1	1	#NULL!	#NULL!	#NULL!	1	1	#NULL!	0	0	2	2
10403	76	2	1	0	1	0	2	1	0	0	0	1	1	0	0	1	4
10407	72	2	0	0	1	#NULL!	1	0	0	0	0	0	0	0	0	4	1
10409	80	1	0	0	1	1	1	#NULL!	1	1	0	0	0	0	0	3	4
10412	86	2	1	#NULL!	0	#NULL!	#NULL!	0	1	#NULL!	0	0	#NULL!	0	0	2	4
10413	81	2	1	1	1	0	1	1	0	0	0	1	1	0	0	2	2
10415	83	2	0	1	0	0	1	1	0	0	0	0	0	0	0	4	1
10424	83	2	1	1	0	0	1	0	0	0	1	1	0	0	0	2	4
10425	91	2	0	1	1	0	1	1	0	0	0	1	0	1	0	2	4
10428	74	2	0	1	0	0	1	0	0	0	0	1	0	0	0	1	3
10429	69	1	0	0	0	1	3	0	0	0	0	0	0	0	0	1	4
10430	65	2	0	0	0	1	2	#NULL!	0	0	0	0	0	0	0	2	4
10434	76	2	0	1	0	1	1	0	0	0	1	0	0	0	0	1	2
10438	66	2	0	0	0	#NULL!	1	1	0	0	0	1	0	0	1	2	4
10439	87	2	1	1	1	0	1	0	0	0	0	0	1	0	0	2	4
10443	66	1	0	0	0	1	1	2	0	1	0	1	0	1	0	1	4
10444	69	1	1	1	0	1	2	2	1	1	0	0	0	0	1	3	4
10446	71	2	0	1	0	1	1	1	0	0	0	0	1	0	0	2	2
10447	65	1	0	0	0	1	1	0	0	1	1	0	0	0	0	2	4
10448	69	1	0	0	0	1	1	1	1	1	0	1	0	0	0	3	4
10450	74	2	0	0	1	1	1	0	0	0	0	0	0	0	0	2	4
10451	71	1	0	0	0	1	1	0	0	1	0	0	0	0	1	2	4
10454	83	2	0	1	0	0	1	1	0	0	0	1	0	0	0	2	2
10456	74	2	0	0	0	1	1	0	0	0	0	1	0	0	0	4	1
10458	70	2	0	0	0	1	3	1	0	0	1	1	0	0	0	3	2

10459	84	2	0	1	0	#NULL!	#NULL!	0	0	#NULL!	0	0	1	0	0	4	1
10460	70	2	0	1	0	0	1	0	0	0	1	1	0	0	0	4	1
10462	70	2	1	0	0	0	1	0	0	0	1	0	0	0	0	2	2
10463	79	2	0	1	1	1	1	1	0	0	1	1	0	0	0	2	4
10468	70	2	1	0	1	1	3	#NULL!	0	0	0	0	0	0	0	1	4
10469	66	1	1	0	1	1	1	1	0	1	0	0	0	0	0	3	4
10470	66	2	0	0	1	0	2	0	0	0	0	0	0	0	1	4	1
10473	67	2	0	1	0	0	1	0	1	0	1	1	0	0	0	2	3
10477	69	1	0	0	1	1	1	1	0	1	0	0	1	0	1	3	4
10478	67	2	0	0	1	1	1	0	0	0	0	0	1	0	1	3	4
10480	79	2	0	1	1	#NULL!	1	0	0	0	0	0	1	1	0	3	4
10482	69	1	0	0	0	1	1	0	1	1	1	0	0	0	0	1	4
10483	66	2	0	0	0	1	3	2	0	0	0	0	1	0	0	2	2
10484	67	1	0	1	0	0	1	0	0	1	0	0	0	0	0	3	4
10485	65	2	0	1	0	1	1	0	0	0	0	0	0	0	0	2	2
10486	74	1	0	0	0	1	1	1	1	1	0	0	0	0	1	2	4
10489	69	2	0	0	0	1	1	1	0	0	0	0	0	0	0	2	2
10491	74	2	0	1	0	0	1	#NULL!	0	0	0	0	0	0	0	3	2
10493	72	2	0	0	0	1	1	2	0	0	1	0	0	0	0	2	3
10500	74	1	0	0	1	0	3	1	1	1	1	0	0	0	0	3	4
10502	71	1	0	#NULL!	0	#NULL!	1	#NULL!	#NULL!	#NULL!	0	0	#NULL!	0	1	2	4
10503	71	2	0	0	1	0	1	0	0	0	0	0	0	0	0	3	3
10504	70	1	0	0	1	1	3	#NULL!	#NULL!	#NULL!	0	0	#NULL!	0	0	1	4
10505	78	1	0	0	0	0	1	1	0	1	0	0	0	0	0	3	4
10506	68	2	0	0	0	1	3	1	0	0	0	0	0	0	0	3	2
10509	76	1	0	0	0	1	1	0	1	0	0	0	0	0	0	2	4
10512	65	1	0	0	0	0	3	2	1	1	0	0	0	0	0	1	4
10513	70	2	0	1	0	1	1	0	0	0	0	0	0	0	0	2	2
10514	87	2	1	1	0	0	1	#NULL!	0	0	0	0	0	0	0	4	1
10515	74	2	0	1	0	0	1	0	0	0	0	0	0	0	0	2	2
10517	78	2	0	0	0	1	1	1	0	0	0	0	0	0	0	1	1
10519	79	2	0	1	1	0	1	1	1	0	0	0	0	1	0	2	4
10524	65	1	0	0	0	1	1	1	0	1	0	0	0	0	0	2	4
10525	66	2	0	0	0	1	1	0	0	0	1	1	0	0	0	1	4
10526	65	1	0	0	0	#NULL!	1	0	0	1	1	1	0	0	1	1	4
10527	72	1	1	0	0	0	1	0	1	1	0	0	1	1	0	2	4
10534	66	1	0	0	1	1	1	0	1	1	0	0	0	0	0	2	4
10536	70	2	0	0	0	1	1	1	0	0	0	0	0	0	1	2	4
10538	70	2	0	0	0	1	1	0	0	0	0	0	0	0	0	2	2

10540	73	1	0	0	0	1	1	2	0	1	0	0	1	0	0	1	4
10543	65	2	0	1	0	1	1	#NULL!	0	0	0	0	1	0	1	3	4
10544	69	1	1	0	1	0	1	1	0	0	1	0	0	0	1	3	4
10545	66	2	0	0	0	0	1	0	0	0	0	0	0	0	0	3	4
10548	69	1	0	0	0	0	1	1	0	0	1	0	0	0	1	1	4
10551	71	2	0	1	1	1	1	1	0	0	0	0	0	0	0	3	4
10552	77	2	0	0	0	1	1	0	0	0	1	0	1	0	0	3	2
10553	76	1	1	0	1	1	1	1	1	1	1	0	0	0	0	3	4
10558	69	1	0	0	0	#NULL!	1	2	0	1	1	0	0	1	0	3	4
10560	66	2	0	0	0	0	1	#NULL!	#NULL!	#NULL!	1	1	#NULL!	0	0	3	2
10575	70	1	0	0	0	0	3	1	1	1	0	0	0	0	1	2	4
10576	67	2	0	0	0	0	1	2	0	0	0	0	0	1	0	2	2
10577	86	1	1	0	0	0	3	2	0	1	0	0	1	1	0	3	4
10578	84	2	1	0	0	#NULL!	1	1	0	0	1	0	#NULL!	0	0	2	2
10583	69	1	0	0	1	1	#NULL!	#NULL!	1	1	0	0	0	1	0	3	4
10584	69	2	0	0	1	1	3	0	0	0	0	0	1	0	0	1	1
10589	74	1	0	0	0	1	1	#NULL!	1	1	0	0	0	0	0	1	4
10590	69	2	0	0	0	1	1	0	0	0	0	1	0	0	0	2	3
10592	67	1	0	0	0	0	1	0	1	1	1	0	0	0	0	2	4
10593	65	1	0	0	0	1	1	1	0	1	0	0	0	0	0	2	4
10595	76	1	0	0	1	1	1	0	1	1	0	0	0	0	0	2	4
10596	72	2	0	0	1	1	1	0	0	0	1	0	0	0	0	2	1
10603	75	2	1	0	0	0	#NULL!	1	0	0	0	0	1	0	0	3	2
10607	71	1	0	0	1	0	1	1	1	1	0	1	0	0	1	3	4
10608	67	2	0	0	0	0	1	1	0	0	0	1	0	0	0	3	1
10611	66	1	0	0	0	0	1	0	1	1	0	0	0	1	0	1	4
10613	68	2	0	1	0	0	1	0	0	0	0	0	0	0	1	1	4
10617	81	2	0	1	0	0	1	0	0	0	0	0	0	0	0	4	1
10618	67	1	0	0	0	0	1	0	1	1	1	1	0	1	1	1	4
10619	66	2	0	0	0	0	1	0	0	0	0	0	0	0	0	2	4
10621	76	1	0	0	0	0	1	1	0	0	1	0	0	0	0	1	4
10622	70	2	0	0	0	0	1	0	0	0	0	0	0	0	0	2	2
10624	83	1	0	0	0	1	3	#NULL!	0	1	0	0	1	0	0	2	4
10627	74	2	0	0	1	1	1	0	0	1	0	0	0	0	0	2	4
10630	75	2	0	0	0	1	2	0	0	0	0	0	0	0	0	2	2
10633	72	2	0	0	0	0	1	0	0	0	0	0	0	0	0	2	3
10638	81	2	0	#NULL!	1	#NULL!	1	#NULL!	#NULL!	#NULL!	1	0	#NULL!	0	0	4	1
10641	76	2	0	#NULL!	0	#NULL!	1	1	0	0	1	0	0	0	0	1	4
10642	77	1	0	0	0	1	1	0	0	0	0	1	0	0	0	3	4

10643	75	2	1	0	0	1	1	0	0	0	0	0	1	0	0	3	2
10646	67	1	0	0	1	1	1	0	1	1	0	0	0	1	1	1	4
10649	66	2	0	0	0	#NULL!	1	1	0	0	0	0	1	0	1	4	2
10650	65	1	0	0	0	1	1	0	1	1	0	0	0	0	1	2	4
10651	71	1	0	1	0	#NULL!	1	0	0	0	0	1	0	0	0	1	4
10655	74	1	0	0	0	0	1	0	1	1	1	1	1	1	1	1	4
10656	73	2	1	0	0	0	1	1	0	0	1	1	0	0	1	4	1
10657	70	1	0	1	0	0	1	0	0	0	0	0	0	0	0	1	4
10658	79	2	0	0	1	0	3	0	0	0	0	0	0	0	0	4	1
10659	75	1	1	0	0	0	1	2	0	1	0	0	0	0	0	4	4
10662	81	2	0	1	0	#NULL!	1	0	0	0	0	0	1	0	0	2	1
10665	67	1	0	0	0	1	3	0	1	1	0	0	0	1	0	2	4
10666	65	2	0	0	0	1	1	2	0	1	0	0	0	0	0	3	2
10672	70	1	0	0	0	1	1	1	0	0	1	1	0	1	0	1	4
10673	67	2	0	0	0	1	2	1	0	0	0	0	0	0	0	2	1
10674	65	2	0	1	0	1	3	1	0	1	1	1	0	0	0	2	3
10675	70	1	0	0	0	1	3	1	0	0	0	1	1	0	0	1	4
10676	76	2	0	1	1	1	1	1	0	0	0	0	0	0	1	3	4
10677	73	2	0	1	1	1	1	1	0	0	0	0	1	0	0	3	4
10680	66	1	0	0	0	0	2	0	0	1	1	0	1	1	1	3	4
10682	71	2	0	1	0	0	1	0	0	0	0	0	0	0	0	2	2
10683	68	1	0	0	0	0	1	1	1	1	0	0	0	0	1	3	4
10685	77	2	0	1	1	1	1	1	0	0	0	0	1	0	0	4	1
10686	67	1	1	0	0	1	1	1	0	1	0	0	0	0	0	2	4
10687	79	2	0	1	0	1	1	1	0	1	0	0	0	0	0	3	2
10688	67	1	0	0	1	0	3	1	1	1	0	0	0	0	1	3	4
10689	66	1	0	0	1	1	1	1	0	1	1	0	0	0	0	2	4
10690	65	2	1	0	0	0	1	1	0	0	1	0	0	0	1	4	4
10697	72	2	1	0	0	1	1	0	0	0	0	0	0	0	0	3	1
10702	69	1	0	0	0	1	1	0	0	1	0	0	0	0	1	2	4
10703	65	2	0	0	0	0	2	0	0	0	0	1	0	0	0	2	2
10705	74	1	0	0	0	1	1	1	1	1	1	1	0	0	0	1	4
10706	69	2	0	0	0	1	1	0	0	0	0	0	1	0	0	2	1
10708	71	2	0	1	0	0	1	1	0	0	1	0	0	0	0	1	4
10710	67	2	0	0	0	1	3	0	0	0	0	1	0	0	0	2	1
10711	68	1	0	0	0	0	1	1	1	1	0	0	0	0	0	2	4
10713	80	2	1	1	0	1	2	#NULL!	#NULL!	#NULL!	0	1	#NULL!	0	0	3	4
10717	75	2	0	0	0	1	#NULL!	0	0	0	0	1	0	0	0	2	4
10719	77	1	0	0	0	0	1	2	1	0	1	0	0	0	0	1	4

10720	75	2	0	0	0	0	1	0	0	0	1	0	0	0	0	2	4
10723	75	2	0	0	0	0	1	0	0	0	0	0	0	0	1	2	4
10725	77	1	0	0	1	1	3	1	0	1	0	0	0	0	1	3	4
10726	76	2	0	0	1	1	1	1	0	0	1	1	1	0	0	2	2
10727	81	1	0	0	1	1	1	2	0	0	0	0	1	0	0	3	4
10728	75	2	0	0	0	1	1	#NULL!	1	0	1	1	0	0	0	2	2
10729	73	1	0	1	1	1	3	1	0	1	0	0	0	0	0	3	4
10730	66	2	1	0	0	1	3	0	0	0	1	1	0	0	0	1	4
10731	66	1	0	0	0	1	1	1	0	1	1	1	0	0	0	1	4
10733	66	2	0	1	0	1	1	0	0	0	0	0	0	0	0	3	2
10735	73	1	0	0	0	1	2	0	0	1	0	0	0	1	0	1	4
10736	67	2	1	0	0	1	1	0	0	1	0	0	0	0	0	2	2
10739	70	2	0	1	0	0	3	1	0	0	0	0	1	0	1	2	4
10743	76	2	0	1	0	0	1	#NULL!	0	0	0	0	0	0	0	2	4
10750	71	2	0	1	1	1	1	1	0	1	1	1	0	0	0	4	1
10752	90	1	0	0	0	1	2	0	0	1	0	0	0	0	0	2	4
10753	85	2	0	0	0	1	2	0	0	0	0	0	0	0	0	4	4
10757	72	2	0	0	0	1	3	1	0	0	0	0	1	0	0	2	3
10758	70	1	1	0	0	1	3	0	1	1	0	0	0	0	1	1	4
10759	69	2	0	0	0	1	1	0	0	0	0	0	0	0	0	2	4
10761	74	1	0	1	0	0	3	0	0	1	0	0	0	0	0	1	4
10762	74	2	0	1	1	#NULL!	1	#NULL!	0	1	0	0	0	0	0	3	2
10764	66	2	0	1	1	1	1	2	0	0	0	0	1	0	0	1	3
10766	74	2	1	0	1	1	1	0	0	0	1	0	0	0	0	3	1
10768	66	1	0	0	0	1	1	0	1	1	1	1	0	0	0	1	4
10769	66	2	0	0	0	1	1	1	0	0	0	0	1	0	0	2	3
10772	70	1	0	0	0	1	1	2	1	1	1	1	0	0	1	1	4
10773	71	1	0	0	0	0	1	1	1	1	1	0	0	0	1	1	4
10774	67	2	0	0	0	0	2	0	0	0	0	0	0	0	0	4	1
10776	67	2	0	0	0	0	2	2	0	0	1	0	0	0	0	3	3
10777	70	1	0	1	0	0	1	2	0	0	0	0	0	0	0	1	4
10778	70	1	0	0	0	0	3	0	0	1	1	1	0	0	0	1	4
10779	69	1	1	0	0	1	1	0	0	1	1	0	1	0	0	3	4
10781	76	2	0	0	1	#NULL!	3	2	1	1	0	0	0	0	0	3	4
10782	68	2	0	0	0	1	1	0	0	0	0	0	0	0	0	2	4
10785	69	1	0	0	0	0	1	0	#NULL!	#NULL!	0	0	#NULL!	0	1	1	4
10789	75	1	1	0	0	1	3	0	0	1	1	1	1	0	0	1	4
10790	68	2	0	0	0	1	3	1	0	0	0	0	1	0	0	2	4
10791	71	1	0	0	0	1	3	1	1	1	1	0	0	0	0	2	4

10792	71	2	0	0	0	1	1	0	0	0	0	0	0	0	0	2	3
10794	83	2	0	1	0	0	1	0	0	0	0	0	0	0	0	1	3
10795	73	1	1	0	1	1	1	0	1	1	1	0	0	1	0	4	4
10798	72	2	0	1	0	0	1	1	0	0	0	0	0	0	0	2	1
10799	73	1	0	0	0	0	3	1	0	1	0	1	0	0	0	1	4
10800	68	2	1	0	0	0	3	1	0	0	0	0	0	1	0	4	1
10801	68	1	0	0	0	1	1	0	0	1	0	0	0	0	0	2	4
10804	76	2	0	1	1	0	1	0	0	0	0	0	0	0	0	1	4
10805	67	2	0	0	0	0	1	1	0	0	1	0	0	0	0	2	2
10806	66	1	0	0	0	0	1	1	0	0	1	0	0	0	1	1	4
10807	68	2	0	0	0	1	1	1	0	0	0	0	1	1	0	3	2
10813	71	1	0	0	1	1	1	2	1	1	1	0	0	0	1	1	4
10814	74	1	0	0	0	0	1	0	0	1	1	0	0	0	0	1	4
10815	70	2	1	0	0	0	1	0	0	0	0	0	0	0	0	2	2
10817	72	2	1	0	1	1	#NULL!	0	0	0	1	0	0	0	0	2	2
10822	66	2	0	0	0	0	3	1	0	0	0	0	0	0	0	1	4
10823	66	1	0	0	0	0	1	0	0	0	1	0	0	0	1	1	4
10825	74	1	0	0	0	0	3	0	1	1	1	1	0	0	1	2	4
10826	66	2	0	0	0	#NULL!	#NULL!	0	0	0	0	0	0	0	0	2	2
10828	67	2	1	1	1	1	3	0	0	1	0	0	0	0	0	2	4
10829	66	2	0	0	0	1	1	0	0	0	0	0	0	0	1	2	4
10831	69	1	0	0	0	#NULL!	1	1	1	1	0	0	0	1	1	1	4
10832	66	2	0	0	0	0	1	0	0	0	0	0	0	0	1	2	4
10833	69	1	0	0	0	0	1	0	1	0	0	0	0	0	0	1	4
10834	71	2	0	0	0	0	1	0	0	0	1	0	0	0	0	3	2
10835	69	1	0	0	0	1	3	0	1	1	1	0	1	0	0	1	4
10842	66	2	0	0	1	1	1	#NULL!	0	0	0	0	0	0	0	3	4
10844	70	1	1	0	0	0	3	2	1	1	0	0	0	0	0	1	4
10849	66	1	0	0	0	0	1	0	1	1	1	0	0	0	0	2	4
10850	66	2	0	0	0	0	1	1	1	0	1	0	0	0	0	3	1
10851	84	2	1	1	0	#NULL!	3	1	0	0	0	0	0	0	0	2	3
10852	66	2	0	1	0	1	1	0	1	0	1	0	1	0	0	1	4
10856	66	2	0	1	0	0	1	0	0	0	0	0	0	0	1	1	4
10857	67	1	0	0	0	0	1	0	1	1	0	0	0	0	0	2	4
10858	66	2	0	0	0	0	2	0	0	0	1	1	0	0	0	2	3
10859	67	1	0	0	0	1	3	0	0	1	0	0	0	0	1	1	4
10863	66	2	0	0	0	1	2	0	0	0	1	0	0	0	0	2	4
10866	65	1	0	0	0	1	1	1	1	1	0	1	0	0	0	1	4
10867	65	2	0	0	0	1	1	#NULL!	0	0	1	1	0	0	1	1	3

10868	82	1	1	0	0	1	1	2	0	0	0	1	1	0	0	2	4
10869	78	2	0	0	0	1	1	0	0	0	0	1	1	0	0	1	1
10870	71	1	0	0	0	1	3	1	0	1	1	1	0	0	0	1	4
10873	79	1	1	0	0	1	1	0	0	1	1	0	1	0	1	2	4
10874	74	2	0	0	1	1	1	1	0	0	0	1	0	0	0	2	4
10875	72	1	0	0	1	1	1	0	0	1	0	0	0	0	1	1	4
10876	66	1	0	0	1	0	1	0	1	1	0	0	0	0	1	1	4
10877	71	2	0	1	0	0	1	1	0	0	0	1	0	0	0	2	4
10878	79	1	0	0	1	1	1	1	0	0	1	1	0	0	0	4	1
10879	77	2	0	0	1	0	3	1	0	0	0	0	0	0	0	3	3
10881	71	2	0	0	0	0	1	0	0	0	1	1	0	0	0	3	4
10882	83	1	1	0	0	1	1	0	1	1	1	0	0	0	0	1	4
10883	77	2	0	0	0	1	3	0	0	0	1	1	0	0	0	3	3
10887	67	1	1	0	0	0	3	2	0	0	1	1	0	0	0	4	1
10889	75	1	1	0	0	#NULL!	1	0	1	1	1	0	0	0	0	3	4
10890	71	2	0	0	0	#NULL!	1	0	1	0	0	0	0	0	0	2	3
10891	80	2	0	1	0	0	1	1	0	0	0	0	0	0	0	4	1
10894	68	2	0	1	0	0	1	#NULL!	#NULL!	#NULL!	0	0	#NULL!	0	0	2	2
10899	70	1	0	0	1	0	1	1	0	1	0	0	0	0	0	1	4
10901	83	2	1	1	1	1	1	1	0	0	0	0	0	1	0	3	4
10903	68	1	0	0	0	1	1	0	0	1	0	0	0	0	0	2	4
10907	69	1	0	0	0	1	1	#NULL!	#NULL!	#NULL!	0	0	#NULL!	1	1	1	4
10910	77	1	0	0	0	1	1	1	1	0	1	1	0	0	0	1	4
10911	72	2	0	0	0	1	1	0	1	0	1	1	0	0	0	1	4
10912	74	2	0	1	0	0	1	1	0	0	1	0	0	0	0	2	4
10914	69	1	0	0	1	1	1	1	1	1	0	0	0	0	1	3	4
10916	68	2	1	0	0	1	2	0	0	0	0	0	1	0	0	4	1
10917	66	2	0	0	1	1	3	1	0	0	0	1	1	0	0	3	4
10918	82	2	0	1	0	0	1	0	0	0	1	1	0	0	1	3	4
10919	69	1	0	0	0	1	1	1	0	0	0	0	0	0	0	1	4
10920	65	2	0	1	0	1	1	0	0	0	1	1	1	0	0	1	4
10931	65	2	0	0	1	1	1	1	0	0	1	0	0	0	0	2	3
10932	65	1	0	#NULL!	0	#NULL!	1	#NULL!	#NULL!	#NULL!	0	0	#NULL!	0	0	2	4
10933	71	1	0	0	0	0	1	1	1	1	0	0	0	0	0	1	4
10934	67	2	0	0	0	1	1	1	0	0	0	0	0	0	0	2	1
10935	66	1	0	0	0	1	3	2	1	1	1	1	0	0	0	1	4
10937	78	2	1	0	1	#NULL!	#NULL!	1	0	#NULL!	0	0	0	0	0	4	2
10941	70	1	0	0	0	0	1	0	0	0	1	1	1	0	0	1	4
10943	65	1	0	0	0	0	1	0	1	0	1	0	0	0	0	1	4

10948	69	1	0	1	0	#NULL!	1	0	1	1	1	0	0	0	0	1	4
10949	73	1	0	0	0	0	2	0	0	0	0	0	0	0	0	1	4
10950	69	2	0	0	0	0	1	0	0	0	0	0	0	0	0	2	2
10952	70	1	0	0	0	#NULL!	2	#NULL!	#NULL!	#NULL!	1	1	#NULL!	0	0	3	4
10954	84	2	0	#NULL!	0	1	1	2	0	0	1	1	0	0	0	1	4
10956	66	2	0	0	1	1	3	2	0	0	0	0	0	0	0	3	4
10961	65	2	1	0	0	1	1	#NULL!	#NULL!	#NULL!	1	0	0	0	0	2	2
10962	79	2	0	1	0	0	1	0	0	0	0	1	0	0	1	2	2
10964	71	2	0	0	1	0	1	0	0	0	1	1	0	0	0	3	4
10969	68	2	0	1	0	0	1	0	0	0	1	1	0	0	0	2	2
10973	65	2	0	0	0	0	1	0	0	0	1	1	0	0	0	2	1
10974	72	2	0	0	0	1	1	0	0	0	1	0	1	0	0	2	2
